# Global prevalence and associated risk factors of scoliosis in children and adolescents: a systematic review and meta-analysis

**DOI:** 10.1186/s12889-025-24905-4

**Published:** 2025-10-28

**Authors:** Shoujian Wang, Miaoxiu Li, Jun Ren, Jiming Tao, Min Fang, Lingjun Kong

**Affiliations:** 1 Department of Tuina, Shuguang Hospital, Shanghai University of Traditional Chinese Medicine, Shanghai, China; 2 Institute of Tuina, Shanghai Institute of Traditional Chinese Medicine, Shanghai, China

**Keywords:** Scoliosis, Children and Adolescents, Prevalence, Risk Factors, Meta-Analysis

## Abstract

**Introduction:**

Synthesised data on the global prevalence of scoliosis in children and adolescents and its associated risk factors remain scarce. This systematic review aimed to estimate the global prevalence of scoliosis in children and adolescents and identify associated risk factors in children and adolescents.

**Methods:**

We conducted a systematic review and meta-analysis, searching nine electronic databases for observational population-based studies on scoliosis in children and adolescents published until June 02, 2024, without language or geographical restrictions. We estimated the global prevalence using the DerSimonian-Laird random-effects model with Free-Tukey double arcsine transformation and stratified prevalence through prespecified subgroup analyses. Fixed-effects models were used for consistent risk factors across studies, while random-effects models were applied for factors with high heterogeneity.

**Results:**

We initially identified 12,339 records, of which 239 studies comprising 46,565,512 participants were included. The estimated global prevalence of scoliosis in children and adolescents was 1.65% (95% CI 1.38–1.94), varying from 0.05% in Ecuador to 16.67% in Romania. We found higher prevalence in European (2.88%) and Americas (2.54%) than other regions (Eastern Mediterranean 1.22%, South-East Asia 1.35%, Western Pacific 1.35%, and African 1.46%). The higher prevalence was in high-latitude regions (2.96% vs mid-latitude 1.62% vs low-latitude 1.73%). The prevalence in girls was nearly double that in boys (1.76% vs 0.87%), with a steady increase observed from ages 7 to 16 years, peaking at 12–14 years for girls and 15–16 years for boys. The prevalence in adolescents (10–18 years) was nearly four times higher than that in children (6–9 years) (1.13% vs 0.26%). Idiopathic scoliosis in children and adolescents constituted the majority of cases, predominantly presenting as mild scoliosis (Cobb angle 10°-19°, 86.65%) with the single thoracolumbar curve being the most common subtype (36.67%). Additionally, the prevalence may increase since 2020 year (3.02%). Scoliosis in children and adolescents was significantly associated with sex (girls, odds ratio 2.16, 95% CI 2.01–2.33), family history (1.92, 95% CI 1.47–2.50), sedentary behavior (≥ 11 h, 1.64, 95% CI 1.20–2.25), poor sitting posture (3.48, 95% CI 2.85–4.24), short outdoor time (< 1 h, 1.32, 95% CI 1.10–1.59), long screen time (≥ 2 h, 2.74, 95% CI 2.29–3.28), unilateral physical activity (1.36, 95% CI 1.20–1.55), and low BMI (1.15, 95% CI 1.08–1.22).

**Conclusions:**

Scoliosis in children and adolescents remains highly prevalent globally. Our findings can help to guide epidemiological characteristics and development of tailored preventive and therapeutic strategies, ultimately enhancing primary prevention and controlling disease progression.

**Trial registration:**

CRD42024584704

**Supplementary Information:**

The online version contains supplementary material available at 10.1186/s12889-025-24905-4.

## Introduction

Scoliosis in children and adolescents is a three-dimensional spinal deformity characterized by a coronal curvature (Cobb angle ≥ 10°) [1]. Idiopathic scoliosis is the most prevalent form, accounting for approximately 75–85% of all cases [2, 3]. This condition not only results in physical deformity but also adversely affects mental health, contributing to anxiety and depression among children and adolescents [4]. While many children and adolescents with scoliosis are asymptomatic, the condition can progress, particularly during the rapid growth spurt typically occurring between ages 11 and 14 years [5, 6]. Untreated, scoliosis can cause severe trunk deformities that restrict chest capacity, functional biomechanics, exercise tolerance, general fitness, and work ability, all impacting quality of life [7, 8].

The prevalence of scoliosis in children and adolescents remains uncertain, with reported rates ranging from 0.52% to 14% [9, 10]. This variability may primarily stem from differences in sampling methods and sample sizes (e.g., 0.52% in a large-scale randomized survey *vs* 3.14% in a smaller non-randomized survey), geographic regions (e.g., 4.62% in Thailand *vs* 2.42% in China), and the inclusion of high-risk subgroups (e.g., up to 14% among adolescent athletes) [9–14]. It detailed regional or country-specific breakdowns are lacking, limiting targeted preventive strategies. Furthermore, although previous studies have only suggested several potential risk factors that might be associated with scoliosis in children and adolescents, the overall quality of evidence remains limited [15, 16]. Most findings are based on observational cross-sectional studies without rigorous adjustment for confounding variables. Moreover, existing meta-analyses have focused on specific countries or regions, limiting their global generalizability [17, 18]. Accurate prevalence estimates of the global prevalence of scoliosis in children and adolescents are essential for updating disease epidemiology, including for research and development of preventive and therapeutic strategies based on risk factors, which could enhance primary prevention and control disease progression.

Therefore, the primary aim of our systematic review and meta-analysis was to estimate the global prevalence of scoliosis in children and adolescents. The secondary aim was to identify risk factors associated with the scoliosis in children and adolescents.

## Methods

### Search strategy and selection criteria

This systematic review was registered with International Prospective Register of Systematic Reviews (PROSPERO, registration number: CRD42024584704) and conducted in accordance with the Preferred Reporting Items for Systematic Reviews and Meta-Analyses (PRISMA) guidelines [19].

We searched electronic databases, including Medline (Ovid platform), Embase (Ovid platform), Web of Science, Global Index Medicus, Chinese Biomedical Literature Database, China Knowledge Resource Integrated Database, Weipu Database for Chinese Technical Periodicals, and Wanfang Data Information for observational population-based studies published from inception to June 02, 2024. Search terms included combinations of keywords such as scoliosis, spinal curvature, vertebral lateral curvature, adolescent, children, prevalence, epidemiology, and risk factors, linked using Boolean operators (OR/AND). No language or geographical restrictions were applied. The detailed electronic search strategy can be showed in Appendix S1 (pp 3–6). We also screened reference lists from relevant systematic reviews and guidelines for additional eligible studies.

Inclusion criteria comprised observational studies meeting the following: 1) observational population-based designs, including cohort, cross-sectional, or case–control studies; 2) reports on the prevalence of scoliosis in children and adolescents (aged 18 years or younger) diagnosed with a lateral curve of at least 10° via X-rays, suspected scoliosis in children and adolescents based on positive primary screening results (e.g., Adam’s test or angle of trunk rotation), or risk factors related to scoliosis. Additionally, studies involving mixed populations were eligible for inclusion if adolescents and children with scoliosis could be clearly distinguished and analyzed separately. For prevalence analyses, only population-based cohort or cross-sectional studies were eligible. Case–control studies were included solely for the assessment of potential risk factors and were not used in any prevalence meta-analysis.

Exclusion criteria included: 1) studies focused on specific subgroups not representative of the general population (e.g., adolescents with specific diseases or young athletes); 2) studies with fewer than 50 participants [20–22].

We compared similar studies for potential data source overlap and selected the one with the most comprehensive results or largest sample size. Following duplicate removal, two independent reviewers screened titles, abstracts, and full texts of identified records using Covidence software (Veritas Health Innovation, Melbourne, Australia; available at www.covidence.org). Non-English or Chinese studies were translated using Google Translate, with assistance sought from native speakers when necessary. Disagreements were resolved through discussion.

### Data analysis

Two reviewers independently extracted study characteristics using a predefined data extraction form, capturing details such as country, year of publication, year of investigation, study type, sample origin, sample size, diagnostic methods, confirmed case counts, age range, and proportion of girls. The methodological quality of cross-sectional studies was assessed using the Joanna Briggs Institute (JBI) Critical Appraisal Checklist, while cohort and case–control studies were evaluated using the Newcastle–Ottawa Scale (NOS) [23, 24]. The reviewers resolved disagreements through discussion.

We proportionally corrected X-rays-confirmed cases based on positive initial screenings, the number of referral cases, and raw confirmed case counts. We used the Freeman-Tukey double arcsine transformation to stabilize the variance of the raw prevalence from included studies before estimating the pooled prevalence of scoliosis in children and adolescents [25]. After the transformation, all prevalence estimates were calculated as odds ratios (ORs) with 95% confidence intervals (CIs) by using DerSimonian Laird random-effects model [26]. Heterogeneity was assessed using the *I*^2^ index and Cochran’s Q test. The meta-analysis encompassed both primary screening positivity rates and X-rays-confirmed prevalence rates. Influence diagnostics, leave-one-out analyses, and forest plot inspections were employed to identify outliers, followed by sensitivity analyses to exclude those disproportionately affecting prevalence estimates. Publication bias was assessed using funnel plots, Egger’s test, Begg’s test, and the trim-and-fill method [27–29].

Prespecified subgroup analyses explored variations in prevalence across investigation periods (before 2000 *vs* 2000–2009 *vs* 2010–2019 *vs* 2020 and later), sexes (girls *vs* boys), WHO-designated regions (African region *vs* region of the Americas *vs* Eastern Mediterranean region *vs* European region *vs* South-East Asia region *vs* Western Pacific region), World Bank income categories (high income *vs* upper-middle income *vs* low-middle income), latitude (low *vs* middle *vs* high), body mass index (BMI) (underweight *vs* normal *vs* overweight/obesity) which was classified according to WS/T 456–2014 [30], family history (positive family history *vs* negative family history), daily physical exercise (< 1 h *vs* ≥ 1 h), daily sleep duration (< 8 h *vs* ≥ 8 h), daily screen time (< 2 h *vs* ≥ 2 h), sitting posture (proper *vs* poor), desk height (adjustable *vs* non-adjustable), type of physical exercise (unilateral *vs* bilateral), and each country worldwide involved in the pooling analysis. For subgroup analysis by geomagnetic latitude (GLAT), study locations were classified into low (GLAT < 30°), mid (30°–60°), and high (GLAT > 60°) latitude regions based on geographic coordinates from Google Earth [31]. In multi-region studies, classification was determined by the primary study location or, if unclear, the lead author’s institutional location. In addition, a post hoc subgroup analyses was performed for age group (children, 6–9 years *vs* adolescents, 10–18 years). This comprehensive approach aimed to examine variations in scoliosis prevalence among children and adolescents across these subgroups.

Additionally, we reported distributions based on aetiological types (idiopathic *vs* non-idiopathic scoliosis), severity (Cobb angle 10°−19° *vs* 20°−39° *vs* ≥ 40°), and curve patterns (single thoracic curve *vs* single thoracolumbar curve *vs* single lumbar curve *vs* double curve *vs* triple curves), providing valuable insights into the characteristics of scoliosis in children and adolescents cases.

A subset of eligible studies assessed potential risk factors associated with scoliosis using regression analysis. We extracted definitions and effect size estimates (e.g., ORs and 95% CIs) for each risk factor. For studies lacking these estimates, we calculated them from original 2 × 2 table data using a built-in function in metabin. Among the reported factors associated with scoliosis in children and adolescents, we calculated ORs with 95% CIs using a fixed-effects model for risk factors assessed in two or more studies. In cases of high heterogeneity in definitions, a random-effects model was employed. Statistical significance was defined as a *p*-value < 0.05. All analyses were conducted using Stata (version 15.0) and R (version 4.2.1).

### Role of the funding source

The funder of the study had no role in study design, data collection, data analysis, data interpretation, or writing of the report. The corresponding author had full access to all the data in the study and had final responsibility for the decision to submit for publication.

## Results

**Fig. 1:**
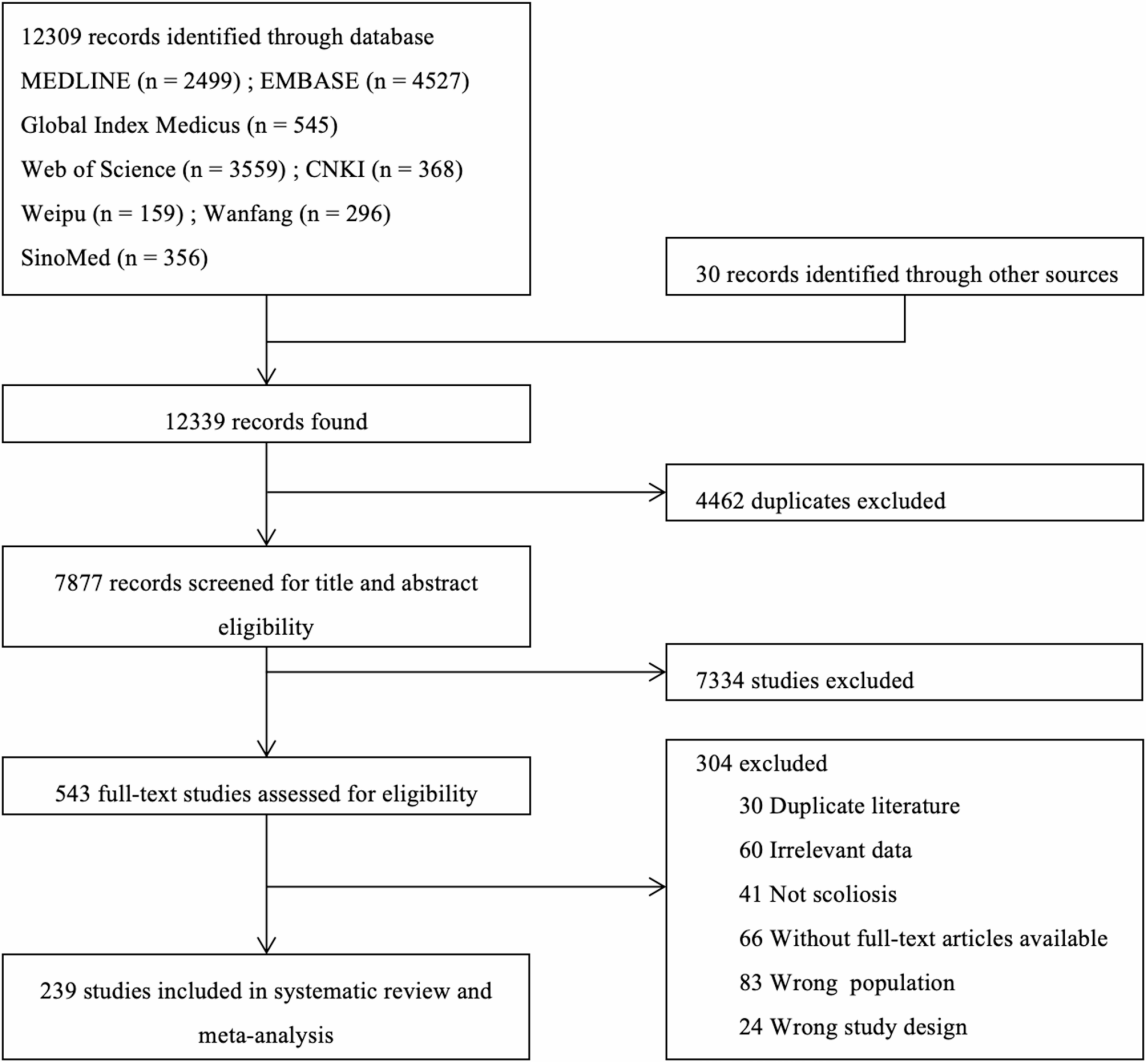
Study selection

We initially identified 12,339 records through our literature search, including 12,309 from databases and 30 from other sources (Fig. 1). After removing 4462 duplicates, we screened titles and abstracts, resulting in the exclusion of 7334 irrelevant records. The full texts of 543 articles were then reviewed, leading to the inclusion of 239 studies comprising 46,565,512 participants in our systematic review and meta-analysis. Among these, 219 were cross-sectional studies, 14 were case–control studies, and 6 were cohort studies. The majority of studies (73.64%) received a quality score of at least 5, with detailed characteristics and quality assessments provided in Appendix S3 (pp 20–26).

**Fig. 2:**
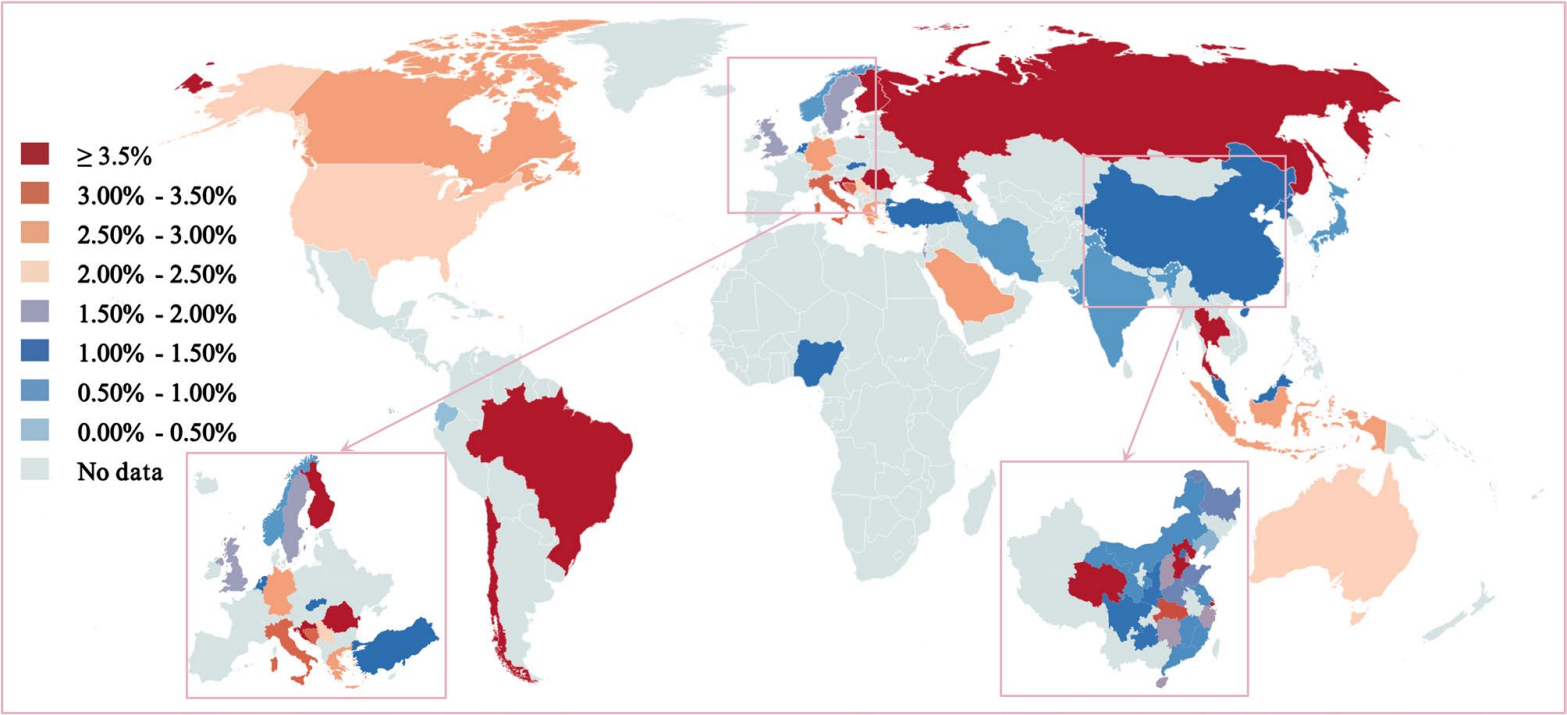
Geographical location of the 150 included articles reporting the prevalence of scoliosis in children and adolescents. The world map shows the prevalence of scoliosis in children and adolescents across 33 included countries, with a color gradient from blue (low prevalence) to red (high prevalence)

A total of 186 studies (7,933,594 participants) from 39 countries reported the prevalence of suspected scoliosis in children and adolescents based on primary screenings, yielding an estimated prevalence of 5.80% (95% CI 4.83–6.86) (Appendix S4, pp 27–29). Additionally, 150 studies (42,901,789 participants) from 33 countries reported the prevalence of scoliosis in children and adolescents diagnosed via X-rays, revealing a global estimated prevalence of 1.65% (95% CI 1.38–1.94) (Table 1; Appendix S5, pp 30–31). Stratified analysis indicated an increase in prevalence, from 1.86% (95% CI 1.37–2.42) before 2000 to 3.02% (95% CI 1.53–4.99) in 2020 and later (Table 1; Appendix S14.1, p 47). Geographically, the highest prevalence of adolescent scoliosis was observed in the European Region (2.88%), while the Eastern Mediterranean Region recorded the lowest prevalence (1.22%) (Table 1; Appendix S14.4, p 50). The prevalence varied significantly among countries, ranging from 0.05% in Ecuador to 16.67% in Romania (Fig. 2; Appendix S5, pp 30–31). Notably, the largest number of studies (79, 52.67%) on prevalence of scoliosis in children and adolescents were conducted in China, where the estimated prevalence was 1.66% (95% CI 1.31–2.04), peaking in Hebei Province at 4.96% and reaching a low of 0.33% in Liaoning Province (Appendix S6, pp 32–33). No significant differences in prevalence were found across regions classified by World Bank income categories; however, variations were noted. The prevalence was 1.97% in high-income countries, 1.54% in upper-middle-income countries, and 0.81% in low-middle-income countries (Table 1; Appendix S14.5, p 51).

**Table 1:**
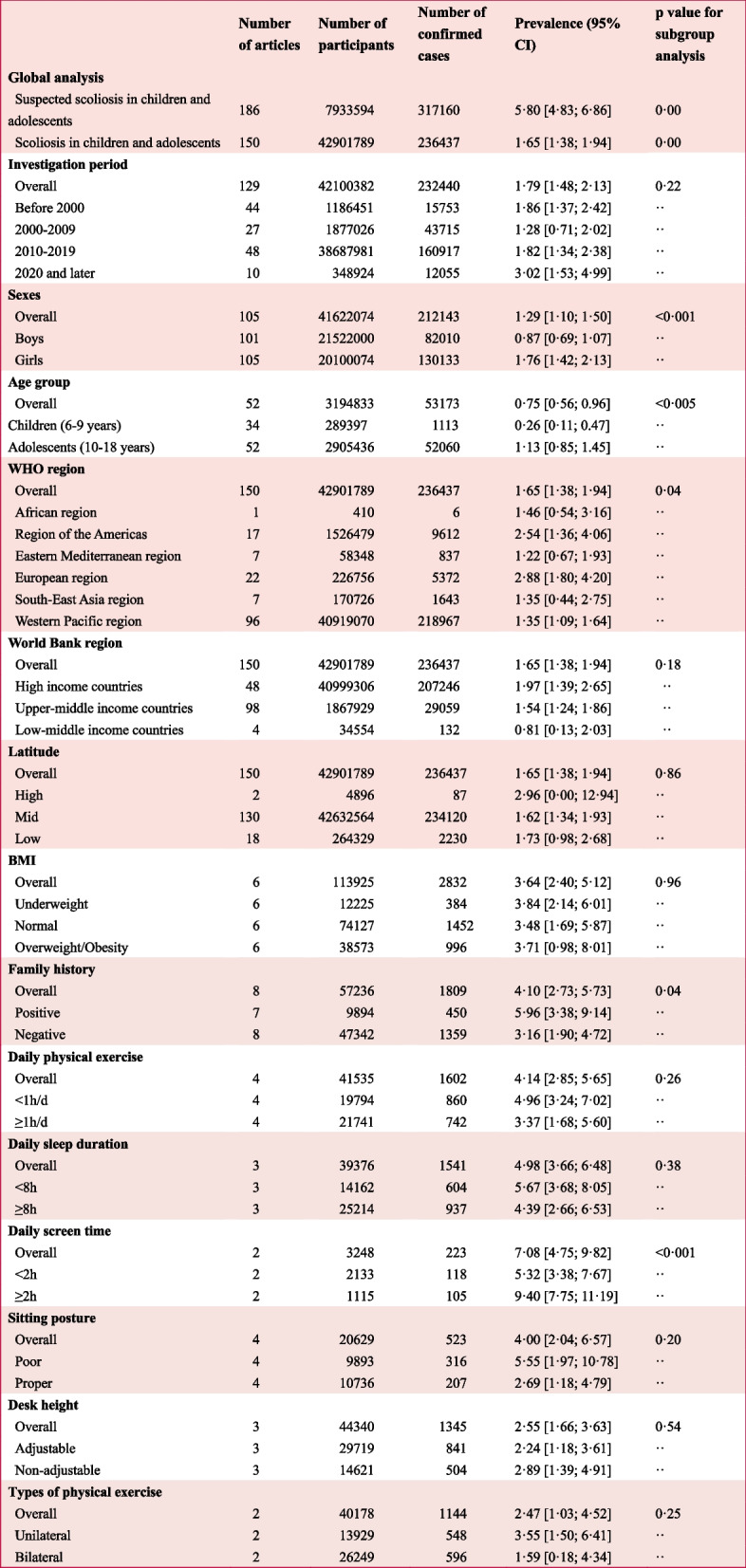
Estimated prevalence of scoliosis in children and adolescents in various subgroups (≤ 18 years)

We estimated the prevalence using the DerSimonian-Laird random-effects model with Free-Tukey double arcsine transformation and stratified prevalence through prespecified subgroup analyses

**Fig. 3:**
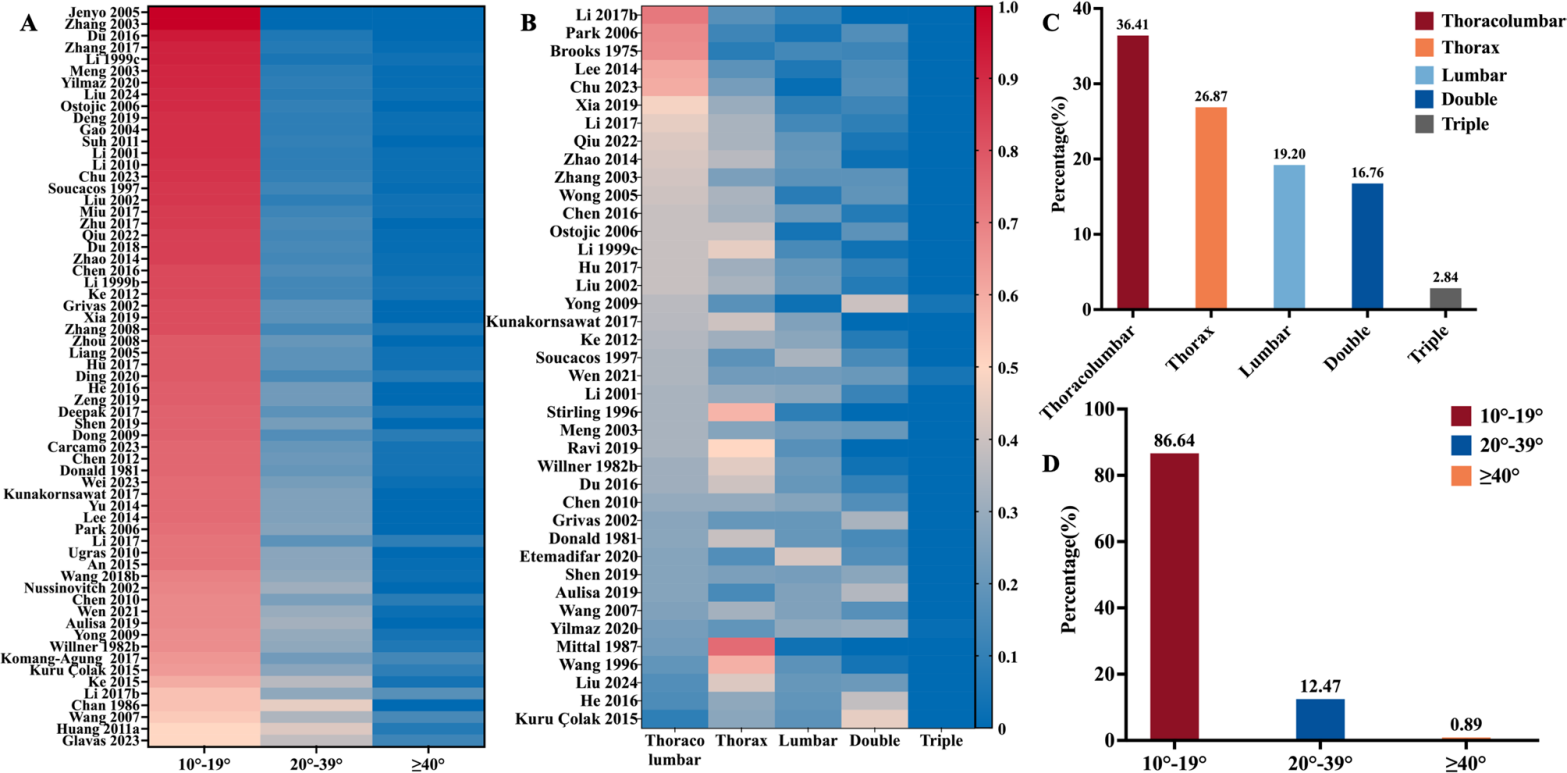
Summary findings of scoliosis in children and adolescents severity and curve type. Figure 3 includes two heatmaps (**A** and **B**) and two bar charts (**C** and **D**) illustrating the characteristics of adolescent scoliosis. Heatmaps A and B display scoliosis severity (10°−19°, 20°−39°, and ≥ 40°) and curve types (thoracolumbar, thoracic, lumbar, double curves and triple curves), respectively, across individual studies. Each row represents a study, with color intensity from blue (low proportion) to red (high proportion). Bar charts C and D show the overall distribution of scoliosis curve types and severity grades, respectively. The height of each bar indicates the cumulative proportion within each category. Distinct color schemes were applied in panels C and D to represent categorical variables, while panels A and B used a shared gradient scale to visualize relative proportions within each study

**Fig. 4:**
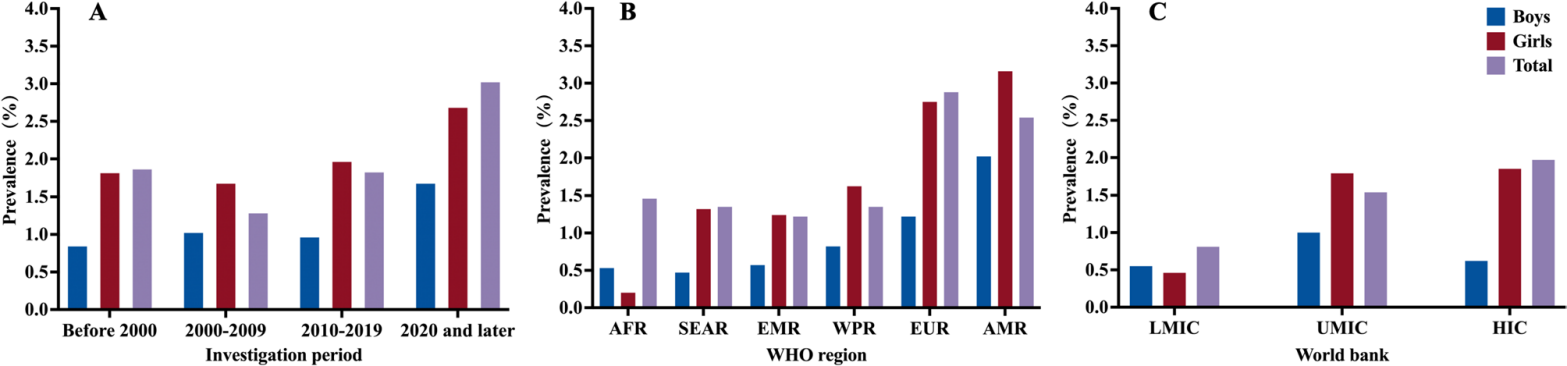
Sex-Specific prevalence of scoliosis in children and adolescents across investigation period, WHO region, and World Bank region. The bar charts show three outcomes: **A** prevalence across four investigation periods (Before 2000, 2000–2009, 2010–2019 and 2020 and later); **B** prevalence by WHO region (AFR, SEAR, EMR, WPR, EUR, AMR); **C** prevalence by income level per World Bank classification (HIC, UMIC, LMIC). Each bar color represents a different group: overall population (purple), boys (blue), and girls (red), with bar height indicating the estimated prevalence. Abbreviations: WHO, World Health Organization; AFR, African Region; AMR, Region of the Americas; SEAR, South-East Asia Region; EUR, European Region; EMR, Eastern Mediterranean Region; WPR, Western Pacific Region; WB, World Bank; HIC, high-income countries; LMIC, low- and middle-income countries; UMIC, upper middle-income countries

**Fig. 5:**
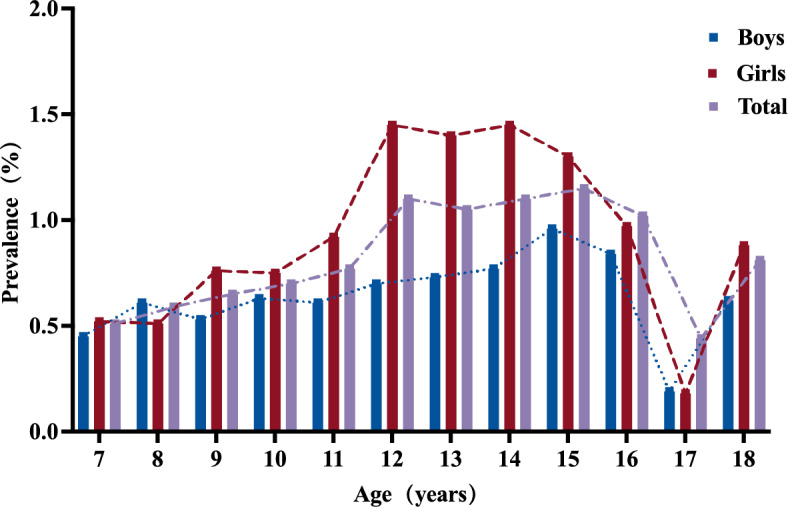
Age- and Sex-specific prevalence of scoliosis in children and adolescents. Each bar color represents a different group: overall population (purple), boys (blue), and girls (red), with the height of each bar indicating the estimated prevalence of scoliosis across age groups from 7 to 18 years. The lines represent the variation in prevalence across ages for each group, with the purple dashed line showing the overall population, the blue dotted line representing boys, and the red dashed line representing girls

Among 69 studies focusing on aetiological types of scoliosis in children and adolescents, the majority (93.60%) were classified as idiopathic scoliosis (Appendix S11, p 43). Severity was reported in 85 studies, indicating that mild scoliosis (Cobb angle 10°−19°) was the most common (86.65%), followed by moderate scoliosis (Cobb angle 20°−39°) at 12.47%, and severe scoliosis (Cobb angle ≥ 40°) at 0.89% (Fig. 3A, 3D; Appendix S12, p 44). Curve patterns of scoliosis were reported in 42 studies, revealing that the most frequent subtype was the single thoracolumbar curve (36.41%), followed by the single thoracic curve (26.87%), single lumbar curve (19.20%), double curves (16.76%), and triple curves (2.84%) (Fig. 3B, 3 C; Appendix S13, p 45–46). The prevalence of scoliosis in children and adolescents in girls (1.76%) was nearly double that of boys (0.87%) (Table 1; Appendix S14.2, p 48). This sex disparity was evident across various temporal surveys (e.g., 2010–2019: girls *vs* boys, 1.96% *vs* 0.96%), WHO regional subdivisions (e.g., European Region: girls *vs* boys, 2.75% *vs* 1.22%), and economic regions defined by the World Bank (e.g., high-income countries: girls *vs* boys, 1.85% *vs* 0.62%) (Fig. 4A-C; Appendix S7-9, pp 34–39). When stratified by age group, the prevalence of scoliosis in adolescents (1.13%) was early four times higher than that in children (0.26%) (Table 1; Appendix S14.3, p 49). Moreover, the prevalence generally increased with age from 7 to 16 years, peaking at ages 12–14 years for girls and 15–16 years for boys (Fig. 5; Appendix S10, pp 40–42). Children and adolescents from high-latitude regions exhibited higher prevalence compared to those from mid- or low-latitude regions (2.96% *vs* 1.62% *vs* 1.73%). Additionally, children and adolescents with a positive family history showed a higher prevalence than those without (5.96% *vs* 3.16%). Those with more than 2 h of daily screen time had significantly higher prevalence than those with less than 2 h (9.40% *vs* 5.42%). Furthermore, children and adolescents engaging in unilateral physical activities may have higher prevalence compared to those engaging in bilateral activities (3.55% *vs* 1.59%), and those exercising for less than 1 h daily may have a higher prevalence than those exercising for more than 1 h (4.96% *vs* 3.37%). Poor sitting posture, which is defined as exhibiting at least one of the following during academic tasks: desk-related forward trunk flexion, asymmetrical sitting (lateral deviation), cross-legged position, or kyphotic alignment, may also be associated with a higher prevalence (5.55% vs 2.69%) [32]. The prevalence of scoliosis in children and adolescents did not differ significantly by BMI, daily sleep duration, or desk height. Further details on subgroup results were provided in Table 1 and Appendix S14 (pp 47–54).

We categorized the effects of 14 factors associated with scoliosis in children and adolescents into three groups: individual characteristics, daily behavioral habits, and household aspects (Table 2). Notably, sex (girls), family history, poor sitting posture, unilateral physical activity, sedentary behavior (≥ 11 h), short outdoor time (< 1 h), long screen time (≥ 2 h), and low BMI may be consistently linked to a higher prevalence of scoliosis in children and adolescents (Table 2). Detailed forest plots results from the individual studies contributing to these meta-analyses were available in Appendix S15 (pp 55–62).

**Table 2:**
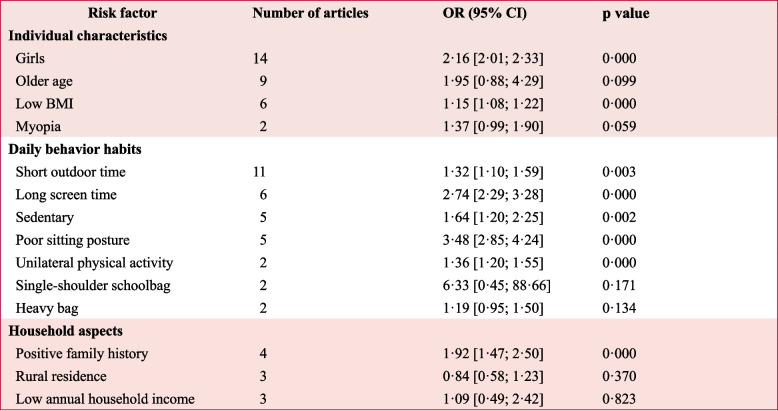
Synthesised effect estimates of three groups of risk factors of scoliosis in children and adolescents

Fixed-effects models were used for consistent risk factors across studies, while random-effects models were applied for factors with high heterogeneity. a random-effects meta-analysis. The association between age and adolescent scoliosis prevalence suggests a higher prevalence among older children than younger ones, but does not imply an increasing trend over time, given the cross-sectional nature of the included studies. OR = odds ratio, BMI = body mass index

A sensitivity analysis was performed using a series of tests (Appendix S16, pp 63–68), revealing that the estimated prevalence of scoliosis in children and adolescents ranged from 1.63% (95% CI 1.36%−1.92%) to 1.69% (95% CI 1.41%−2.00%) after removing outliers from the overall dataset. Importantly, no single study significantly influenced the overall prevalence. We identified asymmetry in the funnel plot, indicating an excess of studies with large effect sizes and low precision, a finding further confirmed by Egger’s test. The trim-and-fill method suggested that 31.2% of studies may remain unpublished, adjusting the effect size to 0.48% (95% CI 0.30–0.71), indicating potential publication bias (Appendix S17, pp 69–70).

## Discussion

This systematic review provided the most comprehensive and up-to-date estimate of the global prevalence of scoliosis in children and adolescents based on available data from the past five decades, estimating a global prevalence of 1.65%, based on data from 42.9 million children and adolescents in 150 studies spanning 33 countries. We observed substantial variability in prevalence across countries, ranging from 0.05% in Ecuador to 16.67% in Romania. We also found higher prevalence in European and Americas than other regions. The higher prevalence was in high-latitude regions than low- or mid-latitude regions. Additionally, the prevalence in girls was nearly double that in boys, with a steady increase observed from ages 7 to 16 years, peaking at 12–14 years for girls and 15–16 years for boys. Idiopathic scoliosis in children and adolescents constituted the vast majority of cases, predominantly presenting as mild scoliosis (Cobb angle 10°−19°), with the single thoracolumbar curve subtype being most common. Since 2000, the prevalence has shown an obvious increasing trend. We extensively explored potential risk factors for scoliosis in children and adolescents, revealing that higher prevalence was found among individuals with risk factors such as sex (girls), family history, poor sitting posture, unilateral physical activity, sedentary behavior (≥ 11 h), short outdoor time (< 1 h), long screen time (≥ 2 h), and low BMI.

This study has several key strengths. First, our comprehensive search strategy had no language or geographical restrictions, ensuring broad applicability of our findings across diverse settings. Second, we proportionally corrected X-ray-confirmed cases based on positive initial screenings, the number of referral cases, and raw confirmed case counts, minimizing the potential underestimation of prevalence due to dropouts at the primary screening stage. Third, our detailed subgroup analyses, based on well-specified characteristics, provided valuable insights into the nature of scoliosis cases without producing misleading prevalence estimates. Additionally, we thoroughly explored possible risk factors of scoliosis in children and adolescents, which could promote the development of preventive and therapeutic strategies of scoliosis in children and adolescents. These strengths could increase the confidence for our results.

Previous studies have reported a wide range of the prevalence for scoliosis in children and adolescents, from 0.05% to 16.67%. A prior review estimated a global prevalence of 3.1%, but our more comprehensive analysis suggests a more accurate prevalence of 1.65% [33]. This is based on a larger dataset, encompassing 42.9 million children and adolescents across 33 countries, nearly five times the sample size of earlier studies. Our study also applied stricter inclusion criteria, focusing on cases with a curvature ≥ 10° confirmed via X-rays, and limited the analysis to children and adolescents aged 18 or younger. Furthermore, our more thorough sensitivity analyses, including influence diagnostics, leave-one-out analyses, and forest plot inspections, confirmed the robustness and stability of our findings. Importantly, our estimates align with previous studies: for example, Li et al. reported a prevalence of 1.7% for adolescent idiopathic scoliosis, and Xu et al. found that the prevalence in China was 1.2%, peaking at ages 13–14 years for girls and 15–16 years for boys [33, 34]. Cao et al. also observed a nearly twofold higher prevalence of idiopathic scoliosis in girls compared to boys [35].

The prevalence of scoliosis in children and adolescents has varied over time and across geographical regions. We found that the prevalence has risen over the past three decades, especially after 2020. This increase may be attributable to the implementation of screening programs in more countries, which have led to higher case detection rates. In terms of regional variations, the higher prevalence in the Europe and the Americas regions may be linked to the higher proportion of small-sample studies (n < 2000), potentially introducing bias. We also observed a trend toward higher scoliosis prevalence in high-latitude regions. One possible explanation is that reduced sunlight exposure at higher latitudes may affect hormonal regulation, including melatonin secretion and vitamin D metabolism, which in turn could influence the timing of puberty [36, 37]. Grivas TB et al. similarly reported a positive association between scoliosis prevalence and geographic latitude [38]. However, the inclusion of only two studies from high-latitude countries may limit the persuasiveness of this finding. Therefore, this hypothesized pathway remains speculative, and further research is needed to clarify the potential role of latitude-related biological mechanisms in scoliosis epidemiology. Country-specific variability, ranging from 0.05% in Ecuador to 16.67% in Romania, could be influenced by differences in economic, educational, and social development, which affect the extent of school and community-based scoliosis screening programs.

Our findings confirm that children and adolescents with a family history of scoliosis have a higher prevalence, consistent with previous studies highlighting the strong genetic predisposition for adolescent idiopathic scoliosis [39–41]. Additionally, we identified other significant risk factors, including sedentary behavior, poor sitting posture, limited outdoor time, and prolonged screen time. These findings suggest that academic pressures may limit outdoor activities for children and adolescents, leading to increased sedentary behavior. The COVID-19 pandemic has likely exacerbated this trend, as online learning has led to significantly increased screen time [42, 43]. Furthermore, unilateral physical activities were found to increase the prevalence of scoliosis, likely due to muscle asymmetries that disrupt spinal posture and movement control [44]. The relationship between BMI and scoliosis remains uncertain; while we found that low BMI may be a risk factor, we did not observe significant differences in prevalence among children and adolescents with varying BMI levels. Roslyn et al. reported that patients with idiopathic scoliosis are more likely to have a low BMI, whereas Li et al. found that overweight children and adolescents may be more prone to developing scoliosis [33, 45].

Despite our standardization of diagnostic criteria, substantial heterogeneity remained in our meta-analysis results. To address this, we conducted subgroup analyses, which revealed significant differences based on the Cochran Q test (p-values < 0.05) and consistently high *I*^2^ values (> 95%) in nearly all subgroups, indicating that the sources of heterogeneity were not fully explained. Previous research has shown that high sample sizes can lead to increased *I*^2^ heterogeneity due to narrower study-specific confidence intervals [46]. Thus, high sample sizes should not be viewed as definitive measures of heterogeneity. Several factors, such as variations in screening methods and operator-dependent procedures, likely contributed to this heterogeneity. For instance, Adam’s forward bending test is influenced by the degree of spinal curvature and the operator’s experience, potentially leading to variability in both referrals cases and those confirmed by X-rays [47, 48]. Addressing these inconsistencies is essential for refining our understanding of adolescent scoliosis prevalence and its associated risk factors, and it will guide future research directions.

This meta-analysis has some limitations that should be addressed in future studies. The methodological quality of the included studies varied, which may have affected the reliability of our prevalence estimates. We estimated ORs for certain risk factors based on a limited number of studies, which may have restricted our ability to fully synthesize the associations between scoliosis in children and adolescents and these selected factors. Although our study covered all WHO regions and included children and adolescents aged 6 to 18, the limited data from certain countries and age groups may reduce the generalizability of our findings. In particular, the inconsistent representation across geographic areas, with some countries, such as those in Africa, high-latitude regions, and low-income settings, having only small-sampled studies or none at all, may pose challenges to our conclusions. Additionally, fewer studies examined the 6 and 16 to 18 age groups, limiting our ability to provide age-specific prevalence estimates. The cross-sectional nature of most studies further constrained our ability to assess trends over time or to evaluate the progression of scoliosis and the long-term impacts of associated risk factors. Moreover, many included studies did not clearly distinguish idiopathic from non-idiopathic scoliosis when reporting risk factors. Although we attempted to contact study authors for clarification, limited responses prevented us from performing type-specific analyses. This may have introduced heterogeneity, and highlights the need for future studies to explicitly differentiate scoliosis subtypes in risk factor research. Despite these limitations, our findings offer valuable insights into the global prevalence of scoliosis in children and adolescents and contribute to a deeper understanding of its worldwide distribution.

Future studies should make improvements by employing more robust sampling methods, addressing low response rates, and providing detailed analyses of risk factors. Multi-factor analysis is especially needed to evaluate the combined effects of various risk factors on scoliosis. Early scoliosis screening should continue to be emphasized, particularly for girls and those with a family history. Schools, families, and society should prioritize adolescent physical activity, ensuring at least 60 min of moderate exercise daily [49]. Guidance on proper sitting posture and screen time management is also critical. Scoliosis in children and adolescents remains a global health issue, and in low-income countries with limited resources, cost-effectiveness studies are necessary to inform policies regarding systematic screening for scoliosis.

In conclusion, our systematic review and meta-analysis demonstrates that scoliosis in children and adolescents remains highly prevalent globally, with evidence of an increasing trend since 2000 year. The global estimated prevalence is 1.65%, with girls affected at twice the rate of boys. Mild scoliosis (Cobb angle 10°−19°) and the single thoracolumbar curve subtype are the most common forms. There is significant difference in prevalence of scoliosis in children and adolescents among different WHO regional subdivisions and countries. Risk factors of scoliosis in children and adolescents may include sex (girls), family history, sedentary behavior (≥ 11 h), poor sitting posture, short outdoor time (< 1 h), long screen time (≥ 2 h), unilateral physical activity, and low BMI.

## Supplementary Information

Supplementary Material 1.

Supplementary Material 2.

## Data Availability

Data extracted from eligible studies will be available from the corresponding authors (Lingjun Kong, chunyong01@163.com and Min Fang, email: fm-tn0510@shutcm.edu.cn) following publication. Data access will be granted upon proposal approval and completion of appropriate data access agreements.

## Abbreviations

CI: Confidence interval
JBI: Joanna Briggs Institute
NOS: Newcastle-Ottawa Scale
ORs: Odds ratios
WHO: World Health Organization
BMI: Body Mass Index
