# Correction: Global prevalence and associated risk factors of scoliosis in children and adolescents: a systematic review and meta-analysis

**DOI:** 10.1186/s12889-025-25763-w

**Published:** 2025-12-23

**Authors:** Shoujian Wang, Miaoxiu Li, Jun Ren, Jiming Tao, Min Fang, Lingjun Kong

**Affiliations:** 1 Department of Tuina, Shuguang Hospital, Shanghai University of Traditional Chinese Medicine, Shanghai, China; 2 Institute of Tuina, Shanghai Institute of Traditional Chinese Medicine, Shanghai, China

**Correction** **:** **BMC Public Health 25, 3640 (2025)**

**https://doi.org/10.1186/s12889-025–24905-4**

Following publication of the original article[1], the authors reported an error found in Figure 5. The image displayed for Figure 5 is a duplicate of Figure 4. The correct figure is provided below.


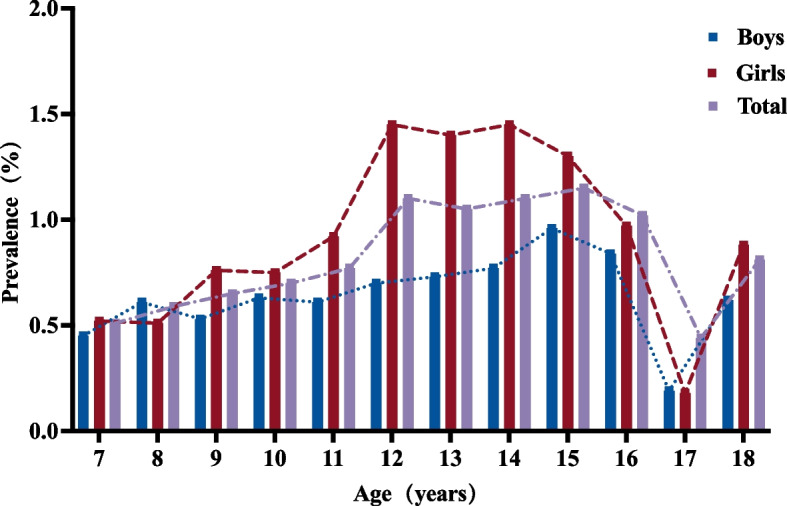


The original article [1] has been updated.
