# Supplementary material for: Global prevalence and associated risk factors of scoliosis in children and adolescents: a systematic review and meta-analysis
Source: BMC Public Health. 2025 Oct 28;25:3640. doi: 10.1186/s12889-025-24905-4 (PMC12570432; doi:10.1186/s12889-025-24905-4)
Supplement: Supplementary file 1 — Supplementary Material 1. [file 12889_2025_24905_MOESM1_ESM.pdf]

Supplementary material

Contents

Appendix S1: Search strategy to identify studies reporting the prevalence of scoliosis in children and adolescents. 3
Appendix S2: Basic information of the included studies in the research. 7
Appendix S3: ROB assessment. 20
S3.1 ROB assessment for the quality of studies reporting prevalence data in JBI Seale. 20
S3.2 ROB assessment for the quality of included analytical cross sectional studies in JBI Seale. 24
S3.3 ROB assessment for the quality of included case control studies in NOS Seale. 26
S3.4 ROB assessment for the quality of included cohort studies in NOS Seale. 26
Appendix S4: Reported prevalence of suspected scoliosis in children and adolescents in 39 countries with available data. . 27
S4.1 Prevalence of suspected scoliosis in children and adolescents in 39 countries. 27
S4.2 Global prevalence distribution of suspected scoliosis in children and adolescents in 39 countries. 28
S4.3 Forest plot of suspected scoliosis in children and adolescents in 39 countries. 29
Appendix S5: Reported prevalence of scoliosis in children and adolescents in 33 countries with available data. 30
S5.1 Prevalence of scoliosis in children and adolescents in 33 countries. 30
S5.2 Forest plot of scoliosis in children and adolescents in 33 countries. 31
Appendix S6: Reported prevalence of scoliosis in children and adolescents in 23 China province with available data. 32
S6.1 Prevalence of scoliosis in children and adolescents in 23 China province. 32
S6.2 Forest plot of scoliosis in children and adolescents in 23 China province. 33
Appendix S7: Reported prevalence of scoliosis in children and adolescents in different investigation periods with available data. 34
S7.1 Prevalence of scoliosis in children and adolescents in different investigation periods. 34
S7.2 Forest plot of scoliosis in children and adolescents in different investigation periods. 35
Appendix S8: Reported prevalence of scoliosis in children and adolescents in different WHO region with available data. .. 36
S8.1 Prevalence of scoliosis in children and adolescents in different WHO region. 36
S8.2 Forest plot of scoliosis in children and adolescents in different WHO region. 37
Appendix S9: Reported prevalence of scoliosis in children and adolescents in different World Bank region with available data. 38
S9.1 Prevalence of scoliosis in children and adolescents in different World Bank region. 38
S9.2 Forest plot of scoliosis in children and adolescents in different World Bank region. 39
Appendix S10: Reported prevalence of scoliosis in children and adolescents in boys and girls aged 6-18 years with available data. 40
S10.1 Prevalence of scoliosis in children and adolescents in boys and girls aged 6-18 years. 40
S10.2 Forest plot of scoliosis in children and adolescents in boys and girls aged 6-18 years. 42
Appendix S11: The proportion of different aetiological types with available data. 43
Appendix S12: The proportion of different Cobb angles with available data. 44
Appendix S13: The proportion of different scoliosis curve types with available data. 45
Appendix S14: Forest plot of subgroup. 47
Appendix S15: Included studies in meta-analyses of associated factors of scoliosis in children and adolescents. 55
Appendix S16: Sensitivity analyses. 63
S16.1 Influence Diagnostics. 63
S16.2 Leave-One-Out Analysis (Sorted by I^2). 67
Appendix S17: Publication bias. 69
S17.1 Funnel plots. 69
S17.2 Egger's and Begg's test. 69
S17.3 The result of the trim and fill method. 70

Appendix S18: List of studies excluded at full-text screening stage. 82

Appendix S1: Search strategy to identify studies reporting the prevalence of scoliosis in children and adolescents.

Database Search terms
MEDLINE
1. exp Scoliosis/
2. Spinal Curvature.mp.
3. Vertebral lateral curvature.mp.
4. Spinal deviation.mp.
5. Curvature deformity.mp.
6. or/1-5
7. exp adolescent/
8. (child or infant or childhood disease*).ti,ab,kf. or (adolescen* or babies or baby or boy? or boyhood or girlhood or child* or girl? or infan* or juvenil* or kid? or minors or minors* or neonat* or neo-nat* or newborn* or new-born* or paediatric* or peadiatric* or pediatric* or perinat* or preschool* or puber* or pubescen* or school* or teen* or toddler? or underage? or under-age? or youth*).ti,ab,kf. or (pediatric* or paediatric* or infan* or child*or adolescen* or young).jn,jw or (pediatric* or paediatric* or infan* or child*or adolescen* or young).in
9. 7 or 8
10. 6 and 9
11. exp Incidence/
12. Prevalence.mp.
13. morbidity.mp.
14. occurrence*.mp.
15. epidemiolog*.mp.
16. exp Risk Factors/
17. predictor.mp.
18. or/11-17
19. limit 18 to abstracts
20. 10 and 19
21. exp animals/ not humans.sh.
22. 20 not 21
EMBASE
1. exp Scoliosis/
2. Spinal Curvature.mp.

3. Vertebral lateral curvature.mp.

4. Spinal deviation.mp.

5. Curvature deformity.mp.

6. or/1-5

7. exp adolescent/

8. (child or infant or childhood disease*).ti,ab,kf. or (adolescen* or babies or baby or boy? or boyhood or girlhood or child* or girl? or infan* or juvenil* or kid? or minors or minors* or neonat* or neo-nat* or newborn* or new-born* or paediatric* or peadiatric* or pediatric* or perinat* or preschool* or puber* or pubescen* or school* or teen* or toddler? or underage? or under-age? or youth*).ti,ab,kf. or (pediatric* or paediatric* or infan* or child*or adolescen* or young).jn,jw or (pediatric* or paediatric* or infan* or child*or adolescen* or young).in

9. 7 or 8

10. 6 and 9

11. exp incidence/

12. Prevalence.mp.

13. morbidity.mp.

14. occurrence*.mp.

15. epidemiolog*.mp.

16. exp Risk Factors/

17. predictor.mp.

18. or/11-17

19. limit 18 to abstracts

20. 10 and 19

21. animal.mp. or *animal/

22. 20 not 21

Web of Science

19. #17 not #18

18. TS=(animal*)

17. #8 and #16

16. #9 OR #10 OR #11 OR #12 OR #13 OR #14 OR #15

15. TS=(predictor*)

14. TS=(Risk Factor*)

13. TS=(epidemiolog*)

12. TS=(occurrence*)

11. TS=(morbidity)

10. TS=(Prevalence)

9. TS=(incidence)

8. #6 and #7

7. TS=(adolescent or child or infant or infant disease* or childhood disease* or adolescen* or babies or baby or boy? or boyhood or girlhood or child* or girl? or infan* or juvenil* or kid? or minors or minors* or neonat* or neo-nat* or newborn* or new-born* or paediatric* or peadiatric* or pediatric* or perinat* or preschool* or puber* or pubescen* or school* or teen* or toddler? or underage? or under-age? or youth* or pediatric* or paediatric* or infan* or child*or adolescen* or young or pediatric* or paediatric* or infan* or child*or adolescen* or young)

6. #1 or #2 or #3 or #4 or #5

5. TS=(Curvature deformity)

4. TS=(Spinal deviation)

3. TS=(Vertebral lateral curvature)

2. TS=(Spinal Curvature)

1. TS=(scoliosis)

Global Index Medicus

(((incidence) OR (prevalence) OR (morbidity) OR (occurrence*) OR (epidemiolog*) OR (risk Factor*) OR (predictor*)) AND (((scoliosis) OR (spinal curvature) OR (vertebral lateral curvature) OR (spinal deviation) OR (curvature deformity)) AND (adolescent OR child OR infant OR infant disease* OR childhood disease* OR adolescen* OR babies OR baby OR boy? OR boyhood OR girlhood OR child* OR girl? OR infan* OR juvenil* OR kid? OR minors OR minors* OR neonat* OR neo-nat* OR newborn* OR new-born* OR paediatric* OR peadiatric* OR pediatric* OR perinat* OR preschool* OR puber* OR pubescen* OR school* OR teen* OR toddler? OR underage? OR under-age? OR youth* OR pediatric* OR paediatric* OR infan* OR child*or adolescen* OR young OR pediatric* OR paediatric* OR infan* OR child*or adolescen* OR young))) NOT (animal*)

China Knowledge Resource Integrated Database

1. (主题=脊柱侧弯) or (篇关摘=脊柱弯曲异常) or (篇关摘=脊柱侧凸) or (篇关摘=脊柱畸形)

2. (主题=青少年) or (篇关摘=儿童) or (篇关摘=中小学生)

3. (主题=发病率) or (篇关摘=患病率) or (篇关摘=流行病学)

4. 主题=危险因素

5. 1 and 2 and 3 and 4

6. 篇关摘=动物

7. 5 not 6

Wanfang Data Information

（((主题：脊柱侧弯）OR（题名或关键词：脊柱弯曲异常 or 脊柱侧凸 or 脊柱畸形））AND（(主题：青少年）OR（题名或关键词：儿童 or 中小学生））AND（(主题：发病率 or 危险因素）OR（题名或关键词：患病率 or 流行病学）））NOT（题名或关键词：动物）

Weipu Database for Chinese Technical Periodicals

(((((题名或关键词=脊柱侧弯 OR 题名或关键词=脊柱弯曲异常) OR 题名或关键词=脊柱侧凸) OR 题名或关键词=脊柱畸形) AND ((题名或关键词=青少年 OR 题名或关键词=儿童) OR 题名或关键词=中小学生)) AND (((题名或关键词=发病率 OR 题名或关键词=危险因素) OR 题名或关键词=患病率) OR 题名或关键词=流行病学))

Chinese biomedical literature service system, SinoMed

(("脊柱侧弯"[摘要:智能] or "脊柱侧凸"[摘要:智能] or "脊柱畸形"[摘要:智能] or "脊柱弯曲异常"[摘要:智能]) and (儿童[摘要:智能] or 青少年[摘要:智能] or 中小学生[摘要:智能]) and ("发病率"[摘要:智能] or "患病率"[摘要:智能] or "流行病学"[摘要:智能] or "危险因素"[摘要:智能]))

Appendix S2: **Basic information of the included studies in the research.**

Study ID	Author (s)	Country	WHO region	WB region	Latitude	Year of publication	Year of investigation	Study type	Sample source	Sample size	Age range (years)	Female (%)	Number of trunk asymmetry cases	Number of Idiopathic scoliosis cases	Number of scoliosis cases
Chu 2023[1]	Chu, et al.	China	WPR	UMIC	MID	2023	2021.9-12	1	school	4180	NR	47.72	334	NR	101
Zeng 2019[2]	Zeng, et al.	China	WPR	UMIC	MID	2019	2017.9-2019.3	1	school	104088	7-18	43.76	3426	480	534
Deng 2019[3]	Deng, et al.	China	WPR	UMIC	MID	2019	2018.2-10	1	school	5126	12-17	46.45	141	107	123
Ding 2020[4]	Ding, et al.	China	WPR	UMIC	MID	2020	2019.9-11	1	school	18518	12-16	49.69	303	207	234
Gao 2004[5]	Gao, et al.	China	WPR	UMIC	MID	2004	2002.6-2003.12	1	school	8652	7-15	49.20	93	63	65
He 2016[6]	He, et al.	China	WPR	UMIC	MID	2016	2015.5	1	school	21415	10-18	46.34	NR	154	154
Hu 2017[7]	Hu, et al.	China	WPR	UMIC	MID	2017	2015.9-2017.6	1	school	19870	12-18	40.31	268	143	151
Huang 2011a[8]	Huang, et al.	China	WPR	UMIC	MID	2011	2007.11-2009.7	1	school	28892	7-20	NR	NR	NR	199
Jia 2022[9]	Jia, et al.	China	WPR	UMIC	MID	2022	2019	1	school	7304	NR	48.30	659	NR	132
Ke 2012[10]	Ke, et al.	China	WPR	UMIC	MID	2012	2009.6-2011.9	1	school	18798	7-15	48.70	213	150	156
Li 2011[11]	Li, et al.	China	WPR	UMIC	MID	2011	1997.10-2009.10	1	school	44058	NR	NR	NR	NR	134
Li 2010[12]	Li, et al.	China	WPR	UMIC	MID	2010	2005.2-12	1	school	11310	7-15	46.79	162	134	137

Li 2001[13]	Li, et al.	China	WPR	UMIC	MID	2001	1996.10-1997.11	1	school	33798	7-15	47.80	453	350	361
Liu 2024[14]	Liu, et al.	China	WPR	UMIC	MID	2024	2022.3-7	1	school	2274	10-16	52.20	NR	NR	61
Lu 2010[15]	Lu, et al.	China	WPR	UMIC	MID	2010	2007.4-2009.5	1	school	17525	7-15	48.55	413	311	335
Ma 2015[16]	Ma, et al.	China	WPR	UMIC	MID	2015	2012.5-2013.4	1	school	6952	10-16	46.06	NR	88	NR
Ma 1995[17]	Ma, et al.	China	WPR	UMIC	MID	1995	1992.12-1993.7	1	school	24130	7-18	48.00	665	313	347
Meng 2003[18]	Meng, et al.	China	WPR	UMIC	MID	2003	1997-1999	1	school	NR	7-17	48.97	315	225	251
Miu 2017[19]	Miu, et al.	China	WPR	UMIC	MID	2017	2015.9-12	1	school	67322	10-17	45.21	2202	195	200
Ren 2014[20]	Ren, et al.	China	WPR	UMIC	MID	2014	2012.9-2013.12	1	school	17348	7-17	43.80	NR	89	NR
Shen 2019[21]	Shen, et al.	China	WPR	UMIC	MID	2019	2017.10-2018.5	1	school	15038	13-16	47.10	435	187	187
Wang 2013[22]	Wang, et al.	China	WPR	UMIC	MID	2013	2010.9-2011.12	1	school	18154	7-18	48.68	191	NR	112
Wen 2021[23]	Wen, et al.	China	WPR	UMIC	MID	2021	2020.10-12	1	school	36728	6-18	48.48	827	NR	44
Wu 2021[24]	Wu, et al.	China	WPR	UMIC	MID	2021	2019.1	1	school	1574	7-17	49.43	NR	110	NR
Yu 1995[25]	Yu, et al.	China	WPR	UMIC	MID	1995	1990-1992	1	school	8263	6-16	46.76	285	NR	158
Yu 2023[26]	Yu, et al.	China	WPR	UMIC	MID	2023	2021	1	school	1674	6-18	48.33	NR	NR	113
Yu 2014[27]	Yu, et al.	China	WPR	UMIC	MID	2014	2012.3-2014.6	1	school	29532	7-18	46.88	420	NR	250
Zhang 2008[28]	Zhang, et al.	China	WPR	UMIC	MID	2008	2002.9-2005.3	1	school	21112	7-18	46.31	305	153	158
Zhang 2011[29]	Zhang, et al.	China	WPR	UMIC	MID	2011	NR	1	school	1260	7-13	50.00	15	NR	10
Zhang 2023a[30]	Zhang, et al.	China	WPR	UMIC	MID	2023	2020.1-2022.12	1	school	27926	7-15	47.06	NR	NR	1067
Zhang 2017[31]	Zhang, et al.	China	WPR	UMIC	MID	2017	2015.3-2016.3	1	school	41258	12-18	46.04	NR	1238	1243

Zheng 2016[32]	Zheng, et al.	China	WPR	UMIC	MID	2016	2014.4-6	1	school	11024	6-13	46.41	282	11	NR
Zhou 2023[33]	Zhou, et al.	China	WPR	UMIC	MID	2023	2019.2-2023.2	1	community	12255	7-18	45.45	241	NR	177
Zhu 2021[34]	Zhu, et al.	China	WPR	UMIC	MID	2021	2020.9-12	1	school	146362	6-19	NR	1308	760	NR
Zhu 2017[35]	Zhu, et al.	China	WPR	UMIC	MID	2017	2014	1	school	4173	15-16	49.65	NR	NR	327
Zou 2022[36]	Zou, et al.	China	WPR	UMIC	MID	2022	2019	1	school	2568	NR	47.35	NR	NR	93
Bondar 2021[37]	Bondar, et al.	America	AMR	HIC	MID	2021	2013	1	database	937254	NR	48.90	NR	1893	NR
Cai 2021[38]	Cai, et al.	China	WPR	UMIC	MID	2021	2018	1，3	school	5497	6-12	45.10	338	338	NR
Carcamo 2023[39]	Carcamo, et al.	Chile	WPR	UMIC	MID	2023	2015-2016	1	school	1200	10-18	54.92	98	46	NR
Chan 1986[40]	Chan, et al.	Australia	WPR	HIC	LOW	1986	1982-1983	1	school	3660	NR	48.30	144	NR	71
Souza 2013[41]	Souza, et al.	Brazil	AMR	HIC	LOW	2013	2012	1	school	418	10-14	52.39	31	18	NR
Dohnert 2008[42]	Dohnert, et al.	Brazil	AMR	HIC	LOW	2008	2005.3-07	1	school	314	9-16	45.54	NR	10	28
Guo 2017[43]	Guo, et al.	China	WPR	UMIC	MID	2017	2015	1	school	5327	11-13	48.41	520	NR	102
Yang 2022b[44]	Yang, et al.	China	WPR	UMIC	MID	2022	NR	3	school	2538	NR	61.10	NR	1269	NR
Etemadifar 2020[45]	Etemadifar, et al.	Iran	EMR	UMIC	MID	2020	2014-2015	1	school	3018	10-14	50.13	335	19	NR
Jenyo 2005[46]	Jenyo, et al.	Nigeria	AFR	LMIC	LOW	2005	NR	1	school	410	9-14	53.66	30	NR	1
Hu 2022[47]	Hu, et al.	China	WPR	UMIC	MID	2022	2019	1	school	10731	11-15	48.58	462	214	NR
Karachalios 1999[48]	Karachalios, et al.	Greece	EUR	UMIC	MID	1999	1987	1	school	2700	8-16	NR	421	NR	32
An 2015[49]	An, et al.	Korea	WPR	HIC	MID	2015	2002-2011	1	school	413351	10-11	43.53	6937	1584	NR
Kim 2020[50]	Kim, et al.	Korea	WPR	HIC	MID	2020	2016	1	database	16613	NR	48.64	NR	434	NR
Komang-Agung 2017[51]	Komang-Agung, et al.	Indonesia	SEAR	UMIC	LOW	2017	2010	1	school	784	9-16	59.80	50	23	NR

Kunakornsawat 2017[52]	Kunakornswat, et al.	Thailand	SEAR	UMIC	LOW	2017	NR	1	school	1818	11-13	100.00	162	81	84
McMaster 2015[53]	McMaster, et al.	England	EUR	HIC	MID	2015	NR	3	school	898	NR	100.00	NR	596	NR
Park 2006[54]	Park, et al.	Korea	WPR	HIC	MID	2006	2004.3-10	1	school	18521	11	44.91	195	57	NR
Nussinovitch 2002[55]	Nussinovith, et al.	Israel	EMR	HIC	LOW	2002	NR	1	school	2380	12-18	52.00	NR	NR	39
Ostojic 2006[56]	Ostojic, et al.	Bosnia and Herzegovina	EUR	LMIC	MID	2006	2002-2003	1	school	2517	7-14	49.46	298	NR	79
Pruijs 1996[57]	Pruijs, et al.	Netherlands	EUR	HIC	MID	1996	1983	1	school	30563	7-14	49.46	3065	NR	245
Ravi 2019[58]	Ravi, et al.	India	SEAR	LMIC	LOW	2019	2016-2017	1	school	3250	11-15	48.00	164	58	NR
Sato 2011[59]	Sato, et al.	Japan	WPR	HIC	MID	2011	2005	1	school	32134	10-15	50.40	NR	51	NR
Yamamoto 2015a[60]	Yamamoto, et al.	Japan	WPR	HIC	MID	2015	1990-1999	1	school	79169	11-14	48.89	1820	205	NR
Yamamoto 2015b[60]	Yamamoto, et al.	Japan	WPR	HIC	MID	2015	2003-2012	1	school	59612	11-14	48.89		214	NR
Smyrnis 2015[61]	Smyrnis, et al.	Italy	EUR	HIC	MID	2015	1997-2011	1	school	1034	4-18	47.58	60	3	4
Stirling 1996[62]	Stirling, et al.	England	EUR	HIC	MID	1996	NR	1	school	15799	6-14	48.19	934	76	NR
Ugras 2010[63]	Ugras, et al.	Turkey	EUR	UMIC	MID	2010	2008	1	school	4259	10-14	48.30	39	NR	11
Yan 2020[64]	Yan, et al.	China	WPR	UMIC	MID	2020	2019-2020	3	school	7858	12-14	50.23	NR	3871	NR
Zhou 2022[65]	Zhou, et al.	China	WPR	UMIC	MID	2022	2020	1	school	9856	6-17	48.50	546	295	364
Yilmaz 2020[66]	Yilmaz, et al.	Turkey	EUR	UMIC	MID	2020	2017.1-2017.6	1	school	16045	10-15	50.90	2545	369	380
Yong 2009[67]	Yong, et al.	Singapore	WPR	HIC	LOW	2009	2003	1	school	93626	9-13	100.00	4906	1119	NR
Zhang 2021[68]	Zhang, et al.	China	WPR	UMIC	MID	2021	NR	3	school	60	NR	53.33	NR	30	NR
Zhu 2023[69]	Zhu, et al.	China	WPR	UMIC	MID	2023	NR	3	school	1488	9-17	71.77	NR	494	NR

Aulisa 2019[70]	Aulisa, et al.	Italy	EUR	HIC	MID	2019	NR	1	school	NR	9-14	53.29	181	NR	65
Arti 2005[71]	Arti, et al.	Iran	EMR	UMIC	MID	2005	2003	1	school	NR	10-14	NR	86	NR	7
Nissinen 1993[72]	Nissinen, et al.	Finland	EUR	HIC	HIGN	1993	1986	2	school	NR	NR	47.99	174	65	NR
Owada 1982[73]	Owada, et al.	Japan	WPR	HIC	MID	1982	1980	1	school	NR	6-15	49.70	NR	NR	36
Zheng 2017[74]	Zheng, et al.	China	WPR	UMIC	MID	2017	2015.4-12	1	school	79122	10-16	45.33	NR	1202	NR
Zhou 1984[75]	Zhou, et al.	China	WPR	UMIC	MID	1984	1983	1	school	4468	12-15	51.21	46	NR	10
Zhou 2008[76]	Zhou, et al.	China	WPR	UMIC	MID	2008	2007-2008	1	school	32004	7-18	46.84	NR	NR	233
Cheng 2006[77]	Chen, et al.	China	WPR	UMIC	MID	2006	2002-2004	1	school	25725	7-15	46.06	607	321	343
Pu 2022[78]	Pu, et al.	China	WPR	UMIC	MID	2022	2021	1	school	140026	6-18	50.06	3190	NR	NR
Huang 2023[79]	Huang, et al.	China	WPR	UMIC	MID	2023	2019-2020	1	school	6757	10-16	46.09	135	NR	NR
Li 1999a[80]	Li, et al.	China	WPR	UMIC	MID	1999	1985	1	school	987	10-14	50.15	100	NR	NR
Li 2022a[81]	Li, et al.	China	WPR	UMIC	MID	2022	2020	1	school	38649	9-18	47.64	1400	NR	NR
Zou 2021[82]	Zou, et al.	China	WPR	UMIC	MID	2021	2019	1	school	2942	NR	46.23	230	NR	NR
Zhang 2023b[83]	Zhang, et al.	China	WPR	UMIC	MID	2023	2021	1	school	17629	11-17	NR	695	NR	NR
Birgani 2006[84]	Birgani, et al.	Iran	EMR	UMIC	MID	2006	2005-2006	1	school	216	14-18	0.00	19	NR	NR
Deepak 2017[85]	Deepak, et al.	Malaysia	SEAR	HIC	LOW	2017	1996-1999	1	school	8966	13-15	51.14	410	NR	87
Dickson 1980[86]	Dickson, et al.	England	EUR	HIC	MID	1980	NR	2	school	1764	13-14	NR	147	NR	44
Freire 2008[87]	Freire, et al.	Brazil	AMR	HIC	LOW	2008	2008	1	school	609	10-15	47.13	46	NR	NR
Glavas 2023[88]	Glavas, et al.	Croatia	EUR	HIC	MID	2023	2019-2020	1	school	18216	NR	49.87	1053	90	NR
Lee 2014[89]	Lee, et al.	Korea	WPR	HIC	MID	2014	2004-2006	1	school	37856	11	45.20	204	NR	71
LevRan 2013[90]	LevRan, et al.	Israel	EMR	HIC	MID	2013	1996-1998	1	school	24846	NR	49.69	69	NR	NR
Natasa	Natasa, et al.	Serbia	EUR	UMIC	MID	2006	1995-1996	1	school	14634	7-14	50.00	1324	NR	357

2006[91]															
Robitaille 1984[92]	Robitaille, et al.	Canada	AMR	HIC	MID	1984	1975-1977	2	school	6873	NR	NR	982	143	NR
Sacco 2019[93]	Sacco, et al.	Malta	EUR	HIC	MID	2019	2012	1	school	245	13-15	46.53	13	NR	NR
Vazquez-Lazarte 2020[94]	Vazquez-Lazarte, et al.	Peru	AMR	UMIC	LOW	2020	2018	1	school	191	12-18	37.70	52	NR	NR
Se-ll 1980[95]	Se-ll, et al.	Korea	WPR	HIC	MID	1980	1977-1979	1	school	5256	12-18	69.18	246	NR	120
Wilczynski 2006[96]	Wilczynski, et al.	Poland	EUR	HIC	MID	2006	2004	1	school	191	13-16	0.00	131	NR	NR
Tzivian 1978[97]	Tzivian, et al.	Russia	EUR	HIC	MID	1978	NR	1	school	4038	7-15	49.26	687	NR	187
AndradeBarcia 1996[98]	AndradeBarcia, et al.	Ecuador	AMR	UMIC	LOW	1996	NR	1	school	7943	NR	NR	83	NR	4
Tisovsky 2004[99]	Tisovsky, et al.	Slovakia	AMR	UMIC	LOW	2004	NR	1	school	680	8-14	48.53	60	NR	10
Li 2020[100]	Li, et al.	China	WPR	UMIC	MID	2020	2019.3-05	1	school	864	11-15	NR	35	NR	NR
Li 2023a[101]	Li, et al.	China	WPR	UMIC	MID	2023	2021.4-06	1	school	5926	10-14	49.92	515	NR	NR
Li 2021[102]	Li, et al.	China	WPR	UMIC	MID	2021	2020.7	1	school	1465	3-16	93.17	81	NR	NR
Liu 2003[103]	Liu, et al.	China	WPR	UMIC	MID	2023	1985.5-1986.1	2	school	20418	7-15	NR	NR	NR	NR
Liu 2001[104]	Liu, et al.	China	WPR	UMIC	MID	2001	1998.6	1	school	7397	7-18	49.72	NR	NR	135
Liu 2002[105]	Liu, et al.	China	WPR	UMIC	MID	2002	1994.10-1997.11	1	school	87546	7-18	46.37	857	NR	653
Liu 2011[106]	Liu, et al.	China	WPR	UMIC	MID	2011	2005.10-2009.10	1	school	24362	6-16	49.83	518	368	423
Meng 2018[107]	Meng, et al.	China	WPR	UMIC	MID	2018	2015-2016	1	school	949708	6-18	NR	689	NR	NR
Qiao 2022[108]	Qiao, et al.	China	WPR	UMIC	MID	2022	2020.9-2020.10	1	school	3053	7-18	52.77	271	NR	NR
Qiu 2022[109]	Qiu, et al.	China	WPR	UMIC	MID	2022	2020.1-2021.11	1	school	18562	7-18	50.79	201	NR	163
Ren 2021[110]	Ren, et al.	China	WPR	UMIC	MID	2021	NR	1	school	936	9-16	60.47	118	NR	NR
Sun 2009[111]	Sun, et al.	China	WPR	UMIC	MID	2009	2007.10-12	1	school	17555	9-16	46.08	1894	NR	184

Tang 2011[112]	Tang, et al.	China	WPR	UMIC	MID	2011	2006-2010	1	school	4417	NR	47.39	335	NR	NR
Heine 1981[113]	Heine, et al.	Germany	EUR	HIC	MID	1981	NR	1	school	1970	10-14	48.38	NR	NR	56
Bunnell 1993[114]	Bunnell, et al.	America	AMR	HIC	MID	1993	NR	1	school	1000	NR	NR	120	NR	NR
AlDaajani 2021[115]	AlDaajani, et al.	Saudi Arabia	EMR	HIC	MID	2019	2018-2019	1	school	225041	NR	NR	1086	NR	NR
Santo 2011[116]	Santo, et al.	Brazil	AMR	UMIC	MID	2011	2002	1	school	3105	NR	NR	382	NR	38
Ibisoglu 2012[117]	Ibisoglu, et al.	Turkey	EUR	UMIC	MID	2012	2008-2009	1	school	8207	12-14	48.50	40	NR	NR
Nery 2010[118]	Nery, et al.	Brazil	AMR	UMIC	MID	2010	2008.2-05	1	school	1340	10-14	49.00	19	NR	NR
Sanchez 2010[119]	Sanchez, et al.	Spain	EUR	HIC	MID	2010	2008.9-12	1	school	682	6-12	0.00	64	NR	NR
Safikhani 2006[120]	Safikhani, et al.	Iran	EMR	UMIC	MID	2006	2004	1	school	1400	11-15	100.00	28	NR	NR
Ciaccia 2017[121]	Ciaccia, et al.	Brazil	AMR	UMIC	MID	2017	2015	1	school	954	NR	53.00	232	NR	NR
Petrovic 2012[122]	Petrovic, et al.	Serbia	EUR	UMIC	MID	2012	NR	1	school	229	7-11	44.98	12	NR	NR
Wang 2022a[123]	Wang, et al.	China	WPR	UMIC	MID	2022	2019-2021	1	school	5974	6-18	49.28	24	NR	NR
Wang 2018a[124]	Wang, et al.	China	WPR	UMIC	MID	2018	2017.5-7	1	school	784	9-16	59.80	50	NR	NR
Wang 2018b[125]	Wang, et al.	China	WPR	UMIC	MID	2018	2014.9-2015.2	1	school	25097	13-18	48.47	233	NR	61
Wang 1996[126]	Wang, et al.	China	WPR	UMIC	MID	1996	1986	1	school	21759	8-14	NR	902	202	231
Wang 2022b[127]	Wang, et al.	China	WPR	UMIC	MID	2022	2021.9-11	1	school	140026	6-18	50.06	3190	NR	NR
Wang 2007[128]	Wang, et al.	China	WPR	UMIC	MID	2007	2005.3-2006.1	1	school	57393	5-20	49.03	NR	59	64
Wei 2023[129]	Wei, et al.	China	WPR	UMIC	MID	2023	2020.5-2022.6	1	school	17520	10-17	46.93	935	NR	114

Xia 2019[130]	Xia, et al.	China	WPR	UMIC	MID	2019	2016.2-2017.12	1	school	3913	7-15	46.92	256	NR	33
Yang 2022a[131]	Yang, et al.	China	WPR	UMIC	MID	2022	NR	1	school	22564	6-15	48.63	826	NR	71
Yu 2010[132]	Yu, et al.	China	WPR	UMIC	MID	2010	NR	1	school	116907	6-20	45.65	5299	NR	NR
Zhang 2023c[133]	Zhang, et al.	China	WPR	UMIC	MID	2023	2022..9-11	1	school	18980	6-18	49.70	1011	NR	NR
Zhang 2003[134]	Zhang, et al.	China	WPR	UMIC	MID	2003	2002.10-2003.5	1	school	8198	7-16	46.05	242	NR	17
Zhao 2022[135]	Zhao, et al.	China	WPR	UMIC	MID	2022	2020.1	1	school	1342	9-18	48.81	106	NR	NR
Zhao 1996[136]	Zhao, et al.	China	WPR	UMIC	MID	1996	1984.9-11	1	school	10073	6-15	48.08	563	NR	487
Zhao 2014[137]	Zhao, et al.	China	WPR	UMIC	MID	2014	2011.7-2012.1	1	school	8351	7-15	49.57	175	81	85
Assiri 2019[138]	Assiri, et al.	Saudi Arabia	EMR	HIC	LOW	2019	2016-2017	1	school	417	11-19	0.00	90	NR	NR
Willner 1984a[139]	Willner, et al.	Sweden	EUR	HIC	HIGH	1984	1980-1982	1	school	6464	10	49.16	943	NR	NR
Brooks 1975[140]	Brooks, et al.	America	AMR	HIC	MID	1975	1971.11-1974.4	1	school	3492	12-14	55.56	624	NR	474
Ortega 2008[141]	Ortega, et al.	Spain	EUR	HIC	MID	2008	2006.2-12	1	school	2956	8-12	49.90	472	NR	NR
Tanchev 1996[142]	Tanchev, et al.	Bulgaria	EUR	LMIC	MID	1996	1995.1.1996.3	1	school	4800	11-15	NR	57	NR	NR
Soucacos 1997[143]	Soucacos, et al.	Greece	EUR	UMIC	MID	1997	1993-1994	1	school	82901	9-14	49.41	5803	NR	1436
Smyrnis 1979[144]	Smyrnis, et al.	Greece	EUR	UMIC	MID	1979	1974	1	school	3494	11-12	NR	362	NR	NR
Kansu 2009[145]	Kansu, et al.	Turkey	EUR	UMIC	MID	2009	2006-2007	1	school	3175	10-15	48.44	15	NR	NR
Kuru Çolak 2015[146]	Kuru Çolak, et al.	Turkey	EUR	UMIC	MID	2015	2012	1	school	2207	11-15	56.50	149	NR	11
Grivas 2002[147]	Grivas, et al.	Greece	EUR	UMIC	MID	2002	1997-1999	1	school	3039	5.5-17.5	50.44	262	NR	90

Mittal 1987[148]	Mittal, et al.	India	SEAR	LMIC	LOW	1987	1985-1986	1	school	25376	5-18	49.03	NR	7	32
Moaleja 2018[149]	Moaleja, et al.	Iran	WPR	UMIC	MID	2018	2016.10-2017.2	1	school	144	7-12	50.00	15	NR	2
Scaturro 2021[150]	Scaturro, et al.	Italy	EUR	HIC	MID	2021	2018-2020	1	school	428	10-13	46.73	66	47	47
Guo 2023[151]	Guo, et al.	China	WPR	UMIC	MID	2023	2020.10-2021.2	1	school	3582	5-18	48.49	201	NR	NR
Hai 2021[152]	Hai, et al.	China	WPR	UMIC	MID	2021	2019.9-2019.10	1	school	3006	8-18	49.20	NR	NR	NR
He 2018[153]	He, et al.	China	WPR	UMIC	MID	2018	2016.1-2016.12	1	school	13121	12-16	50.06	228	NR	NR
Huang 2011b[154]	Huang, et al.	China	WPR	UMIC	MID	2011	2005.6-2010.10	1	school	24060	7-18	47.93	NR	NR	448
Ke 2015[155]	Ke, et al.	China	WPR	UMIC	MID	2015	2013	1	school	15667	12-18	49.29	NR	161	166
Li 2023b[156]	Li, et al.	China	WPR	UMIC	MID	2023	2020	1	school	3354	9-17	50.66	114	NR	NR
Li 2022b[157]	Li, et al.	China	WPR	UMIC	MID	2022	2020.9-2021.12	1	school	980	12-15	48.37	27	NR	NR
Jiang 1994[158]	Jiang, et al.	China	WPR	UMIC	MID	1994	1989.5-1989.11	1	school	37003	6-12	49.06	668	NR	NR
Li 2017[159]	Li, et al.	China	WPR	UMIC	MID	2017	2015.3-2017.6	1	school	15247	11-16	51.16	NR	126	NR
Li 1999b[160]	Li, et al.	China	WPR	UMIC	MID	1999	1997.6-1998.6	1	school	18329	7-15	44.81	187	107	112
Serbescu 2007[161]	Serbescu, et al.	Romania	EUR	UMIC	MID	2007	NR	1	community	252	NR	55.16	NR	NR	42
SantistebanRobles 2011[162]	SantistebanRobles, et al.	Spain	EUR	HIC	MID	2011	2008-2009	1	school	251	6	NR	2	NR	NR
Misawa 2015[163]	Misawa, et al.	Japan	WPR	HIC	MID	2015	2005-2012	1	school	33840	NR	NR	835	NR	429
TristanchoBaró 2019[164]	TristanchoBaró, et al.	Colombia	AMR	HIC	MID	2019	2016-2017	1	school	387	10-18	NR	160	NR	NR
Baroni	Baroni, et al.	Brazil	AMR	UMIC	LOW	2015	2011	1	school	212	7-17	58.49	123	NR	123

2015[165]															
Gashaw 2021[166]	Gashaw, et al.	Ethiopia	AFR	UMIC	LOW	2021	2019.3-2019.6	1	school	1905	5-16	49.55	62	NR	NR
Suh 2011[167]	Suh, et al.	Korea	WPR	HIC	MID	2011	2000-2008	1	school	1134890	10-14	48.49	77910	37339	NR
Wong 2005[168]	Wong, et al.	Singapore	WPR	HIC	LOW	2005	1982-1997	1	school	72699	6-14	51.09	759	279	NR
Group 2005[169]	Group, et al.	China	WPR	UMIC	MID	1980	1979	1	school	2538	7-17	50.51	354	NR	NR
Zeng 2006[170]	Zeng, et al.	China	WPR	UMIC	LOW	2006	2004-2005	1	school	21668	6-18	46.89	465	NR	NR
Chen 2016[171]	Chen, et al.	China	WPR	UMIC	MID	2016	2013.9-2014.1	1	school	27890	7-18	46.90	708	NR	360
Chen 2012[172]	Chen, et al.	China	WPR	UMIC	LOW	2012	2010.11-2011.11	1	school	19646	7-16	45.73	175	NR	136
Chen 2023[173]	Chen, et al.	China	WPR	UMIC	LOW	2023	NR	1	school	19113	3-6	46.52	NR	NR	72
Chen 2022[174]	Chen, et al.	China	WPR	UMIC	LOW	2022	2020.1-12	1	school	969235	9-18	45.08	48587	NR	NR
Chen 2021[175]	Chen, et al.	China	WPR	UMIC	MID	2021	2020.9-2020.12	1	school	1426	4-8	45.93	78	NR	NR
Deng 2016[176]	Deng, et al.	China	WPR	UMIC	MID	2016	NR	3	school	2054	7-17	45.91	101	NR	NR
Di 2020[177]	Di, et al.	China	WPR	UMIC	MID	2020	2019	1	school	1863	7-18	46.65	84	NR	NR
Dong 2009[178]	Dong, et al.	China	WPR	UMIC	MID	2009	2007.3-2009.1	1	school	10119	9-15	46.20	197	62	64
Du 2018[179]	Du, et al.	China	WPR	UMIC	LOW	2018	2013.10-2015.1	1	school	12881	11-17	48.56	263	136	NR
Duan 2023[180]	Duan, et al.	China	WPR	UMIC	MID	2023	2022.10.-2023.5	1	school	1601	7-13	44.66	81	NR	NR
Gao 2023[181]	Gao, et al.	China	WPR	UMIC	MID	2023	2021.4-2022.1	1	school	85146	7-15	47.70	17233	NR	2080
Kapoor 2008[182]	Kapoor, et al.	America	AMR	HIC	MID	2008	NR	1	school	1058	8-15	57.00	30	NR	NR
Morais 1985[183]	Morais, et al.	Canada	AMR	HIC	MID	1985	1977-1978	1	school	29195	8-15	50.31	3336	NR	513

Ramli 2018[184]	Ramli, et al.	Malaysia	SEAR	UMIC	LOW	2018	2011-2015	1	school	34638	11-12	NR	374	NR	132
Tobias 2019[185]	Tobias, et al.	England	EUR	HIC	MID	2019	1991-1992，2001-2002	2	database	14062	10-11	NR	NR	NR	232
Ueno 2011[186]	Ueno, et al.	Japan	WPR	UMIC	MID	2011	2003-2007	2	school	255875	11-14	49.57	3424	NR	2225
Willner 1982a[187]	Willner, et al.	Sweden	EUR	HIC	HIGH	1982	1971-1980	1	school	30031	7-15	49.78	108	NR	NR
Willner 1984b[188]	Willner, et al.	Sweden	EUR	HIC	MID	1984	1973-1980	3	school	2206	10-14	50.41	265	NR	NR
Willner 1982b[189]	Willner, et al.	Sweden	EUR	HIC	MID	1982	1971-1980	1	school	17181	7-16	49.29	NR	NR	327
Abo-Bakr 1992[190]	Abo-Bakr, et al.	Saudi Arabia	EMR	HIC	LOW	1992	NR	1	school	1147	11-13	100.00	40	29	NR
Din 2021[191]	Din, et al.	Malaysia	SEAR	UMIC	LOW	2021	NR	1	school	374	10-12	100.00	21	NR	NR
Baba 2020[192]	Baba, et al.	India	SEAR	LMIC	LOW	2020	2018	1	school	600	10-13	100.00	24	NR	NR
Chen 2010[193]	Chen, et al.	China	WPR	UMIC	MID	2010	2006-2009	1	school	12257	7-16	48.40	NR	38	41
Koukourakis 1997[194]	Koukouraki, et al.	Greece	EUR	HIC	MID	1997	1990-1992	1	school	24184	6-12	42.50	2049	NR	360
Rogala 1978[195]	Rogala, et al.	Canada	AMR	HIC	MID	1978	1974-1976	1	school	30021	12-14	NR	1744	NR	584
Minghelli 2014[196]	Minghelli, et al.	Portugal	EUR	HIC	MID	2014	NR	1	school	966	10-16	54.76	147	NR	NR
Zou 2022b[197]	Zou, et al.	China	WPR	UMIC	MID	2022	2019	1	school	45547	6-17	47.95	1766	NR	NR
Yuan 2024[198]	Yuan, et al.	China	WPR	UMIC	MID	2024	2018-2020	1	school	78853	7-18	51.32	4647	NR	NR
Sung 2021[199]	Sung, et al.	Korea	WPR	HIC	MID	2021	2011-2015	1	database	36984504	0-19	48.17	NR	148169	NR
Sugita 2000[200]	Sugita, et al.	Japan	WPR	HIC	MID	2000	1993-1995	1	school	3299	NR	51.69	NR	NR	106
Wei 2018[201]	Wei, et al.	China	WPR	UMIC	MID	2018	2016	1	school	3483	9-15	48.41	265	NR	NR
Dantas 2021[202]	Dantas, et al.	Brazil	AMR	UMIC	LOW	2021	2017-2019	1	school	520	10-16	NR	51	16	NR
Penha	Penha, et al.	Brazil	AMR	UMIC	LOW	2018	NR	1	school	2562	10-14	58.16	126	NR	37

2018[203]															
Du 2010[204]	Du, et al.	China	WPR	UMIC	MID	2021	2020	1	school	2121	NR	48.14	NR	NR	69
Sun 2021[205]	Sun, et al.	China	WPR	UMIC	MID	2024	2023	1	school	6395	NR	40.34	174	NR	NR
Jian 2024[206]	Jian, et al.	China	WPR	UMIC	MID	2021	2019	1	school	8176	7-18	48.81	792	NR	NR
Wen 2021b[207]	Wen, et al.	China	WPR	UMIC	MID	2020	NR	1	school	595057	NR	45.40	1021	NR	NR
Yang 2020[208]	Yang, et al.	China	WPR	UMIC	MID	2016	2015	1	school	13786	6-19	NR	21844	NR	NR
Huang 2016[209]	Huang, et al.	China	WPR	UMIC	MID	2017	2014	1	school	14326	3-10	49.53	217	NR	NR
Li 2017b[210]	Li, et al.	China	WPR	UMIC	MID	1985	1983	1	school	8165	6-15	48.54	236	NR	29
Pin 1985[211]	Pin, et al.	China	WPR	UMIC	MID	2016	2012-2013	1	school	6824	6-17	49.05	790	167	171
Du 2016[212]	Du, et al.	China	WPR	UMIC	MID	2017	2013-2016	1	school	3100	NR	NR	442	NR	172
Nie 2017[213]	Nie, et al.	China	WPR	UMIC	MID	1999	1997-1998	1	school	21859	7-12	47.90	NR	NR	59
Li 1999c[214]	Li, et al.	China	WPR	UMIC	MID	2005	2003-2004	1	school	8210	4-7	49.30	90	74	NR
Liang 2005[215]	Liang, et al.	China	WPR	UMIC	MID	2011	NR	1	school	40579	6-15	NR	116	67	72
Tang 2011b[216]	Tang, et al.	China	WPR	UMIC	MID	2021	2020	1	school	2121	NR	48.14	73	NR	50
Wang 1998[217]	Wang, et al.	China	WPR	UMIC	MID	1998	1994.10-1995.2	1	school	7964	10-14	56.94	NR	NR	39
Burwell 1983[218]	Burwell, et al.	England	EUR	HIC	MID	1983	NR	1	school	636	8-15	NR	51	NR	8
Dickson 1983[219]	Dickson, et al.	England	EUR	HIC	MID	1983	NR	1	community	5303	10-14	49.27	NR	NR	150
Rocha 2012[220]	Rocha, et al.	Brazil	AMR	UMIC	LOW	2012	NR	1	school	228	12	64.47	110	NR	NR
Pereira 2005[221]	Pereira, et al.	Brazil	AMR	UMIC	LOW	2005	NR	1	school	143	10-15	72.03	71	NR	NR
Penha 2005[222]	Penha, et al.	Brazil	AMR	UMIC	LOW	2005	NR	1	school	132	7-10	NR	59	NR	NR
Bertolini 1997[223]	Bertolini, et al.	Brazil	AMR	UMIC	LOW	1997	NR	1	school	200	11-14	59.50	21	NR	NR

Noll 2012[224]	Noll, et al.	Brazil	AMR	UMIC	LOW	2012	2011	1	school	65	11-16	NR	41	NR	NR
Liu 2021[225]	Liu, et al.	China	WPR	UMIC	MID	2021	2020	1	school	3248	9-19	50.74	327	NR	NR
Yawn 1999[226]	Yawn, et al.	America	AMR	HIC	MID	1999	NR	1	school	3248	9-19	50.74	327	NR	NR
Singh 2022[227]	Singh, et al.	India	SEAR	LMIC	LOW	2022	NR	1	school	2245	NR	NR	NR	NR	41
Goldberg 1995[228]	Goldberg, et al.	America	AMR	HIC	MID	1995	1986-1987	1	school	5518	12-16	NR	NR	24	NR
Adobor 2011[229]	Adobor, et al.	Norway	EUR	HIC	HIC	2011	NR	1	school	8686	NR	100.00	61	NR	33
Lonsete 1976[230]	Lonsete, et al.	America	AMR	HIC	MID	1976	1974-1975	1	school	4000	12	NR	60	NR	22
Donald 1981[231]	Donald, et al.	America	AMR	HIC	MID	1981	1973-1977	1	school	483578	NR	NR	40575	NR	5235
Liston 1981[232]	Liston, et al.	Australia	WPR	HIC	LOW	1981	1977-1979	1	community	8393	5-10	91.05	273	NR	168
Zaina 2013[233]	Zaina,et al.	Italy	EUR	HIC	MID	2013	NR	3	school	NR	NR	NR	NR	100%	NR
Kesak-Ursic 2021[234]	Kesak-Ursic, et al.	Croatia	EUR	HIC	MID	2021	NR	3	school	200	NR	NR	6-18	NR	NR
Krześniak 2022[235]	Krześniak,et al.	Poland	EUR	HIC	MID	2022	2018-2019	3	school	208	NR	NR	7-18	NR	NR
Laskowska 2019[236]	Laskowska,et al.	Poland	EUR	HIC	MID	2019	NR	3	school	80	NR	NR	8-18	88.75 %	NR
Lee 2005[237]	Lee,et al.	China	WPR	UMIC	MID	2005	NR	3	school	898	NR	NR	11-16	100%	NR
Tam 2016[238]	Tam,et al.	China	WPR	UMIC	MID	2016	NR	3	school	264	NR	NR	12-14	100%	NR
Pjanic 2024[239]	Pjanic,et al.	Serbia	EUR	UMIC	MID	2024	NR	3	school	141	NR	NR	7-18	70.92 %	NR

Note: Study type, 1 = cross-sectional study, 2 = cohort study, 3 = case-control study; NR, not reported; WHO, World Health Organization; AFR, African Region; AMR, Region of the Americas; SEAR, South-East Asia Region; EUR, European Region; EMR, Eastern Mediterranean Region; WPR, Western Pacific Region; WB, World Bank; HIC, high-income countries; LMIC, low- and middle-income countries; UMIC, upper middle-income countries.

Appendix S3: **ROB assessment.**

S3.1 ROB assessment for the quality of studies reporting prevalence data in JBI Seale.

Study ID	C1	C2	C3	C4	C5	C6	C7	C8	C9	Quality Score
Zeng 2019[2]	Yes	Yes	Yes	Yes	Unclear	Yes	Yes	Unclear	Unclear	6
Ding 2020[4]	Yes	Yes	Yes	Yes	Unclear	Yes	Yes	Unclear	Unclear	6
Gao 2004[5]	Yes	Yes	Yes	Yes	Unclear	Yes	Yes	Unclear	Unclear	6
He 2016[6]	Yes	Yes	Yes	Yes	Unclear	Yes	Yes	Unclear	Unclear	6
Hu 2017[7]	Yes	Yes	Yes	Yes	Yes	Yes	Yes	Unclear	Yes	8
Huang 2011a[8]	Yes	Yes	Yes	Yes	Unclear	Yes	Yes	Unclear	Unclear	6
Ke 2012[10]	Yes	Yes	Yes	Yes	Unclear	Yes	Unclear	Unclear	Unclear	5
Li 2011[11]	Yes	Yes	Yes	Yes	Unclear	Yes	Yes	Unclear	Unclear	6
Li 2010[12]	Yes	Yes	Yes	Yes	Unclear	Yes	Yes	Unclear	Unclear	6
Li 2001[13]	Yes	Yes	Yes	Yes	Unclear	Yes	Unclear	Unclear	Unclear	5
Lu 2010[15]	Yes	Yes	Yes	Yes	Unclear	Yes	Unclear	Unclear	Unclear	5
Ma 2015[16]	Yes	Yes	Yes	Yes	Unclear	Yes	Unclear	Unclear	Unclear	5
Ma 1995[17]	Yes	Yes	Yes	Yes	Unclear	Yes	Yes	Unclear	Unclear	6
Meng 2003[18]	Yes	Yes	Yes	Yes	Unclear	Yes	Yes	Unclear	Unclear	6
Miu 2017[19]	Yes	Yes	Yes	Yes	Unclear	Yes	Yes	Unclear	Unclear	6
Ren 2014[20]	Yes	Yes	Yes	Yes	Unclear	Yes	Yes	Unclear	Unclear	6
Wang 2013[22]	Yes	Yes	Yes	Yes	Unclear	Yes	Unclear	Unclear	Unclear	5
Wen 2021[23]	Yes	Yes	Yes	Yes	Unclear	Yes	Unclear	Unclear	Yes	6
Yu 1995[25]	Yes	Yes	Yes	Yes	Unclear	Yes	Unclear	Unclear	Unclear	5
Yu 2014[27]	No	Yes	Yes	Yes	Unclear	Yes	Unclear	Unclear	Unclear	4
Zhang 2008[28]	Yes	Yes	Yes	Yes	Unclear	Yes	Yes	Unclear	Unclear	6
Zhang 2011[29]	Yes	Yes	No	Yes	Unclear	Yes	Unclear	Unclear	Unclear	4
Zhang 2017[31]	Yes	Yes	Yes	Yes	Unclear	Yes	Yes	Unclear	Unclear	6
Zhu 2021[34]	Yes	Yes	Yes	Yes	Unclear	Yes	Unclear	Unclear	Unclear	5
Zhu 2017[35]	Yes	Yes	Yes	Yes	Unclear	Yes	Unclear	Unclear	Unclear	5
Carcamo 2023[39]	Yes	Yes	No	Yes	Unclear	Yes	Yes	Yes	Unclear	6
Chan 1986[40]	Yes	Yes	Yes	Yes	Yes	Yes	Yes	Yes	Yes	9
Souza 2013[41]	Yes	Yes	No	Yes	Unclear	Yes	Unclear	Unclear	Unclear	4
Dohnert 2008[42]	Yes	Yes	No	Yes	Unclear	Yes	Unclear	Unclear	Unclear	4
Guo 2017[43]	Yes	Yes	Yes	Yes	Unclear	Yes	Unclear	Unclear	Unclear	5
Jenyo 2005[46]	Yes	Yes	No	Yes	Unclear	Yes	Unclear	Unclear	Unclear	4
Karachalios 1999[48]	Yes	Yes	Yes	Yes	Unclear	Yes	Unclear	Unclear	Unclear	5
An 2015[49]	Yes	Yes	Yes	Yes	Unclear	Yes	Yes	Yes	Unclear	7
Komang-Agung 2017[51]	Yes	Yes	No	Yes	Unclear	Yes	Unclear	Unclear	Unclear	4
Kunakornsawat 2017[52]	Yes	Yes	No	Yes	Unclear	Yes	Unclear	Unclear	Unclear	4
Park 2006[54]	Yes	Yes	Yes	Yes	Unclear	Yes	Unclear	Unclear	Unclear	5
Nussinovitch 2002[55]	Yes	Yes	Yes	Yes	Unclear	Yes	Yes	Unclear	Unclear	6
Ostojic 2006[56]	Yes	Yes	Yes	Yes	Unclear	Yes	Unclear	Unclear	Unclear	5
Pruijs 1996[57]	Yes	Yes	Yes	Yes	Unclear	Yes	Unclear	Unclear	Unclear	5
Yamamoto 2015[60]	Yes	Yes	Yes	Yes	Yes	Yes	Unclear	Unclear	Unclear	6

Smyrnis 2015[61]	Yes	Yes	No	Yes	Unclear	Yes	Unclear	Unclear	Unclear	4
Stirling 1996[62]	Yes	Yes	Yes	Yes	Yes	Yes	Unclear	Unclear	Yes	7
Ugras 2010[63]	Yes	Yes	Yes	Yes	Unclear	Yes	Unclear	Unclear	Unclear	5
Yilmaz 2020[66]	Yes	Yes	Yes	Yes	Unclear	Yes	Yes	Yes	Unclear	7
Aulisa 2019[70]	Yes	Yes	Yes	Yes	Unclear	Yes	Unclear	Unclear	Unclear	5
Arti 2005[71]	Yes	Yes	No	Yes	Unclear	Yes	Unclear	Unclear	Unclear	4
Zhou 1984[75]	Yes	Unclear	Yes	Yes	Unclear	Yes	Unclear	Unclear	Unclear	4
Zhou 2008[76]	Yes	Unclear	Yes	Yes	Unclear	Yes	Unclear	Unclear	Unclear	4
Cheng 2006[77]	Yes	Yes	Yes	Yes	Unclear	Yes	Yes	Unclear	Unclear	6
Pu 2022[78]	Yes	Yes	Yes	Yes	Unclear	No	Yes	Yes	Unclear	6
Li 1999a[80]	Yes	Unclear	No	Yes	Unclear	No	Yes	Unclear	Unclear	3
Deepak 2017[85]	Yes	Unclear	Yes	Yes	Yes	Yes	Unclear	Unclear	Unclear	5
Glavas 2023[88]	Yes	Unclear	Yes	Yes	Unclear	Yes	Unclear	Yes	Unclear	5
Lee 2014[89]	Yes	Unclear	Yes	Yes	Unclear	Yes	Unclear	Unclear	Unclear	4
LevRan 2013[90]	Yes	Yes	Yes	Yes	Yes	No	Unclear	Yes	Yes	7
Natasa 2006[91]	Yes	Unclear	Yes	Yes	Unclear	Yes	Unclear	Unclear	Unclear	4
Sacco 2019[93]	Yes	Unclear	No	Yes	Unclear	No	Unclear	Yes	Unclear	3
Vazquez-Lazarte 2020[94]	Yes	Unclear	No	Yes	Unclear	No	Unclear	Yes	Unclear	3
Se-ll 1980[95]	Yes	Unclear	Yes	Yes	Unclear	Yes	Unclear	Unclear	Unclear	4
Li 2023a[101]	Yes	Yes	Yes	Yes	Unclear	No	Yes	Yes	Unclear	6
Li 2021[102]	Yes	Unclear	No	Yes	Unclear	No	Unclear	Yes	Unclear	3
Liu 2001[104]	Yes	Unclear	Yes	Yes	Unclear	No	Yes	Unclear	Unclear	4
Liu 2002[105]	Yes	Unclear	Yes	Yes	Unclear	Yes	Unclear	Unclear	Unclear	4
Liu 2011[106]	Yes	Unclear	Yes	Yes	Unclear	Yes	Unclear	Unclear	Unclear	4
Meng 2018[107]	Yes	Yes	Yes	Yes	Unclear	No	Unclear	Yes	Unclear	5
Qiao 2022[108]	Yes	Yes	Yes	Yes	Unclear	No	Unclear	Yes	Unclear	5
Qiu 2022[109]	Yes	Unclear	Yes	Yes	Unclear	Yes	Yes	Yes	Unclear	6
Ren 2021[110]	Yes	Yes	No	Yes	Unclear	No	Unclear	Unclear	Unclear	3
Sun 2009[111]	Yes	Unclear	Yes	Yes	Unclear	Yes	Unclear	Unclear	Unclear	4
Tang 2011[112]	Yes	Unclear	Yes	Yes	Unclear	No	Unclear	Unclear	Unclear	3
Heine 1981[113]	Yes	Unclear	Yes	Yes	Unclear	Yes	Unclear	Unclear	Unclear	4
Bunnell 1993[114]	Yes	Unclear	No	Yes	Unclear	No	Yes	Unclear	Unclear	3
AlDaajani 2021[115]	Yes	Yes	Yes	Yes	Unclear	No	Unclear	Yes	Unclear	5
Ibisoglu 2012[117]	Yes	Yes	Yes	Yes	Unclear	No	Unclear	Unclear	Unclear	4
Nery 2010[118]	Yes	Unclear	No	Yes	Unclear	No	Unclear	Yes	Unclear	3
Wang 2022a[123]	Yes	Unclear	Yes	Yes	Unclear	No	Yes	Yes	Unclear	5
Wang 2018b[125]	Yes	Yes	Yes	Yes	Unclear	Yes	Unclear	Unclear	Unclear	5
Wang 1996[126]	Yes	Yes	Yes	Yes	Yes	Yes	Unclear	Unclear	Yes	7
Wei 2023[129]	Yes	Unclear	Yes	Yes	Unclear	Yes	Unclear	Yes	Unclear	5
Xia 2019[130]	Yes	Unclear	Yes	Yes	Unclear	Yes	Unclear	Unclear	Yes	5
Zhang 2003[134]	Yes	Yes	Yes	Yes	Unclear	No	Unclear	Yes	Unclear	5
Zhao 2022[135]	Yes	Unclear	Yes	Yes	Unclear	No	Yes	Unclear	Unclear	4
Zhao 1996[136]	Yes	Unclear	Yes	Yes	Unclear	Yes	Unclear	Unclear	Unclear	4
Zhao 2014[137]	Yes	Unclear	Yes	Yes	Unclear	Yes	Yes	Unclear	Unclear	5
Brooks 1975[140]	Yes	Unclear	Yes	Yes	Unclear	Yes	Unclear	Yes	Unclear	5

Ortega 2008[141]	Yes	Unclear	Yes	Yes	Unclear	No	Yes	Yes	Unclear	5
Tanchev 1996[142]	Yes	Unclear	Yes	Yes	Unclear	No	Yes	Unclear	Unclear	4
Soucacos 1997[143]	Yes	Yes	Yes	Yes	Unclear	Yes	Unclear	Yes	Unclear	6
Smyrnis 1979[144]	Yes	Unclear	Yes	Yes	Unclear	No	Unclear	Unclear	Unclear	3
Kansu 2009[145]	Yes	Yes	Yes	Yes	Unclear	No	Unclear	Yes	Unclear	5
Kuru Çolak 2015[146]	Yes	Unclear	No	Yes	Unclear	Yes	Unclear	Unclear	Unclear	3
Grivas 2002[147]	Yes	Unclear	Yes	Yes	Unclear	Yes	Unclear	Unclear	Unclear	4
Mittal 1987[148]	Yes	Unclear	Yes	Yes	Unclear	Yes	Unclear	Unclear	Unclear	4
Moaleja 2018[149]	Yes	Unclear	No	Yes	Unclear	Yes	Unclear	Yes	Unclear	4
He 2018[153]	Yes	Yes	Yes	Yes	Unclear	No	Unclear	Yes	Unclear	5
Huang 2011b[154]	Yes	Yes	Yes	Yes	Unclear	Yes	Unclear	Unclear	Unclear	5
Ke 2015[155]	Yes	Yes	Yes	Yes	Unclear	Yes	Unclear	Unclear	Unclear	5
Li 2022b[157]	Yes	Unclear	No	Yes	Unclear	No	Unclear	Unclear	Unclear	2
Jiang 1994[158]	Yes	Yes	Yes	Yes	Unclear	No	Unclear	Unclear	Unclear	4
Li 2017[159]	Yes	Yes	Yes	Yes	Unclear	Yes	Unclear	Yes	Unclear	6
Li 1999b[160]	Yes	Yes	Yes	Yes	Unclear	Yes	Yes	Unclear	Unclear	6
Serbescu 2007[161]	Yes	Unclear	No	Yes	Unclear	Yes	Unclear	Unclear	Unclear	3
SantistebanRobles 2011[162]	Yes	Unclear	No	Yes	Unclear	Yes	Unclear	Yes	Unclear	4
Misawa 2015[163]	Yes	Unclear	Yes	Yes	Unclear	Yes	Unclear	Yes	Unclear	5
TristanchoBaró 2019[164]	Yes	Unclear	No	Yes	Unclear	No	Yes	Yes	Unclear	4
Suh 2011[167]	Yes	Yes	Yes	Yes	Unclear	Yes	Yes	Unclear	Unclear	6
Group 2005[169]	Yes	Unclear	Yes	Yes	Unclear	No	Unclear	Unclear	Unclear	3
Zeng 2006[170]	Yes	Yes	Yes	Yes	Unclear	No	Unclear	Unclear	Unclear	4
Chen 2016[171]	Yes	Yes	Yes	Yes	Unclear	Yes	Unclear	Yes	Unclear	6
Chen 2012[172]	Yes	Yes	Yes	Yes	Unclear	Yes	Yes	Unclear	Unclear	6
Chen 2023[173]	Yes	Yes	Yes	Yes	Unclear	Yes	Unclear	Yes	Unclear	6
Chen 2022[174]	Yes	Yes	Yes	Yes	Unclear	No	Yes	Yes	Unclear	6
Di 2020[177]	Yes	Yes	No	Yes	Unclear	No	Unclear	Yes	Unclear	4
Dong 2009[178]	Yes	Yes	Yes	Yes	Unclear	Yes	Unclear	Unclear	Unclear	5
Du 2018[179]	Yes	Yes	Yes	Yes	Unclear	Yes	Yes	Unclear	Unclear	6
Duan 2023[180]	Yes	Yes	Unclear	Yes	Unclear	No	Unclear	Unclear	Unclear	3
Gao 2023[181]	Yes	Yes	Yes	Yes	Unclear	Yes	Yes	Yes	Unclear	7
Kapoor 2008[182]	Yes	Unclear	No	Yes	Unclear	No	Yes	Yes	Unclear	4
Morais 1985[183]	Yes	Yes	Yes	Yes	Unclear	Yes	Unclear	Yes	Unclear	6
Ramli 2018[184]	Yes	Unclear	Yes	Yes	Unclear	Yes	Unclear	Yes	Unclear	5
Willner 1982a[187]	Yes	Yes	Yes	Yes	Unclear	No	Yes	Unclear	Unclear	5
Willner 1982b[189]	Yes	Unclear	Yes	Yes	Unclear	Yes	Yes	Unclear	Unclear	5
Abo-Bakr 1992[190]	Yes	Unclear	No	Yes	Unclear	Yes	Yes	Yes	Unclear	5
Baba 2020[192]	Yes	Yes	No	Yes	Unclear	No	Unclear	Yes	Unclear	4
Chen 2010[193]	Yes	Yes	Yes	Yes	Unclear	Yes	Yes	Unclear	Unclear	6
Koukourakis 1997[194]	Yes	Unclear	Yes	Yes	Unclear	Yes	Yes	Unclear	Unclear	5
Rogala 1978[195]	Yes	Yes	Yes	Yes	Unclear	Yes	Unclear	Unclear	Unclear	5
Yuan 2024[198]	Yes	Unclear	Yes	Yes	Unclear	No	Yes	Yes	Unclear	5
Du 2010[204]	Yes	Yes	Yes	Yes	Unclear	Yes	Unclear	Yes	Unclear	6
Yang 2020[208]	Yes	Unclear	Yes	Yes	Unclear	No	Unclear	Unclear	Unclear	3

Huang 2016[209]	Yes	Yes	Yes	Yes	Unclear	No	Yes	Yes	Unclear	6
Li 2017b[210]	Yes	Yes	Yes	Yes	Unclear	Yes	Yes	Yes	Unclear	7
Pin 1985[211]	Yes	Yes	Yes	Yes	Unclear	Yes	Yes	Unclear	Unclear	6
Du 2016[212]	Yes	Unclear	Yes	Yes	Unclear	Yes	Yes	Yes	Unclear	6
Nie 2017[213]	Yes	Yes	Yes	Yes	Unclear	Yes	Yes	Yes	Unclear	7
Li 1999c[214]	Yes	Yes	Yes	Yes	Unclear	Yes	Unclear	Unclear	Unclear	5
Liang 2005[215]	Yes	Yes	Yes	Yes	Unclear	Yes	Yes	Unclear	Unclear	6
Wang 1998[217]	Yes	Yes	Yes	Yes	Unclear	Yes	Unclear	Unclear	Unclear	5
Burwell 1983[218]	Yes	Yes	No	Yes	Unclear	Yes	Unclear	Unclear	Unclear	4
Dickson 1983[219]	Yes	Yes	Yes	Yes	Unclear	Yes	Yes	Yes	Unclear	7
Penha 2005[222]	Yes	Yes	No	Yes	Unclear	No	Unclear	Unclear	Unclear	3
Yawn 1999[226]	Yes	Yes	Yes	Yes	Unclear	Yes	Yes	Yes	Unclear	7
Goldberg 1995[228]	Yes	Unclear	Yes	Yes	Unclear	Yes	Unclear	Unclear	Unclear	4
Adobor 2011[229]	Yes	Unclear	Yes	Yes	Unclear	Yes	Yes	Yes	Unclear	6
Lonsete 1976[230]	Yes	Unclear	Yes	Yes	Unclear	Yes	Unclear	Yes	Unclear	5
Donald 1981[231]	Yes	Unclear	Yes	Yes	Unclear	Yes	Yes	Unclear	Unclear	5
Liston 1981[232]	Yes	Unclear	Yes	Yes	Unclear	No	Yes	Unclear	Unclear	4
Owada 1982[73]	Yes	Yes	Yes	Yes	Unclear	Yes	Unclear	Unclear	Unclear	5
Noll 2012[224]	Yes	Unclear	No	Yes	Unclear	No	Unclear	Yes	Unclear	3

Note: JBI, Joanna Briggs Institute; C1.Was the sample frame appropriate to address the target population? C2.Were study participants recruited in an appropriate way? C3. Was the sample size adequate? C4. Were the study subjects and setting described in detail? C5. Was data analysis conducted with sufficient coverage of the identified sample? C6. Were valid methods used for the identification of the condition? C7. Was the condition measured in a standard, reliable way for all participants? C8. Was there appropriate statistical analysis? C9. Was the response rate adequate, and if not, was the low response rate managed appropriately?

S3.2 ROB assessment for the quality of included analytical cross sectional studies in JBI Seale.

Study ID	C1	C2	C3	C4	C5	C6	C7	C8	Quality Score
Chu 2023[1]	Yes	Yes	Yes	Yes	Yes	Yes	Yes	Yes	8
Deng 2019[3]	Yes	Yes	Yes	Yes	Yes	Yes	Yes	Yes	8
Jia 2022[9]	Yes	Yes	Yes	Yes	Yes	Unclear	Unclear	Yes	6
Liu 2024[14]	Yes	Yes	Yes	Yes	Yes	Yes	Unclear	Yes	7
Shen 2019[21]	Yes	Yes	Yes	Yes	Yes	Unclear	Unclear	Yes	6
Wu 2021[24]	Yes	Yes	Yes	Yes	Yes	Yes	Yes	Yes	8
Yu 2023[26]	Yes	Yes	Yes	Yes	Yes	Yes	Yes	Yes	8
Zhang 2023a[30]	Yes	Yes	Yes	Yes	Yes	Yes	Yes	Unclear	7
Zheng 2016[32]	Yes	Yes	Yes	Yes	Yes	Unclear	Unclear	Yes	6
Zhou 2023[33]	Yes	Yes	Yes	Yes	Yes	Yes	Unclear	Yes	7
Zou 2022[36]	Yes	Yes	Yes	Yes	Yes	Yes	Unclear	Yes	7
Bondar 2021[37]	Yes	Yes	Yes	Yes	Yes	Yes	Unclear	Yes	7
Cai 2021[38]	Yes	Yes	Yes	Yes	Yes	Yes	Unclear	Yes	7
Etemadifar 2020[45]	Yes	Yes	Yes	Yes	Yes	Unclear	Unclear	Yes	6
Hu 2022[47]	Yes	Yes	Yes	Yes	Yes	Unclear	Yes	Unclear	6
Kim 2020[50]	Yes	Yes	Yes	Yes	Yes	Unclear	Unclear	Unclear	5
Ravi 2019[58]	Yes	Yes	Yes	Yes	Unclear	Unclear	Unclear	Unclear	4
Sato 2011[59]	Yes	Yes	Yes	Yes	Yes	Yes	Unclear	Yes	7
Zhou 2022[65]	Yes	Yes	Yes	Yes	Yes	Yes	Yes	Yes	8
Yong 2009[67]	Yes	Yes	Yes	Yes	Yes	Yes	Yes	Yes	8
Zheng 2017[74]	Yes	Yes	Yes	Yes	Yes	Unclear	Unclear	Yes	6
Huang 2023[79]	Yes	Yes	Yes	No	Yes	Yes	Yes	Yes	7
Li 2022a[81]	Yes	Yes	Yes	No	Yes	Yes	Yes	Yes	7
Zou 2021[82]	Yes	Yes	Yes	No	Yes	Yes	Yes	Yes	7
Zhang 2023b[83]	Yes	Yes	Yes	No	Yes	Yes	Yes	Yes	7
Birgani 2006[84]	Yes	Yes	Yes	Yes	Unclear	Unclear	Unclear	Unclear	4
Freire 2008[87]	Yes	Yes	Yes	No	Yes	Unclear	Unclear	Yes	5
Li 2020[100]	Yes	Yes	Yes	No	Yes	Unclear	Unclear	Yes	5
Santo 2011[116]	Yes	Yes	Yes	No	Yes	Unclear	Unclear	Yes	5
Sanchez 2010[119]	Yes	Yes	Yes	No	No	Unclear	Unclear	Yes	4
Safikhani 2006[120]	Yes	Yes	Yes	No	Yes	Unclear	Unclear	Yes	5
Ciaccia 2017[121]	Yes	Yes	Yes	No	Yes	Yes	Unclear	Yes	6
Petrovic 2012[122]	Yes	Yes	Yes	No	Yes	Yes	Unclear	Yes	6
Wang 2018a[124]	Yes	Yes	Yes	Yes	Yes	Yes	Yes	Yes	8
Wang 2022b[127]	Yes	Yes	Yes	No	Yes	Unclear	Unclear	Yes	5
Wang 2007[128]	No	Yes	Yes	Yes	Yes	Unclear	Yes	Unclear	5
Yang 2022a[131]	Yes	Yes	Yes	Yes	Yes	Unclear	Yes	Yes	7
Yu 2010[132]	No	Yes	Yes	No	Yes	Yes	Yes	Yes	6
Zhang 2023c[133]	Yes	Yes	Yes	No	Yes	Unclear	Unclear	Yes	5
Zhao 2022[135]	Yes	Yes	Yes	No	Yes	Yes	Yes	Yes	7
Assiri 2019[138]	No	Yes	Yes	No	Yes	Yes	Unclear	Yes	5
Scaturro 2021[150]	Yes	Yes	Yes	Yes	Yes	Yes	Unclear	Yes	7

Guo 2023[151]	Yes	Yes	Yes	No	Yes	Yes	Yes	Yes	7
Hai 2021[152]	Yes	Yes	Yes	No	Yes	Yes	Unclear	Yes	6
Li 2023b[156]	Yes	Yes	Yes	No	Yes	Yes	Unclear	Yes	6
Baroni 2015[165]	Yes	Yes	Yes	No	Yes	Yes	Yes	Yes	7
Gashaw 2021[166]	Yes	Yes	Yes	No	Yes	Yes	Yes	Yes	7
Wong 2005[168]	Yes	Yes	Yes	Yes	Yes	Unclear	Unclear	Unclear	5
Chen 2021[175]	Yes	Yes	Yes	No	Yes	Yes	Unclear	Yes	6
Din 2021[191]	Yes	Yes	Yes	No	Yes	Unclear	Yes	Yes	6
Minghelli 2014[196]	Yes	Yes	Yes	No	Yes	Unclear	Unclear	Yes	5
Zou 2022b[197]	Yes	Yes	Yes	No	Yes	Yes	Yes	Yes	7
Sung 2021[199]	No	Yes	Yes	Yes	Yes	Unclear	Unclear	Yes	5
Sugita 2000[200]	Unclear	Yes	Yes	Yes	Yes	Unclear	Yes	Yes	6
Wei 2018[201]	Yes	Yes	Yes	No	Yes	Unclear	Yes	Yes	6
Dantas 2021[202]	Yes	Yes	Yes	Yes	Yes	Unclear	Yes	Yes	7
Penha 2018[203]	Yes	Yes	Yes	Yes	Yes	Yes	Unclear	Yes	7
Sun 2021[205]	Yes	Yes	Yes	No	Yes	Yes	Yes	Yes	7
Jian 2024[206]	Yes	Yes	Yes	No	Yes	Unclear	Yes	Yes	6
Wen 2021b[207]	Yes	Yes	Yes	No	Yes	Unclear	Yes	Yes	6
Tang 2011b[216]	Yes	Yes	Yes	Yes	Yes	Unclear	Yes	Yes	7
Rocha 2012[220]	Yes	Yes	Yes	No	Yes	Unclear	Unclear	Yes	5
Pereira 2005[221]	Yes	Yes	Yes	No	Yes	Unclear	Unclear	Unclear	4
Bertolini 1997[223]	Yes	Yes	Yes	No	Yes	Unclear	Unclear	Yes	5
Liu 2021[225]	No	Yes	Yes	No	Yes	Unclear	Yes	Yes	5
Singh 2022[227]	Yes	Yes	Yes	Yes	Yes	Unclear	Yes	Yes	7

Note: JBI, Joanna Briggs Institute; C1.Were the criteria for inclusion in the sample clearly defined C2.Were the study subjects and the setting described in detail? C3. Was the exposure measured in a valid and reliable way? C4. Were objective, standard criteria used for measurement of the condition? C5. Were confounding factors identified? C6. Were strategies to deal with confounding factors stated? C7. Were the outcomes measured in a valid and reliable way? C8. Was appropriate statistical analysis used?

S3.3 ROB assessment for the quality of included case control studies in NOS Seale.

Study ID	C1	C2	C3	C4	C5	C6	C7	C8	C9	Quality Score
Krześniak 2022[235]	1	1	0	1	1	0	1	1	0	6
Yang 2022b[44]	1	1	1	1	1	0	1	1	0	7
Laskowska 2019[236]	1	1	0	1	0	0	1	1	0	5
Lee 2005[237]	1	1	1	1	1	0	1	1	0	7
McMaster 2015[53]	1	1	1	0	1	0	1	1	0	6
Pjanic 2024[239]	1	1	1	1	1	0	1	1	0	7
Tam 2016[238]	1	1	1	1	1	0	1	1	0	7
Yan 2020[64]	1	1	1	1	1	0	1	1	0	7
Zaina 2013[233]	1	1	0	1	1	0	1	1	0	6
Zhang 2021[68]	1	1	0	1	1	0	1	1	0	6
Zhu 2023[69]	1	1	1	1	1	0	1	1	1	8
Kesak-Ursic 2021[234]	1	1	0	1	1	0	1	1	0	6
Willner 1984b[188]	0	1	1	1	1	0	1	1	0	6
Deng 2016[176]	0	1	1	1	1	0	1	1	0	6

Note: NOS, Newcastle-ottawa quality assessment scale; C1. Is the case definition adequate? C2. Representativeness of the cases. C3. Selection of Controls. C4. Definition of Controls. C5. study controls for the most important factor. C6. study controls for any additional factor. C7. Ascertainment of exposure. C8. Same method of ascertainment for cases and controls. C9. Non-Response rate.

S3.4 ROB assessment for the quality of included cohort studies in NOS Seale.

Study ID	C1	C2	C3	C4	C5	C6	C7	C8	C9	Quality Score
Tobias 2019[185]	1	1	1	1	1	0	1	1	0	7
Ueno 2011[186]	1	1	1	1	1	0	1	1	1	8
Liu 2003[103]	1	1	1	0	1	0	1	0	1	6
Robitaille 1984[92]	1	1	1	0	1	0	1	0	1	6
Dickson 1980[86]	1	1	1	0	1	0	1	1	0	6
Nissinen 1993[72]	1	1	1	0	1	0	1	0	1	6

Note: NOS, Newcastle-ottawa quality assessment scale; C1. Representativeness of the exposed cohort. C2. Selection of the non exposed cohort. C3. Ascertainment of exposure. C4. Demonstration that outcome of interest was not present at start of study. C5. study controls for the most important factor. C6. study controls for any additional factor. C7. Assessment of outcome. C8. Was follow-up long enough for outcomes to occur. C9. Adequacy of follow up of cohorts.

Appendix S4: Reported prevalence of suspected scoliosis in children and adolescents in 39 countries with available data.

S4.1 Prevalence of suspected scoliosis in children and adolescents in 39 countries.

Country	No. of articles	No. of participants	Case of suspected scoliosis in children and adolescents	Prevalence of suspected scoliosis in children and adolescents (%, 95%CI)
Overall	186	7933594	317160	5.80 [4.83; 6.86]
Australia	2	51055	1004	2.76 [1.06; 5.22]
Bosnia and Herzegovina	1	2517	298	11.84 [10.60; 13.17]
Brazil	13	10488	1312	22.61 [11.47; 36.17]
Bulgaria	1	4800	57	1.19 [0.90; 1.54]
Canada	3	66089	6062	10.21 [5.71; 15.82]
Chile	1	1200	98	8.17 [6.68; 9.86]
China	93	4526966	143901	3.70 [3.07; 4.37]
Colombia	1	387	160	41.34 [36.39; 46.43]
Croatia	1	18216	1053	5.78 [5.45; 6.13]
Ecuador	1	7943	83	1.04 [0.83; 1.29]
Ethiopia	1	1905	62	3.25 [2.50; 4.15]
Finland	1	896	174	19.42 [16.88; 22.16]
Greece	5	116318	8897	9.80 [7.23; 12.73]
India	3	7100	352	4.94 [4.44; 5.46]
Indonesia	1	784	50	6.38 [4.77; 8.32]
Iran	5	5714	483	7.71 [4.17;12.20]
Israel	1	24846	69	0.28 [0.22; 0.35]
Italy	3	10457	307	6.68 [1.22; 15.95]
Japan	3	368884	6079	2.00 [1.34; 2.78]
Korea	6	1611267	85773	4.30 [0.86; 10.16]
Malaysia	3	43978	805	3.34 [0.99; 6.99]
Malta	1	245	13	5.31 [2.86; 8.90]
Netherlands	1	30563	3065	10.03 [9.69; 10.37]
Nigeria	1	410	30	7.32 [4.99; 10.28]
Norway	1	4000	60	1.50 [1.15; 1.93]
Peru	1	191	52	27.23 [21.05; 34.12]
Poland	1	191	131	68.59 [61.49; 75.10]
Portugal	1	966	147	15.22 [13.01; 17.64]
Russia	1	4038	687	17.01 [15.87; 18.21]
Saudi Arabia	3	226605	1216	5.91 [0.00; 21.90]
Serbia	2	14863	1336	7.44 [4.28; 11.37]
Singapore	2	166325	5665	2.75 [0.16; 8.32]
Slovakia	1	680	60	8.82 [6.80; 11.21]
Spain	3	3889	538	7.36 [0.85; 19.29]
Sweden	3	38701	1316	7.04 [0.38; 20.86]
Thailand	1	1818	162	8.91 [7.64; 10.32]
Turkey	5	33893	2788	3.26 [0.24; 9.54]
England	3	18199	1132	7.23 [5.63; 9.02]
America	6	506207	41683	6.27 [2.16; 12.29]

S4.2 Global prevalence distribution of suspected scoliosis in children and adolescents in 39 countries.

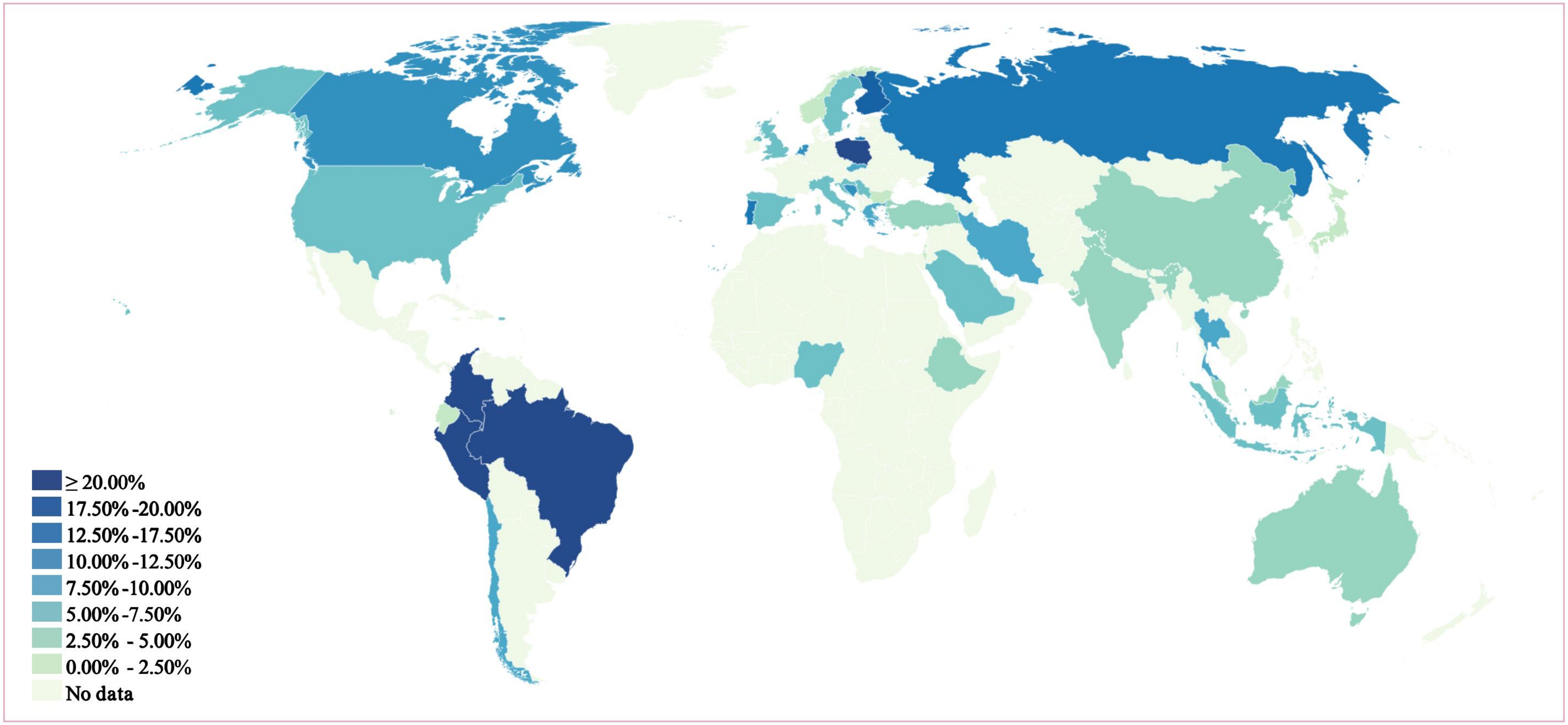

S4.3 Forest plot of suspected scoliosis in children and adolescents in 39 countries.

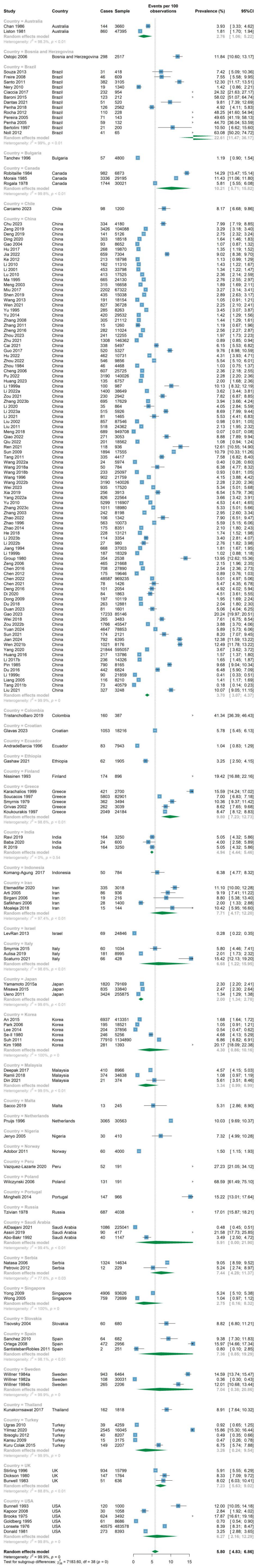

Appendix S5: **Reported prevalence of scoliosis in children and adolescents in 33 countries with available data.**

S5.1 Prevalence of scoliosis in children and adolescents in 33 countries.

Country	No. of articles	No. of participants	Case of scoliosis in children and adolescents	Prevalence of scoliosis in children and adolescents (%, 95%CI)
Overall	150	42901789	236437	1.65 [1.38; 1.94]
Australia	1	3660	7	2.10 [1.66; 2.62]
Bosnia and Herzegovina	1	2517	79	3.14 [2.49; 3.90]
Brazil	5	6919	203	3.83 [1.89; 6.41]
Canada	3	66089	1488	2.70 [1.40; 4.41]
Chile	1	1200	63	5.25 [4.06; 6.67]
China	79	1760062	27301	1.42 [1.12; 1.75]
Croatia	1	18216	894	4.91 [4.60; 5.23]
Ecuador	1	7943	4	0.05 [0.01; 0.13]
Finland	1	896	65	7.25 [5.64; 9.15]
Germany	1	1970	56	2.84 [2.15; 3.68]
Greece	4	112824	2583	2.58 [0.90; 5.10]
India	3	34144	126	0.68 [0.02; 2.18]
Indonesia	1	784	23	2.93 [1.87; 4.37]
Iran	3	4098	28	0.58 [0.35; 0.86]
Israel	1	2380	39	1.64 [1.17; 2.23]
Italy	3	10457	226	3.33 [0.00; 11.93]
Japan	6	468465	3453	0.92 [0.33; 1.78]
Korea	7	38610790	187774	1.06 [0.34; 2.15]
Malaysia	2	43604	361	1.23 [0.02; 4.26]
Netherlands	1	30563	343	1.12 [1.01; 1.25]
Nigeria	1	410	6	1.46 [0.54; 3.16]
Norway	1	4000	22	0.55 [0.34; 0.83]
Romania	1	252	42	16.67 [12.28; 21.85]
Russia	1	4038	187	4.63 [4.00; 5.33]
Saudi Arabia	1	1147	29	2.53 [1.70; 3.61]
Serbia	1	14634	357	2.44 [2.20; 2.70]
Singapore	2	166325	1409	0.74 [0.17; 1.73]
Slovakia	1	680	10	1.47 [0.71; 2.69]
Sweden	1	327	17181	1.90 [1.70; 2.12]
Thailand	1	1818	84	4.62 [3.70; 5.69]
Turkey	3	22511	419	1.06 [0.22; 2.50]
England	5	37564	515	1.68 [0.89; 2.71]
America	6	1443648	7844	2.06 [0.22; 5.68]

S5.2 Forest plot of scoliosis in children and adolescents in 33 countries.

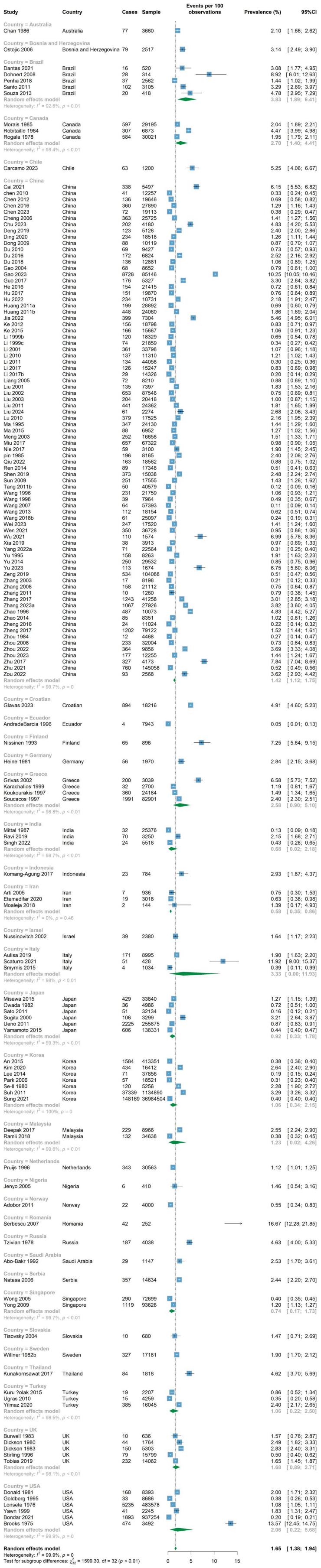

Appendix S6: **Reported prevalence of scoliosis in children and adolescents in 23 China province with available data.**

S6.1 Prevalence of scoliosis in children and adolescents in 23 China province.

Province	No. of articles	No. of participants	Case of scoliosis in children and adolescents	Prevalence of scoliosis in children and adolescents (%, 95%CI)
Overall	78	1739644	29992	1.65 [1.38; 1.94]
Beijing	3	104249	1199	1.24 [0.02; 4.34]
Tianjin	2	23301	531	2.20 [1.68; 2.79]
Shanghai	7	45221	1930	4.47 [3.03; 6.18]
Guangdong	22	606253	5225	0.92 [0.59; 1.31]
Jiangsu	6	200349	2280	0.86 [0.53; 1.27]
Henan	5	207402	2200	1.90 [0.33; 4.71]
Zhejiang	5	55186	1111	2.42 [1.06; 4.30]
Hubei	3	16203	351	3.28 [0.98; 6.84]
Fujian	3	74531	545	0.73 [0.67; 0.79]
Shandong	3	29385	1082	1.95 [0.14; 5.79]
Shaanxi	2	53615	723	1.35 [1.23; 1.47]
Shanxi	2	46694	1173	2.42 [0.73; 5.07]
Sichuan	2	22474	212	1.28 [0.09; 3.78]
Hainan	2	15150	330	2.02 [0.70; 4.00]
Hebei	2	101804	8980	4.96 [0.07; 16.86]
Heilongjiang	2	41887	820	1.98 [1.65; 2.34]
Hunan	1	8165	196	2.40 [2.08; 2.74]
Gansu	1	36728	350	0.95 [0.86; 1.05]
Guizhou	1	17555	251	1.43 [1.26; 1.61]
Inner Mongolia	1	1260	10	0.79 [0.37; 1.37]
Jiangxi	1	10119	88	0.87 [0.70; 1.06]
Liaoning	1	12257	41	0.33 [0.24; 0.45]
Qinghai	1	9856	364	3.69 [3.33; 4.07]

S6.2 Forest plot of scoliosis in children and adolescents in 23 China province.

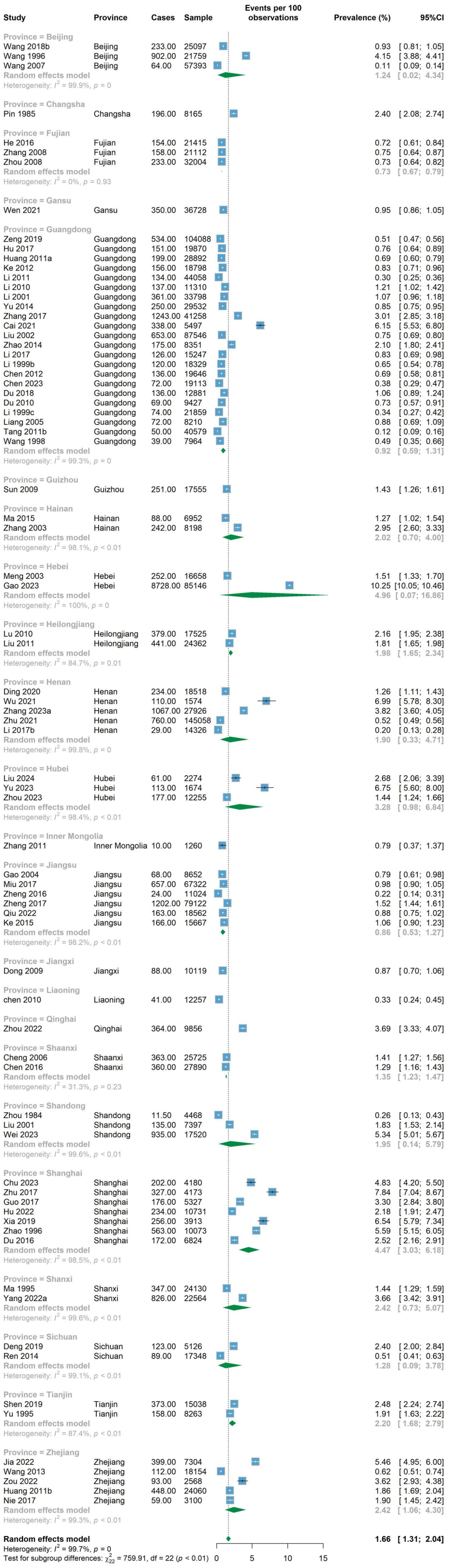

Appendix S7: Reported prevalence of scoliosis in children and adolescents in different investigation periods with available data.

S7.1 Prevalence of scoliosis in children and adolescents in different investigation periods.

Investigation periods	Sex	No. of articles	No. of participants	Case of scoliosis in children and adolescents	Prevalence of scoliosis in children and adolescents (%, 95%CI)
Before 2000					
	Overall	44	1186451	15753	1.86 [1.37; 2.42]
	Boys	21	232203	1747	0.84 [0.61; 1.10]
	Girls	21	230996	3770	1.81 [1.22; 2.51]
2000-2009					
	Overall	27	1877026	43715	1.28 [0.71; 2.02]
	Boys	19	854485	12218	1.02 [0.38; 1.94]
	Girls	19	805595	29579	1.67 [0.81; 2.81]
2010-2019					
	Overall	48	38687981	160917	1.82 [1.34; 2.38]
	Boys	37	19963391	65384	0.96 [0.66; 1.31]
	Girls	37	18554122	91219	1.96 [1.34; 2.69]
2020 and later					
	Overall	10	348924	12055	3.02 [1.53; 4.99]
	Boys	9	105892	2013	1.67 [0.79; 2.86]
	Girls	9	97974	2094	2.68 [1.25; 4.61]

S7.2 Forest plot of scoliosis in children and adolescents in different investigation periods.

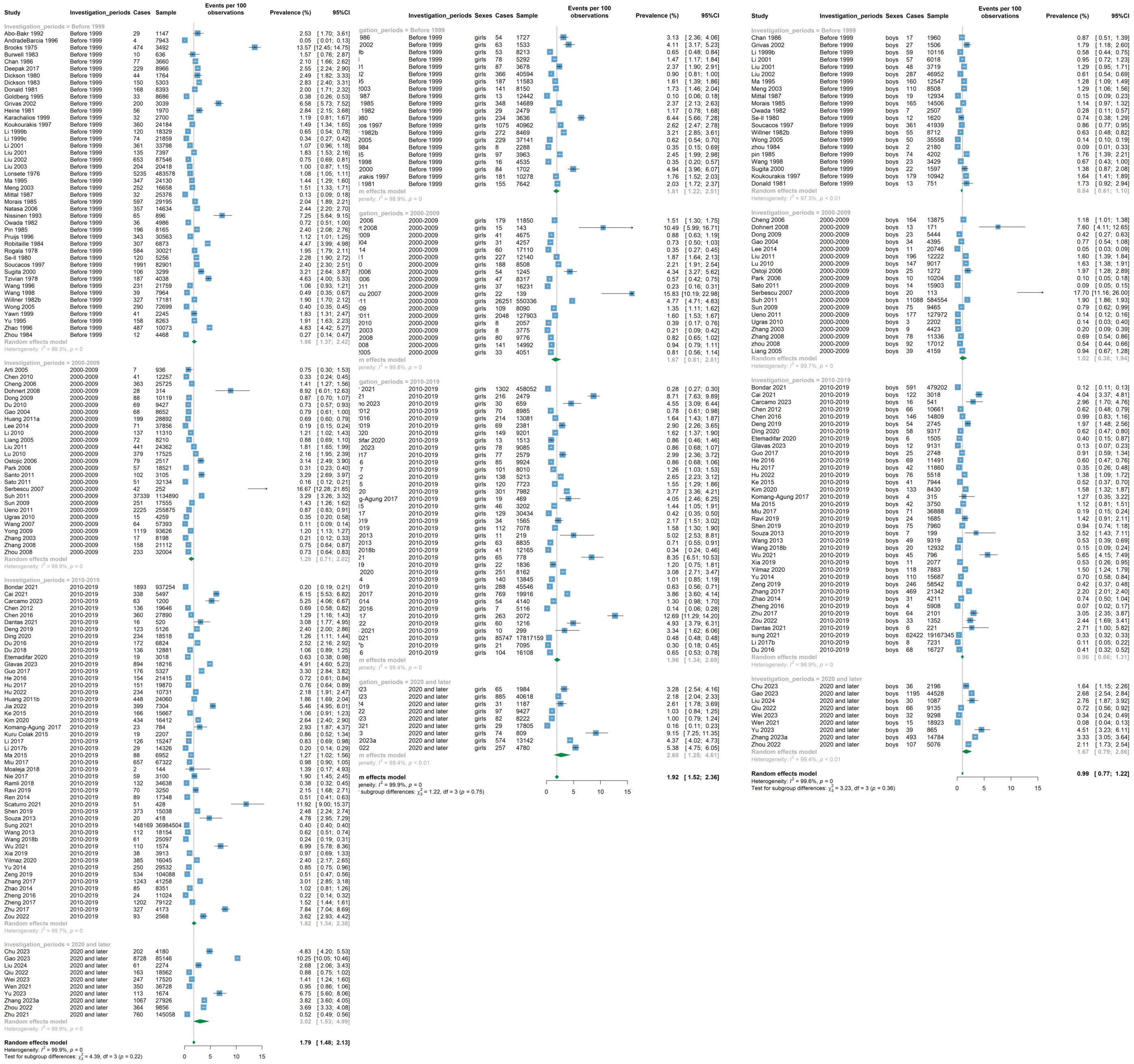

Appendix S8: Reported prevalence of scoliosis in children and adolescents in different WHO region with available data.

S8.1 Prevalence of scoliosis in children and adolescents in different WHO region.

Who region	Sex	No. of articles	No. of participants	Case of scoliosis in children and adolescents	Prevalence of scoliosis in children and adolescents (%, 95%CI)
African Region					
	Overall	1	410	6	1.46 [0.54; 3.16]
	Boys	1	190	1	0.53 [0.01; 2.90]
	Girls	1	220	0	0.00 [0.00; 1.66]
Region of the Americas					
	Overall	17	1526479	9612	2.54 [1.36; 4.06]
	Boys	7	482157	651	2.02 [0.61; 4.13]
	Girls	7	468504	1555	3.16 [1.30; 5.76]
South-East Asia Region					
	Overall	7	170726	1643	1.35 [0.44; 2.75]
	Boys	2	13249	23	0.47 [0.00; 2.24]
	Girls	2	12911	32	1.32 [0.00; 7.93]
European Region					
	Overall	22	226756	5372	2.88 [1.80; 4.20]
	Boys	11	87562	718	1.22 [0.26; 2.83]
	Girls	11	85562	1907	2.75 [1.34; 4.64]
Eastern Mediterranean Region					
	Overall	7	58348	837	1.22 [0.67; 1.93]
	Boys	3	11590	127	0.57 [0.06; 1.56]
	Girls	3	11732	272	1.24 [0.19; 3.14]
Western Pacific Region					
	Overall	96	40919070	218967	1.35 [1.09; 1.64]
	Boys	77	20927252	80490	0.82 [0.64; 1.01]
	Girls	77	19415868	125102	1.62 [1.26; 2.01]

Note: WHO, World Health Organization.

S8.2 Forest plot of scoliosis in children and adolescents in different WHO region.

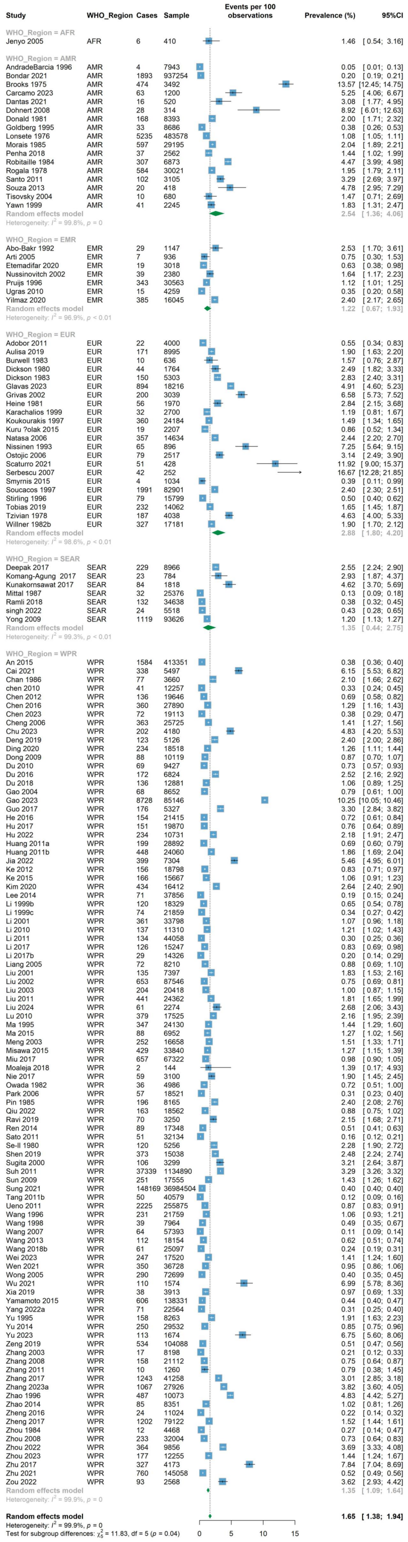

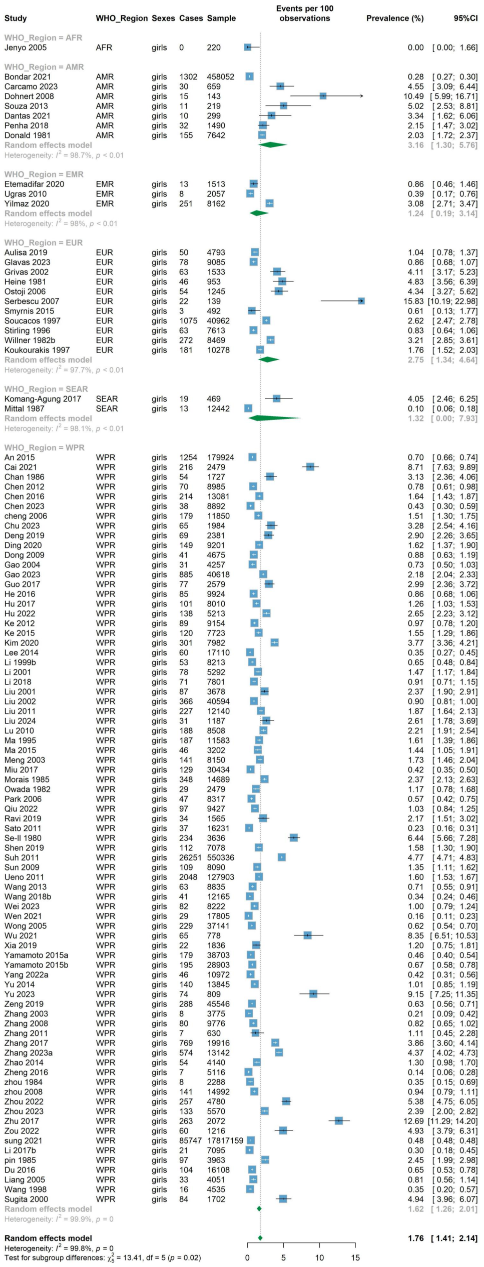

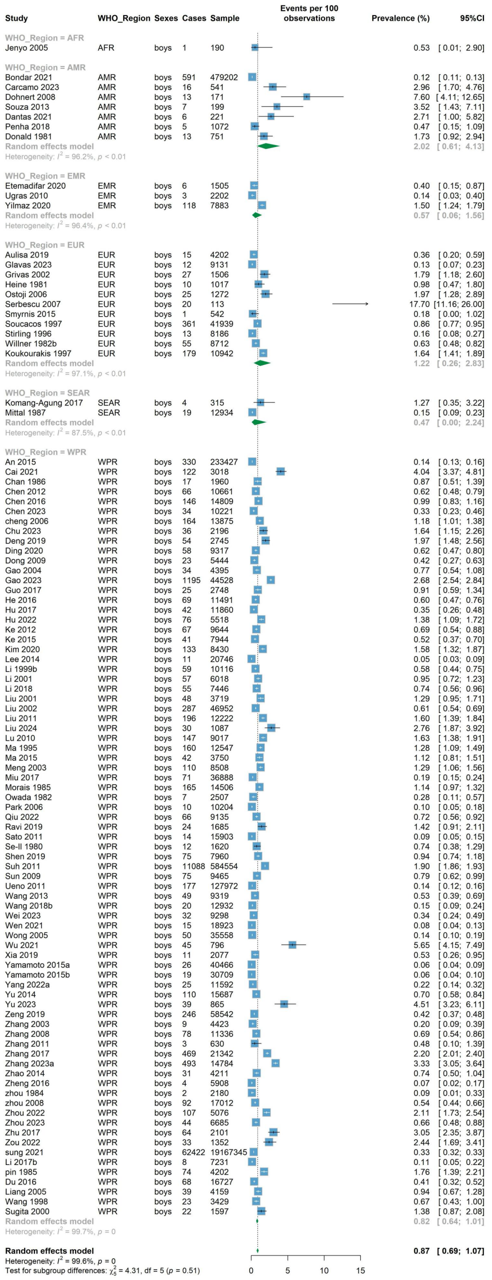

Appendix S9: Reported prevalence of scoliosis in children and adolescents in different World Bank region with available data.

S9.1 Prevalence of scoliosis in children and adolescents in different World Bank region.

World Bank region	Sex	No. of articles	No. of participants	Case of scoliosis in children and adolescents	Prevalence of scoliosis in children and adolescents (%, 95%CI)
High-income countries					
	Overall	48	40999306	207246	1.97 [1.39; 2.65]
	Boys	28	20862031	75575	0.62 [0.30; 1.05]
	Girls	28	19395437	119954	1.85 [1.18; 2.67]
Upper middle-income countries					
	Overall	98	1867929	29059	1.54 [1.24; 1.86]
	Boys	71	649368	6298	0.55 [0.69; 1.07]
	Girls	69	565908	8260	0.46 [0.00; 2.20]
Low- and middle-income countries					
	Overall	4	34554	132	0.81 [0.13; 2.03]
	Boys	3	14809	44	1.00 [0.79; 1.23]
	Girls	3	14227	47	1.79 [1.38; 2.25]

S9.2 Forest plot of scoliosis in children and adolescents in different World Bank region.

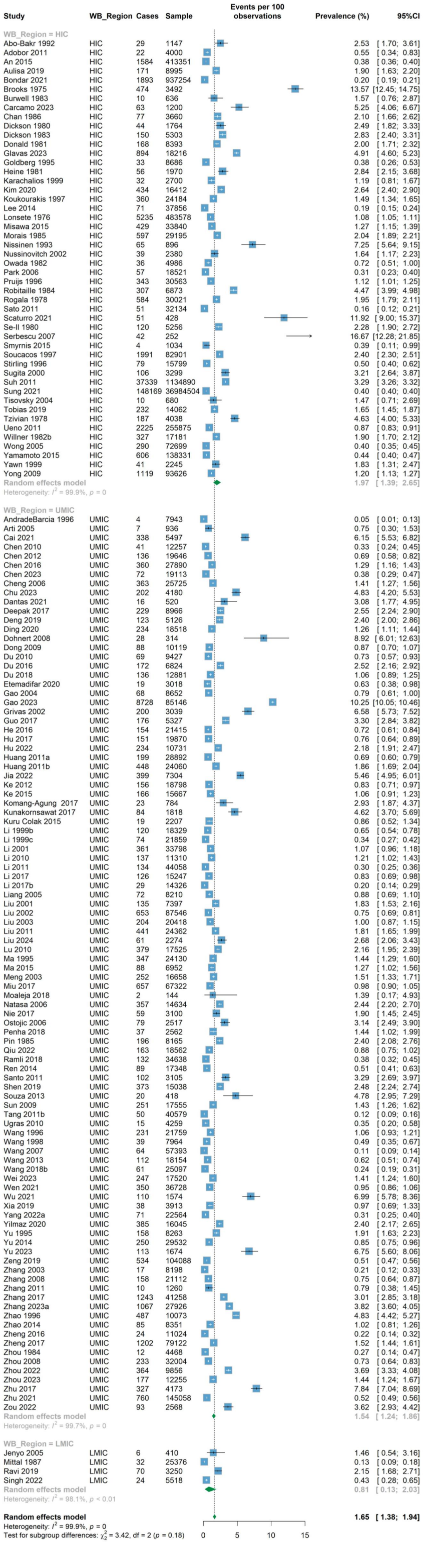

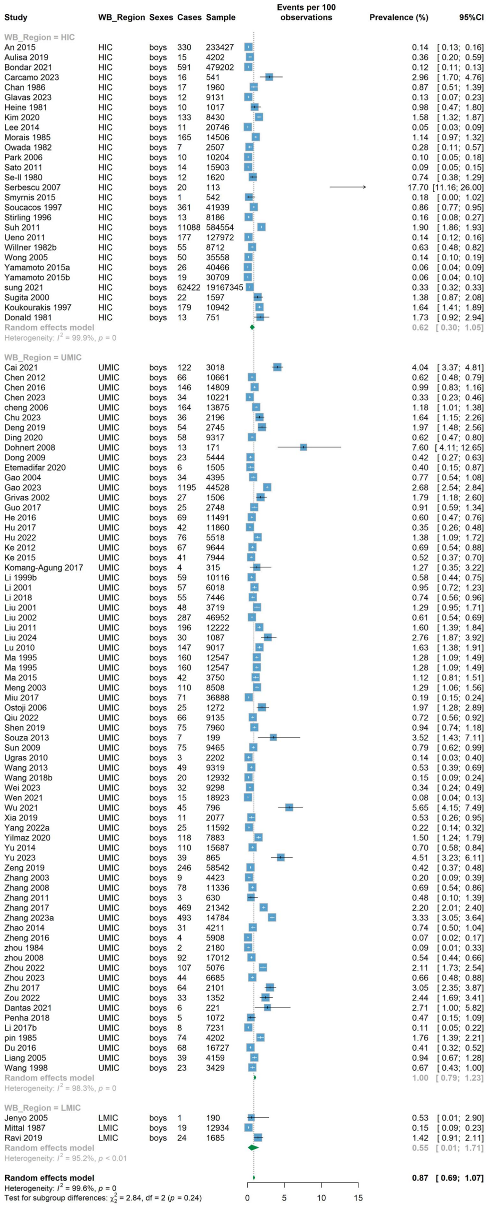

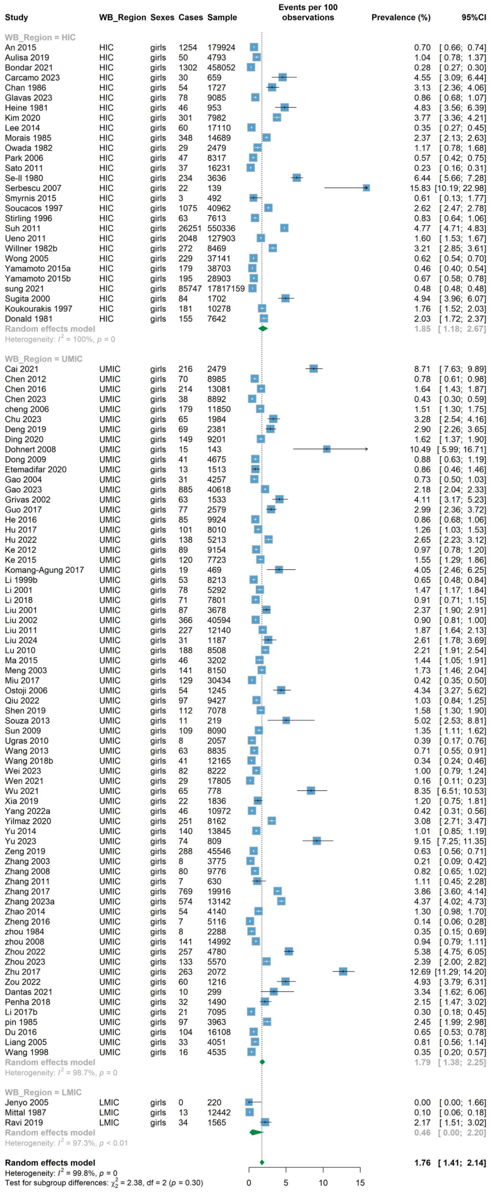

Appendix S10: **Reported prevalence of scoliosis in children and adolescents in boys and girls aged 6-18 years with available data.**

S10.1 Prevalence of scoliosis in children and adolescents in boys and girls aged 6-18 years.

Age	Sex	No. of articles	No. of participants	Case of scoliosis in children and adolescents	Prevalence of scoliosis in children and adolescents (%, 95%CI)
6 years old					
	Overall	3	2745	49	1.31 [0.00; 5.07]
	Boys	3	1355	19	1.04 [0.01; 3.29]
	Girls	3	1390	30	1.69 [0.00; 6.81]
7 years old					
	Overall	14	18558	143	0.51 [0.17; 0.99]
	Boys	14	10050	66	0.45 [0.17; 0.84]
	Girls	14	8508	77	0.52 [0.12; 1.14]
8 years old					
	Overall	14	26035	228	0.59 [0.20; 1.18]
	Boys	14	14151	117	0.61 [0.28; 1.06]
	Girls	14	11884	111	0.51 [0.08; 1.25]
9 years old					
	Overall	15	29139	247	0.65 [0.27; 1.16]
	Boys	15	15584	113	0.53 [0.20; 0.99]
	Girls	15	13555	134	0.76 [0.31; 1.37]
10 years old					
	Overall	16	85166	328	0.70 [0.36; 1.15]
	Boys	16	44874	133	0.63 [0.34; 1.00]
	Girls	16	40292	195	0.75 [0.33; 1.30]
11 years old					
	Overall	16	88186	423	0.77 [0.37; 1..31]
	Boys	16	45803	155	0.61 [0.31; 100]
	Girls	16	42383	268	0.92 [0.40; 1.64]
12 years old					
	Overall	18	99889	621	1.10 [0.54; 1.85]
	Boys	18	52065	209	0.70 [0.44; 1.02]
	Girls	18	47824	413	1.45 [0.55; 2.74]
13 years old					
	Overall	18	133915	1111	1.05 [0.80; 1.34]
	Boys	18	70186	370	0.73 [0.51; 0.98]
	Girls	18	63729	751	1.40 [1.09; 1.74]
14 years old					
	Overall	18	127701	1105	1.10 [0.81; 1.44]
	Boys	18	66313	422	0.77 [0.53; 1.05]
	Girls	18	61388	683	1.45 [1.09; 1.86]
15 years old					
	Overall	18	115369	894	1.15 [0.83; 1.51]
	Boys	18	60539	388	0.96 [0.67; 1.30]
	Girls	18	54830	506	1.30 [0.91; 1.75]

16 years old				
Overall	12	90848	635	1.02 [0.69; 1.40]
Boys	12	47816	249	0.84 [0.48; 1.30]
Girls	12	43032	286	0.97 [0.59; 1.44]
17 years old				
Overall	8	77671	255	0.44 [0.08; 1.01]
Boys	8	40130	106	0.19 [0.00; 0.68]
Girls	8	37541	139	0.18 [0.00; 0.73]
18 years old				
Overall	5	9475	71	0.81 [0.29; 1.54]
Boys	5	4671	30	0.62 [0.10; 1.47]
Girls	5	4804	41	0.88 [0.32; 1.66]

S10.2 Forest plot of scoliosis in children and adolescents in boys and girls aged 6-18 years.

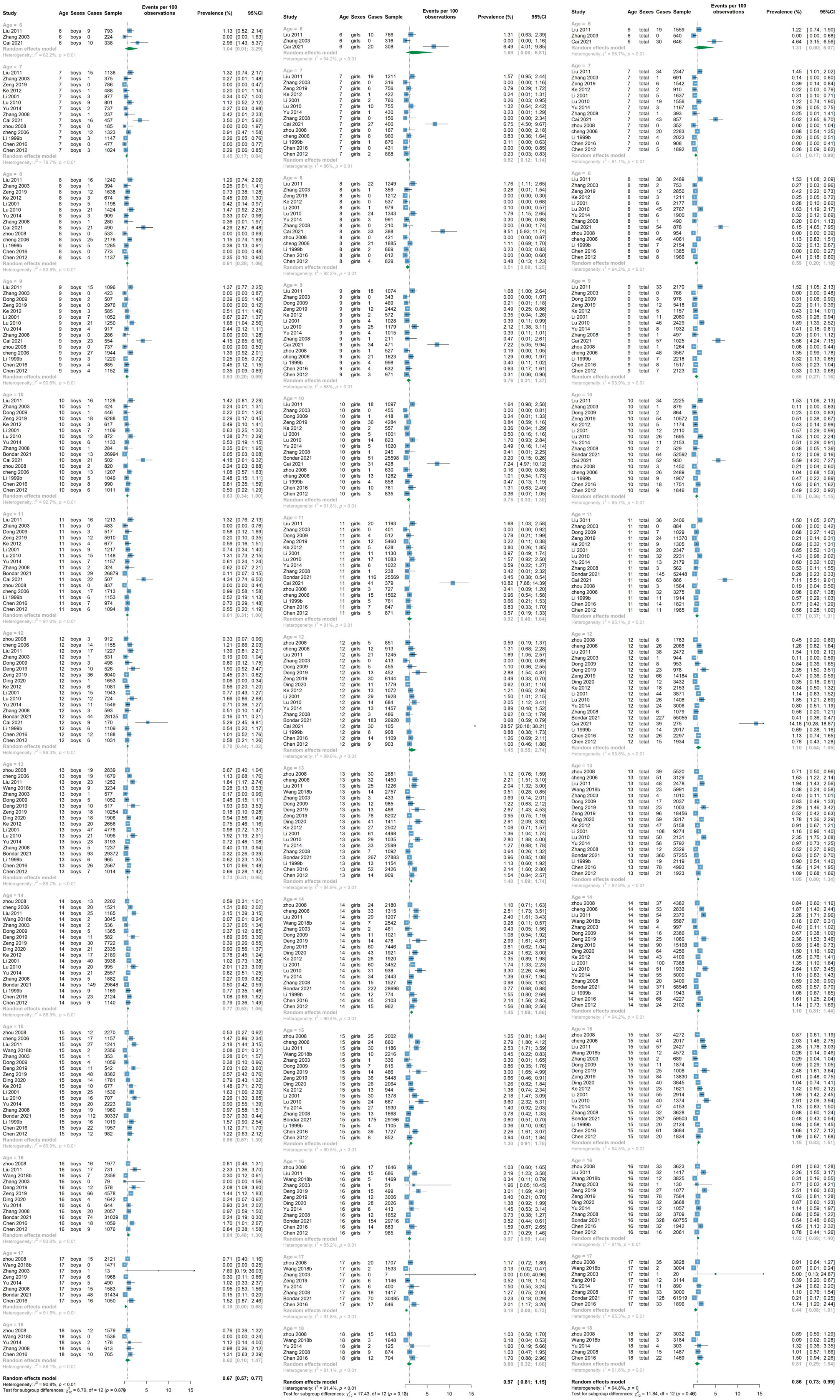

Appendix S11: **The proportion of different aetiological types with available data.**

Study ID	Confirmed case (n)	Idiopathic scoliosis	Nonidiopathic scoliosis	Study ID	Confirmed case (n)	Idiopathic scoliosis	Nonidiopathic scoliosis
Chen 2010[193]	41	92.68%	7.32%	Lu 2010[15]	335	92.84%	7.16%
Cheng 2006[77]	343	93.59%	6.41%	Ma 1995[17]	347	90.20%	9.80%
Deng 2019[3]	123	86.99%	13.01%	Meng 2003[18]	251	89.64%	10.36%
Ding 2020[4]	234	88.46%	11.54%	Mittal 1987[148]	32	21.88%	78.13%
Dohnert 2008[42]	28	35.71%	64.29%	Miu 2017[19]	200	97.50%	2.50%
Dong 2009[178]	64	96.88%	3.13%	Pin 1985[211]	171	97.66%	2.34%
Gao 2004[5]	65	96.92%	3.08%	Scaturro 2021[150]	47	100.00%	0.00%
He 2016[6]	154	100.00%	0.00%	Shen 2019[21]	187	100.00%	0.00%
Hu 2017[7]	151	94.70%	5.30%	Smyrnis 2015[61]	4	75.00%	25.00%
Ke 2012[10]	156	96.15%	3.85%	Wang 1996[126]	231	87.45%	12.55%
Ke 2015[155]	166	96.99%	3.01%	Wang 2007[128]	64	92.19%	7.81%
Kunakornsawat 2017[52]	84	96.43%	3.57%	Yilmaz 2020[66]	380	97.11%	2.89%
Li 1999b[160]	112	95.54%	4.46%	Zeng 2019[2]	534	89.89%	10.11%
Li 2001[13]	361	96.95%	3.05%	Zhang 2008[28]	158	96.84%	3.16%
Li 2010[12]	137	97.81%	2.19%	Zhang 2017[31]	1243	99.60%	0.40%
Liang 2005[215]	72	93.06%	6.94%	Zhao 2014[137]	85	95.29%	4.71%
Liu 2002[105]	653	96.94%	3.06%	Zhou 2022[65]	364	81.04%	18.96%
Liu 2011[106]	423	87.00%	13.00%				

Note: Nonidiopathic scoliosis, such as congenital, neuromuscular, or miscellaneous scoliosis.

Appendix S12: **The proportion of different Cobb angles with available data.**

Study ID	10°-19°(%)	20°-39°(%)	≥40°(%)	Study ID	10°-19°(%)	20°-39°(%)	≥40°(%)	Study ID	10°-19°(%)	20°-39°(%)	≥40°(%)
Jenyo 2005[46]	100.00	0.00	0.00	Zhao 2014[137]	84.71	12.9	2.35	Yu 2014[27]	74.80	25.20	0.00
Zhang 2003[134]	100.00	0.00	0.00	Chen 2016[171]	83.06	15.00	1.94	Lee 2014[89]	74.65	25.35	0.00
Du 2016[212]	94.19	5.81	0.00	Li 1999b[160]	83.04	12.50	4.46	Park 2006[54]	73.68	26.32	0.00
Zhang 2017[31]	92.08	7.27	0.65	Ke 2012[10]	82.69	13.46	3.85	Li 2017[159]	73.02	18.25	8.73
Li 1999c[214]	91.89	5.41	2.70	Grivas 2002[147]	82.22	17.78	0.00	Ugras 2010[63]	72.73	27.27	0.00
Meng 2003[18]	90.67	8.44	0.89	Xia 2019[130]	81.82	18.18	0.00	An 2015[49]	72.55	26.58	0.87
Du 2018[179]	85.19	14.07	0.74	Zhang 2008[28]	81.65	13.29	5.06	Wang 2018b[125]	70.49%	27.87	1.64
Liu 2024[14]	90.16	8.20	1.64	Zhou 2008[76]	79.40	20.17	0.43	Nussinovitch 2002[55]	69.23	30.77	0.00
Chen 2010[193]	68.42	23.68	7.89	Liang 2005[215]	79.17	18.06	2.78	Ostojic 2006[56]	89.87	10.13	0.00
Deng 2019[3]	89.43	8.94	1.63	Hu 2017[7]	79.02	18.18	2.80	Wen 2021[23]	68.18	29.55	2.27
Gao 2004[5]	89.23	9.23	1.54	Ding 2020[4]	78.63	14.10	7.26	Aulisa 2019[70]	67.69	32.31	0.00
Suh 2011[167]	89.09	10.46	0.44	He 2016[6]	77.92	22.08	0.00	Yong 2009[67]	67.38	28.87	3.75
Li 2001[13]	88.92	8.86	2.22	Zeng 2019[2]	77.53	22.47	0.00	Willner 1982b[189]	66.97	27.52	5.50
Li 2010[12]	88.32	9.49	2.19	Deepak 2017[85]	77.01	18.39	4.60	Soucacos 1997[143]	87.40	11.84	0.77
Chu 2023[1]	88.12	9.90	1.98	Shen 2019[21]	77.01	22.99	0.00	Kuru Çolak 2015[146]	63.64	27.27	9.09
Liu 2002[105]	87.14	9.49	3.37	Dong 2009[178]	76.56	15.63	7.81	Ke 2015[155]	60.25	36.02	3.73
Miu 2017[19]	86.00	11.50	2.50	Carcamo 2023[39]	76.09	19.57	4.35	Li 2017b[210]	55.17	27.59	17.24
Zhu 2017[35]	85.93	14.07	0.00	Chen 2012[172]	75.74	20.59	3.68	Chan 1986[40]	54.93	45.07	0.00
Qiu 2022[109]	85.28	13.50	1.23	Donald 1981[231]	75.60	20.24	4.17	Wang 2007[128]	52.54	33.90	13.56
Wei 2023[129]	75.44	22.81	1.75	Huang 2011a[8]	50.25	43.22	6.53	Yilmaz 2020[66]	90.51	8.67	0.81
Komang-Agung 2017[51]	65.22	21.74	13.04	Kunakornsawat 2017[52]	75.31	24.69	0.00	Glavas 2023[88]	50.00	37.78	12.22

Appendix S13: **The proportion of different scoliosis curve types with available data.**

Study ID	Single curve Thoracolumbar	Single curve Thorax	Single curve Lumbar	Double curve	Triple curve	Study ID	Single curve Thoracolumbar	Sngle curve Thorax	single curve Lumbar	Double curve	Triple curve
Li 2017b[210]	72.41%	17.24%	10.34%	0.00%	0.00%	Li 2001[13]	32.96%	28.53%	27.42%	11.08%	0.00%
Park 2006[54]	68.42%	12.28%	3.51%	15.79%	0.00%	Stirling 1996[62]	32.89%	57.89%	9.21%	0.00%	0.00%
Brooks 1975[140]	66.67%	8.28%	13.38%	11.68%	0.00%	Meng 2003[18]	32.89%	25.78%	21.78%	19.56%	0.00%
Lee 2014[89]	60.56%	18.31%	5.63%	15.49%	0.00%	Ravi 2019[58]	32.76%	50.00%	17.24%	0.00%	0.00%
Chu 2023[1]	60.40%	22.77%	0.99%	15.84%	0.00%	Willner 1982b[189]	31.19%	44.34%	21.41%	3.06%	0.00%
Xia 2019[130]	48.48%	30.30%	9.09%	12.12%	0.00%	Du 2016[212]	30.81%	39.53%	19.77%	9.88%	0.00%
Li 2017[159]	45.24%	32.54%	13.49%	8.73%	0.00%	Chen 2010[193]	28.95%	28.95%	26.32%	15.79%	0.00%
Qiu 2022[109]	42.94%	33.13%	19.02%	4.91%	0.00%	Grivas 2002[147]	26.70%	20.00%	20.00%	NA	NA
Zhao 2014[137]	41.98%	35.80%	19.75%	2.47%	0.00%	Donald 1981[231]	27.38%	39.29%	19.64%	13.69%	0.00%
Zhang 2003[134]	41.18%	23.53%	17.65%	17.65%	0.00%	Etemadifar 2020[45]	26.32%	15.79%	42.11%	15.79%	0.00%
Wong 2005[168]	40.07%	33.33%	7.87%	NA	NA	Shen 2019[21]	26.20%	24.06%	22.99%	26.74%	0.00%
Chen 2016[171]	39.44%	32.22%	21.11%	7.22%	0.00%	Aulisa 2019[70]	26.15%	13.85%	24.62%	35.38%	0.00%
Ostojic 2006[56]	39.24%	39.24%	3.80%	17.72%	0.00%	Wang 2007[128]	25.42%	32.20%	25.42%	16.95%	0.00%
Li 1999c[214]	39.19%	44.59%	13.51%	2.70%	0.00%	Yilmaz 2020[66]	19.02%	23.10%	27.17%	29.35%	1.36%
Hu 2017[7]	39.16%	31.47%	19.58%	9.79%	0.00%	Mittal 1987[148]	21.88%	75.00%	3.13%	0.00%	0.00%
Liu 2002[105]	39.05%	33.23%	20.52%	7.20%	0.00%	Wang 1996[126]	19.03%	58.85%	18.14%	3.98%	0.00%
Yong 2009[67]	17.35%	36.31%	2.42%	39.62%	4.29%	Liu 2024[14]	16.39%	42.62%	19.67%	21.31%	0.00%
Wen 2021[23]	20.93%	32.56%	20.93%	20.93%	4.65%	He 2016[6]	14.94%	27.92%	18.83%	38.31%	0.00%

Ke 2012[10]	34.62%	31.41%	26.92%	7.05%	0.00%	Kuru Çolak 2015[146]	9.09%	27.27%	18.18%	45.45%	0.00%
Soucacos 1997[143]	34.33%	18.18%	33.08%	14.42%	0.00%	Kunakornsawat 2017[52]	35.80%	39.51%	24.69%	0.00%	0.00%

Appendix S14: Forest plot of subgroup.

S14.1 Investigation periods.

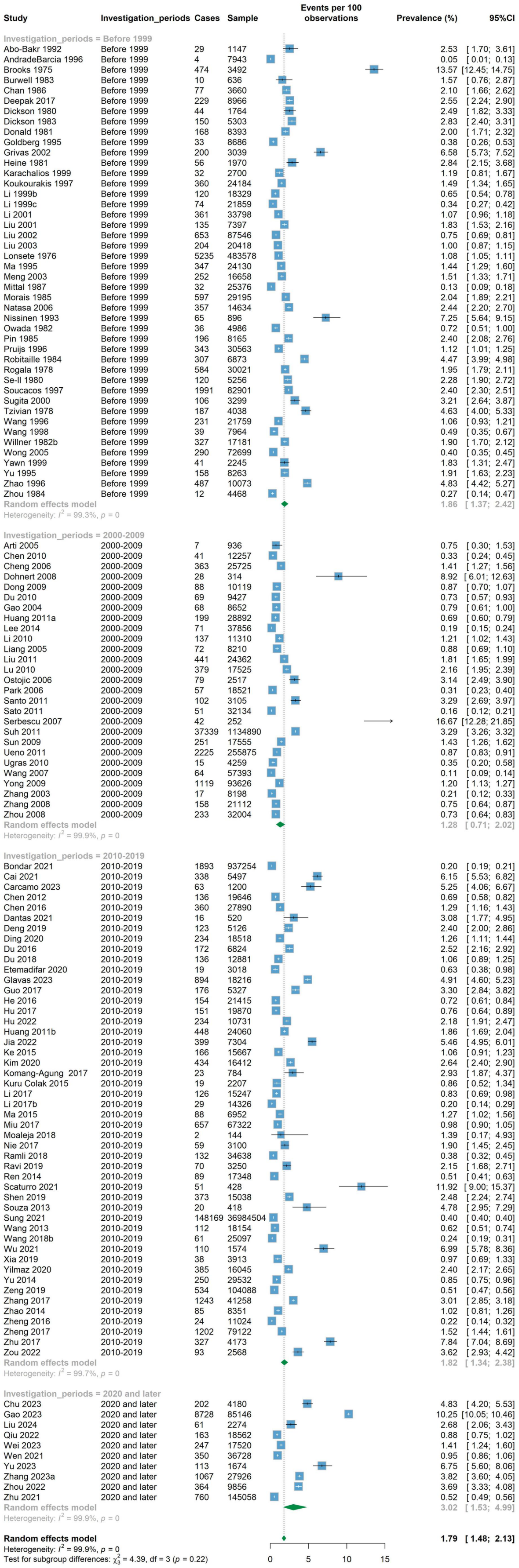

S14.2 Sexes.

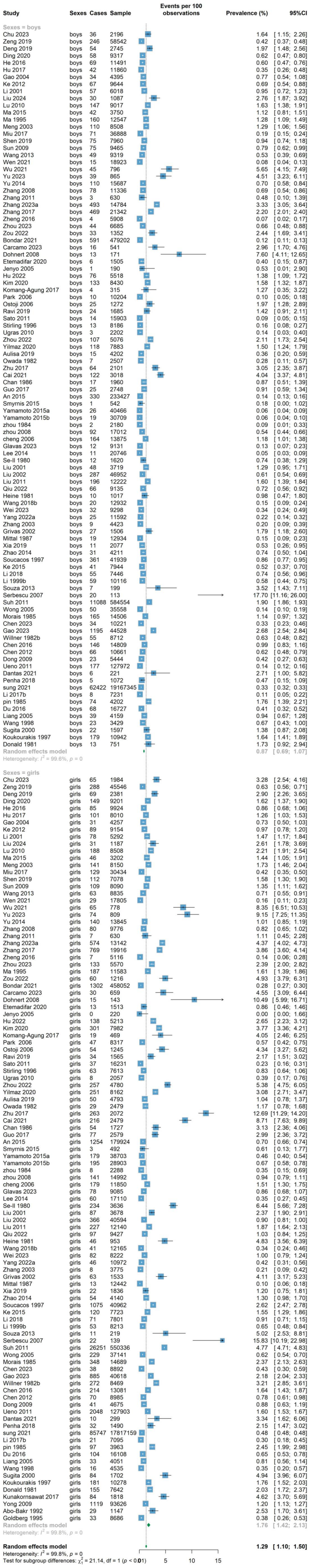
Study Sexes Cases Sample
Events per 100 observations
Prevalence (%) 95%CI
Sexes = boys
Chu 2023 boys 36 2196 1.64 [1.15; 2.26]
Zeng 2019 boys 246 58542 0.42 [0.37; 0.48]
Deng 2019 boys 54 2745 1.97 [1.48; 2.56]
Ding 2020 boys 58 9317 0.62 [0.47; 0.80]
He 2016 boys 69 11491 0.60 [0.47; 0.76]
Hu 2017 boys 42 11860 0.35 [0.26; 0.48]
Gao 2004 boys 34 4395 0.77 [0.54; 1.08]
Ke 2012 boys 67 9644 0.69 [0.54; 0.88]
Li 2001 boys 57 6018 0.95 [0.72; 1.23]
Liu 2024 boys 30 1087 2.76 [1.87; 3.92]
Lu 2010 boys 147 9017 1.63 [1.38; 1.91]
Ma 2015 boys 42 3750 1.12 [0.81; 1.51]
Ma 1995 boys 160 12547 1.28 [1.09; 1.49]
Meng 2003 boys 110 8508 1.29 [1.06; 1.56]
Miu 2017 boys 71 36888 0.19 [0.15; 0.24]
Shen 2019 boys 75 7960 0.94 [0.74; 1.18]
Sun 2009 boys 75 9465 0.79 [0.62; 0.99]
Wang 2013 boys 49 9319 0.53 [0.39; 0.69]
Wen 2021 boys 15 18923 0.08 [0.04; 0.13]
Wu 2021 boys 45 796 5.65 [4.15; 7.49]
Yu 2023 boys 39 865 4.51 [3.23; 6.11]
Yu 2014 boys 110 15687 0.70 [0.58; 0.84]
Zhang 2008 boys 78 11336 0.69 [0.54; 0.86]
Zhang 2011 boys 3 630 0.48 [0.10; 1.39]
Zhang 2023a boys 493 14784 3.33 [3.05; 3.64]
Zhang 2017 boys 469 21342 2.20 [2.01; 2.40]
Zheng 2016 boys 4 5908 0.07 [0.02; 0.17]
Zhou 2023 boys 44 6685 0.66 [0.48; 0.88]
Zou 2022 boys 33 1352 2.44 [1.69; 3.41]
Bondar 2021 boys 591 479202 0.12 [0.11; 0.13]
Carcamo 2023 boys 16 541 2.96 [1.70; 4.76]
Dohnert 2008 boys 13 171 7.60 [4.11; 12.65]
Etemadifar 2020 boys 6 1505 0.40 [0.15; 0.87]
Jenyo 2005 boys 1 190 0.53 [0.01; 2.90]
Hu 2022 boys 76 5518 1.38 [1.09; 1.72]
Kim 2020 boys 133 8430 1.58 [1.32; 1.87]
Komang-Agung 2017 boys 4 315 1.27 [0.35; 3.22]
Park 2006 boys 10 10204 0.10 [0.05; 0.18]
Ostoji 2006 boys 25 1272 1.97 [1.28; 2.89]
Ravi 2019 boys 24 1685 1.42 [0.91; 2.11]
Sato 2011 boys 14 15903 0.09 [0.05; 0.15]
Stirling 1996 boys 13 8186 0.16 [0.08; 0.27]
Ugras 2010 boys 3 2202 0.14 [0.03; 0.40]
Zhou 2022 boys 107 5076 2.11 [1.73; 2.54]
Yilmaz 2020 boys 118 7883 1.50 [1.24; 1.79]
Aulisa 2019 boys 15 4202 0.36 [0.20; 0.59]
Owada 1982 boys 7 2507 0.28 [0.11; 0.57]
Zhu 2017 boys 64 2101 3.05 [2.35; 3.87]
Cai 2021 boys 122 3018 4.04 [3.37; 4.81]
Chan 1986 boys 17 1960 0.87 [0.51; 1.39]
Guo 2017 boys 25 2748 0.91 [0.59; 1.34]
An 2015 boys 330 233427 0.14 [0.13; 0.16]
Smyrnis 2015 boys 1 542 0.18 [0.00; 1.02]
Yamamoto 2015a boys 26 40466 0.06 [0.04; 0.09]
Yamamoto 2015b boys 19 30709 0.06 [0.04; 0.10]
zhou 1984 boys 2 2180 0.09 [0.01; 0.33]
zhou 2008 boys 92 17012 0.54 [0.44; 0.66]
cheng 2006 boys 164 13875 1.18 [1.01; 1.38]
Glavas 2023 boys 12 9131 0.13 [0.07; 0.23]
Lee 2014 boys 11 20746 0.05 [0.03; 0.09]
Se-Il 1980 boys 12 1620 0.74 [0.38; 1.29]
Liu 2001 boys 48 3719 1.29 [0.95; 1.71]
Liu 2002 boys 287 46952 0.61 [0.54; 0.69]
Liu 2011 boys 196 12222 1.60 [1.39; 1.84]
Qiu 2022 boys 66 9135 0.72 [0.56; 0.92]
Heine 1981 boys 10 1017 0.98 [0.47; 1.80]
Wang 2018b boys 20 12932 0.15 [0.09; 0.24]
Wei 2023 boys 32 9298 0.34 [0.24; 0.49]
Yang 2022a boys 25 11592 0.22 [0.14; 0.32]
Zhang 2003 boys 9 4423 0.20 [0.09; 0.39]
Grivas 2002 boys 27 1506 1.79 [1.18; 2.60]
Mittal 1987 boys 19 12934 0.15 [0.09; 0.23]
Xia 2019 boys 11 2077 0.53 [0.26; 0.95]
Zhao 2014 boys 31 4211 0.74 [0.50; 1.04]
Soucacos 1997 boys 361 41939 0.86 [0.77; 0.95]
Ke 2015 boys 41 7944 0.52 [0.37; 0.70]
Li 2018 boys 55 7446 0.74 [0.56; 0.96]
Li 1999b boys 59 10116 0.58 [0.44; 0.75]
Souza 2013 boys 7 199 3.52 [1.43; 7.11]
Serbescu 2007 boys 20 113 17.70 [11.16; 26.00]
Suh 2011 boys 11088 584554 1.90 [1.86; 1.93]
Wong 2005 boys 50 35558 0.14 [0.10; 0.19]
Morais 1985 boys 165 14506 1.14 [0.97; 1.32]
Chen 2023 boys 34 10221 0.33 [0.23; 0.46]
Gao 2023 boys 1195 44528 2.68 [2.54; 2.84]
Willner 1982b boys 55 8712 0.63 [0.48; 0.82]
Chen 2016 boys 146 14809 0.99 [0.83; 1.16]
Chen 2012 boys 66 10661 0.62 [0.48; 0.79]
Dong 2009 boys 23 5444 0.42 [0.27; 0.63]
Ueno 2011 boys 177 127972 0.14 [0.12; 0.16]
Dantas 2021 boys 6 221 2.71 [1.00; 5.82]
Penha 2018 boys 5 1072 0.47 [0.15; 1.09]
sung 2021 boys 62422 19167345 0.33 [0.32; 0.33]
Li 2017b boys 8 7231 0.11 [0.05; 0.22]
pin 1985 boys 74 4202 1.76 [1.39; 2.21]
Du 2016 boys 68 16727 0.41 [0.32; 0.52]
Liang 2005 boys 39 4159 0.94 [0.67; 1.28]
Wang 1998 boys 23 3429 0.67 [0.43; 1.00]
Sugita 2000 boys 22 1597 1.38 [0.87; 2.08]
Koukourakis 1997 boys 179 10942 1.64 [1.41; 1.89]
Donald 1981 boys 13 751 1.73 [0.92; 2.94]
Random effects model 0.87 [0.69; 1.07]
Heterogeneity: I^2 = 99.6%, p = 0
Sexes = girls
Chu 2023 girls 65 1984 3.28 [2.54; 4.16]
Zeng 2019 girls 288 45546 0.63 [0.56; 0.71]
Deng 2019 girls 69 2381 2.90 [2.26; 3.65]
Ding 2020 girls 149 9201 1.62 [1.37; 1.90]
He 2016 girls 85 9924 0.86 [0.68; 1.06]
Hu 2017 girls 101 8010 1.26 [1.03; 1.53]
Gao 2004 girls 31 4257 0.73 [0.50; 1.03]
Ke 2012 girls 89 9154 0.97 [0.78; 1.20]
Li 2001 girls 78 5292 1.47 [1.17; 1.84]
Liu 2024 girls 31 1187 2.61 [1.78; 3.69]
Lu 2010 girls 188 8508 2.21 [1.91; 2.54]
Ma 2015 girls 46 3202 1.44 [1.05; 1.91]
Meng 2003 girls 141 8150 1.73 [1.46; 2.04]
Miu 2017 girls 129 30434 0.42 [0.35; 0.50]
Shen 2019 girls 112 7078 1.58 [1.30; 1.90]
Sun 2009 girls 109 8090 1.35 [1.11; 1.62]
Wang 2013 girls 63 8835 0.71 [0.55; 0.91]
Wen 2021 girls 29 17805 0.16 [0.11; 0.23]
Wu 2021 girls 65 778 8.35 [6.51; 10.53]
Yu 2023 girls 74 809 9.15 [7.25; 11.35]
Yu 2014 girls 140 13845 1.01 [0.85; 1.19]
Zhang 2008 girls 80 9776 0.82 [0.65; 1.02]
Zhang 2011 girls 7 630 1.11 [0.45; 2.28]
Zhang 2023a girls 574 13142 4.37 [4.02; 4.73]
Zhang 2017 girls 769 19916 3.86 [3.60; 4.14]
Zheng 2016 girls 7 5116 0.14 [0.06; 0.28]
Zhou 2023 girls 133 5570 2.39 [2.00; 2.82]
Ma 1995 girls 187 11583 1.61 [1.39; 1.86]
Zou 2022 girls 60 1216 4.93 [3.79; 6.31]
Bondar 2021 girls 1302 458052 0.28 [0.27; 0.30]
Carcamo 2023 girls 30 659 4.55 [3.09; 6.44]
Dohnert 2008 girls 15 143 10.49 [5.99; 16.71]
Etemadifar 2020 girls 13 1513 0.86 [0.46; 1.46]
Jenyo 2005 girls 0 220 0.00 [0.00; 1.66]
Hu 2022 girls 138 5213 2.65 [2.23; 3.12]
Kim 2020 girls 301 7982 3.77 [3.36; 4.21]
Komang-Agung 2017 girls 19 469 4.05 [2.46; 6.25]
Park 2006 girls 47 8317 0.57 [0.42; 0.75]
Ostoji 2006 girls 54 1245 4.34 [3.27; 5.62]
Ravi 2019 girls 34 1565 2.17 [1.51; 3.02]
Sato 2011 girls 37 16231 0.23 [0.16; 0.31]
Stirling 1996 girls 63 7613 0.83 [0.64; 1.06]
Ugras 2010 girls 8 2057 0.39 [0.17; 0.76]
Zhou 2022 girls 257 4780 5.38 [4.75; 6.05]
Yilmaz 2020 girls 251 8162 3.08 [2.71; 3.47]
Aulisa 2019 girls 50 4793 1.04 [0.78; 1.37]
Owada 1982 girls 29 2479 1.17 [0.78; 1.68]
Zhu 2017 girls 263 2072 12.69 [11.29; 14.20]
Cai 2021 girls 216 2479 8.71 [7.63; 9.89]
Chan 1986 girls 54 1727 3.13 [2.36; 4.06]
Guo 2017 girls 77 2579 2.99 [2.36; 3.72]
An 2015 girls 1254 179924 0.70 [0.66; 0.74]
Smyrnis 2015 girls 3 492 0.61 [0.13; 1.77]
Yamamoto 2015a girls 179 38703 0.46 [0.40; 0.54]
Yamamoto 2015b girls 195 28903 0.67 [0.58; 0.78]
zhou 1984 girls 8 2288 0.35 [0.15; 0.69]
zhou 2008 girls 141 14992 0.94 [0.79; 1.11]
cheng 2006 girls 179 11850 1.51 [1.30; 1.75]
Glavas 2023 girls 78 9085 0.86 [0.68; 1.07]
Lee 2014 girls 60 17110 0.35 [0.27; 0.45]
Se-Il 1980 girls 234 3636 6.44 [5.66; 7.28]
Liu 2001 girls 87 3678 2.37 [1.90; 2.91]
Liu 2002 girls 366 40594 0.90 [0.81; 1.00]
Liu 2011 girls 227 12140 1.87 [1.64; 2.13]
Qiu 2022 girls 97 9427 1.03 [0.84; 1.25]
Heine 1981 girls 46 953 4.83 [3.56; 6.39]
Wang 2018b girls 41 12165 0.34 [0.24; 0.46]
Wei 2023 girls 82 8222 1.00 [0.79; 1.24]
Yang 2022a girls 46 10972 0.42 [0.31; 0.56]
Zhang 2003 girls 8 3775 0.21 [0.09; 0.42]
Grivas 2002 girls 63 1533 4.11 [3.17; 5.23]
Mittal 1987 girls 13 12442 0.10 [0.06; 0.18]
Xia 2019 girls 22 1836 1.20 [0.75; 1.81]
Zhao 2014 girls 54 4140 1.30 [0.98; 1.70]
Soucacos 1997 girls 1075 40962 2.62 [2.47; 2.78]
Ke 2015 girls 120 7723 1.55 [1.29; 1.86]
Li 2018 girls 71 7801 0.91 [0.71; 1.15]
Li 1999b girls 53 8213 0.65 [0.48; 0.84]
Souza 2013 girls 11 219 5.02 [2.53; 8.81]
Serbescu 2007 girls 22 139 15.83 [10.19; 22.98]
Suh 2011 girls 26251 550336 4.77 [4.71; 4.83]
Wong 2005 girls 229 37141 0.62 [0.54; 0.70]
Morais 1985 girls 348 14689 2.37 [2.13; 2.63]
Chen 2023 girls 38 8892 0.43 [0.30; 0.59]
Gao 2023 girls 885 40618 2.18 [2.04; 2.33]
Willner 1982b girls 272 8469 3.21 [2.85; 3.61]
Chen 2016 girls 214 13081 1.64 [1.43; 1.87]
Chen 2012 girls 70 8985 0.78 [0.61; 0.98]
Dong 2009 girls 41 4675 0.88 [0.63; 1.19]
Ueno 2011 girls 2048 127903 1.60 [1.53; 1.67]
Dantas 2021 girls 10 299 3.34 [1.62; 6.06]
Penha 2018 girls 32 1490 2.15 [1.47; 3.02]
sung 2021 girls 85747 17817159 0.48 [0.48; 0.48]
Li 2017b girls 21 7095 0.30 [0.18; 0.45]
pin 1985 girls 97 3963 2.45 [1.99; 2.98]
Du 2016 girls 104 16108 0.65 [0.53; 0.78]
Liang 2005 girls 33 4051 0.81 [0.56; 1.14]
Wang 1998 girls 16 4535 0.35 [0.20; 0.57]
Sugita 2000 girls 84 1702 4.94 [3.96; 6.07]
Koukourakis 1997 girls 181 10278 1.76 [1.52; 2.03]
Donald 1981 girls 155 7642 2.03 [1.72; 2.37]
Kunakornsawat 2017 girls 84 1818 4.62 [3.70; 5.69]
Yong 2009 girls 1119 93626 1.20 [1.13; 1.27]
Abo-Bakr 1992 girls 29 1147 2.53 [1.70; 3.61]
Goldberg 1995 girls 33 8686 0.38 [0.26; 0.53]
Random effects model 1.76 [1.42; 2.13]
Heterogeneity: I^2 = 99.8%, p = 0
Random effects model 1.29 [1.10; 1.50]
Heterogeneity: I^2 = 99.8%, p = 0
Test for subgroup differences: χ^2_1 = 21.14, df = 1 (p < 0.01)
0 5 10 15

S14.3 Age group.

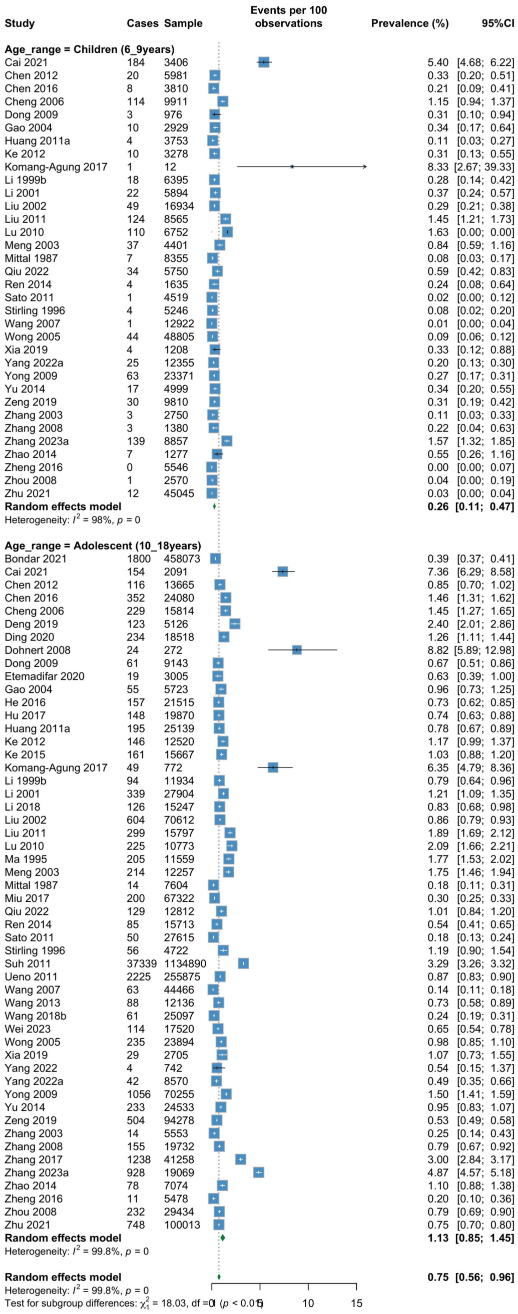

S14.4 WHO region.

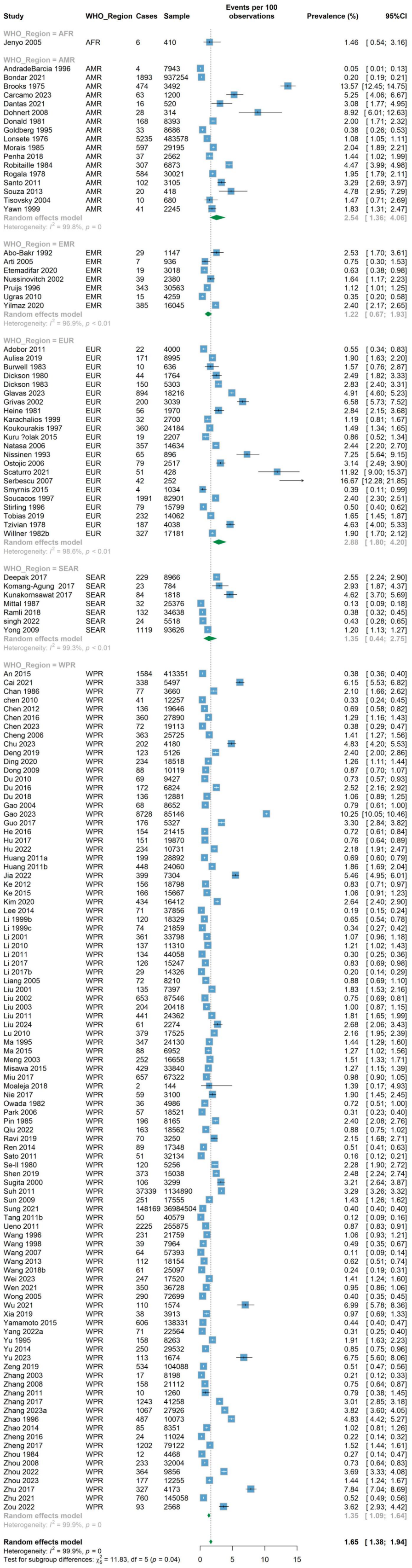

S14.5 World Bank region.

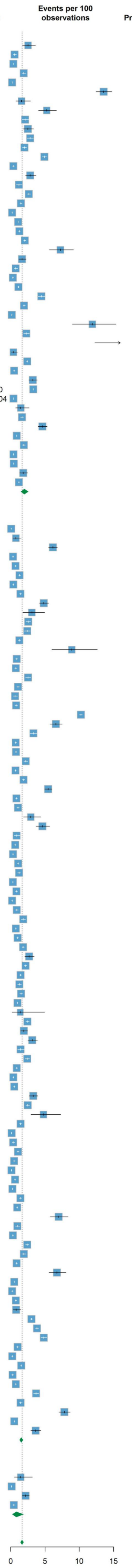

Study	WB_Region	Cases	Sample	Prevalence (%)	95%CI
**WB_Region = HIC**					
Abo-Bakr 1992	HIC	29	1147	2.53	[1.70; 3.61]
Adobor 2011	HIC	22	4000	0.55	[0.34; 0.83]
An 2015	HIC	1584	413351	0.38	[0.36; 0.40]
Aulisa 2019	HIC	171	8995	1.90	[1.63; 2.20]
Bondar 2021	HIC	1893	937254	0.20	[0.19; 0.21]
Brooks 1975	HIC	474	3492	13.57	[12.45; 14.75]
Burwell 1983	HIC	10	636	1.57	[0.76; 2.87]
Carcamo 2023	HIC	63	1200	5.25	[4.06; 6.67]
Chan 1986	HIC	77	3660	2.10	[1.66; 2.62]
Dickson 1980	HIC	44	1764	2.49	[1.82; 3.33]
Dickson 1983	HIC	150	5303	2.83	[2.40; 3.31]
Donald 1981	HIC	168	8393	2.00	[1.71; 2.32]
Glavas 2023	HIC	894	18216	4.91	[4.60; 5.23]
Goldberg 1995	HIC	33	8686	0.38	[0.26; 0.53]
Heine 1981	HIC	56	1970	2.84	[2.15; 3.68]
Karachalios 1999	HIC	32	2700	1.19	[0.81; 1.67]
Kim 2020	HIC	434	16412	2.64	[2.40; 2.90]
Koukourakis 1997	HIC	360	24184	1.49	[1.34; 1.65]
Lee 2014	HIC	71	37856	0.19	[0.15; 0.24]
Lonsete 1976	HIC	5235	483578	1.08	[1.05; 1.11]
Misawa 2015	HIC	429	33840	1.27	[1.15; 1.39]
Morais 1985	HIC	597	29195	2.04	[1.89; 2.21]
Nissinen 1993	HIC	65	896	7.25	[5.64; 9.15]
Nussinovitch 2002	HIC	39	2380	1.64	[1.17; 2.23]
Owada 1982	HIC	36	4986	0.72	[0.51; 1.00]
Park 2006	HIC	57	18521	0.31	[0.23; 0.40]
Pruijs 1996	HIC	343	30563	1.12	[1.01; 1.25]
Robitaille 1984	HIC	307	6873	4.47	[3.99; 4.98]
Rogala 1978	HIC	584	30021	1.95	[1.79; 2.11]
Sato 2011	HIC	51	32134	0.16	[0.12; 0.21]
Scaturro 2021	HIC	51	428	11.92	[9.00; 15.37]
Se-Il 1980	HIC	120	5256	2.28	[1.90; 2.72]
Serbescu 2007	HIC	42	252	16.67	[12.28; 21.85]
Smyrnis 2015	HIC	4	1034	0.39	[0.11; 0.99]
Soucacos 1997	HIC	1991	82901	2.40	[2.30; 2.51]
Stirling 1996	HIC	79	15799	0.50	[0.40; 0.62]
Sugita 2000	HIC	106	3299	3.21	[2.64; 3.87]
Suh 2011	HIC	37339	1134890	3.29	[3.26; 3.32]
Sung 2021	HIC	148169	36984504	0.40	[0.40; 0.40]
Tisovsky 2004	HIC	10	680	1.47	[0.71; 2.69]
Tobias 2019	HIC	232	14062	1.65	[1.45; 1.87]
Tzivian 1978	HIC	187	4038	4.63	[4.00; 5.33]
Ueno 2011	HIC	2225	255875	0.87	[0.83; 0.91]
Willner 1982b	HIC	327	17181	1.90	[1.70; 2.12]
Wong 2005	HIC	290	72699	0.40	[0.35; 0.45]
Yamamoto 2015	HIC	606	138331	0.44	[0.40; 0.47]
Yawn 1999	HIC	41	2245	1.83	[1.31; 2.47]
Yong 2009	HIC	1119	93626	1.20	[1.13; 1.27]
**Random effects model**				**1.97**	**[1.39; 2.65]**
Heterogeneity: I^2 = 99.9%, p = 0					
**WB_Region = UMIC**					
AndradeBarcia 1996	UMIC	4	7943	0.05	[0.01; 0.13]
Arti 2005	UMIC	7	936	0.75	[0.30; 1.53]
Cai 2021	UMIC	338	5497	6.15	[5.53; 6.82]
Chen 2010	UMIC	41	12257	0.33	[0.24; 0.45]
Chen 2012	UMIC	136	19646	0.69	[0.58; 0.82]
Chen 2016	UMIC	360	27890	1.29	[1.16; 1.43]
Chen 2023	UMIC	72	19113	0.38	[0.29; 0.47]
Cheng 2006	UMIC	363	25725	1.41	[1.27; 1.56]
Chu 2023	UMIC	202	4180	4.83	[4.20; 5.53]
Dantas 2021	UMIC	16	520	3.08	[1.77; 4.95]
Deepak 2017	UMIC	229	8966	2.55	[2.24; 2.90]
Deng 2019	UMIC	123	5126	2.40	[2.00; 2.86]
Ding 2020	UMIC	234	18518	1.26	[1.11; 1.44]
Dohnert 2008	UMIC	28	314	8.92	[6.01; 12.63]
Dong 2009	UMIC	88	10119	0.87	[0.70; 1.07]
Du 2010	UMIC	69	9427	0.73	[0.57; 0.93]
Du 2016	UMIC	172	6824	2.52	[2.16; 2.92]
Du 2018	UMIC	136	12881	1.06	[0.89; 1.25]
Etemadifar 2020	UMIC	19	3018	0.63	[0.38; 0.98]
Gao 2004	UMIC	68	8652	0.79	[0.61; 1.00]
Gao 2023	UMIC	8728	85146	10.25	[10.05; 10.46]
Grivas 2002	UMIC	200	3039	6.58	[5.73; 7.52]
Guo 2017	UMIC	176	5327	3.30	[2.84; 3.82]
He 2016	UMIC	154	21415	0.72	[0.61; 0.84]
Hu 2017	UMIC	151	19870	0.76	[0.64; 0.89]
Hu 2022	UMIC	234	10731	2.18	[1.91; 2.47]
Huang 2011a	UMIC	199	28892	0.69	[0.60; 0.79]
Huang 2011b	UMIC	448	24060	1.86	[1.69; 2.04]
Jia 2022	UMIC	399	7304	5.46	[4.95; 6.01]
Ke 2012	UMIC	156	18798	0.83	[0.71; 0.97]
Ke 2015	UMIC	166	15667	1.06	[0.91; 1.23]
Komang-Agung 2017	UMIC	23	784	2.93	[1.87; 4.37]
Kunakornsawat 2017	UMIC	84	1818	4.62	[3.70; 5.69]
Kuru Colak 2015	UMIC	19	2207	0.86	[0.52; 1.34]
Li 1999b	UMIC	120	18329	0.65	[0.54; 0.78]
Li 1999c	UMIC	74	21859	0.34	[0.27; 0.42]
Li 2001	UMIC	361	33798	1.07	[0.96; 1.18]
Li 2010	UMIC	137	11310	1.21	[1.02; 1.43]
Li 2011	UMIC	134	44058	0.30	[0.25; 0.36]
Li 2017	UMIC	126	15247	0.83	[0.69; 0.98]
Li 2017b	UMIC	29	14326	0.20	[0.14; 0.29]
Liang 2005	UMIC	72	8210	0.88	[0.69; 1.10]
Liu 2001	UMIC	135	7397	1.83	[1.53; 2.16]
Liu 2002	UMIC	653	87546	0.75	[0.69; 0.81]
Liu 2003	UMIC	204	20418	1.00	[0.87; 1.15]
Liu 2011	UMIC	441	24362	1.81	[1.65; 1.99]
Liu 2024	UMIC	61	2274	2.68	[2.06; 3.43]
Lu 2010	UMIC	379	17525	2.16	[1.95; 2.39]
Ma 1995	UMIC	347	24130	1.44	[1.29; 1.60]
Ma 2015	UMIC	88	6952	1.27	[1.02; 1.56]
Meng 2003	UMIC	252	16658	1.51	[1.33; 1.71]
Miu 2017	UMIC	657	67322	0.98	[0.90; 1.05]
Moaleja 2018	UMIC	2	144	1.39	[0.17; 4.93]
Natasa 2006	UMIC	357	14634	2.44	[2.20; 2.70]
Nie 2017	UMIC	59	3100	1.90	[1.45; 2.45]
Ostojic 2006	UMIC	79	2517	3.14	[2.49; 3.90]
Penha 2018	UMIC	37	2562	1.44	[1.02; 1.99]
Pin 1985	UMIC	196	8165	2.40	[2.08; 2.76]
Qiu 2022	UMIC	163	18562	0.88	[0.75; 1.02]
Ramli 2018	UMIC	132	34638	0.38	[0.32; 0.45]
Ren 2014	UMIC	89	17348	0.51	[0.41; 0.63]
Santo 2011	UMIC	102	3105	3.29	[2.69; 3.97]
Shen 2019	UMIC	373	15038	2.48	[2.24; 2.74]
Souza 2013	UMIC	20	418	4.78	[2.95; 7.29]
Sun 2009	UMIC	251	17555	1.43	[1.26; 1.62]
Tang 2011b	UMIC	50	40579	0.12	[0.09; 0.16]
Ugras 2010	UMIC	15	4259	0.35	[0.20; 0.58]
Wang 1996	UMIC	231	21759	1.06	[0.93; 1.21]
Wang 1998	UMIC	39	7964	0.49	[0.35; 0.67]
Wang 2007	UMIC	64	57393	0.11	[0.09; 0.14]
Wang 2013	UMIC	112	18154	0.62	[0.51; 0.74]
Wang 2018b	UMIC	61	25097	0.24	[0.19; 0.31]
Wei 2023	UMIC	247	17520	1.41	[1.24; 1.60]
Wen 2021	UMIC	350	36728	0.95	[0.86; 1.06]
Wu 2021	UMIC	110	1574	6.99	[5.78; 8.36]
Xia 2019	UMIC	38	3913	0.97	[0.69; 1.33]
Yang 2022a	UMIC	71	22564	0.31	[0.25; 0.40]
Yilmaz 2020	UMIC	385	16045	2.40	[2.17; 2.65]
Yu 1995	UMIC	158	8263	1.91	[1.63; 2.23]
Yu 2014	UMIC	250	29532	0.85	[0.75; 0.96]
Yu 2023	UMIC	113	1674	6.75	[5.60; 8.06]
Zeng 2019	UMIC	534	104088	0.51	[0.47; 0.56]
Zhang 2003	UMIC	17	8198	0.21	[0.12; 0.33]
Zhang 2008	UMIC	158	21112	0.75	[0.64; 0.87]
Zhang 2011	UMIC	10	1260	0.79	[0.38; 1.45]
Zhang 2017	UMIC	1243	41258	3.01	[2.85; 3.18]
Zhang 2023a	UMIC	1067	27926	3.82	[3.60; 4.05]
Zhao 1996	UMIC	487	10073	4.83	[4.42; 5.27]
Zhao 2014	UMIC	85	8351	1.02	[0.81; 1.26]
Zheng 2016	UMIC	24	11024	0.22	[0.14; 0.32]
Zheng 2017	UMIC	1202	79122	1.52	[1.44; 1.61]
Zhou 1984	UMIC	12	4468	0.27	[0.14; 0.47]
Zhou 2008	UMIC	233	32004	0.73	[0.64; 0.83]
Zhou 2022	UMIC	364	9856	3.69	[3.33; 4.08]
Zhou 2023	UMIC	177	12255	1.44	[1.24; 1.67]
Zhu 2017	UMIC	327	4173	7.84	[7.04; 8.69]
Zhu 2021	UMIC	760	145058	0.52	[0.49; 0.56]
Zou 2022	UMIC	93	2568	3.62	[2.93; 4.42]
**Random effects model**				**1.54**	**[1.24; 1.86]**
Heterogeneity: I^2 = 99.7%, p = 0					
**WB_Region = LMIC**					
Jenyo 2005	LMIC	6	410	1.46	[0.54; 3.16]
Mittal 1987	LMIC	32	25376	0.13	[0.09; 0.18]
Ravi 2019	LMIC	70	3250	2.15	[1.68; 2.71]
Singh 2022	LMIC	24	5518	0.43	[0.28; 0.65]
**Random effects model**				**0.81**	**[0.13; 2.03]**
Heterogeneity: I^2 = 98.1%, p < 0.01					
**Random effects model**				**1.65**	**[1.38; 1.94]**
Heterogeneity: I^2 = 99.9%, p = 0					
Test for subgroup differences: χ^2_2 = 3.42, df = 2 (p = 0.18)					

S14.6 Latitude.

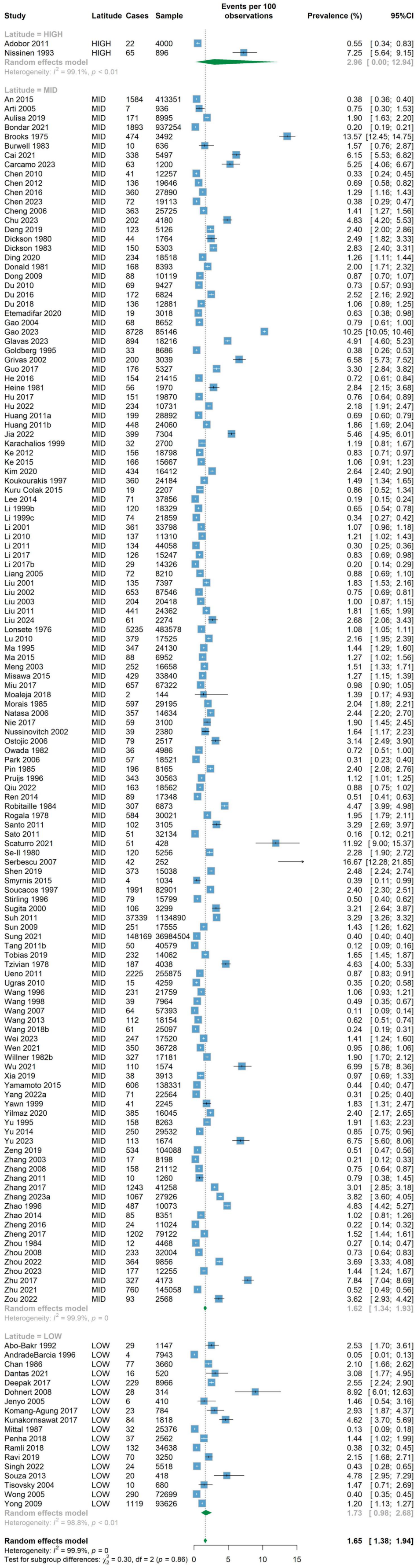

S14.7 BMI.

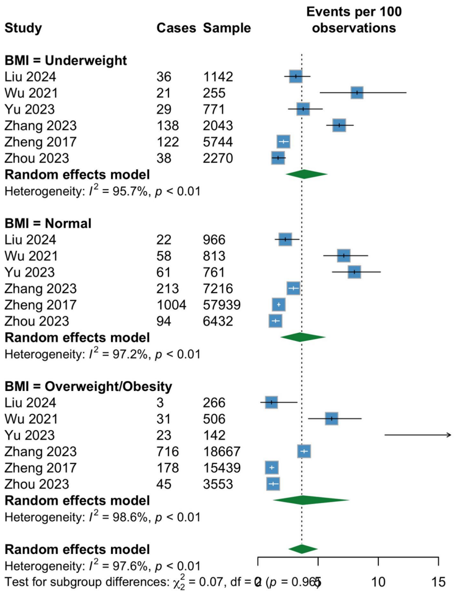

S14.8 Family history.

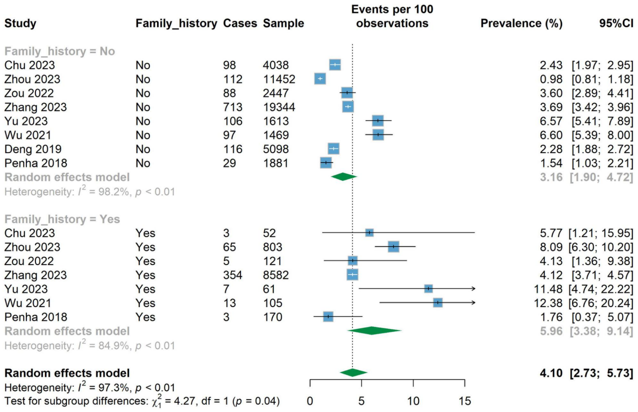

S14.9 Daily physical exercise.

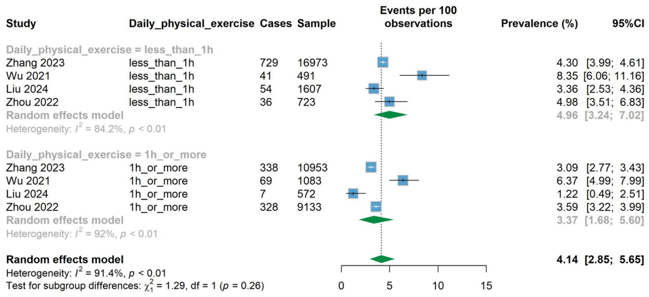

S14.10 Daily sleep duration.

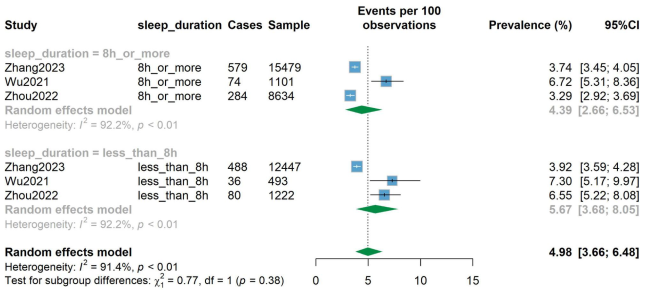

S14.11 Daily screen time.

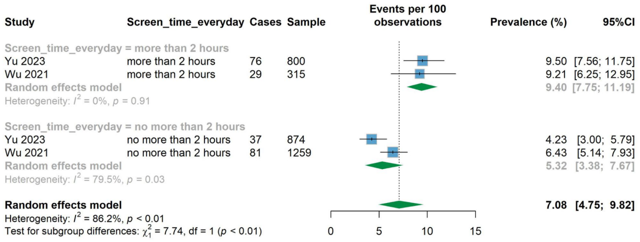

S14.12 Sitting posture.

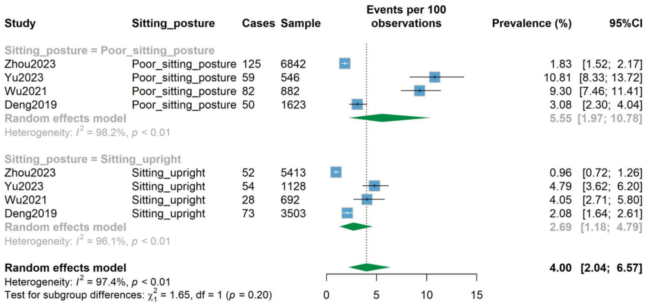

S14.13 Desk height.

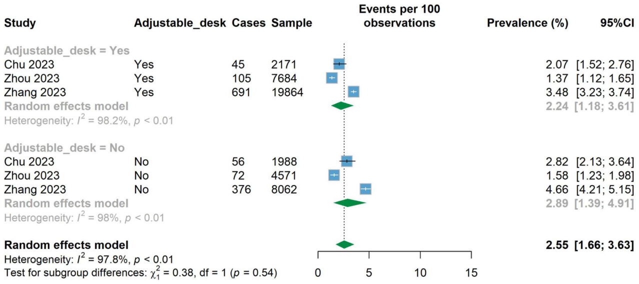

S14.14 Types of physical exercise.

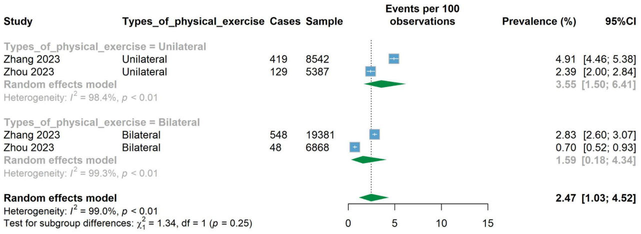

Appendix S15: **Included studies in meta-analyses of associated factors of scoliosis in children and adolescents.**

Risk factor	Author	Year Published	Country	WHO region	WB region	Forest plot
**Factor 1-Sexes (girls)**						
Chu 2023[1]	Chu, et al.	2023	China	WPR	UMIC	
Deng 2019[3]	Deng, et al.	2019	China	WPR	UMIC	
Wu 2021[24]	Wu, et al.	2021	China	WPR	UMIC	
Yu 2023[26]	Yu, et al.	2023	China	WPR	UMIC	
Zhang 2023a[30]	Zhang, et al.	2023	China	WPR	UMIC	
Zhou 2023[33]	Zhou, et al.	2023	China	WPR	UMIC	
Zou 2022[36]	Zou, et al.	2022	China	WPR	UMIC	
Bondar 2021[37]	Bondar, et al.	2021	America	AMR	HIC	
Hu 2022[47]	Hu, et al.	2022	China	WPR	UMIC	
Yan 2020[64]	Yan, et al.	2020	China	WPR	UMIC	
Zhou 2022[65]	Zhou, et al.	2022	China	WPR	UMIC	
Zheng 2016[32]	Zheng, et al.	2016	China	WPR	UMIC	
Cai 2021[38]	Cai, et al.	2021	China	WPR	UMIC	
Santo 2011[116]	Santo, et al.	2011	Brazil	AMR	UMIC	

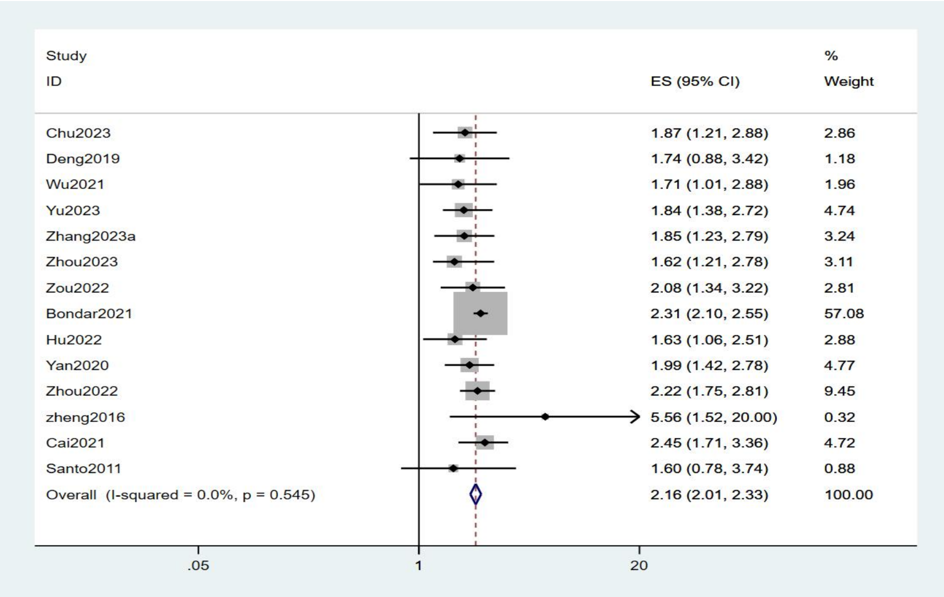

Risk factor	Author	Year Published	Country	WHO region	WB region	Forest plot
**Factor 2-Older age**						
Zhang 2023a[30]	Zhang, et al.	2023	China	WPR	UMIC	
Bondar 2021[37]	Bondar, et al.	2021	America	AMR	HIC	
Zou 2022[36]	Zou, et al.	2022	China	WPR	UMIC	
Yan 2020[64]	Yan, et al.	2020	China	WPR	UMIC	
Zhou 2022[65]	Zhou, et al.	2022	China	WPR	UMIC	
Yong 2009[67]	Yong, et al.	2009	Singapore	WPR	HIC	
Zheng 2016[32]	Zheng, et al.	2016	China	WPR	UMIC	
Yang 2022b[44]	Yang, et al.	2022	China	WPR	UMIC	
Santo 2011[116]	Santo, et al.	2011	Brazil	AMR	UMIC	

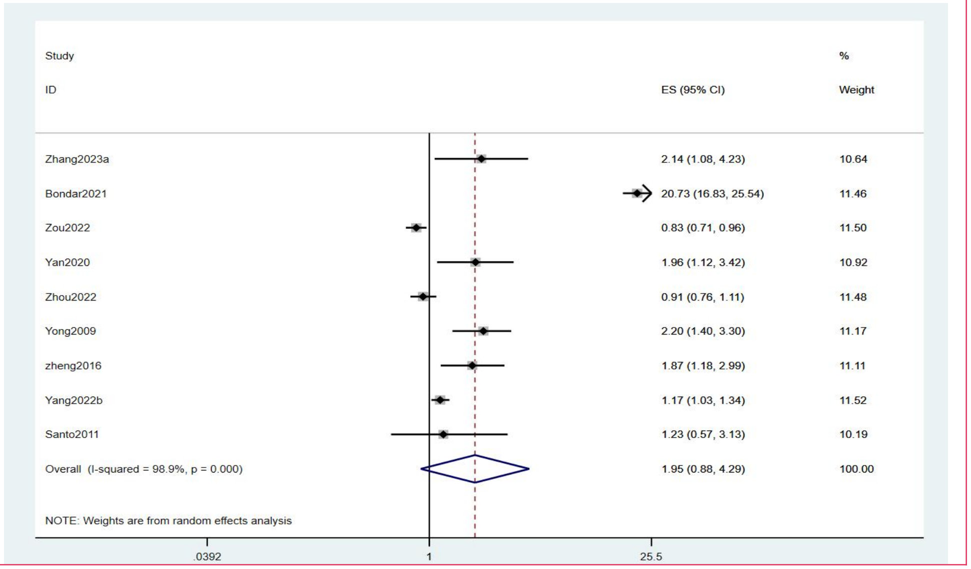

**Factor 3-Low BMI**					
Chu 2023[1]	Chu, et al.	2023	China	WPR	UMIC
Zhou 2022[65]	Zhou, et al.	2022	China	WPR	UMIC
Cai 2021[38]	Cai, et al.	2021	China	WPR	UMIC
Zheng 2016[32]	Zheng, et al.	2016	China	WPR	UMIC
Yu 2023[26]	Yu, et al.	2023	China	WPR	UMIC
Zhang 2023a[30]	Zhang, et al.	2023	China	WPR	UMIC

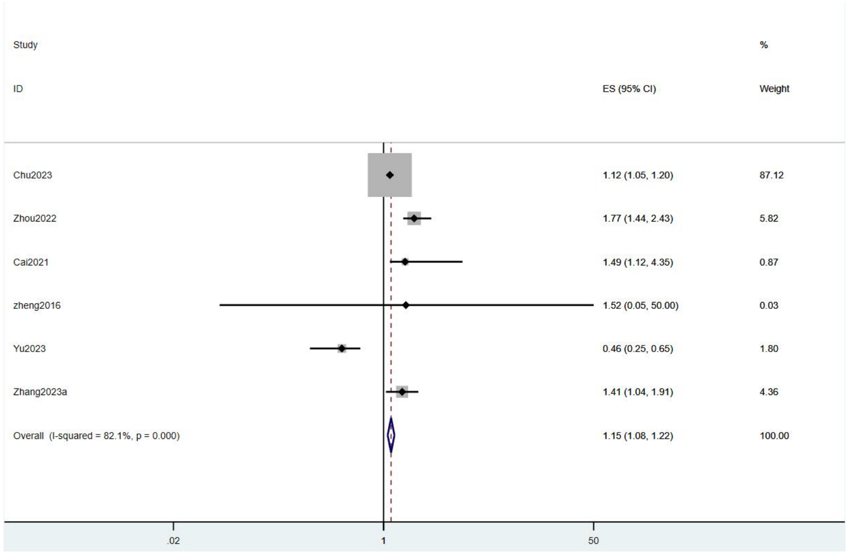

**Factor 4-Myopia**					
Zhou 2022[65]	Zhou, et al.	2022	China	WPR	UMIC
Cai 2021[38]	Cai, et al.	2021	China	WPR	UMIC

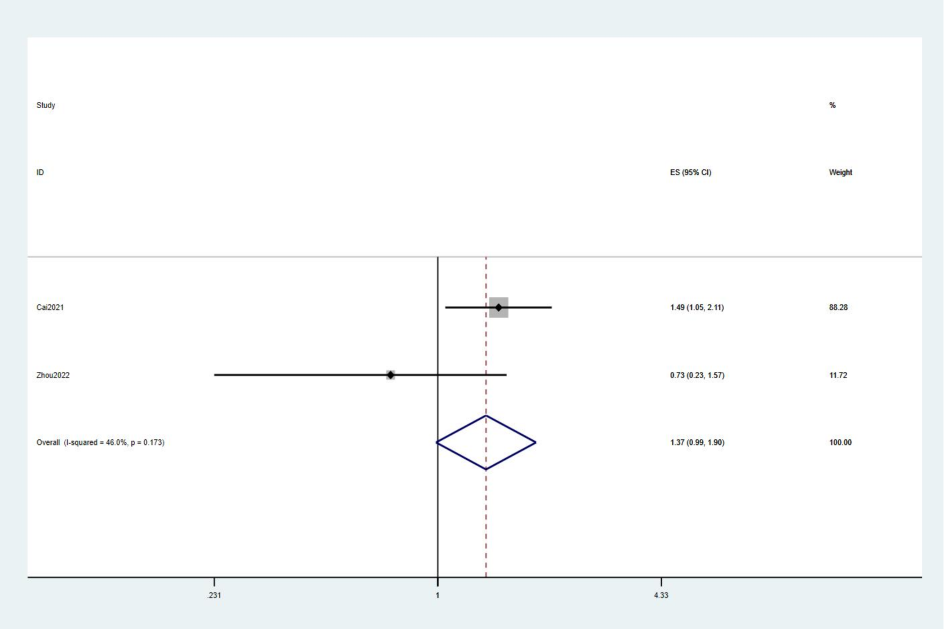

Factor 5-Short outdoor time (<1 hour)					
Liu 2024[14]	Liu, et al.	2024	China	WPR	UMIC
Wu 2021[24]	Wu, et al.	2021	China	WPR	UMIC
Zhang 2023a[30]	Zhang, et al.	2023	China	WPR	UMIC
Zhou 2022[65]	Zhou, et al.	2022	China	WPR	UMIC
Yang 2022b[44]	Yang, et al.	2022	China	WPR	UMIC
Kim 2020[50]	Kim, et al.	2020	Korea	WPR	HIC
Zhu 2023[69]	Zhu, et al.	2023	China	WPR	UMIC
Zheng 2016[32]	Zheng, et al.	2016	China	WPR	UMIC
Cai 2021[38]	Cai, et al.	2021	China	WPR	UMIC

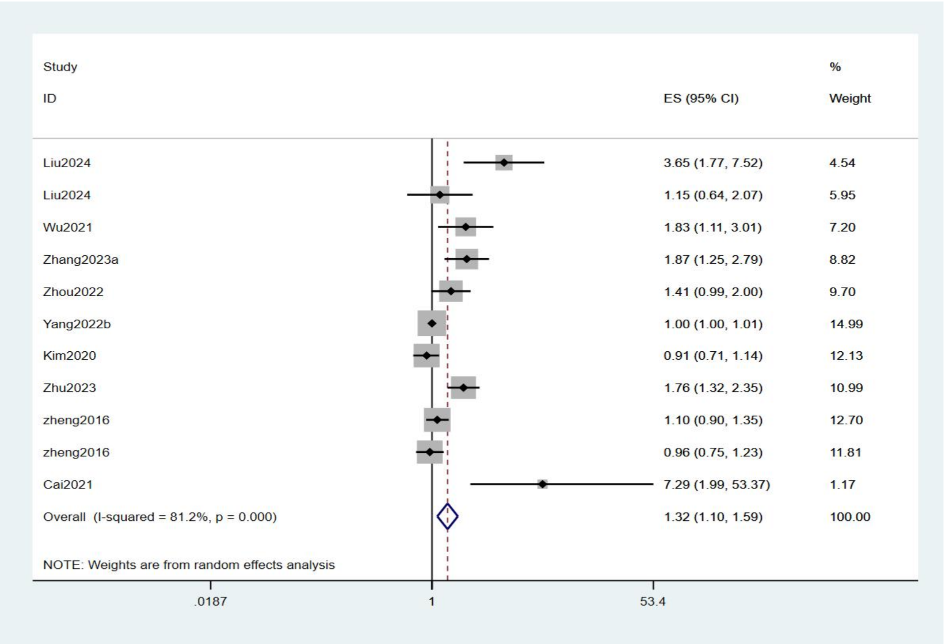

Factor 6-Long screen time (≥2 hours)					
Wu 2021[24]	Wu, et al.	2021	China	WPR	UMIC
Yu 2023[26]	Yu, et al.	2023	China	WPR	UMIC
Liu 2024[14]	Liu, et al.	2024	China	WPR	UMIC
Zhu 2023[69]	Zhu, et al.	2023	China	WPR	UMIC

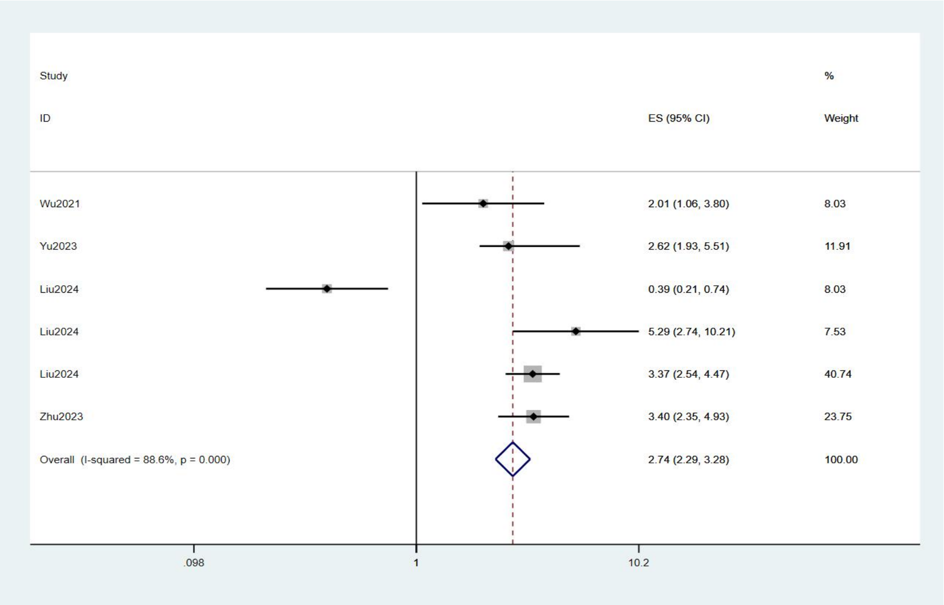

**Factor 7-Sedentary behavior (≥11 hours)**					
Zhang 2023a[30]	Zhang, et al.	2023	China	WPR	UMIC
Wu 2021[24]	Wu, et al.	2021	China	WPR	UMIC
Yu 2023[26]	Yu, et al.	2023	China	WPR	UMIC
Zhou 2023[33]	Zhou, et al.	2023	China	WPR	UMIC
Hu 2022[47]	Hu, et al.	2022	China	WPR	UMIC

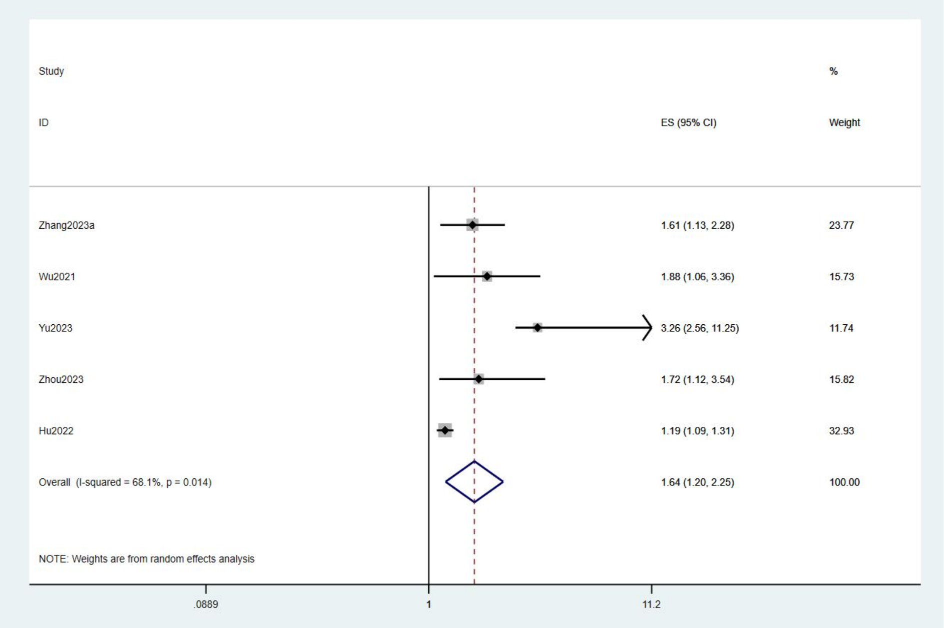

**Factor 8-Poor sitting posture**					
Liu 2024[14]	Liu, et al.	2024	China	WPR	UMIC
Wu 2021[24]	Wu, et al.	2021	China	WPR	UMIC
Yu 2023[26]	Yu, et al.	2023	China	WPR	UMIC
Deng 2019[3]	Deng, et al.	2019	China	WPR	UMIC
Yang 2022b[44]	Yang, et al.	2022	China	WPR	UMIC

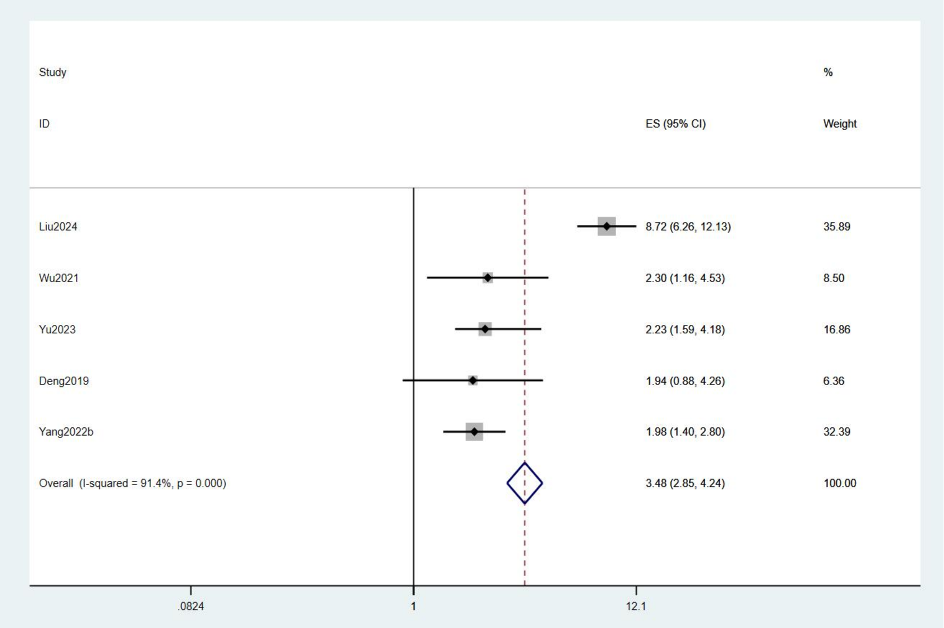

**Factor 9-Unilateral physical activity**						
Zhang 2023a[30]	Zhang, et al.	2023	China	WPR	UMIC	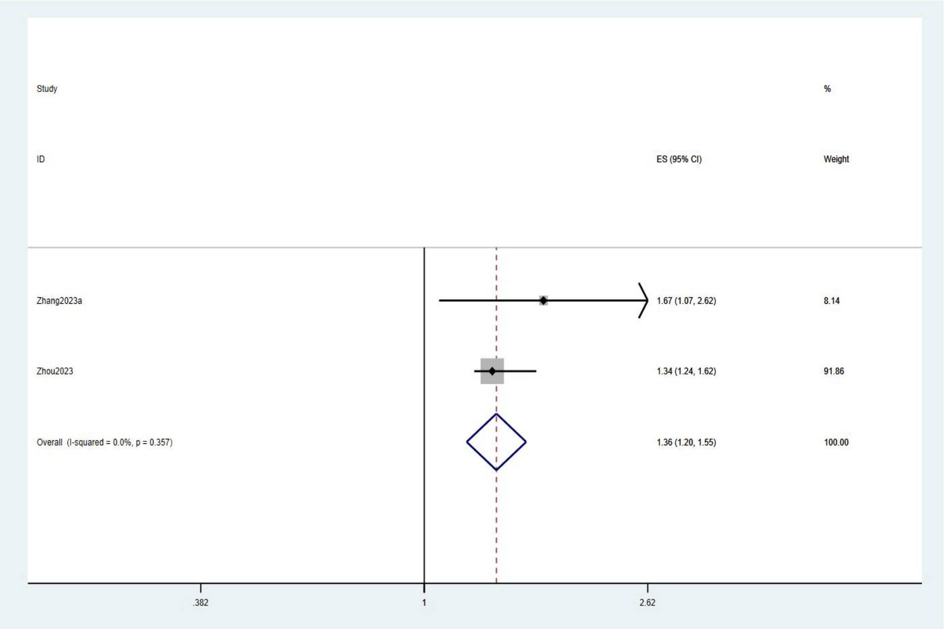
Zhou 2023[33]	Zhou, et al.	2023	China	WPR	UMIC	
**Factor 10-Single-shoulder schoolbag**						
Zhang 2023a[30]	Zhang, et al.	2023	China	WPR	UMIC	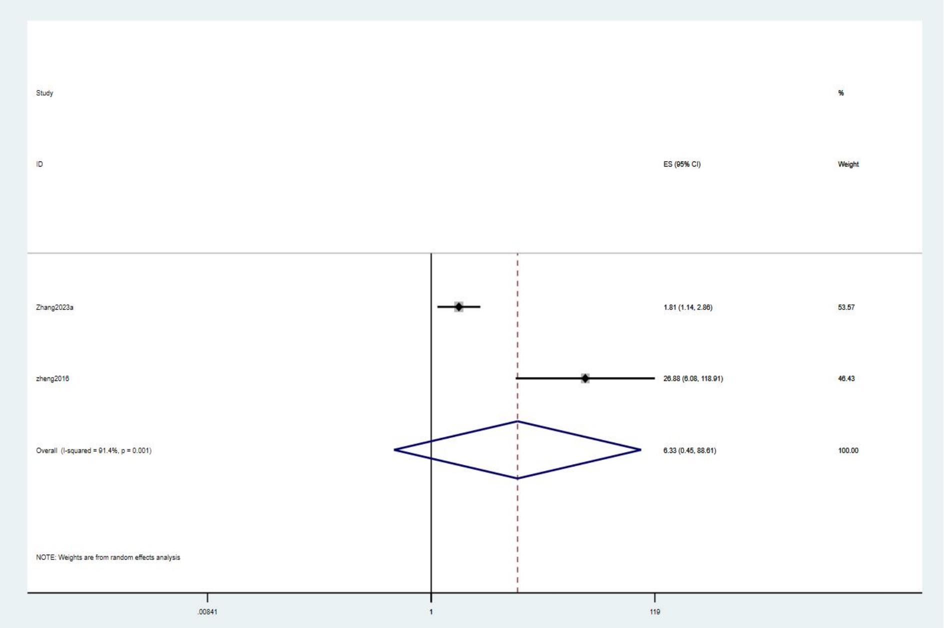
Zheng 2016[32]	Zheng, et al.	2016	China	WPR	UMIC	

**Factor 11-Heavy bag**					
Zhang 2023a[30]	Zhang, et al.	2023	China	WPR	UMIC
Zheng 2016[32]	Zheng, et al.	2016	China	WPR	UMIC

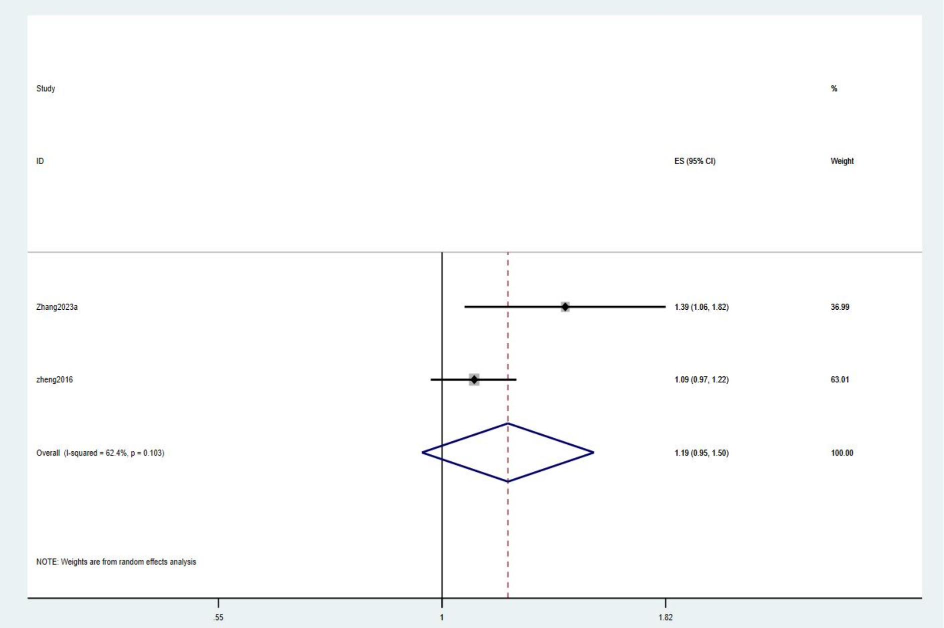

**Factor 12-Positive family history**					
Deng 2019[3]	Deng, et al.	2019	China	WPR	UMIC
Wu 2021[24]	Wu, et al.	2021	China	WPR	UMIC
Zhang 2023a[30]	Zhang, et al.	2023	China	WPR	UMIC
Zhou 2023[33]	Zhou, et al.	2023	China	WPR	UMIC

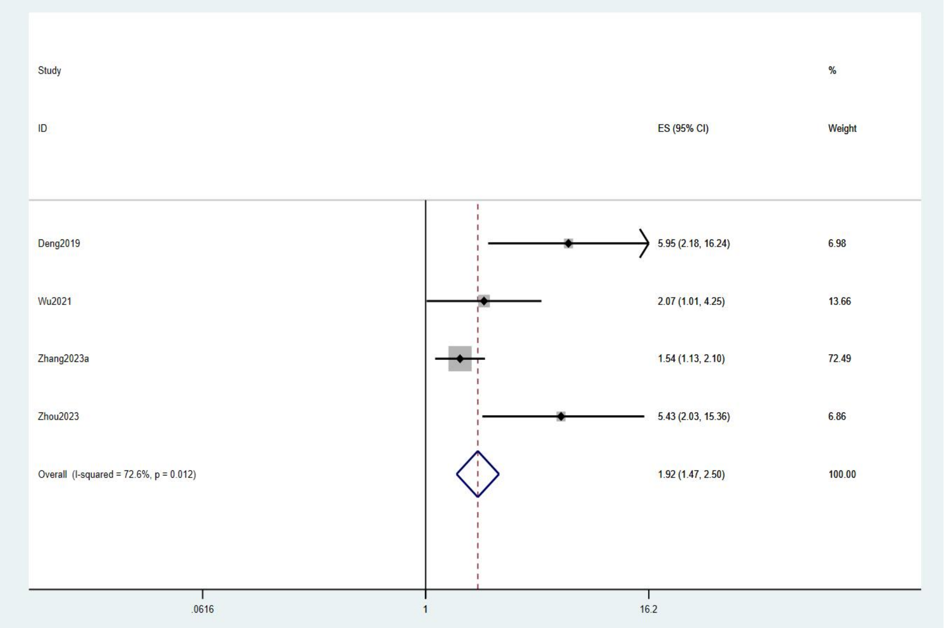

Factor 13-Rural residence					
Zhou 2022[65]	Zhou, et al.	2022	China	WPR	UMIC
Deng 2019[3]	Deng, et al.	2019	China	WPR	UMIC
Ren 2014[20]	Ren, et al.	2014	China	WPR	UMIC

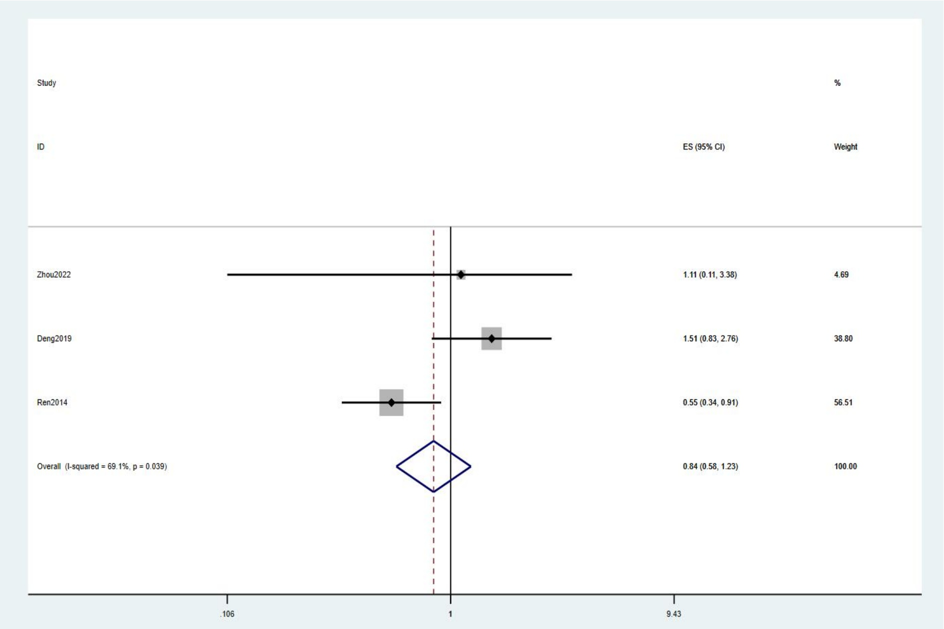

Factor 14-Low annual household income					
Zheng 2016[32]	Zheng, et al.	2016	China	WPR	UMIC
Zhou 2022[65]	Zhou, et al.	2022	China	WPR	UMIC
Santo 2011[116]	Santo, et al.	2011	Brazil	AMR	UMIC

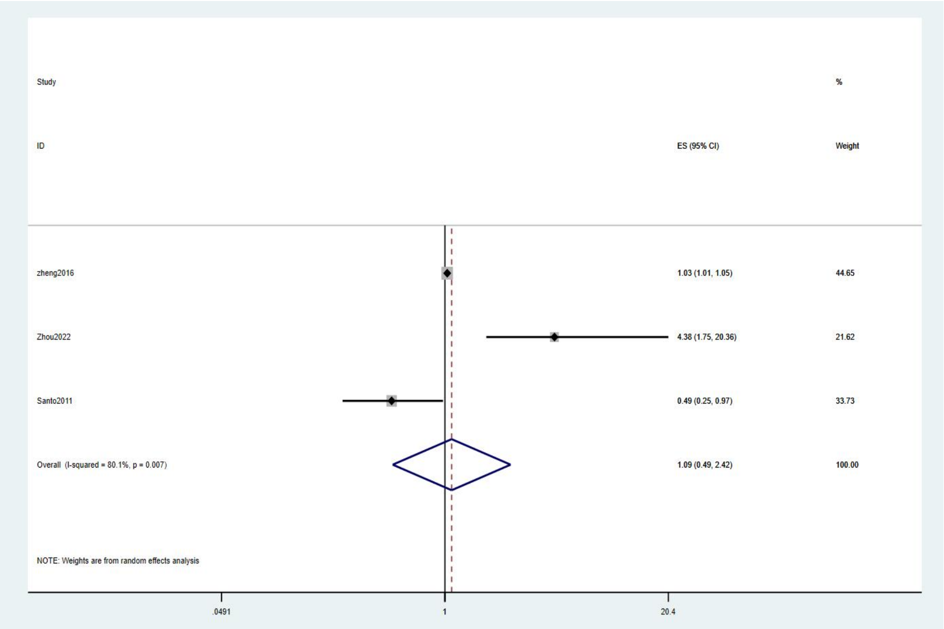

Note: WHO, World Health Organization; AFR, African Region; AMR, Region of the Americas; SEAR, South-East Asia Region; EUR, European Region; EMR, Eastern Mediterranean Region; WPR, Western Pacific Region; WB, World Bank; HIC, high-income countries; LMIC, low- and middle-income countries; UMIC, upper middle-income countries.

Appendix S16: **Sensitivity analyses.**

S16.1 Influence Diagnostics.

ID	rstudent	dffits	cook.d	cov.r	QE.del	hat	weight	infl
Abo-Bakr 1992[190]	0.454	0.036	0.001	1.012	122279.51	0.006	0.647	
Adobor 2011[229]	-0.793	-0.066	0.004	1.01	122317.45	0.007	0.669	
An 2015[49]	-0.992	-0.082	0.007	1.007	122223.21	0.007	0.678	
AndradeBarcia 1996[98]	-1.557	-0.127	0.016	0.997	122251.51	0.007	0.673	
Arti 2005[71]	-0.569	-0.046	0.002	1.011	122316.44	0.006	0.641	
Aulisa 2019[70]	0.134	0.01	0	1.014	122146.46	0.007	0.674	
Bondar 2021[37]	-1.245	-0.102	0.01	1.003	120012.9	0.007	0.678	
Brooks 1975[140]	3.795	0.327	0.098	0.919	120992.73	0.007	0.667	*
Burwell 1983[218]	-0.009	-0.002	0	1.013	122309.02	0.006	0.625	
Cai 2021[38]	1.793	0.15	0.022	0.991	121595.35	0.007	0.671	
Carcamo 2023[39]	1.484	0.121	0.015	0.998	122191.09	0.006	0.649	
Chan 1986[40]	0.242	0.019	0	1.013	122232.19	0.007	0.668	
Chen 2010[193]	-1.046	-0.086	0.007	1.006	122311.72	0.007	0.675	
Chen 2012[172]	-0.674	-0.056	0.003	1.011	122302.62	0.007	0.676	
Chen 2016[171]	-0.226	-0.02	0	1.014	122097.38	0.007	0.676	
Chen 2023[173]	-0.996	-0.082	0.007	1.007	122313.22	0.007	0.676	
Cheng 2006[77]	-0.15	-0.013	0	1.014	122063.99	0.007	0.676	
Chu 2023[1]	1.358	0.112	0.013	1.001	121929.5	0.007	0.669	
Dantas 2021[202]	0.693	0.054	0.003	1.009	122293.02	0.006	0.614	
Deepak 2017[85]	0.458	0.037	0.001	1.012	122019.67	0.007	0.674	
Deng 2019[3]	0.386	0.031	0.001	1.013	122165.04	0.007	0.671	
Dickson 1980[86]	0.435	0.035	0.001	1.012	122260.81	0.007	0.658	
Dickson 1983[219]	0.583	0.047	0.002	1.011	122106.76	0.007	0.671	
Ding 2020[4]	-0.243	-0.021	0	1.014	122179.12	0.007	0.676	
Dohnert 2008[42]	2.426	0.189	0.035	0.976	122247.86	0.006	0.579	
Donald 1981[231]	0.187	0.014	0	1.014	122140.59	0.007	0.673	
Dong 2009[178]	-0.523	-0.044	0.002	1.012	122294.3	0.007	0.674	
Du 2010[204]	-0.636	-0.053	0.003	1.011	122307.7	0.007	0.674	
Du 2016[212]	0.443	0.036	0.001	1.012	122095.95	0.007	0.672	
Du 2018[179]	-0.385	-0.033	0.001	1.013	122259.64	0.007	0.675	
Etemadifar 2020[45]	-0.713	-0.059	0.003	1.01	122316.49	0.007	0.666	
Gao 2004[5]	-0.59	-0.049	0.002	1.012	122304.64	0.007	0.674	
Gao 2023[181]	2.976	0.255	0.062	0.953	99878.36	0.007	0.677	*
Glavas 2023[88]	1.388	0.116	0.013	1	120590.03	0.007	0.676	
Goldberg 1995[228]	-0.987	-0.081	0.007	1.007	122316.07	0.007	0.674	
Grivas 2002[147]	1.921	0.16	0.025	0.988	121877.91	0.007	0.666	
Guo 2017[43]	0.785	0.064	0.004	1.009	122043.19	0.007	0.671	
He 2016[6]	-0.651	-0.054	0.003	1.011	122297.21	0.007	0.676	
Heine 1981[113]	0.591	0.047	0.002	1.011	122238.11	0.007	0.66	
Hu 2017[7]	-0.615	-0.051	0.003	1.011	122292.35	0.007	0.676	
Hu 2022[47]	0.278	0.022	0	1.013	122050.81	0.007	0.674	
Huang 2011a[8]	-0.678	-0.057	0.003	1.011	122296.22	0.007	0.677	

Huang 2011b[154]	0.111	0.008	0	1.014	121879.58	0.007	0.676
Jenyo 2005[46]	-0.047	-0.005	0	1.012	122312.7	0.006	0.599
Jia 2022[9]	1.573	0.131	0.017	0.996	121507.28	0.007	0.673
Karachalios 1999[48]	-0.283	-0.024	0.001	1.013	122300.29	0.007	0.665
Ke 2012[10]	-0.557	-0.047	0.002	1.012	122281.93	0.007	0.676
Ke 2015[155]	-0.383	-0.033	0.001	1.013	122246.39	0.007	0.675
Kim 2020[50]	0.499	0.04	0.002	1.012	121738.31	0.007	0.676
Komang-Agung 2017[51]	0.633	0.05	0.003	1.01	122283.74	0.006	0.634
Koukourakis 1997[194]	-0.102	-0.01	0	1.014	122046.99	0.007	0.676
Kunakornsawat 2017[52]	1.278	0.105	0.011	1.002	122159.15	0.007	0.658
Kuru Çolak 2015[146]	-0.512	-0.043	0.002	1.012	122312.59	0.007	0.662
Lee 2014[89]	-1.266	-0.104	0.011	1.003	122215.38	0.007	0.677
Li 1999b[160]	-0.707	-0.059	0.003	1.011	122307.93	0.007	0.676
Li 1999c[214]	-1.044	-0.086	0.007	1.006	122307.21	0.007	0.676
Li 2001[13]	-0.378	-0.032	0.001	1.013	122160.45	0.007	0.677
Li 2010[12]	-0.277	-0.024	0.001	1.014	122241.9	0.007	0.675
Li 2011[11]	-1.091	-0.09	0.008	1.006	122282.34	0.007	0.677
Li 2017[159]	-0.56	-0.047	0.002	1.012	122289.15	0.007	0.675
Li 2017b[210]	-1.236	-0.101	0.01	1.003	122284.66	0.007	0.675
Liang 2005[215]	-0.516	-0.043	0.002	1.012	122298.04	0.007	0.673
Liu 2001[104]	0.093	0.007	0	1.014	122187.99	0.007	0.673
Liu 2002[105]	-0.63	-0.053	0.003	1.011	122216.3	0.007	0.677
Liu 2003[103]	-0.427	-0.036	0.001	1.013	122240.64	0.007	0.676
Liu 2011[106]	0.083	0.006	0	1.014	121899.1	0.007	0.676
Liu 2024[14]	0.519	0.042	0.002	1.012	122234.65	0.007	0.662
Lonsete 1976[230]	-0.369	-0.031	0.001	1.013	119955.05	0.007	0.678
Lu 2010[15]	0.268	0.021	0	1.013	121889.06	0.007	0.676
Ma 1995[17]	-0.133	-0.012	0	1.014	122068.66	0.007	0.676
Ma 2015[16]	-0.239	-0.021	0	1.014	122265.13	0.007	0.673
Meng 2003[18]	-0.087	-0.008	0	1.014	122123.99	0.007	0.676
Misawa 2015[163]	-0.242	-0.021	0	1.014	122062.65	0.007	0.677
Mittal 1987[148]	-1.38	-0.113	0.013	1.001	122202.65	0.007	0.676
Miu 2017[19]	-0.446	-0.038	0.001	1.013	122083.22	0.007	0.677
Moaleja 2018[149]	0.021	0.001	0	1.01	122315.76	0.005	0.494
Morais 1985[183]	0.208	0.016	0	1.014	121676.93	0.007	0.677
Natasa 2006[91]	0.404	0.032	0.001	1.013	121870.11	0.007	0.675
Nie 2017[213]	0.139	0.01	0	1.014	122258.07	0.007	0.666
Nissinen 1993[72]	2.085	0.171	0.029	0.984	122168.51	0.006	0.639
Nussinovitch 2002[55]	-0.003	-0.001	0	1.014	122284.04	0.007	0.663
Ostojic 2006[56]	0.716	0.058	0.003	1.01	122197.97	0.007	0.664
Owada 1982[73]	-0.639	-0.053	0.003	1.011	122312.73	0.007	0.671
Park 2006[54]	-1.083	-0.089	0.008	1.006	122303.95	0.007	0.676
Penha 2018[203]	-0.118	-0.011	0	1.014	122290.47	0.007	0.664
Pin 1985[211]	0.386	0.031	0.001	1.013	122074.84	0.007	0.673
Pruijs 1996[57]	-0.34	-0.029	0.001	1.013	122152.93	0.007	0.677

Qiu 2022[109]	-0.519	-0.044	0.002	1.012	122273.35	0.007	0.676	
Ramli 2018[184]	-0.993	-0.082	0.007	1.007	122309.94	0.007	0.677	
Ravi 2019[58]	0.268	0.021	0	1.013	122238.2	0.007	0.667	
Ren 2014[20]	-0.844	-0.07	0.005	1.009	122317.54	0.007	0.676	
Robitaille 1984[92]	1.23	0.102	0.01	1.003	121750.69	0.007	0.672	
Rogala 1978[195]	0.155	0.012	0	1.014	121720.63	0.007	0.677	
Santo 2011[116]	0.777	0.063	0.004	1.009	122158.79	0.007	0.666	
Sato 2011[59]	-1.317	-0.108	0.012	1.002	122206.43	0.007	0.677	
Scaturro 2021[150]	3.195	0.257	0.063	0.95	122179.38	0.006	0.602	*
Se-ll 1980[95]	0.33	0.026	0.001	1.013	122174.83	0.007	0.671	
Serbescu 2007[161]	4.058	0.315	0.092	0.922	122192.23	0.006	0.559	*
Shen 2019[21]	0.423	0.034	0.001	1.013	121843.8	0.007	0.675	
Singh 2022[227]	-0.918	-0.076	0.006	1.008	122317.77	0.007	0.671	
Smyrnis 2015[61]	-0.911	-0.074	0.005	1.008	122317.89	0.006	0.644	
Soucacos 1997[143]	0.385	0.031	0.001	1.013	119853.05	0.007	0.677	
Souza 2013[41]	1.3	0.102	0.01	1.001	122278.48	0.006	0.601	
Stirling 1996[62]	-0.857	-0.071	0.005	1.009	122317.8	0.007	0.675	
Sugita 2000[200]	0.747	0.061	0.004	1.01	122154.91	0.007	0.667	
Suh 2011[167]	0.779	0.064	0.004	1.01	62870.99	0.007	0.678	
Sun 2009[111]	-0.138	-0.012	0	1.014	122138.85	0.007	0.676	
Sung 2021[199]	-0.972	-0.08	0.006	1.007	81767.04	0.007	0.678	
Tang 2011b[216]	-1.389	-0.114	0.013	1	122127.88	0.007	0.677	
Tisovsky 2004[99]	-0.07	-0.007	0	1.013	122309.72	0.006	0.628	
Tobias 2019[185]	-0.007	-0.002	0	1.014	122119.07	0.007	0.675	
Tzivian 1978[97]	1.287	0.106	0.011	1.002	121965.63	0.007	0.669	
Ueno 2011[186]	-0.528	-0.044	0.002	1.012	121729.29	0.007	0.678	
Ugras 2010[63]	-1.01	-0.083	0.007	1.007	122316.52	0.007	0.669	
Wang 1996[126]	-0.382	-0.032	0.001	1.013	122218.21	0.007	0.676	
Wang 1998[217]	-0.863	-0.071	0.005	1.009	122317.91	0.007	0.673	
Wang 2007[128]	-1.415	-0.116	0.013	1	122020.73	0.007	0.677	
Wang 2013[22]	-0.742	-0.062	0.004	1.01	122311.64	0.007	0.676	
Wang 2018b[125]	-1.176	-0.097	0.009	1.004	122277.91	0.007	0.676	
Wei 2023[129]	-0.15	-0.013	0	1.014	122145.13	0.007	0.676	
Wen 2021[23]	-0.463	-0.039	0.002	1.013	122199.7	0.007	0.677	
Willner 1982b[189]	0.134	0.01	0	1.014	121990.45	0.007	0.676	
Wong 2005[168]	-0.973	-0.08	0.006	1.007	122306.64	0.007	0.677	
Wu 2021[24]	2.029	0.168	0.028	0.985	122069.56	0.007	0.655	
Xia 2019[130]	-0.438	-0.037	0.001	1.013	122303.99	0.007	0.669	
Yamamoto 2015[60]	-0.928	-0.077	0.006	1.008	122312.18	0.007	0.677	
Yang 2022a[131]	-1.075	-0.089	0.008	1.006	122302.29	0.007	0.676	
Yawn 1999[226]	0.101	0.007	0	1.014	122277.79	0.007	0.662	
Yilmaz 2020[66]	0.385	0.031	0.001	1.013	121841.53	0.007	0.675	
Yong 2009[67]	-0.291	-0.025	0.001	1.014	121714.99	0.007	0.677	
Yu 1995[25]	0.14	0.01	0	1.014	122158.48	0.007	0.673	
Yu 2014[27]	-0.545	-0.046	0.002	1.012	122256.8	0.007	0.677	

Yu 2023[26]	1.96	0.163	0.026	0.987	122066.22	0.007	0.657
Zeng 2019[2]	-0.848	-0.07	0.005	1.009	122315.86	0.007	0.677
Zhang 2003[134]	-1.222	-0.1	0.01	1.003	122300.18	0.007	0.673
Zhang 2008[28]	-0.625	-0.052	0.003	1.011	122292.76	0.007	0.676
Zhang 2011[29]	-0.547	-0.045	0.002	1.012	122315.53	0.007	0.65
Zhang 2017[31]	0.662	0.054	0.003	1.011	120496.57	0.007	0.677
Zhang 2023a[30]	0.991	0.082	0.007	1.007	120506.75	0.007	0.676
Zhao 1996[136]	1.361	0.113	0.013	1	121382.98	0.007	0.674
Zhao 2014[137]	-0.41	-0.035	0.001	1.013	122284.02	0.007	0.673
Zheng 2016[32]	-1.209	-0.099	0.01	1.004	122295.95	0.007	0.674
Zheng 2017[74]	-0.085	-0.008	0	1.014	121389.66	0.007	0.677
Zhou 1984[75]	-1.12	-0.092	0.008	1.005	122313.03	0.007	0.67
Zhou 2008[76]	-0.644	-0.054	0.003	1.011	122285.01	0.007	0.677
Zhou 2022[65]	0.941	0.077	0.006	1.007	121711.42	0.007	0.674
Zhou 2023[33]	-0.128	-0.012	0	1.014	122189.65	0.007	0.675
Zhu 2017[35]	2.295	0.193	0.036	0.977	121550.33	0.007	0.669
Zhu 2021[34]	-0.837	-0.069	0.005	1.009	122312.76	0.007	0.678
Zou 2022[36]	0.911	0.074	0.006	1.008	122163.85	0.007	0.664

S16.2 Leave-One-Out Analysis (Sorted by I^2).

ID	Effect	LLCI	ULCI	I^2	ID	Effect	LLCI	ULCI	I^2
Suh 2011[167]	0.129	0.118	0.14	0.998	Miu 2017[19]	0.13	0.119	0.141	0.999
Sung 2021[199]	0.13	0.119	0.141	0.998	Moaleja 2018[149]	0.129	0.118	0.14	0.999
Abo-Bakr 1992[190]	0.129	0.118	0.14	0.999	Morais 1985[183]	0.129	0.118	0.14	0.999
Adobor 2011[229]	0.13	0.119	0.141	0.999	Natasa 2006[91]	0.129	0.118	0.14	0.999
An 2015[49]	0.13	0.119	0.141	0.999	Nie 2017[213]	0.129	0.118	0.14	0.999
AndradeBarcia 1996[98]	0.13	0.119	0.141	0.999	Nissinen 1993[72]	0.128	0.118	0.139	0.999
Arti 2005[71]	0.13	0.119	0.141	0.999	Nussinovitch 2002[55]	0.129	0.118	0.14	0.999
Aulisa 2019[70]	0.129	0.118	0.14	0.999	Ostojic 2006[56]	0.129	0.118	0.14	0.999
Bondar 2021[37]	0.13	0.119	0.141	0.999	Owada 1982[73]	0.13	0.119	0.141	0.999
Brooks 1975[140]	0.128	0.117	0.138	0.999	Park 2006[54]	0.13	0.119	0.141	0.999
Burwell 1983[218]	0.129	0.118	0.14	0.999	Penha 2018[203]	0.129	0.118	0.141	0.999
Cai 2021[38]	0.129	0.118	0.139	0.999	Pin 1985[211]	0.129	0.118	0.14	0.999
Carcamo 2023[39]	0.129	0.118	0.14	0.999	Pruijs 1996[57]	0.13	0.118	0.141	0.999
Chan 1986[40]	0.129	0.118	0.14	0.999	Qiu 2022[109]	0.13	0.119	0.141	0.999
Chen 2010[193]	0.13	0.119	0.141	0.999	Ramli 2018[184]	0.13	0.119	0.141	0.999
Chen 2012[172]	0.13	0.119	0.141	0.999	Ravi 2019[58]	0.129	0.118	0.14	0.999
Chen 2016[171]	0.129	0.118	0.141	0.999	Ren 2014[20]	0.13	0.119	0.141	0.999
Chen 2023[173]	0.13	0.119	0.141	0.999	Robitaille 1984[92]	0.129	0.118	0.14	0.999
Cheng 2006[77]	0.129	0.118	0.141	0.999	Rogala 1978[195]	0.129	0.118	0.14	0.999
Chu 2023[1]	0.129	0.118	0.14	0.999	Santo 2011[116]	0.129	0.118	0.14	0.999
Dantas 2021[202]	0.129	0.118	0.14	0.999	Sato 2011[59]	0.13	0.119	0.141	0.999
Deepak 2017[85]	0.129	0.118	0.14	0.999	Scaturro 2021[150]	0.128	0.117	0.139	0.999
Deng 2019[3]	0.129	0.118	0.14	0.999	Se-ll 1980[95]	0.129	0.118	0.14	0.999
Dickson 1980[86]	0.129	0.118	0.14	0.999	Serbescu 2007[161]	0.128	0.117	0.138	0.999
Dickson 1983[219]	0.129	0.118	0.14	0.999	Shen 2019[21]	0.129	0.118	0.14	0.999
Ding 2020[4]	0.129	0.118	0.141	0.999	Singh 2022[227]	0.13	0.119	0.141	0.999
Dohnert 2008[42]	0.128	0.117	0.139	0.999	Smyrnis 2015[61]	0.13	0.119	0.141	0.999
Donald 1981[231]	0.129	0.118	0.14	0.999	Soucacos 1997[143]	0.129	0.118	0.14	0.999
Dong 2009[178]	0.13	0.119	0.141	0.999	Souza 2013[41]	0.129	0.118	0.14	0.999
Du 2010[204]	0.13	0.119	0.141	0.999	Stirling 1996[62]	0.13	0.119	0.141	0.999
Du 2016[212]	0.129	0.118	0.14	0.999	Sugita 2000[200]	0.129	0.118	0.14	0.999
Du 2018[179]	0.13	0.118	0.141	0.999	Sun 2009[111]	0.129	0.118	0.141	0.999
Etemadifar 2020[45]	0.13	0.119	0.141	0.999	Tang 2011b[216]	0.13	0.119	0.141	0.999
Gao 2004[5]	0.13	0.119	0.141	0.999	Tisovsky 2004[99]	0.129	0.118	0.14	0.999
Gao 2023[181]	0.128	0.117	0.139	0.999	Tobias 2019[185]	0.129	0.118	0.14	0.999
Glavas 2023[88]	0.129	0.118	0.14	0.999	Tzivian 1978[97]	0.129	0.118	0.14	0.999
Goldberg 1995[228]	0.13	0.119	0.141	0.999	Ueno 2011[186]	0.13	0.119	0.141	0.999
Grivas 2002[147]	0.128	0.118	0.139	0.999	Ugras 2010[63]	0.13	0.119	0.141	0.999
Guo 2017[43]	0.129	0.118	0.14	0.999	Wang 1996[126]	0.13	0.118	0.141	0.999
He 2016[6]	0.13	0.119	0.141	0.999	Wang 1998[217]	0.13	0.119	0.141	0.999
Heine 1981[113]	0.129	0.118	0.14	0.999	Wang 2007[128]	0.13	0.119	0.141	0.999
Hu 2017[7]	0.13	0.119	0.141	0.999	Wang 2013[22]	0.13	0.119	0.141	0.999
Hu 2022[47]	0.129	0.118	0.14	0.999	Wang 2018b[125]	0.13	0.119	0.141	0.999

Huang 2011a[8]	0.13	0.119	0.141	0.999	Wei 2023[129]	0.129	0.118	0.141	0.999
Huang 2011b[154]	0.129	0.118	0.14	0.999	Wen 2021[23]	0.13	0.119	0.141	0.999
Jenyo 2005[46]	0.129	0.118	0.14	0.999	Willner 1982b[189]	0.129	0.118	0.14	0.999
Jia 2022[9]	0.129	0.118	0.14	0.999	Wong 2005[168]	0.13	0.119	0.141	0.999
Karachalios 1999[48]	0.13	0.118	0.141	0.999	Wu 2021[24]	0.128	0.118	0.139	0.999
Ke 2012[10]	0.13	0.119	0.141	0.999	Xia 2019[130]	0.13	0.119	0.141	0.999
Ke 2015[155]	0.13	0.118	0.141	0.999	Yamamoto 2015[60]	0.13	0.119	0.141	0.999
Kim 2020[50]	0.129	0.118	0.14	0.999	Yang 2022a[131]	0.13	0.119	0.141	0.999
Komang-Agung 2017[51]	0.129	0.118	0.14	0.999	Yawn 1999[226]	0.129	0.118	0.14	0.999
Koukourakis 1997[194]	0.129	0.118	0.141	0.999	Yilmaz 2020[66]	0.129	0.118	0.14	0.999
Kunakornsawat 2017[52]	0.129	0.118	0.14	0.999	Yong 2009[67]	0.13	0.118	0.141	0.999
Kuru Çolak 2015[146]	0.13	0.119	0.141	0.999	Yu 1995[25]	0.129	0.118	0.14	0.999
Lee 2014[89]	0.13	0.119	0.141	0.999	Yu 2014[27]	0.13	0.119	0.141	0.999
Li 1999b[160]	0.13	0.119	0.141	0.999	Yu 2023[26]	0.128	0.118	0.139	0.999
Li 1999c[214]	0.13	0.119	0.141	0.999	Zeng 2019[2]	0.13	0.119	0.141	0.999
Li 2001[13]	0.13	0.118	0.141	0.999	Zhang 2003[134]	0.13	0.119	0.141	0.999
Li 2010[12]	0.13	0.118	0.141	0.999	Zhang 2008[28]	0.13	0.119	0.141	0.999
Li 2011[11]	0.13	0.119	0.141	0.999	Zhang 2011[29]	0.13	0.119	0.141	0.999
Li 2017[159]	0.13	0.119	0.141	0.999	Zhang 2017[31]	0.129	0.118	0.14	0.999
Li 2017b[210]	0.13	0.119	0.141	0.999	Zhang 2023a[30]	0.129	0.118	0.14	0.999
Liang 2005[215]	0.13	0.119	0.141	0.999	Zhao 1996[136]	0.129	0.118	0.14	0.999
Liu 2001[104]	0.129	0.118	0.14	0.999	Zhao 2014[137]	0.13	0.119	0.141	0.999
Liu 2002[105]	0.13	0.119	0.141	0.999	Zheng 2016[32]	0.13	0.119	0.141	0.999
Liu 2003[103]	0.13	0.119	0.141	0.999	Zheng 2017[74]	0.129	0.118	0.14	0.999
Liu 2011[106]	0.129	0.118	0.14	0.999	Zhou 1984[75]	0.13	0.119	0.141	0.999
Liu 2024[14]	0.129	0.118	0.14	0.999	Zhou 2008[76]	0.13	0.119	0.141	0.999
Lonsete 1976[230]	0.13	0.118	0.141	0.999	Zhou 2022[65]	0.129	0.118	0.14	0.999
Lu 2010[15]	0.129	0.118	0.14	0.999	Zhou 2023[33]	0.129	0.118	0.141	0.999
Ma 1995[17]	0.129	0.118	0.141	0.999	Zhu 2017[35]	0.128	0.117	0.139	0.999
Ma 2015[16]	0.129	0.118	0.141	0.999	Zhu 2021[34]	0.13	0.119	0.141	0.999
Meng 2003[18]	0.129	0.118	0.14	0.999	Zou 2022[36]	0.129	0.118	0.14	0.999
Misawa 2015[163]	0.129	0.118	0.141	0.999	Mittal 1987[148]	0.13	0.119	0.141	0.999

Appendix S17: Publication bias.

S17.1 Funnel plots.

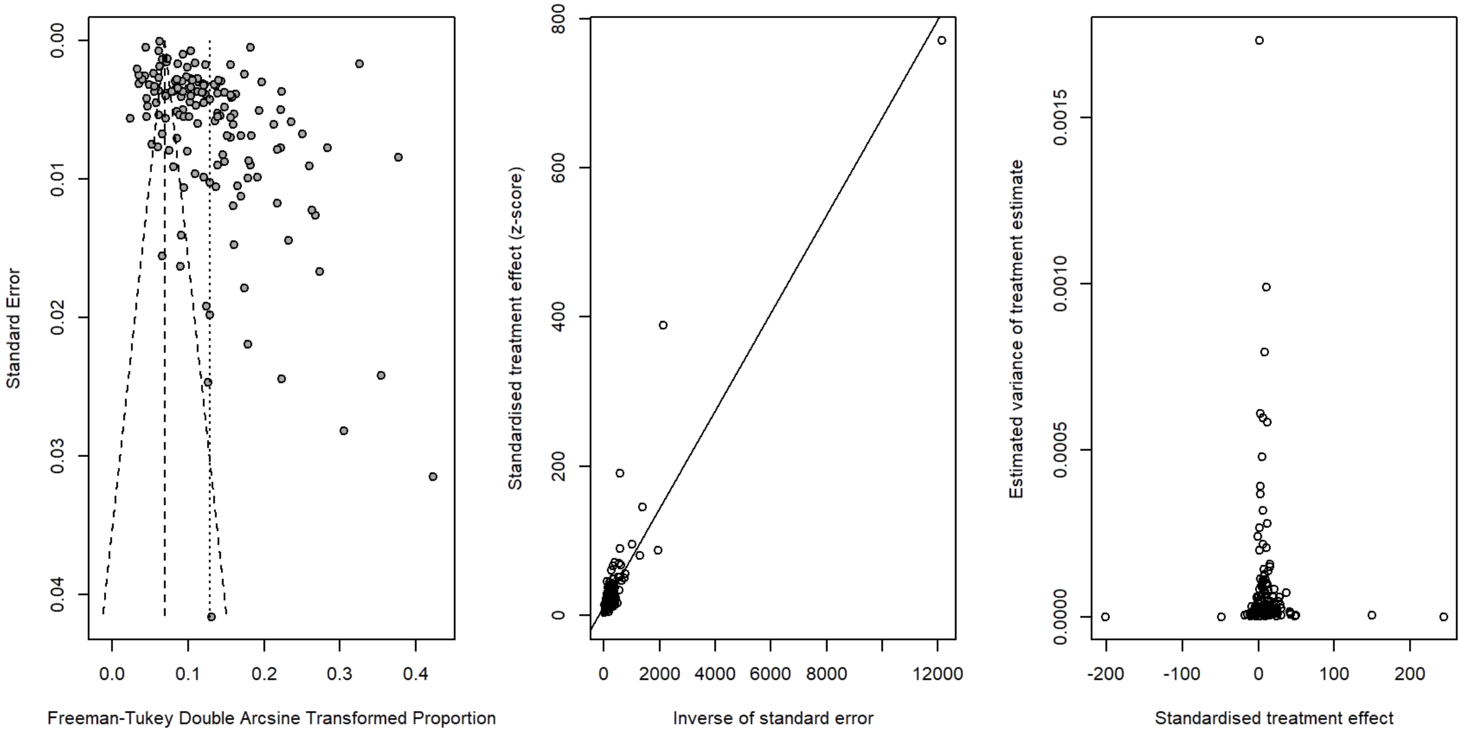

S17.2 Egger's and Begg's test.

Statistical method	Effect size	p value
Egger's test	-0.41	0.6834
Begg's test	5.66	<0.0001

S17.3 The result of the trim and fill method.

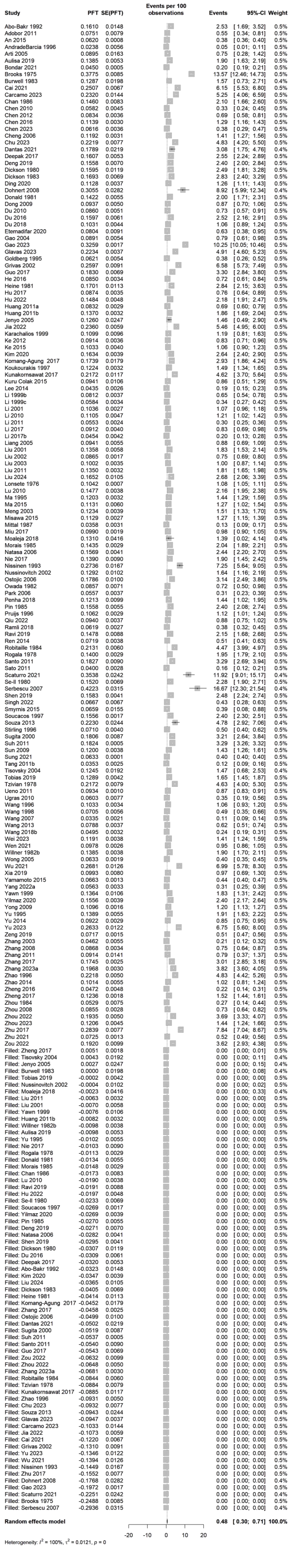

1. Chu L, Zhang F, Qi W, Qi Y. Screening for scoliosis and associated factors among primary and middle school students in Shanghai. Chin J Sch Health 2023; 44(08): 1134-9.
2. Zeng L. Investigation on the prevalence of scoliosis among adolescents in Huizhou area. *Shenzhen J Integr Tradit Chin Med* 2019; **29**(16): 196-7.
3. Deng X, Wu Y, Deng M. Investigation on Current Situation of Adolescent Scoliosis in Ganzi Tibetan Autonomous Prefecture in 2018. *J Prev Med Inf* 2019; **35**(07): 667-70.
4. Ding X, Teng J, Chai S, Li X. A survey of prevalence rate of idiopathic scoliosis of middle school students in Zhengdong new district of Zhengzhou. *J Trad Chin Orthop Trauma* 2020; **32**(06): 31-4+7.
5. Gao W, Ni X, Shao Y. The application and revelation of "point Line" in scoliosis investigation. *Hebei Med* 2004; (12): 1073-5.
6. He T, Zhang J. Investigation and analysis of scoliosis in primary and secondary school students in Jinjiang City. *The Medical Forum* 2016; **20**(36): 5081-2.
7. Hu G, Liu C, Liu H, Yan B. Prevalence and prevention of idiopathic scoliosis among middle school students in Nanshan district of Shenzhen city *Chin community doctors* 2017; **33**(35): 110-1.
8. Huang N, Guo H, Liu J, Huang G. A survey on adolescent scoliosis in Guangzhou. *Chin J Epidemiol* 2011; **32**(2): 138-41.
9. Jia J, Lin Y, Zhu G, Hong X. A cross-sectional study on prevalence of scoliosis among primary and middle school students in Jiaxing City *Prev Med* 2022; **34**(08): 782-7.
10. Ke Y, He J, Pan Z. Survey on prevalence of scoliosis in adolescents in Foshan City. *J Practive Med* 2012; **28**(05): 832-4.
11. Li Q, Yuan Y, Liang D, al. e. A survey on adolescent scoliosis under the step intervention. *J of Clinical Orthop* 2011; **14**(05): 481-3.
12. Li W, Wang Z, Song B, al. e. Scoliosis prevalence survey of adolescents in Guangzhou City during different periods. *J Clin Rehabil Tissue Eng Res* 2010; **14**(46): 8712-6.
13. Li W, Liu S, Chen Z, Zhang G, Ding Y, He J. A survey on scoliosis in school-age population in Guangzhou. *Chin J Pediatr Surg* 2001; (02): 40-2.
14. Liu K, Luo M, Miu X, Xiong J, Ma Y. Analysis of current situation and related factors of scoliosis in adolescents aged 10-16 in Hanyang District, Wuhan City. *Chin J School Doctor* 2024; **38**(01): 8-11+22.
15. Lu M, Chen Q, Gao J, Zhang C, Qu J. A general survey of adolescent scoliosis in Heilongjiang province. *Orthop J Chin* 2010; **18**(07): 591-3.
16. Ma J, Liu Q, Chen Z, Lin M, Gao Z. Epidemiology of adolescent idiopathic scoliosis in Sanya. *Hainan Med J* 2015; **26**(13): 2000-2.
17. Ma X, Zhao B, Lin Q, Wang G. Prevalence of scoliosis in primary and secondary school students in Shanxi Province. *Chin J Epidemiol* 1995; (02): 109-10.
18. Meng L, Meng L, Ao B, Wang Z. An Epidemiological Survey of Scoliosis among Primary and Junior Middle School Students in Langfang Area. *J Med Theor &Prac* 2003; (05): 516-8.
19. Miu G, Xu C. Epidemiological investigation of scoliosis among adolescents in Jiangyin City. *Jiangsu J Prev Med* 2017; **28**(02): 195-6+213.
20. Ren K, Gong X, Zhang R, Zeng X, Zhan J, Liu J. Investigation of Idiopathic Scoliosis in Urban and Rural Middle and Primary School Students in Zigong City. *Chin J School Health* 2014; **35**(12): 1901-2.
21. Sheng H. Epidemiological Study of Scoliosis Among Eighth Grade Students in the Six Districts of Tianjin. 2019.
22. Wang Y, Wu B, Lin Y. A scoliosis-prevalence survey of students in primary and secondary schools in Wenzhou city. *J Trad Chin Orthop Trauma* 2013; **25**(04): 25-7.
23. Wen J, Cui X, Zhang C, et al. A survey on the current situation of adolescent scoliosis in Yuzhong County,Gansu Province. . *Health Med Res Pract* 2021; **18**(05): 35-8.
24. Wu C, Li D, Zhang X. Current Status and Influencing Factors of Scoliosis Among Children and Adolescents in Zhengzhou. . *South China J Prev Med* 2021; **47**(05): 673-5.

25. Yu Z, Qu Z, Wang M, et al. Survey and Early Diagnosis and Treatment of Scoliosis in Rural Primary and Secondary School Students. *Chin J Orthop* 1995; (07): 418-21.
26. Yu D, Zhou L, Zhou Z, Zhou L. Screening and influencing factors of spine problems of children and adolescents aged 6-18 years. *J Pub Health Prev Med* 2023; **34**(04): 135-8.
27. Yu S, Zeng M, Yin D, Yang Z. A survey on the prevalence of scoliosis among teenagers in Guangzhou city. *Acad J Guangzhou Med Univ* 2014; **42**(06): 67-70.
28. Zhang J, Lin G, Zeng X, Gao T, Liu X. A Survey on the Incidence of Juvenile Scoliosis in Quanzhou Area. *Chin J Trad Med Traum & Orthop* 2008; (04): 1-4.
29. Zhang S, Ma S, Liu H, et al. The study about the growing development and scoliosis of Mongolian, Han and Hui pupils in Inner Mongolia. *J Dis Monit Control* 2011; **5**(03): 131-3.
30. Zhang W, Liu F, Wang J, et al. Epidemiological characteristics and influencing factors of scoliosis among primary and secondaryschool students aged 7-15 years in Henan Province. *South China J Prev Med* 2023; **49**(10): 1239-42+47.
31. Zhang Z, Huang F, Wu J, et al. Investigation of the prevalence of idiopathic scoliosis among junior high school students in Zhongshan City. *Inner Mongol Med J* 2017; **49**(05): 541-3.
32. Zheng Y, Wu X, Sun N, Yang Y. Prevalence of Idiopathic Scoliosis in Primary School Children in Beitang District, Wuxi, Jiangsu, China. *Chin J Rehabil Theory Pract* 2016; **22**(03): 335-40.
33. Zhou Y, Wei Z, Xu W, Chen W, Wu Q, Deng C. Current situation and influencing factors of scoliosis in students of primary and secondary school in the community of Huazhong University of Science and Technology of Wuhan City from 2019 to 2023. *J Occup Health Dam* 2023; **38**(03): 163-7.
34. Zhu L, Zhao W, Wu Y. Investigation and rehabilitation intervention of primary and secondaryschool students with scoliosis in Zhengzhou in 2020. *J Med Forum* 2021; **42**(12): 79-84.
35. Zhu X, Liu P, Zhan Y, Ma H. An investigation of scoliosis incidence in middle school graduates in 2014 in yangpu district. *Chin Imaging J Integr Tradit Chin Med* 2017; **15**(04): 426-7+31.
36. Zou Y, Lin Y, He H, et al. Factors affecting scoliosis among children and adolescents. *Chin J Prev Med* 2022; **34**(04): 395-9.
37. Bondar K, Nguyen A, Vatani J, Kessler J. The Demographics and Epidemiology of Infantile, Juvenile, and Adolescent Idiopathic Scoliosis in a Southern California Integrated Health Care System. *Spine (Phila Pa 1976)* 2021; **46**(21): 1468-77.
38. Cai Z, Wu R, Zheng S, Qiu Z, Wu K. Morphology and epidemiological study of idiopathic scoliosis among primary school students in Chaozhou, China. *Environ Health Prev Med* 2021; **26**(1): 71.
39. Cárcamo M, Espinoza P, Rodas M, Urrejola Ó, Bettany-Saltikov J, Grivas TB. [Prevalence, risk of progression and quality of life assessment in adolescents undergoing school screening for adolescent idopathic scoliosis]. *Andes Pediatr* 2023; **94**(1): 78-85.
40. Chan A, Moller J, Vimpani G, Paterson D, Southwood R, Sutherland A. The case for scoliosis screening in Australian adolescents. *Med J Aust* 1986; **145**(8): 379-83.
41. Souza FId, Ferreira RBD, Labres D, Elias R, Sousa APMd, Pereira RE. Epidemiologia da escoliose idiopática do adolescente em alunos da rede pública de Goiânia-GO. *Acta Ortopédica Brasileira* 2013; **21**.
42. Döhnert M, Tomasi E. Validity of computed photogrammetry for detecting idiopathic scoliosis in adolescents. *Brazilian Journal of Physical Therapy* 2008; **12**: 290-7.
43. Guo Y, Jiang Q, Tanimoto T, al. e. Low hospital referral rates of school scoliosis screening positives in an urban district of mainland China. *Medicine (Baltimore)* 2017; **96**(14): e6481.
44. Yang J, Huang S, Cheng M, al. e. Postural habits and lifestyle factors associated with adolescent idiopathic scoliosis (AIS) in China: results from a big case-control study. *J Orthop Surg Res* 2022; **17**(1): 472.
45. Etemadifar M, Hadi A, Nazem K, et al. Epidemiology of adolescent idiopathic scoliosis in Isfahan, Iran: A school-based study during 2014-2015. *J Res Med Sci* 2020; **25**: 48.
46. Jenyo MS, Asekun-Olarinmoye EO. Prevalence of scoliosis in secondary school children in Osogbo, Osun State, Nigeria. *Afr J Med Med Sci* 2005; **34**(4): 361-4.

47. Hu M, Zhang Z, Zhou X, al. e. Prevalence and determinants of adolescent idiopathic scoliosis from school screening in Huangpu district, Shanghai, China. *Am J Transl Res* 2022; **14**(6): 4132-8.

48. Karachalios T, Sofianos J, Roidis N, al. e. Ten-year follow-up evaluation of a school screening program for scoliosis. Is the forward-bending test an accurate diagnostic criterion for the screening of scoliosis? *Spine (Phila Pa 1976)* 1999; **24**(22): 2318-24.

49. An K-C, Park D-H, Kong GM, et al. Prevalence Study of Adolescent Idiopathic Scoliosis in Ten-, Eleven-Year Olds for 10 Years. 2015; **50**: 25-30.

50. Kim S, Uhm JY, Chae DH, al. e. Low Body Mass Index for Early Screening of Adolescent Idiopathic Scoliosis: A Comparison Based on Standardized Body Mass Index Classifications. *Asian Nurs Res (Korean Soc Nurs Sci)* 2020; **14**(1): 24-9.

51. Komang-Agung IS, Dwi-Purnomo SB, Susilowati A. Prevalence Rate of Adolescent Idiopathic Scoliosis: Results of School-based Screening in Surabaya, Indonesia. *Malays Orthop J* 2017; **11**(3): 17-22.

52. Kunakornsawat S, Popan N, Piyaskulkaew C, Pruttikul P, Pluemvitayaporn T, Kittithamvongs P. Prevalence of idiopathic scoliosis in Thai female students aged 11-13 years. *Journal of the Medical Association of Thailand* 2017; **100**: 533-8.

53. McMaster ME, Lee AJ, Burwell RG. Physical activities of Patients with adolescent idiopathic scoliosis (AIS): preliminary longitudinal case-control study historical evaluation of possible risk factors. *Scoliosis* 2015; **10**: 6.

54. Park MS, Lee C-S, Kim Y-T, Ko SH, Eo J, Cho SDJTJoTKOA. Idiopathic Scoliosis in the Eleven Years Old - Prevalence Study. 2006; **41**: 263-7.

55. Nussinovitch M, Finkelstein Y, Amir J, al. e. Adolescent screening for orthopedic problems in high school. *Public Health* 2002; **116**(1): 30-2.

56. Ostojić Z, Kristo T, Ostojić L, et al. Prevalence of scoliosis in school-children from Mostar, Bosnia and Herzegovina. *Coll Antropol* 2006; **30**(1): 59-64.

57. Pruijs JE, van der Meer R, Hageman MA, Keessen W, van Wieringen JC. The benefits of school screening for scoliosis in the central part of The Netherlands. *Eur Spine J* 1996; **5**(6): 374-9.

58. Ravi J, Ramachandran A, S D. Analysis of prevalence and factors influencing adolescent idiopathic scoliosis among school students in thiruvallur district. *Int J Physiother* 2019; **6**.

59. Sato T, Hirano T, Ito T, et al. Back pain in adolescents with idiopathic scoliosis: epidemiological study for 43,630 pupils in Niigata City, Japan. *Eur Spine J* 2011; **20**(2): 274-9.

60. Yamamoto S, Shigematsu H, Kadono F, al. e. Adolescent Scoliosis Screening in Nara City Schools: A 23-Year Retrospective Cross-Sectional Study. *Asian Spine J* 2015; **9**(3): 407-15.

61. Smyrnis P, Alexopoulos A, Sekouris N, et al. Idiopathic Scoliosis Prevalence Is 5 Times Less in Roma Than Greek Children and Adolescents. *Spine Deform* 2015; **3**(3): 253-62.

62. Stirling AJ, Howel D, Millner PA, Sadiq S, Sharples D, Dickson RA. Late-onset idiopathic scoliosis in children six to fourteen years old. A cross-sectional prevalence study. *J Bone Joint Surg Am* 1996; **78**(9): 1330-6.

63. Ugras AA, Yilmaz M, Sungur I, Kaya I, Koyuncu Y, Cetinus ME. Prevalence of scoliosis and cost-effectiveness of screening in schools in Turkey. *J Back Musculoskelet Rehabil* 2010; **23**(1): 45-8.

64. Yan B, Lu X, Qiu Q, Nie G, Huang Y. Association Between Incorrect Posture and Adolescent Idiopathic Scoliosis Among Chinese Adolescents: Findings From a Large-Scale Population-Based Study. *Front Pediatr* 2020; **8**: 548.

65. Zhou L, Yang H, Hai Y, al. e. Scoliosis among children in Qinghai-Tibetan Plateau of China: A cross-sectional epidemiological study. *Front Public Health* 2022; **10**: 983095.

66. Yılmaz H, Zateri C, Kusvuran Ozkan A, al. e. Prevalence of adolescent idiopathic scoliosis in Turkey: an epidemiological study. *Spine J* 2020; **20**(6): 947-55.

67. Yong F, Wong HK, Chow KY. Prevalence of adolescent idiopathic scoliosis among female school children in Singapore. *Ann Acad Med Singap* 2009; **38**(12): 1056-63.

68. Zhang C, Wang Y, Yu J, al. e. Analysis of sagittal curvature and its influencing factors in adolescent idiopathic scoliosis. *Medicine (Baltimore)* 2021; **100**(23): e26274.

69. Zhu L, Ru S, Wang W, al. e. Associations of physical activity and screen time with adolescent idiopathic scoliosis. *Environ Health Prev Med* 2023; **28**: 55.

70. Aulisa AG, Giordano M, Guzzanti V, Falciglia F, Pizzetti P, Toniolo RM. Effectiveness of school scoliosis screening and the importance of this method in measures to reduce morbidity in an Italian territory. *J Pediatr Orthop B* 2019; **28**(3): 271-7.

71. Arti H, Kar S, Tavakoli AR, Javdan MJ, Gangi FJJoSUoMS. Evaluation of scoliosis screening results in 10-14 years old students of Shahrekord. 2005; **7**: 23-7.

72. Nissinen M, Heliövaara M, Seitsamo J, al. e. Trunk asymmetry, posture, growth, and risk of scoliosis. A three-year follow-up of Finnish prepubertal school children. *Spine (Phila Pa 1976)* 1993; **18**(1): 8-13.

73. Owada OH, S.; Takiuchi, Y. School screening on scoliosis in Sapporo, Nishi-ku. *Hokkaido Journal of Orthopedic and Traumatic Surgery* 1982; **26**(1-2): 164-6.

74. Zheng Y, Dang Y, Wu X, al. e. Epidemiological study of adolescent idiopathic scoliosis in Eastern China. *J Rehabil Med* 2017; **49**(6): 512-9.

75. Zhou B, Chen B, Chen X, et al. Trunk Asymmetry and Primary Idiopathic Scoliosis: A Survey of 4,770 Middle School Students. *J Qingdao Univ (Med Sci)* 1984; (02): 87-93.

76. Zhou H, Zhang J, Lin S. Epidemiological investigation of scoliosis among adolescents in Hui′ an county，Fujian province. *Chin J Spine Spinal Cord* 2008; (11): 824-7.

77. Chen B, Li F, Song J. Survey on prevalence rate of scoliosis in 25 725 students of middle schools and elementary school in Xi'an city. *Chin J Tissue Eng Res* 2006; (08): 8-9.

78. Pu G, Wang X, Zhao Z, Lu J. Detection rate and influencing factors of suspected scoliosis among primary and middle school students in Dali Bai Autonomous Prefecture. *J Chin J Orthop* 2022; **42**(21): 1433-40.

79. Huang S, Lin F, Yang J, et al. Prebalence and factors influencing adolescent idiopathic scoliosis in Zhongshan City. *Chin J School Health* 2023; **44**(06): 925-9.

80. Li B, Chen J. School Screening for Scoliosis among Fifth and Sixth Graders of Two Elementary Schools in Taipei. *Chin J Public Health* 1999; **18**(5): 303-12.

81. Li M, Qu Y, Sun Y, Gan P, Shen S. Epidemiological characteristics and influencing factors of scoliosis in primary and secondary school students in Guangdong Province. *Chin J School Health* 2022; **43**(02): 292-5.

82. Zou Y, Zhang R, Chen L, et al. Influencing factors for abnormality of the angle of trunk rotation in primaryand secondary school students. *Chin J Prev Med* 2021; **33**(05): 462-7.

83. Zhang X, Sun Z, Liu Z, Feng B, Xu K. Analysis of epidemiological characteristics and influencing factors of scoliosis among middle school students in Tianjin. . *Chin J School Health* 2023; **44**(01): 115-8.

84. Birgani AG, Mahfouzpour S, Farzinfard F, Baghban ARA, Farideh Y. Assessment of vertebral column (skeletal) disorders in 14-16 years old high school male students at shaheed beheshti medical university, tehran-Iran. 2006.

85. Deepak AS, Ong JY, Choon D, et al. The Clinical Effectiveness of School Screening Programme for Idiopathic Scoliosis in Malaysia. *Malays Orthop J* 2017; **11**(1): 41-6.

86. Dickson RA, Stamper P, Sharp AM, Harker P. School screening for scoliosis: cohort study of clinical course. *Br Med J* 1980; **281**(6235): 265-7.

87. Freire T. Prevalência de escoliose em alunos do quinto ao nono ano, na faixaetária de 10 a 15 anos, matriculados nas escolas públicas municipais de Guaramiranga, CE no primeiro trimestre de 2008. 2008.

88. Glavaš J, Rumboldt M, Karin Ž, et al. The role of school medicine in the early detection and management of adolescent idiopathic scoliosis. *Wien Klin Wochenschr* 2023; **135**(11-12): 273-81.

89. Lee JY, Moon SH, Kim HJ, al. e. The prevalence of idiopathic scoliosis in eleven year-old Korean adolescents: a 3 year epidemiological study. *Yonsei Med J* 2014; **55**(3): 773-8.

90. Lev Ran R, Knishkowy B, Adler B. Screening physical examinations in 25,000 Israeli schoolchildren. *Int J Adolesc Med Health* 2013; **25**(1): 47-53.

91. Nataša M, Milovan Z. The early detection of scoliosis in school children: Gender and age differences at cross-sectional screening. *Medicus* 2006; **7**.
92. Robitaille Y, Villavicencio-Pereda C, Gurr J. Adolescent idiopathic scoliosis: epidemiology and treatment outcome in a large cohort of children six years after screening. *Int J Epidemiol* 1984; **13**(3): 319-23.
93. Mark Sacco MC. The Prevalence of Trunk Asymmetries in the small Island state of Malta *Malta Medical Journal* 2019; **31**(03): 8-16.
94. Vázquez-Lazarte AS. Frequency of adolescent idiopathic scoliosis among high school students from six Northern Lima schools. 2020.
95. Suk SI, Choi IH, Ahn JW, Kim IK. The Incidence of Scoliosis in Korea Part III: The Incidence of Scoliosis in the Middle and High School Students. *jkoa* 1980; **15**(1): 1-6.
96. Wilczyński J. The most common faulty postures among boys aged 13-16 years measured by Moiré's photogrammetric method. *Med Pr* 2006; **57**(4): 347-52.
97. Tzivian JLN, M. A.; Korzhavin, G. M. Deformities of the spine and maldevelopments of the thorax in schoolchildren. *Ortopediya Travmatologiya i Protezirovanie* 1978; **4**: 16-9.
98. Andrade Barcia AAB, Jaime. Escoliosis estructural idiopática y otras alteraciones esqueléticas en escolares de los niveles primario y secundario de la ciudad de portoviejo. *Educ méd contin* 1996; **53**: 17-24.
99. Tisovsky PD, P.; Rehak, L.; Kokavec, M.; Novorolsky, K.; Horvath, J.; Makai, F. Prevalence of trunk abnormalities in children aged 8-14 years in Bratislava. *Lekarsky Obzor* 2004; **53**(9): 341-3.
100. Liao Y, Bai L. Research updates on the pathogenesis of adolescent idiopathic scoliosis and sports intervention. *Chin J School Health* 2022; **43**(9).
101. Li X, Chen H. Present Situation and Analysis of Scoliosis in Children and Adolescents in Fujian Province. *Fujian Sports Sci Technol* 2023; **42**(5): 38-44.
102. Li Z, Zhou J, Zhou Y, Chen Y, Zhou J. Epidemiological Investigation of Scoliosis in Adolescents andChildren in an Art Training School in Leshan City. *West Leather* 2021; **43**(19): 50-2.
103. Liu Z, Li Z, Li Z, Cui J, Nie Z. Natural nistory of the mild adolescent Idiopathic scoliosis. . *Chin J Orthop* 2003; (10): 38-41.
104. Liu R, Xin C. Investigation and Analysis of Scoliosis in Primary and Secondary School Students in the Yimeng Mountain Area. *Occupation and Health* 2001; (02): 75.
105. Liu S, Li W, Li Y, et al. A survey on adolescent scoliosis in Guangdong province. *Chin J Spine Spinal Cord* 2002; (01): 41-3.
106. Liu W, Qinghe, Wang L, et al. A general survey of the adolescent scoliosis in Harbin and an analysis of the result of the brace treatment. *Orthop J Chin* 2011; **19**(15): 1244-7.
107. Meng Z, He L, Ye Y, Pan C, Chen M. Epidemic situation of common diseases in schools in Longhua District ofShenzhen City from 2015-2016. *Occupation and Health* 2018; **34**(11): 1548-52.
108. Qiao B, Gao D, Zhao W, Zhang Y. Investigation and Analysis of the Current Situation of Scoliosis AmongPrimary and Secondary School Students in Liangzhou District in 2020. *Smart Healthcare* 2022; **8**(19): 130-3.
109. Qiu Y. Epidemiological Survey of Adolescent Idiopathic Scoliosis Among Primary and Secondary School Students in Jiangyin City. *Doctor* 2022; **7**(4): 90-2.
110. Ren Y, Zhu Z, Wang J, Yang J. Analysis of Scoliosis Screening in Primary School Students in Xixia District, Yinchuan City, 2021. *Ningxia Med J* 2021; **43**(12): 1193-4.
111. Sun R, Yin X, Liu G, et al. Analysis of the Prevalence Survey of Adolescent Idiopathic Scoliosis in the Central Urban Area of Liupanshui City, Guizhou Province, in 2007. *Guizhou Med J* 2009; **33**(01): 73-4.
112. Tang Y. Analysis of the Detection Results of Spinal Abnormal Curvature Among Junior High School Students in a School from 2006 to 2010. *Chin J School Doctor* 2011; **25**(05): 335-6.
113. Heine J, Heine F. On the incidence of thoracal scoliosis in juveniles (author's transl). *Z Orthop Ihre Grenzgeb* 1981; **119**(4): 340-3.

114. Bunnell WP. Outcome of spinal screening. *Spine (Phila Pa 1976)* 1993; **18**(12): 1572-80.
115. Al Daajani MM, Al-Habib DM, Ibrahim MH, et al. Prevalence of Health Problems Targeted by the National School-Based Screening Program among Primary School Students in Saudi Arabia, 2019. *Healthcare (Basel)* 2021; **9**(10).
116. do Espírito Santo A, Guimarães LV, Galera MF. Prevalence of idiopathic scoliosis and associated variables in schoolchildren of elementary public schools in Cuiabá, state of Mato Grosso, 2002. *Rev Bras Epidemiol* 2011; **14**(2): 347-56.
117. On A. Prevalence of Scoliosis among Primary School Children Aged 12-14 Years Living in a Town in Western Turkey. 2013.
118. Nery LS, Halpern R, Nery PC, al. e. Prevalence of scoliosis among school students in a town in southern Brazil. *Sao Paulo Med J* 2010; **128**(2): 69-73.
119. Fernández Sánchez M, Zurita Ortega F, Fernández Sánchez C, Fernández García R, Muñoz-Cruzado y Barba M, Labajos Manzanares MT. Prevalencia de escoliosis, dominancia manual lateral y transporte de material en una población masculina de 6–12 años. *Apunts Medicina de l'Esport* 2010; **45**(168): 243-9.
120. Safikhani Z, Fakor M, Soori H, Hejazian L. Prevalence of scoliosis in female students 11-15 years of age in Ahwaz, Iran. *Neurosciences (Riyadh)* 2006; **11**(2): 97-8.
121. Ciaccia MCC, Castro JS, Rahal MA, et al. PREVALENCE OF SCOLIOSIS IN PUBLIC ELEMENTARY SCHOOL STUDENTS. *Rev Paul Pediatr* 2017; **35**(2): 191-8.
122. Petrovic J, Puzović V, Djordjevic D, Obrenovic MR, Medic V, Jakovljevic VLJ. PREVALENCE OF SPINE DEFORMITY AMONG 7-11 YEAR OLD CHILDREN. 2012; 2012.
123. Wang G, Gao Y, Guo W, Zhang Y, Ruan H. Analysis of common diseases surveillance in students in Yongjing countyof Gansu province from 2019 to 2021. *Bull Dis Control Prev (China)* 2022; **37**(06): 40-4.
124. Wang H, Sun Z, Wang T, Duan Y. Prevalence and risk factors of adolescent idiopathic scoliosis in Kunming. *Chin J School Health* 2018; **39**(12): 1851-4.
125. Wang Y, Chen X, Yuan X, et al. The epidemiological investigation of adolescent scoliosis in Beijing tongzhou district. *Chin J Spine Spinal Cord* 2018; **28**(07): 667-9.
126. Wang Y, Ye Q, Wu B, Wu 吴之康 K. Beijing Area Prevalence Survey Report on Scoliosis. *Chin J Epidemiol* 1996; (03): 160-2.
127. Wang Y. Prefecture School-Age Children and Adolescent Scoliosis Survey Study in Dali. 2022.
128. Wang Z, Li Z, Liu Z, et al. Investigation of scoliosis among school childr en in Beijing. *Chin J Spine Spinal Cord* 2007; (06): 440-2.
129. Wei J, Xue H, Li Y, Mao S, Jiang L, Wang D. Investigation on scoliosis incidence among primary and secondary school adolescents of Laoshan district in Qingdao. *Ningxia Med J* 2023; **45**(12): 1113-5.
130. Xia C, Guan J, Ma L, Cai Y, Shen J. Investigation of prevalence of scoliosis among adolescents in a community of JiadingDistrict, Shanghai. *Shanghai Med Pharm J* 2019; **40**(08): 53-5.
131. Yang R, Ren Y, Chen K, Li Z, Cheng X, Liu W. Study on the current status of spinal morphology and factors influencing it in primary andsecondary school students in Taiyuan. *J Shanxi Univ Chin Med* 2022; **23**(05): 452-5.
132. Yu H, Liu Z, Qiu A, et al. Analysis of the Current Situation and Influencing Factors of Scoliosis in Primary and Secondary School Students in Xiamen. *Chin J School Health* 2010; **31**(10): 1271-2.
133. Zhang L, Zhao Z, Wang Y, et al. Epidemiological Characteristics of Scoliosis in Children and Adolescents in Nanhua County of Yunnan Province. *Orthop* 2023; **14**(02): 150-4.
134. Zhang S, Jin X, Guo X, et al. A Survey on Scoliosis in School－age Population in Hainan. *Hainan Med J* 2003; (12): 5-6.
135. Zhao F, Zhao R, Duan J, Li L. Analysis of the Status and Influencing Factors of Spinal Curvature Abnormalities in Primary and Secondary School Students Aged 9-18 in a District of Beijing. *South China J Prev Med* 2022; **48**(10): 1272-4.
136. Zhao G, Tian J, Wu X, Shi Q. Survey on the Prevalence of Scoliosis Among Schoolchildren in Some Primary and Secondary Schools in Shanghai. . *Chin J Trad Med Traum & Orthop* 1996; (06): 30-1.
137. Zhao Z, Lan H, Wang Z, Feng J, Wu D. Investigation and study of prevalence rate of adolescent scoliosis inGuangzhou Liwan district. *Chin Mod Med* 2014; **21**(19): 137-9.

138. Assiri AM, Awadalla AM, Abolyazid AY, Abogamal MSA, Alsabaani AAJIJoMR, Sciences H. School Screening for Scoliosis among Male Adolescents in Abha City, Southwestern Saudi Arabia. 2019; **8**: 190-5.
139. Willner S. Prevalence study of trunk asymmetries and structural scoliosis in 10-year-old school children. *Spine (Phila Pa 1976)* 1984; **9**(6): 644-7.
140. Brooks HL, Azen SP, Gerberg E, Brooks R, Chan L. Scoliosis: A prospective epidemiological study. *J Bone Joint Surg Am* 1975; **57**(7): 968-72.
141. Zurita Ortega F, Moreno Lorenzo C, Ruiz Rodríguez L, al. e. Screening of scoliosis in a school population of 8 to 12 years in the province of Granada (Spain). *An Pediatr (Barc)* 2008; **69**(4): 342-50.
142. Tanchev P, Dikov D, Dzherov A, et al. School screening for scoliosis in Sofia. An analysis of screening results of 4800 students. 1996; **33**: 69-73.
143. Soucacos PN, Soucacos PK, Zacharis KC, Beris AE, Xenakis TA. School-screening for scoliosis. A prospective epidemiological study in northwestern and central Greece. *J Bone Joint Surg Am* 1997; **79**(10): 1498-503.
144. Smyrnis PN, Valavanis J, Alexopoulos A, Siderakis G, Giannestras NJ. School screening for scoliosis in Athens. *J Bone Joint Surg Br* 1979; **61-b**(2): 215-7.
145. Cilli K, Tezeren G, Taş T, et al. School screening for scoliosis in Sivas, Turkey. *Acta Orthop Traumatol Turc* 2009; **43**(5): 426-30.
146. Çolak TK, Apti A, Dereli EE, Özdinçler AR, Çolak İ. Scoliosis screening results of primary school students (11-15 years old group) in the west side of Istanbul. *J Phys Ther Sci* 2015; **27**(9): 2797-801.
147. Grivas TB, Samelis P, Polyzois BD, al. e. School screening in the heavily industrialized area--Is there any role of industrial environmental factors in idiopathic scoliosis prevalence? *Stud Health Technol Inform* 2002; **91**: 76-80.
148. Mittal RL, Aggerwal R, Sarwal AK. School screening for scoliosis in India. The evaluation of a scoliometer. *Int Orthop* 1987; **11**(4): 335-8.
149. Moalej S, Asadabadi M, Hashemi R, al. e. Screening of scoliosis in school children in Tehran: The prevalence rate of idiopathic scoliosis. *J Back Musculoskelet Rehabil* 2018; **31**(4): 767-74.
150. Scaturro D, Costantino C, Terrana P, et al. Risk Factors, Lifestyle and Prevention among Adolescents with Idiopathic Juvenile Scoliosis: A Cross Sectional Study in Eleven First-Grade Secondary Schools of Palermo Province, Italy. *Int J Environ Res Public Health* 2021; **18**(23).
151. Guo H, Huang J, Jin H, et al. Prevalence of early-onset and adolescent scoliosis and its associatesamong primary and secondary school students in Qinghai province: ascreening survey. *Chin J Public Health* 2023; **39**(06): 762-8.
152. Hai B, Shen H, Liu M, Hu J, Yang H, Shen L. Survey on Scoliosis among Primary and Secondary School Students in Suzhou. *Chin J Prev Med* 2021; **33**(09): 940-3.
153. He Y, Guan B, Wang X, et al. Investigation and Research on the Incidence of Adolescent Idiopathic Scoliosis among Middle School Students in Xining. *Qinghai Med J* 2018; **48**(04): 69-71.
154. Huang X, Du H, Wang Y, Chen Y, Chen Y. Analysis of Adolescent Idiopathic Scoliosis and Myopic Anisometropia in Wenzhou. *Chin J School Health* 2011; **32**(05): 625.
155. Ke R, Cao X, Huang Y, Liu F, Chen Y, lv F. Prevalence of adolescent idiopathic scoliosis in Zhenjiang. *Jiangsu Med J* 2015; **41**(18): 2130-2.
156. Li F, Chang L, Yang Y, et al. Prevalence and influencing factors of vertebral column defects amongprimary and secondary school students in Dali City. *South China J Prev Med* 2023; **49**(08): 975-80.
157. Li J. Epidemiological Study on the Incidence of Adolescent Idiopathic Scoliosis Among Junior High School Students. *Med Health* 2022; (8).
158. Jiang H, Jiang Y, Zhao C, Tian C, Wang J, Li L. A Survey on the Prevalence of Scoliosis Among Students in 57 Primary Schools in Hongqiao District, Tianjin City. *Chin J Orthop* 1994; (06): 362-4.
159. Li M, Su L, Zhong H, et al. An Investigation about the Prevalence Rate of Idiopathic Scoliosis in Middle School Students in Shenzhen. *Shenzhen J Integr Tradit Chin Med* 2018; **28**(02): 3-5.

160. Li Q, Liu S, Xu Z, et al. A General Survey and Treatment of Scoliosis in Primary and Middle School Students in Zhongshan of Guangdong. *Chin J Orthop* 1999; (05): 9-12.

161. Serbescu C, Ianc D, Straciuc O, Carp G, Courteix D. Étude épidémiologique de la scoliose chez les enfants roumains scolarisés. *Science & Sports* 2007; **22**(6): 307-8.

162. Robles MS, Torres CAJApteo. Evaluación del Programa de Salud Escolar de un centro de salud urbano (curso 2008-2009). 2011; **69**: 112-6.

163. Misawa A, Hongo M, Kudo D, al. e. Evaluation of scoliosis screening using Moire topography in school children. *Scoliosis* 2015; **10**: P9.

164. Baró A, Gómez F, Montero C, Meneses D, Godoy W. Experiencia en la implementación de un protocolo para el tamizaje de escoliosis idiopática del adolescente en instituciones educativas de Bogotá. *Investigaciones en Seguridad Social y Salud* 2019; **21**: 4-10.

165. Baroni MP, Sanchis GJ, de Assis SJ, et al. Factors associated with scoliosis in schoolchildren: a cross-sectional population-based study. *J Epidemiol* 2015; **25**(3): 212-20.

166. Gashaw M, Janakiraman B, Belay GJ. Idiopathic scoliosis and associated factors among school children: a school-based screening in Ethiopia. *Arch Public Health* 2021; **79**(1): 107.

167. Suh SW, Modi HN, Yang JH, Hong JY. Idiopathic scoliosis in Korean schoolchildren: a prospective screening study of over 1 million children. *Eur Spine J* 2011; **20**(7): 1087-94.

168. Wong HK, Hui JH, Rajan U, Chia HP. Idiopathic scoliosis in Singapore schoolchildren: a prevalence study 15 years into the screening program. *Spine (Phila Pa 1976)* 2005; **30**(10): 1188-96.

169. Group. A Preliminary Investigation of Spinal Curvature in Primary and Secondary School Students in Xining Translated into English *Qinghai Med J* 1980; (04): 9-12.

170. Zeng X, Xie N, Zhong X, Chen X. Investigation of Spinal Curvature Abnormalities Among 21,668 Primary and Secondary School Students in Quanzhou City. *Chin J Public Health* 2006; (08): 912.

171. Chen J, Yang F, Guo H, et al. Investigative Study on Adolescent Idiopathic Scoliosis in Urban Areas of Xi'an City. *Shaanxi Med J* 2016; **45**(03): 371-3.

172. Chen L, Chen H, Lin J, Ao R, Lai H, Zeng X. A General Survey of Scoliosis in Primary and Middle School Students in YangJiang Area of Guangdong Province. *Med Innov China* 2012; **9**(18): 89-91.

173. Chen P, Li S, Li R. A Study on Early Screening and Epidemiological Characteristics of Idiopathic Scoliosis in Preschool Children. *New Mom and New Born* 2023; (2): 25-7.

174. Chen X, Ru S, Zhu Z, et al. Analysis of Scoliosis Screening Results of Primary and Middle School Students in Shenzhen. *Shenzhen J Integr Tradit Chin Med* 2022; **32**(04): 5-8.

175. Chen Y, Li L, Yang H, Hu W, Jia F, Zhai F. Current status and influencing factors of scoliosis of children in Shijiazhuang. *Chin J School Health* 2021; **42**(11): 1674-8.

176. Deng W, Zhang J, Du R, Wang X. Abnormal spinal curvature situation and influencing factors in schoolchildren in Xi-angyang City. *Chin J School Doctor* 2016; **30**(04): 285-7.

177. Di J, Xing B, Lei G, Fang X, Zhang C. Analysis of Common Disease Surveillance in Primary and Secondary School Students in Gaotai County in 2019. *Bull Dis Control Prev (China)* 2020; **35**(05): 70-2+5.

178. Dong Z, Xiong L, Zhou P, Tang X, Xiao Q. Investigation of Scoliosis Among School Children in Nanchang. *J Nanchang Univ Med Sci* 2009; **49**(02): 129-32.

179. Du J, Cai S, Jiang B, Zhao Z, Ma Z. Survey analysis of idiopathic scoliosis in 12 881 junior middle school students from Shantou city,Guangdong province. *Chin J Prima Med Pharm* 2018; **25**(15): 1976-9.

180. Duan S, Jin Q, Sun X, Wei Q. Prevalence of scoliosis among primary school students in Shinan District,Qingdao. *J Community Health* 2023; **21**(23): 1212-6.

181. Gao C, Xu Y, Ma H. A Survey on the Prevalence of Idiopathic Scoliosis Among Primary and Secondary School Students in Tangshan Area. *Renowned Doctor* 2023; (17): 24-6.

182. Kapoor M, Laham SG, Sawyer JR. Children at risk identified in an urban scoliosis school screening program: a new model. *J Pediatr Orthop B* 2008; **17**(6): 281-7.
183. Morais T, Bernier M, Turcotte F. Age- and sex-specific prevalence of scoliosis and the value of school screening programs. *Am J Public Health* 1985; **75**(12): 1377-80.
184. MS R. 5 Years Experience Of School Scoliosis Screening Program In Perak Population-A Clinical Evaluation Of Epidemiology, Effectiveness And Limitation Of Scoliosis Screening From 2011 To 2015.
185. Tobias JH, Fairbank J, Harding I, Taylor HJ, Clark EM. Association between physical activity and scoliosis: a prospective cohort study. *Int J Epidemiol* 2019; **48**(4): 1152-60.
186. Ueno M, Takaso M, Nakazawa T, et al. A 5-year epidemiological study on the prevalence rate of idiopathic scoliosis in Tokyo: school screening of more than 250,000 children. *J Orthop Sci* 2011; **16**(1): 1-6.
187. Willner S. A comparative study of the efficiency of different types of school screening for scoliosis. *Acta Orthop Scand* 1982; **53**(5): 769-74.
188. Willner S. Development of trunk asymmetries and structural scoliosis in prepuberal school children in Malmö: follow-up study of children 10-14 years of age. *J Pediatr Orthop* 1984; **4**(4): 452-5.
189. Willner S, Udén A. A prospective prevalence study of scoliosis in Southern Sweden. *Acta Orthop Scand* 1982; **53**(2): 233-7.
190. Abo-Bakr A, Al-Mazyiad A, Al-Hussein M, Al-Sudairy R, Krimli M, Patel PJ. Adolescent idiopathic scoliosis screening of schoolgirls. *Ann Saudi Med* 1992; **12**(6): 555-7.
191. Aisha Mohd Din AMAL, Nurul Nadia Subandi. Associated Factors of Growth With the Prevalence of Adolescent Idiopathic Scoliosis Among Female Primary School Children in Kuala Langat. *Mal J Med Health Sci* 2021: 273-83.
192. Baba MR, Shenoy RM, Soman AJIJoPHR, Development. A Cost-Effective and Innovative Screening Approach, for Idiopathic Scoliosis in Girls, Before their Skeletal Maturity. 2020.
193. Chen C, Tong B, Cong Y, et al. Investigation of Scoliosis Prevalence Among Primary and Middle School Students in Jinzhou, Liaoning Province. *Chin Med Innov* 2010; **7**(8): 44-6.
194. Koukourakis I, Giaourakis G, Kouvidis G, al. e. Screening school children for scoliosis on the island of Crete. *J Spinal Disord* 1997; **10**(6): 527-31.
195. Rogala EJ, Drummond DS, Gurr J. Scoliosis: incidence and natural history. A prospective epidemiological study. *J Bone Joint Surg Am* 1978; **60**(2): 173-6.
196. Minghelli B, Nunes C, Oliveira R. Prevalence of scoliosis in southern Portugal adolescents. *Pediatr Endocrinol Rev* 2014; **11**(4): 374-82.
197. Zou Y, Lin Y, Meng J, al. e. The Prevalence of Scoliosis Screening Positive and Its Influencing Factors: A School-Based Cross-Sectional Study in Zhejiang Province, China. *Front Public Health* 2022; **10**: 773594.
198. Yuan P, Wang ZH, Jiang H, al. e. Prevalence and plasma exosome-derive microRNA diagnostic biomarker screening of adolescent idiopathic scoliosis in Yunnan Province, China. *Front Pediatr* 2024; **12**: 1308931.
199. Sung S, Chae HW, Lee HS, et al. Incidence and Surgery Rate of Idiopathic Scoliosis: A Nationwide Database Study. *Int J Environ Res Public Health* 2021; **18**(15).
200. Sugita K. Epidemiological study on idiopathic scoliosis in high school students. Prevalence and relation to physique, physical strength and motor ability. *Nihon Koshu Eisei Zasshi* 2000; **47**(4): 320-5.
201. Wei C, Lu Z, Huang X, deng. Screening for Abnormal Thoracic Kyphosis Angles Among 3483 Adolescents in Yichang City. *Chin J Gen Pract* 2018; **17**(1): 59-62.
202. Dantas MGB, Aquino AN, Correia HJ, et al. Prevalence of Back Pain and Idiopathic Scoliosis in Adolescents From the Semiarid Region of Brazil: A Cross-sectional Study. *J Chiropr Med* 2021; **20**(3): 97-107.
203. Penha PJ, Ramos N, de Carvalho BKG, Andrade RM, Schmitt ACB, João SMA. Prevalence of Adolescent Idiopathic Scoliosis in the State of São Paulo, Brazil. *Spine (Phila Pa 1976)* 2018; **43**(24): 1710-8.
204. Du Q, Yin H, Huang M, et al. Epidemiological Survey Report on the Prevalence of Adolescent Idiopathic Scoliosis Among Primary and Secondary School Students in Shunde District. *Lingnan Modern Clinics in Surgery* 2010; **10**(1): 52-4.

205. Sun Y, Liu W, Xiong L, et al. Analysis of the Current Status and Influencing Factors of Scoliosis in Middle School Students in Guangzhou City. *Chin J Sch Health* 2021; **42**(12): 1867-70+73.
206. Jian J, Jian X. Investigation and Analysis of Scoliosis in 6395 Primary and Secondary School Students. *Chin J Prima Health Care* 2024; **38**(03): 69-73.
207. Wen X, Xu H, Liu N, Qian L, Huang C. Analysis of the Survey Results on Abnormal Spinal Curvature in Primary and Secondary School Students Aged 7 to 18 in Mianyang City in 2019. *J Prev Med Inf* 2021; **37**(06): 828-32.
208. Yang L, Lu X, Yan B, al. e. Prevalence of Incorrect Posture among Children and Adolescents: Finding from a Large Population-Based Study in China. *iScience* 2020; **23**(5): 101043.
209. HUANG Z, Chen L, Zhang Y, Shui L, Cui L. Analysis of the Detection Results of Scoliosis in Primary and Secondary School Students in Kunming. *Hainan Med J* 2016; **27**(14): 2390-1.
210. Li Y, Cui W, Yan X, Wang H. Survey on the Prevalence of Congenital Scoliosis in Children in Luohe, Henan. *Chin J Pediatr Surg* 2017; **38**(3): 221-4.
211. Pin LH, Mo LY, Lin L, et al. Early diagnosis of scoliosis based on school-screening. *J Bone Joint Surg Am* 1985; **67**(8): 1202-5.
212. Du Q, Zhou X, Negrini S, et al. Scoliosis epidemiology is not similar all over the world: a study from a scoliosis school screening on Chongming Island (China). *BMC Musculoskelet Disord* 2016; **17**: 303.
213. Nie Y, Jin Z, Zhang L, et al. Internet Screening and Early Intervention for Adolescent Idiopathic Scoliosis. *China Mod Doctor* 2017; **55**(16): 16-9+22.
214. Li Y, Li Z, Huang S, Xu R, He H, Liu G. Epidemiological Survey and Early Treatment of Adolescent Idiopathic Scoliosis in the Northern Guangdong Mountainous Area. *Chin J School Doctor* 1999; (06): 415-4.
215. Liang X, Huang S, Yu B, Chen Z. Epidemiological Survey and Prevention of Scoliosis in Preschool Children in the Urban Area of Zhaoqing City, Guangdong Province. *Matemmal and Child Health Care of China* 2005; (12): 1496-7.
216. Tang S, Fu G. Analysis of Routine Survey of Spinal Deformities in 40,579 Primary and Secondary School Students. *J Clin Ped Sur* 2011; **10**(6): 430-3.
217. Wang X, Wang S. Analysis of a Spinal Scoliosis Survey Among 13,560 Adolescents. *J New Med* 1998; (10): 534-5.
218. Burwell RG, James NJ, Johnson F, Webb JK, Wilson YG. Standardised trunk asymmetry scores. A study of back contour in healthy school children. *J Bone Joint Surg Br* 1983; **65**(4): 452-63.
219. Dickson RA. Scoliosis in the community. *Br Med J (Clin Res Ed)* 1983; **286**(6365): 615-8.
220. Tatmatsu-Rocha J, Tatmatsu D, de Araújo Vilela D. Associação entre uso de mochilas escolares e escoliose em adolescentes de escolas públicas e privadasAssociation between use of school backpacks and scoliosis in adolescents in public and private schoolsJ.C.T. Rocha, D.I.B. Tatmatsu, D.A. Vilela. *motricidade* 2012; **8**: 803-9.
221. Pereira LM. ESCOLIOSE: TRIAGEM EM ESCOLARES DE 10 A 15 ANOS. 2016.
222. Penha PJ, João SM, Casarotto RA, Amino CJ, Penteado DC. Postural assessment of girls between 7 and 10 years of age. *Clinics (Sao Paulo)* 2005; **60**(1): 9-16.
223. Bertolini S, Gomes A. ESTUDO DA INCIDÊNCIA DE CIFOSE POSTURAL EM ADOLESCENTES NA FAIXA ETÁRIA DE 11 A 14 ANOS DA REDE ESCOLAR DE MARINGÁ. *Revista da Educação Física/UEM* 2008; **8**.
224. Noll M, Rosa B, Candotti C, al. e. Alterações Posturais em Escolares do Ensino Fundamental de Uma Escola de Teutônia/RS. *Revista Brasileira de Ciência & Movimento* 2012; **20**: 32-42.
225. Liu Y, Xie Y, Wang D, et al. Survey on Adolescent Idiopathic Scoliosis Among Junior High School Students in Longkou City. *Orthop J Chin* 2021; **29**(19): 1749-52.
226. Yawn BP, Yawn RA, Hodge D, al. e. A population-based study of school scoliosis screening. *Jama* 1999; **282**(15): 1427-32.
227. Singh H, Shipra, Sharma V, et al. The first study of epidemiology of adolescent idiopathic scoliosis shows lower prevalence in females of Jammu and Kashmir, India. *Am J Transl Res* 2022; **14**(2): 1100-6.
228. Goldberg CJ, Dowling FE, Fogarty EE, al. e. School scoliosis screening and the United States Preventive Services Task Force. An examination of long-term results. *Spine (Phila Pa 1976)* 1995; **20**(12): 1368-74.

229. Adobor RD, Rimeslatten S, Steen H, Brox JI. School screening and point prevalence of adolescent idiopathic scoliosis in 4000 Norwegian children aged 12 years. *Scoliosis* 2011; **6**: 23.
230. Lonstein JE. Screening for spinal deformities in Minnesota schools. *Clin Orthop Relat Res* 1977; (126): 33-42.
231. Gore DR, Passehl R, Sepic S, al. e. Scoliosis screening: results of a community project. *Pediatrics* 1981; **67**(2): 196-200.
232. Liston C. An evaluation of school screening for scoliosis in Western australia. *Aust J Physiother* 1981; **27**(2): 37-43.
233. Zaina F, Donzelli S, Lusini M, et al. Adolescent idiopathic scoliosis and eating disorders: is there a relation? Results of a cross-sectional study. *Res Dev Disabil* 2013; **34**(4): 1119-24.
234. Kesak-Ursić Đ, Fotez I, Ćurtović A, Katunac L, Bogojević R, Cigić BJPC. Procjena povezanosti perinatalnih čimbenika, ranog psihomotornog razvoja i prirođenih malformacija lokomotornog sustava s pojavnošću idiopatske skolioze radi ranijeg probira rizične djece. 2021; **65**: 13-20.
235. Dobies-Krześniak B, Tarnacka B, Werblińska AJAoA, Medicine E. Joint hypermobility in school-aged children and adolescents with idiopathic scoliosis – A chance for more accurate screening? 2022.
236. Laskowska M, Olczak-Kowalczyk D, Zadurska M, et al. Evaluation of a relationship between malocclusion and idiopathic scoliosis in children and adolescents. *J Child Orthop* 2019; **13**(6): 600-6.
237. Lee WT, Cheung CS, Tse YK, et al. Generalized low bone mass of girls with adolescent idiopathic scoliosis is related to inadequate calcium intake and weight bearing physical activity in peripubertal period. *Osteoporos Int* 2005; **16**(9): 1024-35.
238. Tam EMS, Liu Z, Lam TP, et al. Lower Muscle Mass and Body Fat in Adolescent Idiopathic Scoliosis Are Associated With Abnormal Leptin Bioavailability. *Spine (Phila Pa 1976)* 2016; **41**(11): 940-6.
239. Pjanic S, Jevtic N, Grivas TB. Menarche in Scoliotic and Non-Scoliotic Balkan Girls and the Relationship between Menarche and the Laterality of Scoliotic Curves. *J Clin Med* 2023; **13**(1).

Appendix S18: **List of studies excluded at full-text screening stage.**

	Study ID	Reason
1	Souza 2013[1]	Duplicate literature
2	Chen 2021[2]	Duplicate literature
3	Santo 2011[3]	Duplicate literature
4	Tang 2017[4]	Duplicate literature
5	Huang 2023[5]	Duplicate literature
6	Gabriele 2013[6]	Duplicate literature
7	Sun 2008[7]	Duplicate literature
8	Wang 1996[8]	Duplicate literature
9	Cheng 2006[9]	Duplicate literature
10	Li 2022[10]	Duplicate literature
11	Huang 2018[11]	Duplicate literature
12	huang 2020[12]	Duplicate literature
13	Pu 2023[13]	Duplicate literature
14	Rodríguez 1985[14]	Duplicate literature
15	Seong-Woo 2001[15]	Duplicate literature
16	Milenkovic 2004[16]	Duplicate literature
17	Sakullertphasuk 2015[17]	Duplicate literature
18	Nissinen 1989[18]	Duplicate literature
19	zhou 2023[19]	Duplicate literature
20	Lu 2010[20]	Duplicate literature
21	Huang 2011[21]	Duplicate literature
22	fan 2016[22]	Duplicate literature
23	zhou 2023[23]	Duplicate literature
24	Zhang 2003[24]	Duplicate literature
25	Pu 2022[25]	Duplicate literature
26	Guo 2022[26]	Duplicate literature
27	Gong 2016[27]	Duplicate literature
28	Cheng 2006[28]	Duplicate literature
29	Kim 1988[29]	Duplicate literature
30	Hosny 1991[30]	Without full-text articles available
31	Keskin 1997[31]	Without full-text articles available
32	David 1996[32]	Without full-text articles available
33	CarneiroNeto 1999[33]	Without full-text articles available
34	Figueiredo 1981[34]	Without full-text articles available
35	Mastalerz-Migas 2006[35]	Without full-text articles available
36	Kluszczynski 2007[36]	Without full-text articles available
37	Moezy 2015[37]	Without full-text articles available
38	Hansen 1994[38]	Without full-text articles available
39	Andersen 2000[39]	Without full-text articles available
40	Davoine 1996[40]	Without full-text articles available
41	AlvarezNúñez 1988[41]	Without full-text articles available
42	Lanik 1982[42]	Without full-text articles available
43	Lanik 1980[43]	Without full-text articles available

44	Zairul 2012[44]	Without full-text articles available
45	Perea 2005[45]	Without full-text articles available
46	Venancio 1990[46]	Without full-text articles available
47	J 1993[47]	Without full-text articles available
48	Mohammed 1994[48]	Without full-text articles available
49	Abdullah 1989[49]	Without full-text articles available
50	Yilmaz 2011[50]	Without full-text articles available
51	ChagubMoreno 1992[51]	Without full-text articles available
52	DiscacciatideLértora 2006[52]	Without full-text articles available
53	JimenezGarcia 1996[53]	Without full-text articles available
54	Tahirbegolli 2021[54]	Wrong population
55	Almahmoud 2024[55]	Wrong population
56	Yawn 1999[56]	Irrelevant data
57	Ryan 1987[57]	Wrong population
58	Grivas 2006[58]	Wrong study design
59	Wu 2006[59]	Wrong population
60	Lonner 2010[60]	Wrong population
61	Theroux 2015[61]	Wrong population
62	Taylor 1980[62]	Wrong population
63	Xiao 2022[63]	Wrong study design
64	Wood 2002[64]	Wrong study design
65	He 2019[65]	Wrong study design
66	Liu 2023[66]	Wrong study design
67	Liu 2022[67]	Irrelevant data
68	Kulis 2009[68]	Not scoliosis
69	Lin 1989[69]	Wrong population
70	Qian 1991[70]	Without full-text articles available
71	Nada 2019[71]	Irrelevant data
72	Yang 2012[72]	Irrelevant data
73	Shohat 1988[73]	Not scoliosis
74	Huang 2022[74]	Not scoliosis
75	Detsch 2007[75]	Wrong population
76	Wu 2019[76]	Wrong population
77	Detsch 2007[77]	Duplicate literature
78	Qiu 2007[78]	Wrong population
79	Velezis 2002[79]	Not scoliosis
80	Fong 2012[80]	Without full-text articles available
81	Han 2017[81]	Wrong population
82	Hershkovich 2014[82]	Not scoliosis
83	Gao 2018[83]	Irrelevant data
84	Miu 2016[84]	Wrong study design
85	Sun 2021[85]	Wrong population
86	Lonstein 1977[86]	Not scoliosis
87	Donovic 2009[87]	Wrong study design
88	Kadhim 2020[88]	Not scoliosis

89	Kenanidis 2008[89]	Wrong population
90	Lee 2014[90]	Irrelevant data
91	Zheng 2017[91]	Without full-text articles available
92	Misawa 2015[92]	Without full-text articles available
93	Zeng 2023[93]	Not scoliosis
94	Liu 2020[94]	Without full-text articles available
95	Luk 2010[95]	Irrelevant data
96	Bueno 2013[96]	Not scoliosis
97	Steinberg 2013[97]	Wrong population
98	Grauers 2015[98]	Irrelevant data
99	Huang 1997[99]	Wrong population
100	Zhou 2021[100]	Not scoliosis
101	Liu 2008[101]	Irrelevant data
102	Zhao 2007[102]	Irrelevant data
103	Francis 1987[103]	Not scoliosis
104	Gao 2013[104]	Irrelevant data
105	Liu 2024[105]	Wrong population
106	Nissinen 2000[106]	Wrong population
107	Zhou 2008[107]	Wrong population
108	Liang 2013[108]	Wrong population
109	Zhou 2012[109]	Irrelevant data
110	Watanabe 2017[110]	Not scoliosis
111	Carneiro 2005[111]	Not scoliosis
112	Qi 2021[112]	Wrong population
113	Wang 1985[113]	Wrong population
115	Poussa 2005[115]	Not scoliosis
116	Yuan 2021[116]	Wrong population
117	Feng 2011[117]	Wrong population
118	Mao 2013[118]	Wrong population
119	Lonstein 1982[119]	Wrong population
120	Zloof 2022[120]	Not scoliosis
121	Fang 1986[121]	Not scoliosis
122	Zhang 2000[122]	Not scoliosis
123	Qiu 2008[123]	Irrelevant data
124	Long 2020[124]	Wrong population
125	He 2023[125]	Wrong population
126	Moon 2013[126]	Irrelevant data
127	Miyake 2013[127]	Wrong population
128	Ochsmann 2010[128]	Not scoliosis
129	Qin 2023[129]	Wrong study design
130	Ge 2022[130]	Wrong study design
131	Zhao 2014[131]	Wrong study design
132	Shere 2022[132]	Wrong study design
133	Li 1992[133]	Without full-text articles available
134	Adegoke 2011[134]	Wrong population

135	Zhou 1996[135]	Not scoliosis
136	Drennan 1977[136]	Wrong study design
137	Wang 2006[137]	Without full-text articles available
138	Li 2021[138]	Wrong population
139	Huang 2021[139]	Wrong population
140	Zhang 2023[140]	Wrong population
141	Li 2021[141]	Wrong population
142	WaneeratGalassi 2010[142]	Wrong population
143	Lam 2009[143]	Without full-text articles available
144	Saglam 2024[144]	Wrong study design
145	Kamtsiuris 2007[145]	Irrelevant data
146	Heine 1981[146]	Wrong population
147	Jeon 2021[147]	Wrong study design
148	Gecheva 2023[148]	Not scoliosis
149	Atia 2023[149]	Not scoliosis
150	Qiao 2023[150]	Wrong population
151	Kulis 2006[151]	Wrong study design
152	Zhang 2020[152]	Not scoliosis
153	Li 2021[153]	Wrong population
154	Ropac 2013[154]	Not scoliosis
155	Heffernan 2018[155]	Without full-text articles available
156	Chen 2009[156]	Irrelevant data
157	Daruwalla 1985[157]	Not scoliosis
158	Mulu 2023[158]	Not scoliosis
159	Li 2006[159]	Without full-text articles available
160	Gozdzialska 2016[160]	Wrong study design
161	Herzog 2018[161]	Without full-text articles available
162	Han 2015[162]	Without full-text articles available
163	Group 2010[163]	Wrong study design
164	Bueno 2013[164]	Not scoliosis
165	Guo 2022[165]	Wrong study design
166	He 2023[166]	Wrong population
167	Baidoo 2015[167]	Without full-text articles available
168	Negrini 2015[168]	Wrong study design
169	Zhang 1997[169]	Wrong study design
170	Group 1982[170]	Not scoliosis
171	Leal 2006[171]	Wrong population
172	Lee 2010[172]	Not scoliosis
173	Tobias 2019[173]	Wrong population
174	Leone 2010[174]	Wrong study design
175	Zhu 2015[175]	Without full-text articles available
176	Stolinski 2012[176]	Not scoliosis
177	Yin 2010[177]	Wrong population
178	Li 2021[178]	Wrong population
179	Sun 2021[179]	Wrong population

180	Chen 2023[180]	Wrong population
181	Janusz 2014[181]	Wrong population
182	Walker 1984[182]	Not scoliosis
183	Nikolova 2019[183]	Without full-text articles available
184	Lin 2022[184]	Not scoliosis
185	Beausejour 2007[185]	Wrong population
186	Sun 2024[186]	Wrong population
187	Li 2022[187]	Without full-text articles available
188	Grivas 2002[188]	Without full-text articles available
189	Wang 2008[189]	Wrong population
190	Zhang 2023[190]	Wrong population
191	Ma 2023[191]	Wrong population
192	Zhao 2023[192]	Wrong population
193	Zhang 1996[193]	Wrong population
194	Vercauteren 1982[194]	Not scoliosis
195	Ji 2013[195]	Not scoliosis
196	Se-Il 1978[196]	Not scoliosis
197	Liu 2010[197]	Wrong population
198	Singh 2022[198]	Wrong population
199	Bremberg 1986[199]	Irrelevant data
200	Qin 2017[200]	Irrelevant data
201	Anasheva 2014[201]	Without full-text articles available
202	Wang 1982[202]	Wrong population
203	Ma 2023[203]	Wrong population
204	Fu 2006[204]	Wrong study design
205	Bellyei 1977[205]	Wrong population
206	Jia 2008[206]	Irrelevant data
207	Soucacos 2010[207]	Without full-text articles available
208	Dohnert 2008[208]	Duplicate literature
209	Khalchitsky 2019[209]	Without full-text articles available
210	Ramli 2018[210]	Without full-text articles available
211	Chen 2004[211]	Wrong study design
212	Ding 2005[212]	Irrelevant data
213	Wen 2013[213]	Wrong population
214	Huang 2024[214]	Wrong population
215	Zhang 1990[215]	Wrong population
216	Adamczewska 2019[216]	Irrelevant data
217	Xuan 2014[217]	Without full-text articles available
218	Tan 1988[218]	Wrong population
219	Tan 1984[219]	Wrong population
220	Bulkees 2023[220]	Without full-text articles available
221	Grivas 2002[221]	Irrelevant data
222	Minghelli 2013[222]	Without full-text articles available
223	Yao 1987[223]	Irrelevant data
224	Zhang 1986[224]	Irrelevant data

225	Liu 2009[225]	Wrong population
226	Qiu 2008[226]	Wrong population
227	Yang 2023[227]	Wrong population
228	Chen 2022[228]	Wrong population
229	Ren 2023[229]	Wrong population
230	Chen 2024[230]	Wrong population
231	Hazebroekkampschreur 1992[231]	Not scoliosis
232	Zhao 2022[232]	Irrelevant data
233	Safikhani 2005[233]	Irrelevant data
234	Arienti 2019[234]	Irrelevant data
235	Assiri 2020[235]	Irrelevant data
236	Eksi 2020[236]	Wrong population
237	Wang 2023[237]	Without full-text articles available
238	Taylor 2012[238]	Without full-text articles available
239	Dong 2019[239]	Wrong population
240	LevRan 2013[240]	Not scoliosis
241	Jae-Chul 2001[241]	Wrong population
242	Baba 2020[242]	Irrelevant data
243	Zhou 2024[243]	Wrong population
244	Bai 2023[244]	Wrong population
245	Zhao 2023[245]	Wrong population
246	Huang 2016[246]	Wrong population
247	Koga 1986[247]	Irrelevant data
248	Kratenova 2005[248]	Not scoliosis
249	Kuroki 2018[249]	Wrong population
250	Adamczewska 2020[250]	Irrelevant data
251	Chang 2012[251]	Wrong population
252	Banerjee 2022[252]	Without full-text articles available
253	Lazic 2021[253]	Not scoliosis
254	Normand 2024[254]	Wrong population
255	Se-Il 1984[255]	Wrong population
256	Jeon 2018[256]	Irrelevant data
257	Zhao 2023[257]	Irrelevant data
258	Pedrotti 2007[258]	Without full-text articles available
259	Francis 1988[259]	Wrong population
260	Fu 2022[260]	Not scoliosis
261	Inoue 2002[261]	Irrelevant data
262	Nikolova 2016[262]	Irrelevant data
263	Zhao 2012[263]	Irrelevant data
264	Tzivian 1978[264]	Without full-text articles available
265	Sadler 2019[265]	Irrelevant data
266	AndradeBarcia 1996[266]	Without full-text articles available
267	Yeung 2006[267]	Irrelevant data
268	Liu 2017[268]	Irrelevant data
269	Xu 2017[269]	Irrelevant data

270	Wu 2023[270]	Irrelevant data
271	Xu 2017[271]	Irrelevant data
272	Nelson 2011[272]	Irrelevant data
273	Wu 2021[273]	Irrelevant data
274	ParicioTalayero 1998[274]	Without full-text articles available
275	Negri 1982[275]	Without full-text articles available
276	Tisovsky 2004[276]	Without full-text articles available
277	Xia 2019[277]	Irrelevant data
278	Xu 2015[278]	Irrelevant data
279	Takahashi 2011[279]	Irrelevant data
280	Zhu 2014[280]	Irrelevant data
281	Qiu 2014[281]	Irrelevant data
282	Qiu 2008[282]	Irrelevant data
283	Qiu 2007[283]	Irrelevant data
284	Hitesh 2008[284]	Wrong population
285	Nikolova 2019[285]	Irrelevant data
286	Yuan 2024[286]	Irrelevant data
287	DelCastilloCampos 1997[287]	Wrong population
288	Tisovsky 2004[288]	Without full-text articles available
289	Pratelli 2020[289]	Without full-text articles available
290	Akram 1986[290]	Without full-text articles available
291	M 1993[291]	Without full-text articles available
292	Weisz 1988[292]	Without full-text articles available
293	NguyenHuu 2004[293]	Without full-text articles available
294	Wu 2023[294]	Irrelevant data
295	Ascani 1977[295]	Irrelevant data
296	Wilczynski 2006[296]	Without full-text articles available
297	Biggi 1980[297]	Without full-text articles available
298	Deng 2006[298]	Without full-text articles available
299	Chen 2015[299]	Irrelevant data
300	Garg 2023[300]	Irrelevant data
301	He 2023[301]	Irrelevant data
302	Zhang 2020[302]	Irrelevant data
303	Eun-Su 2007[303]	Wrong study design
304	Min 2023[304]	Irrelevant data

1. Souza FId, RBD Ferreira, D Labres, et al. Epidemiologia da escoliose idiopática do adolescente em alunos da rede pública de Goiânia-GO. Acta ortop bras. 2013;21(4):223-5.
2. Chen-Y-J. Current status and influencing factors of scoliosis of children in Shijiazhuang. Chinese Journal of School Health. 2021(12):1674-8.
3. Espírito Santo Ad, LV Guimarães, MF Galera. Prevalência de escoliose idiopática e variáveis associadas em escolares do ensino fundamental de escolas municipais de Cuiabá, MT, 2002. Rev bras epidemiol. 2011;14(2):347-56.
4. Tang Q-R, Zhu M-L, Shang Y, et al. A survey on the prevalence of idiopathic scoliosis among junior middle school students in Jingan District, Shanghai. International Journal of Bone Science. 2017;38(03):205-6.

5. Huang S-Z, Li F-H, Yang J-L, et al. Status and related factors of idiopathic scoliosis in adolescents in Zhongshan City. School health in China. 2023;44(06):925-9.
6. Gabriele AA, V Guzzanti, F Falciglia, et al. Is the screening able to lower morbidity in the territory? Scoliosis. 2013;8(Supplement 2).
7. Sun R, Yin X-Y, Liu G-Q, Wen C-X. Census analysis of 17555 adolescents with scoliosis. Henan J of Surgery. 2008(03):11-2.
8. Wang YP, QB Ye, B Wu. Result on the screening of scoliosis among school students in Beijing area. Zhonghua liu xing bing xue za zhi = Zhonghua liuxingbingxue zazhi. 1996;17(3):160-2.
9. Cheng B, Li F-T, Song J-H. Prevalence of scoliosis among 25 725 primary and secondary school students in Xi 'an City. Clinical rehabilitation in China. 2006(08):8-9.
10. Li M, Qu Y-B, Sun Y, Gan P, Shen S-J. Epidemic characteristics and influencing factors of scoliosis in primary and secondary school students in Guangdong Province. School health in China. 2022;43(02):292-5.
11. Huang F-L, Wu J-Z, Huang S-Z, et al. Investigation and analysis of prevalence of idiopathic scoliosis in middle school students in Zhongshan City, Guangdong Province. Frontiers of med. 2018;8(27):374-5.
12. Huang F-L, YH Liu, JZ Wu, et al. Incidence of scoliosis among junior high school students in Zhongshan city, Guangdong and the possible importance of decreased miR-30e expression. J Int Med Res. 2020;48(6):8.
13. Pu G-H. Epidemiological study on the risk of scoliosis among primary and secondary school students at different altitudes in Dali, Yunnan Province.Kunming Medical University.2023.
14. Rodríguez CAC, MAM Teixeira, MRdO Casartelli. Escoliose: levantamento epidemiológico em alunos da Escola Estadual Lilia Neves; Vila da Quinta, mun. do Rio Grande, RS, 1982. Vittalle. 1985;1:67-76.
15. Seong-Woo SUH, HUR Chang-Yong, C In-Jung, et al. Idiopathic Scoliosis in Korean Middle School Students: Prevalence study. The Journal of the Korean Orthopaedic Association. 2001:33-8.
16. Milenkovic SM, RI Kocijancic, GA Belojevic. Left handedness and spine deformities in early adolescence. Eur J Epidemiol. 2004;19(10):969-72.
17. Sakullertphasuk W, C Suwanasri, L Saetang, et al. Prevalence of scoliosis among high school students. Journal of the Medical Association of Thailand. 2015;98(Supplement 5):S18-S22.
18. Nissinen M, M Heliovaara, K Tallroth, M Poussa. Trunk asymmetry and scoliosis. Anthropometric measurements in prepuberal school children. Acta Paediatrica Scandinavica. 1989;78(5):747-53.
19. Zhou J, YS Wang, JM Xie, et al. Scoliosis school screening of 139,922 multi-ethnic children in Dali, southwestern China: A large epidemiological study. iScience. 2023;26(12):15.
20. Miao LU, C Qinghe, GAO Jichang. A general survey of adolescent scoliosis in Heilongjiang province. Orthopedic Journal of China. 2006(24).
21. Huang N-Q, HS Guo, J Liu, et al. [A survey on adolescent scoliosis in Guangzhou]. Zhonghua liu xing bing xue za zhi = Zhonghua liuxingbingxue zazhi. 2011;32(2):138-41.
22. Hengwei F, H Zifang, W Qifei, et al. Prevalence of idiopathic scoliosis in Chinese schoolchildren. Spine. 2016;41(3):259-64.
23. Zhou X-X, Zhang X, Jiang Y, Jin Y-Y, Wang Z-R. Current situation and influencing factors of scoliosis in primary and secondary school students in Taizhou City. International Journal of Epidemiology and Infectious Diseases. 2023;50(2).
24. Zhang S, Jin X-H,Liang N,Lin Y-R,Huang S. A survey of adolescent scoliosis and its relationship with melatonin. Chin J of Orthopaedic Surgery. 2003(24):56-8.
25. Pu G-H, Wang X-W, Zhao Z, et al. Detection rate and influencing factors of suspected scoliosis in primary and secondary school students in Dali Bai Autonomous Prefecture. Chinese Journal of Orthopedics. 2022;42(21).
26. Guo H, Lv Z-L, Guo Y-L, Wu M-P, Wang X. Analysis of surveillance results of common diseases among primary and secondary school students in Nanyang City from 2018 to 2020. Clinical medicine. 2022;42(07):28-30.
27. Gong M-L. Prevention of abnormal spinal curvature in children and adolescents. Med information. 2016;29(27):252-3.
28. Cheng B, Li J-T, Song J-H. Prevalence of scoliosis and its prevention and treatment in adolescents in Xi 'an City. Chinese Journal of Spinal cord. 2006;16(3).

29. Bok-Yong KIM, P Jung-Han, KIM Poong-Taek. Cross-sectional Survey for Prevalence Rate of Scoliosis in Primary, Middle and High School Boys in Pusan City. Korean Journal of Preventive Medicine. 1988:217-23.
30. Hosny A AR. Identification of spinal deformities in a sample of preparatory school children. 1991. p. 47-59.
31. Keskin D, H Bodur, F Acar, et al. School screening for scoliosis in Turkish children. European Journal of Physical Medicine and Rehabilitation. 1997;7(2):42-5.
32. David R, A Jamal, M Soudry. Screening for scoliosis in western Galilee schools. Harefuah. 1996;130(5):297-358.
33. Carneiro Neto C, C Pawlowski, M Dirani, et al. Prevalência de escoliose em uma escola da periferia da cidade de Porto Alegre. Pesqui méd (Porto Alegre). 1999;33(1/2):31-3.
34. Figueiredo JD, UM Figueiredo. Incidencia de escoliose no Maranhao. Rev bras ortop. 1981;16(4):121-7.
35. Mastalerz-Migas A, T Krupa, A Muszynska, A Steciwko, J Drobnik. Epidemiology of children's and teenagers faulty posture in Opole voivodeship. Family Medicine and Primary Care Review. 2006;8(3):692-4.
36. Kluszczynski M. The prevalence of postural defects and back asymmetry in children from rural areas. Fizjoterapia Polska. 2007;7(1):71-9.
37. Moezy A, S Jalaei, B Vassaghi. Prevalence of kyphosis and scoliosis deformities among junior high school female students in Tehran with emphasize on effects of physical activities and sitting posture on these deformities. 2015. p. 310-9.
38. Hansen TB. Adolescent idiopathic scoliosis among girls in the Herning region. A follow-up of girls with adolescent idiopathic scoliosis found in an earlier screening at school. Ugeskrift for laeger. 1994;156(35):4979-82.
39. Andersen ML, MO Andersen, GR Andersen, SB Christensen. Prevalence of idiopathic scoliosis in the municipality of Hillerod. Ugeskrift for laeger. 2000;162(25):3595-6.
40. Davoine P, C Rouge, J Grison, et al. Trunk asymmetries and back pain in school children. Annales de Readaptation et de Medecine Physique. 1996;39(4):201-7.
41. Alvarez Núñez R, P Oquendo Vázquez. Factores escolares predisponentes en la escoliosis idiopática. Rev cuba pediatr. 1988;60(5):708-19.
42. Lanik V, M Sojakova, H Urbankova. Questions of depistage of scolioses in children. Lekarsky Obzor. 1982;31(1-2):69-73.
43. Lanik V, H Urbankova, M Sojakova. Early screening in scoliosis. Rehabilitacia Supplementum. 1980;13(21).
44. Zairul K, A Zulkefli, M Faridah, et al. School scoliosis screening program: A report of the first year experience in perak. Malaysian Orthopaedic Journal. 2012;6(SUPPL. A):71.
45. Perea DCBNM, MA Auad, CL Tuicci. Incidência da escoliose idiopática do adolescente em escolares da cidade de Descalvado através do Teste de Adams. Reabilitar. 2005;7(28):17-21.
46. Venancio SI, S Elias, MDCG Rodrigues. Escoliose idiopática: proposta de exame clínico simplificado e sua aplicação em 572 escolares. Rev paul pediatr. 1990;8(30):87-90.
47. J.R C. School screening for scoliosis in Saudi Arabia. 1993. p. 209-12.
48. Mohammed H AT. School screening for scoliosis in Riyadh. 1994. p. 277-80.
49. Abdullah H J. Adolescent idiopathic scoliosis in school children. 1989. p. 213-5.
50. Yilmaz H, C Zateri, S Vurur, C Bakar. The prevalence of scoliosis among primary school children in Canakkale. Turkiye Fiziksel Tip ve Rehabilitasyon Dergisi. 2011;57(SUPPL. 1):140.
51. Chagub Moreno AM, J Gómez Naranjo. Prevalencia de escoliosis en una población escolar urbana. Rev cuba hig epidemiol. 1992;30(2):108-13.
52. Discacciati de Lértora MS, MF Lértora, G Quintero de Lucas. Relación entre actitud postural y disgnacias maxilares en adolescentes de la ciudad de Corrientes. Rev Asoc Argent Ortop Funcional Maxilares. 2006;35(2):35-40.
53. Jimenez Garcia E, A Herrera Rodriguez, P Romero Golvano, F Martinez Delgado. Early detection of spinal deformities in school medical examinations. Revista de Ortopedia y Traumatologia. 1996;40(3):222-7.
54. Tahirbegolli B, R Obertinca, A Bytyqi, et al. Factors affecting the prevalence of idiopathic scoliosis among children aged 8-15 years in Prishtina, Kosovo. Sci Rep. 2021;11(1):16786.

55. Almahmoud OH, B Baniodeh, R Musleh, et al. Assessment of idiopathic scoliosis among adolescents and associated factors in Palestine. J Pediatr Nurs. 2024;74:85-91.
56. Yawn BP, RA Yawn, D Hodge, et al. A population-based study of school scoliosis screening. JAMA-J Am Med Assoc. 1999;282(15):1427-32.
57. Ryan MD, A Nachemson. Thoracic adolescent idiopathic scoliosis: perinatal and environmental aspects in a Swedish population and their relationship to curve severity. Journal of pediatric orthopedics. 1987;7(1):72-7.
58. Grivas TB, E Vasiliadis, O Savvidou, V Mouzakis, G Koufopoulos. Geographic latitude and prevalence of adolescent idiopathic scoliosis. Studies in health technology and informatics. 2006;123:84-9.
59. Wu J, Q Yong, L Zhang, et al. Association of estrogen receptor gene polymorphisms with susceptibility to adolescent idiopathic scoliosis. Spine. 2006;31(10):1131-6.
60. Lonner BS, JD Auerbach, P Sponseller, AD Rajadhyaksha, PO Newton. Variations in Pelvic and Other Sagittal Spinal Parameters as a Function of Race in Adolescent Idiopathic Scoliosis. Spine. 2010;35(10):E374-E7.
61. Théroux J, S Le May, C Fortin, H Labelle. Prevalence and management of back pain in adolescent idiopathic scoliosis patients: A retrospective study. Pain Res Manag. 2015;20(3):153-7.
62. Taylor JR, BS Slinger. Scoliosis screening and growth in Western Australian students. Medical Journal of Australia. 1980;1(10):475-8.
63. Xiao D, Liu H-T, Shi Y-K, Wang-J, Hao-Z. Metrological analysis of the international Adolescent idiopathic scoliosis study. The 12th National Convention on Sport Science of China; 2022.
64. Wood KB. Spinal deformity in the adolescent athlete. Clin Sports Med. 2002;21(1):77-92.
65. He Y. Survey of idiopathic scoliosis among 12-16 year old adolescents in Xining City. 2019-01-18.
66. Liu R-N. Watch out! The incidence of scoliosis in adolescents is 8%. 2023-05-08.
67. Liu BW, S Zhao, L Liu, et al. Aberrant interaction between mutated ADAMTSL2 and LTBP4 is associated with adolescent idiopathic scoliosis. Gene. 2022;814:9.
68. Kulis A, J Jaskiewicz. Concentration of selected regulators of calcium-phosphate balance in girls with idiopathic scoliosis. Ortopedia, traumatologia, rehabilitacja. 2009;11(5):438-47.
69. Lin Z-E. Analysis of students' health status in Fujian Province. Chinese school doctor. 1989(3).
70. Qian G-P. Study on scoliosis and its etiology in Nanjing adolescents. sports science. 1991(06):35.
71. Nada D, C Julien, PH Rompré, et al. Association of Circulating YKL-40 Levels and <i>CHI3L</i>1 Variants with the Risk of Spinal Deformity Progression in Adolescent Idiopathic Scoliosis. Sci Rep. 2019;9:13.
72. Yang T, Q Jia, H Guo, et al. Epidemiological survey of idiopathic scoliosis and sequence alignment analysis of multiple candidate genes. Int Orthop. 2012;36(6):1307-14.
73. Shohat M, T Shohat, M Nitzan, et al. Growth and ethnicity in scoliosis. Acta Orthop Scand. 1988;59(3):310-3.
74. Huang JL, X Zhou, X Li, et al. Regional disparity in epidemiological characteristics of adolescent scoliosis in China: Data from a screening program. Front Public Health. 2022;10:9.
75. Detsch C, AMH Luz, CT Candotti, et al. Prevalence of postural changes in high school students in a city in southern Brazil. Revista Panamericana de Salud Publica/Pan American Journal of Public Health. 2007;21(4):231-8.
76. Wu Z, Y Wang, Z Dai, et al. Genetic Variants of ABO and SOX6 are Associated With Adolescent Idiopathic Scoliosis in Chinese Han Population. Spine. 2019;44(18):E1063-E7.
77. Detsch C, AMH Luz, CT Candotti, et al. Prevalência de alterações posturais em escolares do ensino médio em uma cidade no Sul do Brasil. Rev panam salud pública. 2007;21(4):231-8.
78. Qiu XS, LS Tang, HY Yeung, et al. Effect of genetic polymorphism of MTNR1A gene on adolescent idiopathic scoliosis. Zhonghua wai ke za zhi [Chinese journal of surgery]. 2007;45(18):1264-6.
79. Velezis MJ, PF Sturm, J Cobey. Scoliosis screening revisited: Findings from the District of Columbia. J Pediatr Orthop. 2002;22(6):788-91.

80. Fong DYT, CF Lee, YY Wan, et al. Cost of school scoliosis screening: A cohort analysis of 306,144 students followed until skeletal maturity. Eur Spine J. 2012;21(SUPPL. 3):S313.
81. Han K-Y, Guo T, Liu S-Y, Qu Q-Y. Incidence and imaging characteristics of idiopathic scoliosis among college entrance examination students in Qilihe District and Anning District of Lanzhou City in 2016. Imaging research and medical applications. 2017;1(09):85-6.
82. Hershkovich O, A Friedlander, B Gordon, et al. Association between body mass index, body height, and the prevalence of spinal deformities. Spine Journal. 2014;14(8):1581-7.
83. Junsheng GAO. Correlation analysis between interleukin 6 polymorphism and adolescent idiopathic scoliosis susceptibility and bracing effectiveness. Chinese Journal of Reparative and Reconstructive Surgery. 2018(12):678-84.
84. Miu G-Z. Screening methods and prevalence of scoliosis in children and adolescents in China. Notification of disease Prevention and control. 2016;31(01):11-4+27.
85. Sun Y, Liu W-J, Xiong L-H, et al. Current situation and influencing factors of scoliosis in middle school students in Guangzhou. School health in China. 2021;42(12):1867-70+73.
86. Lonstein JE. Screening for spinal deformities in Minnesota schools. Clin Orthop Rel Res. 1977;126:33-42.
87. Donovic N, C Milic, S Kocic, S Radovanovic. Scoliosis in school children aged from 7 to 8 and conditions in primaryand secondary schools in Kragujevac. Medicinski pregled. 2009;62(9-10):445-9.
88. Kadhim M, T Lucak, S Schexnayder, et al. Current status of scoliosis school screening: targeted screening of underserved populations may be the solution. Public Health. 2020;178:72-7.
89. Kenanidis E, ME Potoupnis, KA Papavasiliou, FE Sayegh, GA Kapetanos. Adolescent idiopathic scoliosis and exercising: Is there truly a liaison? Spine. 2008;33(20):2160-5.
90. Lee JY, SH Moon, HJ Kim, et al. The Prevalence of Idiopathic Scoliosis in Eleven Year-Old Korean Adolescents: A 3 Year Epidemiological Study. Yonsei Medical Journal. 2014;55(3):773-8.
91. Zheng Y, XJ Wu, YN Dang, et al. Prevalence of idiopathic adolescent scoliosis among primary and middle school students in Wuxi, China. Scoliosis and Spinal Disorders. 2017;12(Supplement 1).
92. Misawa A, M Hongo, D Kudo, Y Shimada. Evaluation of scoliosis screening using Moire topography in school children. Scoliosis. 2015;10(SUPPL. 1).
93. Zeng-Jie Z-LRENQZHUHZ-L. Abnormal spinal curvature and associated factors among primary and secondary school students in Sichuan Province. Chinese Journal of School Health. 2023(12):1130-3.
94. Liu YQ, BH Li, HY Zhu, RF Gao, BW Li. Investigation On The Prevalence Of Scoliosis In Primary And Secondary School Students In Changzhou. Med Sci Sports Exerc. 2020;52(7):994-.
95. Luk KDK, CF Lee, KMC Cheung, et al. Clinical effectiveness of school screening for adolescent idiopathic scoliosis: A large population-based retrospective cohort study. Spine. 2010;35(17):1607-14.
96. Bueno RCS, RR Rech. Postural deviations of students in Southern Brazil. Revista Paulista de Pediatria. 2013;31(2):237-42.
97. Steinberg N, I Hershkovitz, S Peleg, et al. Morphological characteristics of the young scoliotic dancer. Phys Ther Sport. 2013;14(4):213-20.
98. Grauers A, JW Wang, E Einarsdottir, et al. Candidate gene analysis and exome sequencing confirm LBX1 as a susceptibility gene for idiopathic scoliosis. Spine Journal. 2015;15(10):2239-46.
99. Huang SC. Cut-off point of the scoliometer in school scoliosis screening. Spine. 1997;22(17):1985-9.
100. Zhou Z-Y, Ye Y-Q. Analysis of common diseases among students in Xianyou County in 2019. Straits Journal of Preventive Medicine. 2021;27(2).
101. Zhou Z-Y, Ye Y-Q. Analysis of common diseases among students in Xianyou County in 2019. Straits Journal of Preventive Medicine. 2021;27(02):55-7.
102. Zhao D. Association between SNP polymorphism of CALM1 gene and ER1 gene and idiopathic scoliosis in adolescents. China Union Medical College.2007.

103. Francis RS, GR Bryce. Screening for musculoskeletal deviations--a challenge for the physical therapist. The Utah Study. Phys Ther. 1987;67(8):1221-5.
104. Ghanbari F, N Otomo, I Gamache, et al. Interrogating Causal Effects of Body Composition and Puberty-Related Risk Factors on Adolescent Idiopathic Scoliosis: A Two-Sample Mendelian Randomization Study. JBMR Plus. 2023;7(12):e10830.
105. Liu L, X Wang, SQ Du, et al. Prevalence of adolescent idiopathic scoliosis in Shijiazhuang, Hebei, China: a cross-sectional study. Eur Spine J. 2024;33(2):673-9.
106. Nissinen MJ, MM Heliovaara, JT Seitsamo, et al. Development of trunk asymmetry in a cohort of children ages 11 to 22 years. Spine. 2000;25(5):570-4.
107. Zhou H-Q, Zhang J-X, Lin S-S. Epidemiological survey of scoliosis in adolescents in Hui 'an County, Fujian Province. Chinese Journal of Spinal cord. 2008(11):824-7.
108. Liang F-M, Liang R-B. An analysis of 96 cases of scoliosis found in DR Chest photos during physical examination of college entrance examination. Clinical medical engineering. 2013;20(6).
109. Zhou S, Z Zhu, X Qiu, et al. Association study of IL-17RC, CHL1, DSCAM and CNTNAP2 genes polymorphisms with adolescent idiopathic scoliosis susceptibility in a Chinese Han population. Studies in health technology and informatics. 2012;176:47-51.
110. Watanabe K, T Michikawa, I Yonezawa, et al. Physical activities and lifestyle factors related to adolescent idiopathic scoliosis. Journal of Bone and Joint Surgery - American Volume. 2017;99(4):284-94.
111. Carneiro KM, D Moreira. Escoliose: perfil postural em escolares do 1º ano do 2º grau do Colégio Objetivo, Brasília. J Health Sci Inst. 2005;23(3).
112. Qi-Deyun LILJ-YZ-Y, LILJ-YZ-Y Qi-Deyun. Abnormal spinal curvature and influencing factors in children and adolescents in Hongkou District, Shanghai. Chinese Journal of School Health. 2021(12):444-7.
113. Wang X-Y, Xu J-D,Qian-M,Gu C. Investigation and analysis of spinal curvature of primary and middle school students in Nanjing. Journal of Nanjing Medical College. 1985(03):209-11+70.
114. Yan ZOU, ZR Hua, CL Yan, et al. Influencing factors for abnormality of the angle of trunk rotation in primary and secondary school students. Journal of Preventive Medicine. 2021(12):462-7.
115. Poussa MS, MM Heliövaara, JT Seitsamo, et al. Development of spinal posture in a cohort of children from the age of 11 to 22 years. Eur Spine J. 2005;14(8):738-42.
116. Yuan D-Y, Li L-P, Jiang Y-W, Zhou Y. Status and influencing factors of abnormal spinal curvature in children and adolescents in Hongkou District of Shanghai. School health in China. 2021;42(03):444-7+53.
117. Shang F-Q, Zheng F-Y. Investigation on physical development of rural children in five prefectural cities of Shanxi Province. Shanxi Med J. 2011;40(02):115-6.
118. Mao S, L Xu, Z Zhu, et al. Association between genetic determinants of peak height velocity during puberty and predisposition to adolescent idiopathic scoliosis. Spine. 2013;38(12):1034-9.
119. Lonstein JE, S Bjorklund, MH Wanninger, RP Nelson. Voluntary school screening for scoliosis in Minnesota. The Journal of bone and joint surgery American volume. 1982;64(4):481-8.
120. Zloof Y, R Ankory, AE Braun, et al. The Hereditary Nature of Adolescent Spinal Deformities: A Study of Over 600,000 Adolescents. Spine. 2022;47(12):841-6.
121. Fang L-G, Zhang B-T, Ma X-C, et al. Early diagnosis of scoliosis by ripple photography. Chinese Journal of Pediatric Surgery. 1986;0(6).
122. Zhang X-M, Li M-S. Investigation and analysis of abnormal spinal curvature of adolescent bone development. Shanxi maternal and child health. 2000;11(3).
123. Qiu XS, NLS Tang, HY Yeung, Y Qiu, JCY Cheng. Association study between adolescent idiopathic scoliosis and the DPP9 gene which is located in the candidate region identified by linkage analysis. Postgrad Med J. 2008;84(995):498-501.
124. Long F, Wei H-L, Liang Y-T, et al. Incidence and risk factors of scoliosis among adolescents in Haidian District in 2015. Public health in China. 2020;36(10):1496-8.

125. He Q, Mu Z-Y, Liu Y-L, Tang Z-B, Qiu L-P. Analysis on detection of common diseases among primary and middle school students in Chuanshan District of Suining City from 2019 to 2021. Occupational and health. 2023;39(09):1243-6+51.

126. Moon ES, HS Kim, V Sharma, et al. Analysis of Single Nucleotide Polymorphism in Adolescent Idiopathic Scoliosis in Korea: For Personalized Treatment. Yonsei Medical Journal. 2013;54(2):500-9.

127. Miyake A, I Kou, Y Takahashi, et al. Identification of a Susceptibility Locus for Severe Adolescent Idiopathic Scoliosis on Chromosome 17q24.3. PLoS One. 2013;8(9):5.

128. Ochsmann EB, CL Escobar Pinzón, S Letzel, et al. Prevalence of diagnosis and direct treatment costs of back disorders in 644,773 children and youths in Germany. BMC Musculoskelet Disord. 2010;11:9.

129. Qin X-H, Zheng W-T. Investigation and analysis of scoliosis among primary school students in XX City. The first Hubei Province Sports Science Conference. 2023.

130. Ge J-L. Epidemiological investigation and intervention of scoliosis (turtle back) in children in a district of Wuhan City.Hubei University of Chinese Medicine.2022.

131. Zhao H. Investigation and analysis of epidemiology of rare diseases in Shandong Province University of Jinan.2014.

132. Shere C, EM Clark. Is musculoskeletal hypermobility associated with adolescent idiopathic scoliosis? A cross-sectional study in the Avon Longitudinal Study of Parents and Children (ALSPAC). JBMR Plus. 2022;6(Supplement 2):48-9.

133. Li B-J. Analysis and evaluation of abnormal spinal curvature of 20826 primary and middle school students in power district of Harbin City. Chinese school doctor. 1992(05):39.

134. Adegoke BOA, AO Akinpelu, BL Taylor. Adolescent idiopathic scoliosis in Ibadan, Nigeria. Internet Journal of Epidemiology. 2011;9(2).

135. Zhou J-W. Investigation on the prevalence of common diseases among students in Zhangjiakou City. Occupational and health. 1996;12(2).

136. Drennan JC, JB Campbell, H Ridge. Denver: a metropolitan public school scoliosis survey. Pediatrics. 1977;60(2):193-6.

137. Wang Z-T, Li Z-S, Wang W-G, et al., editors. A survey on the prevalence of scoliosis among students in 63 medical and health monitoring schools in 12 districts and counties of Beijing. The First International Congress of Chinese Orthopaedic Association; 2006.

138. Li Y, Z Wu, L Xu, et al. Genetic Variant of TBX1 Gene Is Functionally Associated With Adolescent Idiopathic Scoliosis in the Chinese Population. Spine. 2021;46(1):17-21.

139. Huang Y, Fan A-P. Surveillance of key common diseases among primary and secondary school students in Tai 'an City in 2019. Preventive Medicine Forum. 2021;27(9).

140. Zhang GH, LX Chen, X Chen, et al. [Prevalence and influencing factors of abnormal spinal curvature in primary and secondary school students in Shandong Province in 2020]. Zhonghua yu fang yi xue za zhi [Chinese journal of preventive medicine]. 2023;57(11):1839-42.

141. Li-Shangle Z-QR-X. Abnormal spinal curvature in primary and secondary school students in Tibet. Chinese Journal of School Health. 2021(12):1384-7.

142. Waneerat Galassi MD. A Retrospective Prevalence Study of Scoliosis in Thai Adolescents. 2010.

143. Lam TP, VWY Hung, HY Yeung, et al. Adolescent Idiopathic Scoliosis (AIS) is associated with deranged bone quality - A case-control study using quantitative ultrasound on 636 AIS subjects and 269 normal controls. Bone. 2009;45(Suppl. 2):S62.

144. Saglam Y, I Bingöl, NE Yasar, et al. The burden of scoliosis: a nationwide database study on demographics, incidence, and surgical rates. Eur Spine J. 2024;33(2):655-62.

145. Kamtsiuris P, K Atzpodien, U Ellert, R Schlack, M Schlaud. Prevalence of somatic diseases in German children and adolescents. Results of the German Health Interview and Examination Survey for Children and Adolescents (KiGGS). Bundesgesundheitsblatt-Gesund. 2007;50(5-6):686-700.

146. Heine J, F Heine. [On the incidence of thoracal scoliosis in juveniles (author's transl)]. Uber die Haufigkeit der Thorakalskoliose bei Jugendlichen. 1981;119(4):340-3.

147. Jeon KK, DI Kim. Low body mass index levels and idiopathic scoliosis in korean children: A cross-sectional study. Children. 2021;8(7):570.

148. Gecheva-Fermendzhieva GY. COMPARATIVE ANALYSIS OF THE INCIDENCE OF SPINAL DISTORTIONS IN ADOLESCENTS FOR THE PERIOD 2009-2021. Journal of IMAB - Annual Proceeding (Scientific Papers). 2023;29(2):4932-4.
149. Atia DT, NI Elsayed, AF Abdelmonem, et al. Prevalence of Musculoskeletal Disorders among General and Technical Secondary School Students in Egypt. Int J Environ Res Public Health. 2023;20(2):11.
150. Qiao J-J, Chen-H, Han-X, et al. Prevalence of scoliosis among primary and middle school students in Dongcheng District, Beijing in 2021. Capital Public Health. 2023;17(04):240-3.
151. Kulis A, D Zarzycki, J Jaskiewicz. Concentration of estradiol in girls with idiophatic scoliosis. Ortopedia Traumatologia Rehabilitacja. 2006;8(4):455-9.
152. Li S-Y. Prevalence and influencing factors of abnormal spinal curvature among primary and secondary school students in Shandong Province in 2020. Chin J of Preventive Medicine. 2023;57(11).
153. Li S-L, Zhang Q, Rong X. Analysis of abnormal spinal curvature of primary and secondary school students in Xizang region. School health in China. 2021;42(09):1384-7.
154. Ropac D, I Stasevic, D Samardzic, Z Mijakovic. Spinal deformities among pupils - A growing issue. Collegium Antropologicum. 2013;37(SUPPL.2):139-45.
155. Heffernan MJ, T Lucak, F Lindsey, et al. Rates of scoliosis screening are low and appear related to school classification. Pediatrics. 2018;141(1).
156. Chen Z-J, Qiu Y, Wang B, Yu-Y, Zhu Z-Z. The predictive value of Matrilin-1 gene polymorphism in the progression of idiopathic scoliosis in adolescents. Chin J of Orthopedics. 2009(5).
157. Daruwalla JS, P Balasubramaniam, SO Chay, U Rajan, HP Lee. Idiopathic scoliosis. Prevalence and ethnic distribution in Singapore schoolchildren. Journal of Bone and Joint Surgery - Series B. 1985;67(2):182-4.
158. Mulu A, A Gissila, M Jegnie, F Minichil. The Prevalence of Scoliosis among Adolescent Chest Radiographs Obtained at Tikur Anbessa Specialized Hospital in 2019. Ethiopian journal of health sciences. 2023;33(4):641-8.
159. Li W-P, Wang Z-Y, Huang J-R, et al., editors. Investigation on the prevalence of scoliosis in adolescents in Guangzhou in different periods. The First International Congress of Chinese Orthopaedic Association; 2006.
160. Gozdzialska A, J Jaskiewicz, M Knapik-Czajka, et al. Association of Calcium and Phosphate Balance, Vitamin D, PTH, and Calcitonin in Patients With Adolescent Idiopathic Scoliosis. Spine. 2016;41(8):693-7.
161. Herzog J, D Chan, N Patel, et al. First presentation adolsecent idiopathic scoliosis in the UK. A case for school screening. Glob Spine J. 2018;8(1 Supplement 1):110S.
162. Han X, L Zhen, Z Zhu, et al. Polymorphism of rs2767485 in leptin receptor (LEPR) gene is associated with the occurrence of adolescent idiopathic scoliosis. Spine Journal. 2015;15(10 Supplement 1):185S.
163. Group. Application of heavy plumb line principle scoliosis measuring instrument in screening adolescent scoliosis in Nanchang area. 2010-09-07.
164. Bueno RdCdS, RR Rech. Desvios posturais em escolares de uma cidade do Sul do Brasil. Rev paul pediatr. 2013;31(2):237-42.
165. Guo HB, N Chen, YQ Yang, et al. Ethnic Disparity in the Incidence of Scoliosis Among Adolescents in Tianzhu Tibetan Autonomous County, China. Front Public Health. 2022;10:10.
166. He R. Analysis of current situation and influencing factors of scoliosis in primary and secondary school students in Anshan City. Chinese Medical Sciences University.2023.
167. Baidoo NAB, J Quartey, KP Essuman, JE Armah, DP Asamoah. Association between bag weight, carrying style and low back pain and spinal curvatures among school children in ablekuma south. Physiotherapy (United Kingdom). 2015;101(SUPPL. 1):eS1243.
168. Fary R, D Hopper, B Longworth. Prevalence and Predictors of Adolescent Idiopathic Scoliosis in Adolescent Ballet Dancers Response. Arch Phys Med Rehabil. 2015;96(6):1181-2.
169. Zhang G-B. Census measurement and treatment of scoliosis. J of med research. 1997;31(10).
170. Lai Z-H. Investigation on spinal curvature of 7516 primary and secondary school students in Shaoguan and Zhanjiang of Guangdong Province. Guangdong health and epidemic prevention data. 1982(01):99-105.

171. Leal JS, MCPdS Leal, CER Gomes, MDC Guimarães. Inquérito epidemiológico sobre escoliose idiopática do adolescente. Rev bras ortop. 2006;41(8):309-19.
172. Lee CF, DYT Fong, KMC Cheung, et al. Referral Criteria for School Scoliosis Screening Assessment and Recommendations Based on a Large Longitudinally Followed Cohort. Spine. 2010;35(25):E1492-E8.
173. Clark EM, JH Tobias. Association between physical activity and scoliosis: A prospective cohort study. Journal of Musculoskeletal Neuronal Interactions. 2018;18(1):120.
174. Leone A, A Aulisa, C Perisano, T Re, M Galli. Advantages of a two-step procedure for school-based scoliosis screening. La Radiologia medica. 2010;115(2):238-45.
175. Zhu Z, W Zhang, Z Liu, X Sun, Y Qiu. The prevalence of intraspinal anomalies in infantile and juvenile patients with presumed idiopathic scoliosis: A MRI-based analysis of 504 patients. Eur Spine J. 2015;24(6 SUPPL. 1):S728-S9.
176. Stolinski L, T Kotwicki. Trunk asymmetry in one thousand school children aged 7-10 years. Studies in health technology and informatics. 2012;176:259-63.
177. Yin S-H, Zou Y-N, Zhang J-Z. Epidemiological survey of occult and idiopathic scoliosis in young people of appropriate age in Nanhua County.Chin modern doctor. 2010(24).
178. Li G, Zeng L-H, Song-J. Distribution characteristics of scoliosis among adolescents in Huizhou area. Heilongjiang traditional Chin med. 2021;50(06):171-2.
179. Sun Y-F, Zheng Z-J, Sun L-J, Wu Y-L. Abnormal spinal curvature and poor visual acuity of primary and middle school students in Caofeidian District of Tangshan City. Henan J of Preventive Med. 2021;32(08):637-9.
180. Chen-Jingjing SUNXLIW. Spinal curvature and associated factors among middle school students in the Yangtze River Delta Region. Chinese Journal of School Health. 2023(12):135-8.
181. Janusz P, M Kotwicka, M Andrusiewicz, et al. Estrogen receptors genes polymorphisms and age at menarche in idiopathic scoliosis. BMC Musculoskelet Disord. 2014;15:383.
182. Walker AP, RA Dickson. School screening and pelvic tilt scoliosis. Lancet (London, England). 1984;2(8395):152-3.
183. Nikolova S, V Yablanski, E Vlaev, A Savov, I Kremensky. Association between a common variant near LBX1 and idiopathic scoliosis in Bulgarian population. Eur J Hum Genet. 2019;26(Supplement 1):870.
184. Lin X-P, Zhang-Q. Analysis of health examination status of children aged 4 -16 in Shenzhen. Nobile Hospital. 2022;22(02):283-5.
185. Beausejour M, M Roy-Beaudry, L Goulet, H Labelle. Patient characteristics at the initial visit to a scoliosis clinic: a cross-sectional study in a community without school screening. Spine. 2007;32(12):1349-54.
186. Sun WD, JJ Li, JL Zhou, et al. Meeting the Canadian 24-Hour Movement Guidelines and physical-mental comorbidity among Chinese children and adolescents: Prevalence, associations, and the population impacts. J Psychosomat Res. 2024;176:10.
187. Li S. Investigation and application of prevention and control of common diseases among students -- taking Lanzhou City as an example. 2022-05-13.
188. Grivas TB, K Koukos, UI Koukou, C Maziotou, BD Polyzois. The incidence of idiopathic scoliosis in Greece--analyais of domestic school screening programs. Studies in health technology and informatics. 2002;91:71-5.
189. Wang H, Z Wu, Q Zhuang, et al. Association study of tryptophan hydroxylase 1 and arylalkylamine n-acetyltransferase polymorphisms with adolescent idiopathic scoliosis in han chinese. Spine. 2008;33(20):2199-203.
190. Zhang J. Prevalence of scoliosis and its correlation with BMI in primary and secondary school students in Shenyang. Chinese Medical Sciences University.2023.
191. Ma X-Y, Zhang X-H, Song-Y, et al. Prevalence trend and related factors of scoliosis and myopia in middle school students in Inner Mongolia from 2019 to 2022. School health in China. 2023;44(09):1289-93.
192. Zhao J-F, Wei N-N, Zhao J, et al. Abnormal spinal curvature of primary and secondary school students in Hohhot City. School health in China. 2023;44(02):295-8.
193. Wu D-H. Survey and prevention of scoliosis in primary and secondary school students. Chinese school doctor. 1996;10(5).
194. Vercauteren M, M Van Beneden, R Verplaetse, et al. Trunk asymmetries in a Belgian school population. Spine. 1982;7(6):555-62.

195. Ji XR, ZD Yang, XH Yang, et al. Change of selenium in environment and risk of adolescent idiopathic scoliosis: a retrospective cohort study. Eur Rev Med Pharmacol Sci. 2013;17(18):2499-503.
196. Se-Il SUK, C In-Ho. The Incidence of Scoliosis in Korea Part II : The Incidence of Scoliosis in the Middle and High School Male Students. The Journal of the Korean Orthopaedic Association. 1978:317-23.
197. Liu Z, NLS Tang, XB Cao, et al. Lack of association between the promoter polymorphisms of MMP-3 and IL-6 genes and adolescent idiopathic scoliosis: A case-control study in a chinese han population. Spine. 2010;35(18):1701-5.
198. Singh H, Shipra, V Sharma, et al. The first study of epidemiology of adolescent idiopathic scoliosis shows lower prevalence in females of Jammu and Kashmir, India. Am J Transl Res. 2022;14(2):1100-6.
199. Bremberg S, B Nilsson-Berggren. School screening for adolescent idiopathic scoliosis. Journal of pediatric orthopedics. 1986;6(5):564-7.
200. Qin X, L Xu, C Xia, et al. Genetic Variant of GPR126 Gene is Functionally Associated With Adolescent Idiopathic Scoliosis in Chinese Population. Spine. 2017;42(19):E1098-E103.
201. Anasheva D, G Gabdullina. The morbidity of the dorsal spine of 1-st year students of astana medical university and measures of its prophylaxis. Annals of Anatomy. 2014;196(SUPPL. 1):33-4.
202. Wang J-J, Li Z-H, Chen Q-P, Tao M-T. Abnormal spinal curvature of primary and secondary school students. Henan J of Preventive Med. 1982(03):97-106.
203. Ma-Xinyue Z-XS-YD-YY-TMACGAOS. Prevalence trend and related factors of scoliosis and myopia co-morbidities among primary and middle school students in Inner Mongolia Autonomous Region from 2019 to 2022. Chinese Journal of School Health. 2023(12):1289-93.
204. Fu G-B, Tang S-P. Pathogenesis and pathogenic factors of idiopathic scoliosis in adolescents. J of Clinical Pediatric Surgery. 2006(05):361-4.
205. Bellyei A, A Czeizel, O Barta, T Magda, L Molnar. Prevalence of adolescent idiopathic scoliosis in Hungary. Acta Orthop Scand. 1977;48(2):177-80.
206. Jia Q-Z. Genetic epidemiological investigation of AIS and comparative analysis of SH3GL1 sequence in Chongqing, China.Third Military Medical University,2008.
207. Soucacos PK. School screening for scoliosis, the Greek experience. Scoliosis. 2010;5(SUPPL. 1).
208. Döhnert MB, E Tomasi. Validity of computed photogrammetry for detecting idiopathic scoliosis in adolescents. Rev Bras Fisioter. 2008;12(4):290-7.
209. Khalchitsky SE, MV Sogoyan, AN Filippova, et al. Folate cycle and interleukin 6 genes polymorphisms in children with idiopathic scoliosis and its relation with pathogenesis of disease: A case control study. Journal of Clinical and Diagnostic Research. 2019;13(11):RC01-RC5.
210. Ramli MS. 5 Years Experience Of School Scoliosis Screening Program In Perak Population - A Clinical Evaluation Of Epidemiology, Effectiveness And Limitation Of Scoliosis Screening From 2011 To 2015. Malaysian Orthopaedic Journal. 2018:169-.
211. Chen L-P, Ma P, Wu J. Dynamic analysis and countermeasures of physical health status in primary and secondary schools in Nanjing for 15 years -- a new concept of common diseases in children and adolescents in Nanjing. The 6th National Academic Exchange Meeting of Children's Health Branch of China Preventive Medicine Association and the 3rd Academic exchange meeting of School branch of China Health Education Association; 2004.
212. Ding S-S. Screening of scoliosis in primary and secondary school students by fluoroscopy. Chin J of Med Imaging. 2005(06):72.
213. Wen W-H. Investigation and analysis of health examination results of primary and secondary school students in Changshu City.Suzhou University.2013.
214. Huang S-L, Zhang F-Y, Qu X-S, et al. Status and related factors of common diseases in middle school students in Shanghai in 2021. School health in China.1-6.
215. Zhang R-M. Surveillance and analysis of five diseases among primary and secondary school students in Shandong Province. Public health in Chin. 1990(04):165-6.

216. Adamczewska K, M Wiernicka, E Malchrowicz-Mosko, J Malecka, J Lewandowski. The angle of trunk rotation in school children: A study from an idiopathic scoliosis screening. prevalence and optimal age screening value. Int J Environ Res Public Health. 2019;16(18):3426.
217. Xuan Z, D Qing, N Stefano, C Peijie. An epidemiological study on the prevalence rate of scoliosis from local area. PM and R. 2014;6(8 SUPPL. 2):S90.
218. Tan C-M, Lu B-H, Ruan Q, et al. Survey report of poor vision, trachoma, dental caries and scoliosis in 33079 primary and secondary school students in Guangxi in 1987. Guangxi med. 1988(05):314-5.
219. Tan M-N. Investigation on abnormal spinal curvature of primary and secondary school students in Guangzhou. Guangzhou Med. 1984(05):21-3+13.
220. Bulkees N, N Rajakanthan, N Perera, et al. 'ADOLESCENT IDIOPATHIC SCOLIOSIS of CHILDREN'. PREVALENCE, ITS ASSOCIATIONS & CO-MORBIDITIES and the ACCURACY of NON-RADIOLOGICAL SCREENING METHODS. Arch Dis Child. 2023;108(Supplement 2):A188.
221. Grivas TB, A Arvaniti, C Maziotou, MM Manesioti, A Fergadi. Comparison of body weight and height between normal and scoliotic children. Studies in health technology and informatics. 2002;91:47-53.
222. Minghelli B, W Rodrigues, V Barreto, et al. Prevalence of scoliosis in adolescent in South of Portugal. Atencion Primaria. 2013;45(SUPPL. 2):140.
223. Yao J-X. A survey of students' health status in Liaoning Province. J of Chin Med University. 1987(S1):85-7+93.
224. Zhang J-X. Epidemiological investigation of adolescent primary scoliosis and six-year treatment follow-up. Guangdong health & epidemic prevention data. 1986(02):49-52.
225. Wen J-L, Qiu Y, Chen Z-J, et al. Association between insulin-like growth factor-1 receptor gene polymorphisms and with susceptibility to adolescent idiopathic scoliosis. Chinese Journal of Surgery. 2009(12):1813-6.
226. Qiu XS, LS Deng, XE Yang, ZY Zheng, Y Qiu. Genetic polymorphism of growth hormone gene in adolescent idiopathic scoliosis. Zhonghua wai ke za zhi [Chinese journal of surgery]. 2008;46(22):1741-3.
227. Yang-Tian Z-XGAOSLIGZ-JZ-J. Abnormal spinal curvature and its influencing factors among middle and high school students in Inner Mongolia Autonomous Region. Chinese Journal of School Health. 2023(12):447-50.
228. Chen L, Xu X-W. Current situation and related factors of common diseases among primary school students in Cixi City in 2020. Health studies. 2022;42(4).
229. Ren Q-Q, Zhou L, Chen J-Y. Current situation and influencing factors of abnormal spinal curvature in primary and secondary school students in Sichuan Province. Jiangsu preventive med. 2023;34(03):360-2.
230. Chen S-S, Zhou-X, Li X, et al. Screening and risk factors analysis of scoliosis in middle school students in Kaiyuan city, Yunnan Province. J of Educational biology. 2024;12(01):14-8+25.
231. Hazebroekkampschreur A, A Hofman, AP Vandijk, B Vanlinge. PREVALENCE OF TRUNK ABNORMALITIES IN 11-YEAR-OLD SCHOOLCHILDREN IN ROTTERDAM, THE NETHERLANDS. J Pediatr Orthop. 1992;12(4):480-4.
232. Zhao X-F, Li C, Liu Y, Zeng Y, Yi G-F. The relationship between physical activity sedentary behavior and body posture health in primary school students. School health in China. 2022;43(8).
233. Safikhani Z, M Fakor, H Soori, L Hejazian. The study of scoliosis and its relationship with the length of extremities in girl students of Guidance School in Ahwaz, Iran. Pakistan Journal of Medical Sciences. 2005;21(4):470-1.
234. Arienti C, R Buraschi, S Donzelli, et al. Trunk asymmetry is associated with dominance preference: results from a cross-sectional study of 1029 children. Braz J Phys Ther. 2019;23(4):324-8.
235. Assiri A, AA Mahfouz, NJ Awadalla, AY Abolyazid, M Shalaby. Vitamin D deficiency and clinically detected scoliosis among maladolescents at high-altitude area in Southwestern Saudi Arabia. Open Access Macedonian Journal of Medical Sciences. 2020;8(E):213-8.
236. Eksi MS, EE Ozcan-Eksi, SE Huet, et al. Prevalence of Thoracic Scoliosis in Adolescents in Turkey: Analysis of 1065 Chest Radiographs. World Neurosurg. 2020;135:e527-e40.

237. Wang W-R, Liu-X, Wu P-Q, et al., editors. Study on the incidence of idiopathic scoliosis in adolescents with home isolation/online learning. The 13th National Convention on Sport Science of China; 2023.
238. Taylor HJ, IJ Harding, IW Nelson, JH Tobias, EM Clark. Use of a novel low-radiation method to assess prevalence of spinal curvature in a large population based cohort. Eur Spine J. 2012;21(SUPPL. 2):S231-S2.
239. Dong-Gune C, KIM Gang-Un, SUK Se-Il, et al. Prevalence of Thoracic Scoliosis in Koreans Using Simple Chest Radiography. Journal of Korean Society of Spine Surgery. 2019:56-62.
240. Lev Ran R, B Knishkowy, B Adler. Screening physical examinations in 25,000 Israeli schoolchildren. International Journal of Adolescent Medicine and Health. 2013;25(1):47-53.
241. Jae-Chul YOO, SUH Seung-Woo, J Bok-Ja, et al. Asymmetric Exercise and Scoliosis: A Study of Volleyball Athletes. The Journal of the Korean Orthopaedic Association. 2001:455-60.
242. Baba MR, RM Shenoy, A Soman. School based screening for idiopathic scoliosis in premenarcheal girls: A pilot study. Indian Journal of Public Health Research and Development. 2020;11(3):2259-64.
243. Zhou-W, Cai Y-B, Lu Y-J. Investigation and influencing factors of scoliosis among primary and middle school students in Jiangbei District of Ningbo City. Health studies. 2024;44(02):143-7.
244. Bai Y-Q, Li-S, Yang-L, Wang J-Y. Investigation on spinal curvature of primary and middle school students in Lanzhou from 2020 to 2021. Gansu Sci & Tech. 2023;52(03):88-91.
245. Zhao-Jufang WEINZ-JY-TLIGGAOJZ-X. Spinal curvature disorders among primary and middle school students in Hohhot. Chinese Journal of School Health. 2023(12):295-8.
246. Huang Z-H, Chen L-R, Zhang-Y, Shui-L, Cui-L. Analysis of scoliosis detection results in primary and secondary school students in Kunming City. Hainan Med. 2016;27(14):2390-1.
247. Koga Y. [The result of the author's screening system of scoliosis in elementary and junior-high schools]. Nihon Seikeigeka Gakkai zasshi. 1986;60(1):61-71.
248. Kratenova J, K Zejglicova, M Maly, V Filipova. Risk factors and prevalence of bad posture in school-age children. Prakticky Lekar. 2005;85(11):629-34.
249. Kuroki H, T Nagai, E Chosa, N Tajima. School scoliosis screening by Moire topography - Overview for 33 years in Miyazaki Japan. Journal of orthopaedic science : official journal of the Japanese Orthopaedic Association. 2018;23(4):609-13.
250. Adamczewska K, M Wiernicka, E Kaminska, et al. Annual Observation of Changes in the Angle of Trunk Rotation. Trunk Asymmetry Predictors. A Study from a Scoliosis Screening in School Adolescents. Int J Environ Res Public Health. 2020;17(6):9.
251. Chang-Hyun OH, KIM Chan-Gyu, LEE Myoung-Seok, et al. Usefulness of Chest Radiographs for Scoliosis Screening: A Comparison with Thoraco-Lumbar Standing Radiographs. Yonsei Medical Journal. 2012:1183-9.
252. Banerjee A, S Ilankathir. Prevalence of Scoliosis among School going Children in Puducherry. Journal of Clinical and Diagnostic Research. 2022;16(SUPPL 2):88.
253. Lazic I, IP Markovic, SS Antunovic, et al. Influence of physical activity on prevention and occurrence of spinal deformities in children during development. Vojnosanit Pregl. 2021;78(7):730-5.
254. Normand E, A Franco, S Parent, et al. Association between the GLP1R A316T Mutation and Adolescent Idiopathic Scoliosis in French Canadian and Italian Cohorts. Genes. 2024;15(4):481.
255. Se-Il SUK, LEE Young-Goo, CHO Hyoun-Oh, JO Jeong-Hyeon, C Jang-Seuk. The Prevalence of Scoliosis in Junior and Senior High School Students, Pusan, Korea. The Journal of the Korean Orthopaedic Association. 1984:431-5.
256. Jeon K, DI Kim. The Association between Low Body Weight and Scoliosis among Korean Elementary School Students. Int J Environ Res Public Health. 2018;15(12):7.
257. Zhao J, Z-YGAOSY-TZ-JLIGBATZ-X. Status of scoliosis and the related factors among 12 year old school students from Inner Mongolia Autonomous Region in 2021. Chin J of School Health. 2023(12):1399-402.
258. Pedrotti L, R Mora, B Bertani, G Tuvo, I Crivellari. Association among postural and skull-cervico-mandibular disorders in childhood and adolescence. Analysis of 428 subjects. La Pediatria medica e chirurgica : Medical and surgical pediatrics. 2007;29(2):94-8.

259. Francis RS. Scoliosis screening of 3,000 college-aged women. The Utah study - Phase 2. Phys Ther. 1988;68(10):1513-6.
260. Fu T-Y. Correlation analysis of common diseases and complex factors among primary and secondary school students in a city. Chengdu Medical College. 2022.
261. Inoue M, S Minami, Y Nakata, et al. Association between estrogen receptor gene polymorphisms and curve severity of idiopathic scoliosis. Spine. 2002;27(21):2357-62.
262. Nikolova ST, VT Yablanski, EN Vlaev, et al. Association Between IL-6 and MMP3 Common Genetic Polymorphisms and Idiopathic Scoliosis in Bulgarian Patients: A Case-control Study. Spine. 2016;41(9):785-91.
263. Zhao QH, ZZ Zhu, Y Qiu, et al. [Association between tartrate-resistant acid phosphatase 5 gene polymorphism and adolescent idiopathic scoliosis]. Zhonghua wai ke za zhi [Chinese journal of surgery]. 2012;50(1):66-9.
264. Tzivian JL, MA Nikolsky, GM Korzhavin. Deformities of the spine and maldevelopments of the thorax in schoolchildren. Ortopediya Travmatologiya i Protezirovanie. 1978;No. 4:16-9.
265. Sadler B, G Haller, L Antunes, et al. Distal chromosome 16p11.2 duplications containing SH2B1 in patients with scoliosis. J Med Genet. 2019;56(7):427-33.
266. Andrade Barcia A, J Andrade Barcia. Escoliosis estructural idiopática y otras alteraciones esqueléticas en escolares de los niveles primario y secundario de la ciudad de portoviejo. Educ méd contin. 1996(53):17-24.
267. Yeung HY, NL Tang, KM Lee, et al. Genetic association study of insulin-like growth factor-I (IGF-I) gene with curve severity and osteopenia in adolescent idiopathic scoliosis. Studies in health technology and informatics. 2006;123:18-24.
268. Liu S, N Wu, Y Zuo, et al. Genetic Polymorphism of LBX1 Is Associated with Adolescent Idiopathic Scoliosis in Northern Chinese Han Population. Spine. 2017;42(15):1125-9.
269. Xu L, C Xia, W Sun, et al. Genetic Polymorphism of NUCKS1 Is Associated With the Susceptibility of Adolescent Idiopathic Scoliosis. Spine. 2017;42(21):1629-34.
270. Zengyu W, Z Yuechuan, P Yue, et al. Genetic Polymorphisms of SPRY4 are Associated with Adolescent Idiopathic Scoliosis in Chinese Han Population : A Single Center Retrospective Study. Medical Journal of Peking Union Medical College Hospital. 2023;14(3):553-8.
271. Xu L, C Xia, X Qin, et al. Genetic variant of BNC2 gene is functionally associated with adolescent idiopathic scoliosis in Chinese population. Molecular Genetics and Genomics. 2017;292(4):789-94.
272. Nelson LM, K Ward, JW Ogilvie. Genetic variants in melatonin synthesis and signaling pathway are not associated with adolescent idiopathic scoliosis. Spine. 2011;36(1):37-40.
273. Wu Z, Z Dai, W Yuwen, et al. Genetic Variants of CHD7 Are Associated with Adolescent Idiopathic Scoliosis. Spine. 2021;46(11):E618-E24.
274. Paricio Talayero JM, L Santos Serrano, A Fernandez Feijoo, et al. Health examination of children from the Democratic Sahara Republic (northwest Africa) on vacation in Spain. Anales Espanoles de Pediatria. 1998;49(1):33-8.
275. Negri V. [Incidence of scoliosis in adolescents of the schools of Parma]. Incidenza della scoliosi negli adolescenti delle scuole di Parma. 1982;53(1):41-5.
276. Tisovsky P, P Deco, L Rehak, et al. Incidence of trunk abnormalities in children aged 8-14 years in Bratislava. Lekarsky Obzor. 2004;53(9):338-40.
277. Xia C, B Xue, Y Wang, et al. Investigating Role of IRX Family in Development of Female Adolescent Idiopathic Scoliosis: Which One Is Real Cause? World Neurosurg. 2019;127:e132-e6.
278. Xu L, S Huang, X Qin, et al. Investigation of the 53 Markers in a DNA-Based Prognostic Test Revealing New Predisposition Genes for Adolescent Idiopathic Scoliosis. Spine. 2015;40(14):1086-91.
279. Takahashi Y, M Matsumoto, T Karasugi, et al. Lack of association between adolescent idiopathic scoliosis and previously reported single nucleotide polymorphisms in MATN1, MTNR1B, TPH1, and IGF1 in a Japanese population. Journal of orthopaedic research : official publication of the Orthopaedic Research Society. 2011;29(7):1055-8.
280. Zhu F, J Qiao, XS Qiu, et al. Lack of association between suppressor of cytokine signaling-3 gene polymorphism and susceptibility and curve severity of adolescent idiopathic scoliosis. Eur Spine J. 2014;23(11):2432-6.

281. Qiu X-S, F Lv, Z-Z Zhu, et al. Lack of association between the CHL1 gene and adolescent idiopathic scoliosis susceptibility in Han Chinese: a case-control study. BMC Musculoskelet Disord. 2014;15:38.
282. Qiu XS, NLS Tang, HY Yeung, JCY Cheng, Y Qiu. Lack of association between the promoter polymorphism of the MTNR1A gene and adolescent idiopathic scoliosis. Spine. 2008;33(20):2204-7.
283. Qiu XS, NLS Tang, HY Yeung, et al. Melatonin receptor 1B (MTNR1B) gene polymorphism is associated with the occurrence of adolescent idiopathic scoliosis. Spine. 2007;32(16):1748-53.
284. Hitesh M, S S, S Satyen, et al. Muscle Imbalance in Volleyball Players Initiates Scoliosis in Immature Spines: A Screening Analysis. Asian Spine Journal. 2008:38-43.
285. Nikolova S, M Dikova, D Dikov, et al. Positive association between a polymorphic locus near the LBX1 gene and predisposition of idiopathic scoliosis in Southeastern European population. J Appl Biomed. 2019;17(3):184-9.
286. Yuan P, ZH Wang, H Jiang, et al. Prevalence and plasma exosome-derive microRNA diagnostic biomarker screening of adolescent idiopathic scoliosis in Yunnan Province, China. Front Pediatr. 2024;12:1308931.
287. Del Castillo Campos MJ, JJ Ramos Alvarez, O Antelo Suarez, MT Lara Hernandez. Prevalence of scoliosis in young elite athletes of Madrid. Archivos de Medicina del Deporte. 1997;14(58):119-25.
288. Tisovsky P, P Deco, L Rehak, et al. Prevalence of trunk abnormalities in children aged 8-14 years in Bratislava. Lekarsky Obzor. 2004;53(9):341-3.
289. Pratelli E, L Apicella, B Bertaccini, et al. Results of a vertebral deformity screening in the students of the district of Florence (Tuscany Region, Central Italy). Epidemiol Prev. 2020;44(2-3):154-61.
290. Akram O S. School screening for scoliosis in Amman. 1986. p. 219-27.
291. Alsharbati MM, TS Alchalabi, SF Rummani. SCHOOL SCREENING FOR SCOLIOSIS IN BAGHDAD. Saudi Med J. 1993;14(4):298-302.
292. Weisz I, G Volpin, V Bialik, J Fishman, H Stein. School screening for symptomless spinal deformities. Medical Science Research. 1988;16(14):729-30.
293. Nguyen Huu C. Scoliosis status and its risk factors in pupils at Hai Phong City. Journal of Vietnamese Medicine. 2004:167-75.
294. Wu Z-Y, Zhang-YC, Peng Y, et al. SPRY4 genetic polymorphisms are associated with idiopathic scoliosis in adolescents in a Chinese Han population: a single-center retrospective study. Union Med J. 2023;14(3).
295. Ascani E, V Salsano, G Giglio. The incidence and early detection of spinal deformities. A study based on the screening of 16,104 schoolchildren. Italian journal of orthopaedics and traumatology. 1977;3(1):111-7.
296. Wilczynski J. The most common faulty postures among boys aged 13-16 years measured by Moire's photogrammetric method. Med Pr. 2006;57(4):347-52.
297. Biggi F, A Romano. Tracking results and considerations in diagnosis of school age scoliosis. Chirurgia Italiana. 1980;32(5 Suppl. 1):18-24.
298. Deng N, Chen Q, Yu B, Zhou Z, Shao Y. A preliminary investigation report of scoliosis in primary and middle school students in Chengdu. The First International Congress of Chinese Orthopaedic Association; 2006.
299. Chen JS, Yang F, Wang JB, et al. An epidemiological survey of scoliosis among middle and primary school students in Xi 'an City. jilin med J. 2015;36(13):2783-4.
300. Garg B, N Mehta, S Shekhar, T Bansal. 16. Incidence of intraspinal anomalies in congenital and other subtypes of idiopathic scoliosis: a radiological insight based on 320 scoliosis patients. Spine Journal. 2023;23(9 Supplement):S8-S9.
301. Huang H. Epidemiological analysis of 91 cases of scoliosis in children and adolescents. Weekly Digest Pension Weekly. 2023(24).
302. Zhang C, YF Zhang, LD Wang, et al. A Comparative Study of Sagittal Spinal-Pelvic Parameters Between Patients with Adolescent Idiopathic Scoliosis and Healthy Controls. Int J Morphol. 2020;38(2):415-22.
303. Eun-Su M, M Seong-Hwan, LEE Hwan-Mo, et al. A Comparison of Bone Mineral Density between Adolescent Idiopathic Scoliosis and Neuromuscular Scoliosis. Journal of Korean Society of Spine Surgery. 2007:17-24.

304. Min K, Y Li, Z Wu, et al. A Genetic Variant of FAM46A is Associated With the Development of Adolescent Idiopathic Scoliosis in the Chinese Population. Spine. 2023;48(17):1253-8.
